# Urinary metabolome dynamics in ^13^C-labeled mice

**DOI:** 10.1007/s11306-025-02391-4

**Published:** 2025-12-29

**Authors:** Annelaure Damont, Anaïs Legrand, Kathleen Rousseau, Laurent Bellanger, Séverine Boiry, Frédéric Gibiat, Jean-Jacques Leguay, Christophe Junot, François Fenaille, Eric Ezan

**Affiliations:** 1 Paris-Saclay University, CEA, INRAE, Département Médicaments et Technologies pour la Santé (DMTS), MetaboHUB, F-91191 Gif sur Yvette, France; 2 Paris-Saclay University, CEA, INRAE, Département Médicaments et Technologies pour la Santé (DMTS), F-30200 Bagnols-sur-Cèze, France; 3 Aix Marseille University, CEA, CNRS, BIAM, F-13115 Saint Paul-Lez-Durance, France

**Keywords:** ^13^C-metabolomics, Isotope tracing, In vivo, Mammal labeling, Mass spectrometry

## Abstract

**Introduction:**

Analysis of specific pathways of metabolic flux is usually achieved by administering stable isotope-labeled precursors and measuring their incorporation into selected metabolites by high-resolution mass spectrometry coupled to liquid chromatography (LC-HRMS).

**Objectives:**

In this study, we undertook a novel approach aiming at covering the whole metabolome dynamics by providing mice with a diet fully enriched in carbon-13 (^13^C).

**Methods:**

Three animals were fed for six weeks with small pellets composed of a mixture of ^13^C-spirulina and ^13^C-wheat. All animals grew normally and their urine was collected daily. Three control mice were treated in the same way, but with an unenriched diet. LC-HRMS-based metabolomics profiling were conducted on all collected samples as well as isotope tracing.

**Results:**

Comparative LC-HRMS analysis of the ^12^C- and ^13^C-samples unambiguously identified 238 metabolites, whose ^13^C-labeling profiles were then studied over a 39-day period. Although overall urine labeling was fast and rapidly reached a high level of ^13^C-content (> 90% after 22 days), isotopic monitoring of each molecular species demonstrated that ^13^C-incorporation kinetics are considerably variable between metabolites, reflecting their biological function, origin and rate of biosynthesis in vivo.

**Conclusion:**

This study demonstrates that mice tolerate a diet entirely labeled with carbon-13 (> 97 atom % ^13^C) over a period of six-weeks. It reveals the dynamics of a large part of the metabolome at the mammalian species level through urine analysis coupled to in vivo ^13^C-labeling. The study provides valuable insights by comprehensively covering metabolic pathways, and stands out from targeted fluxomic studies which are carried out by administering a very limited number of specific labeled precursors.

**Supplementary Information:**

The online version contains supplementary material available at 10.1007/s11306-025-02391-4.

## Introduction

Biochemical exploration of metabolite and protein dynamics can provide insights into homeostasis and its disruption following disease, nutritional, or pharmacological intervention (Reisz and D’Alessandro 2017; Y. Wang et al. 2020). The combination of stable isotope tracers with sensitive analytical techniques such as NMR and high-resolution mass spectrometry (HRMS) has significantly advanced this field through the analysis of both cells and whole organisms (Arroo et al. 2021; Bednarski et al. 2021; Kim et al. 2020; Kobak et al. 2022; Lehmann 2017; Rolfs et al. 2021; Salvador et al. 2022; Y. Wang et al. 2020). For instance, it has been demonstrated in 2017, through the administration of labeled nutrients to mice followed by HRMS detection, that glucose fuels the tricarboxylic acid cycle via circulating lactate, thereby showing that ATP production can be decoupled from glucose catabolism (Hui et al. 2017).

Metabolite tracers are labeled versions of endogenous compounds that undergo biochemical transformation identical to their unlabeled counterparts in a given biological environment. They usually consist in compounds incorporating deuterium (^2^H), carbon-13 (^13^C) or nitrogen-15 (^15^N) atoms. Labeled lactate, glucose, propionate, palmitate or glutamine are easily available and constitute substrates of choice for the study of central metabolic pathways since they can be chemically resolved by HRMS through their mass shift from their unlabeled counterparts. However, one of the associated limitations is that they mainly target central metabolism (Laro et al., 2025) or specific macromolecules biosynthesis and cannot cover complex metabolic networks over time whereas metabolite biosynthesis may originate from multiple precursors (Kim et al., 2022). Therefore, isotope-assisted exploration of the entire metabolome has been restricted to cellular models and remains a challenge for the study of whole organisms where multiple biochemical pathways are highly connected in space and in time. A first option would be to combine as many labeled precursors as existing biochemical pathways, but this is not affordable given the limited number of commercially available precursors. Another approach is to investigate the metabolism of an organism using a “whole-food” diet enriched with stable isotopes (e.g., ^2^H, ^13^C, ^15^N, ^18^O). A major concern with heavy isotopes is their impact on metabolism and animal’s survival. For example, deuterium (^2^H or D), often administered to animals as D_2_O in drinking water, is poorly tolerated at concentrations above 20% of body weight, as it significantly affects enzyme kinetics, thereby reducing growth and viability (Hodel et al., 1982; Kasumov et al., 2013; Kushner et al., 1999; Thomson, 1960; Wiberg, 1955). Although some species can survive higher levels (De Carli et al., 2021; Kampmeyer et al., 2020), deuterium is not ideal for achieving high molecular pool enrichment without toxic effects. In contrast, carbon-13 is ubiquitous in bio-organic molecules and has minimal isotopic effects *in vivo.* An early study showed that mice fed a 80% ^13^C-labeled chow for eight months remained healthy and resulted in significant tissue labeling, although molecular-level analyses were limited at the time (Gregg et al., 1973). Nitrogen-15 was also found to be safe for small mammals. For instance, rats fed for seven weeks with ^15^N-labeled algal cells and spirulina survived and reached > 90% whole-body ^15^N-enrichment, with extracted tissues used as reference standards for protein quantification (Wu et al., 2004). Such approaches laid the foundation for the SILAM (Stable Isotope LAbeling in Mammals) strategy for quantitative proteomics (Rauniyar et al., 2013; Wilkinson, 2018). The approach has also been used to study proteome dynamics in mouse, interspecies protein turnover and phenotypic alterations in pathological mice (Filiou et al., 2012; Liu et al., 2020; Swovick et al., 2018, 2021). A more recent study showed that a diet based on ^13^C-labeled bacteria in mice provided labeled plasma and tissues that could serve as internal standards for mass spectrometry-based metabolomics (Dethloff et al., 2018).

Here, we intend to provide a holistic exploration of the metabolome turnover in mice fed a fully labeled chow. We provided a ^13^C-enriched diet of uniformly labeled (> 97% ^13^C) wheat and spirulina to mice for 39 days and monitored the level of ^13^C incorporation into urinary metabolites as a reflection of the kinetics of the whole metabolism. Analysis of the metabolome was performed by high-resolution mass spectrometry coupled to liquid chromatography (LC-HRMS). Its annotation was performed, after detection of ^12^C/^13^C ion pairs for biological compound validation, by comparing mass spectra (MS1) and fragmentation spectra (MS2) with an internal database of more than 10,000 spectra (gathered from approx. 1200 compounds). Fully labeled animals grew normally until the end of the experiment, allowing metabolite labeling kinetics to be obtained through the gradual replacement of initial ^12^C by ^13^C in organic metabolites, thanks to MS-based carbon isotopolog analysis. This proof-of-concept study addresses the labeling dynamics of ~ 250 metabolites in a mammalian species. It highlights differential metabolite turnovers, with fast isotope exchanges reflecting central biochemical pathways, while slow isotope exchanges illustrate release from deep storage compartments.

## Methods

### In vivo ^13^C-labeling and sample collection

**Fig. 1:**
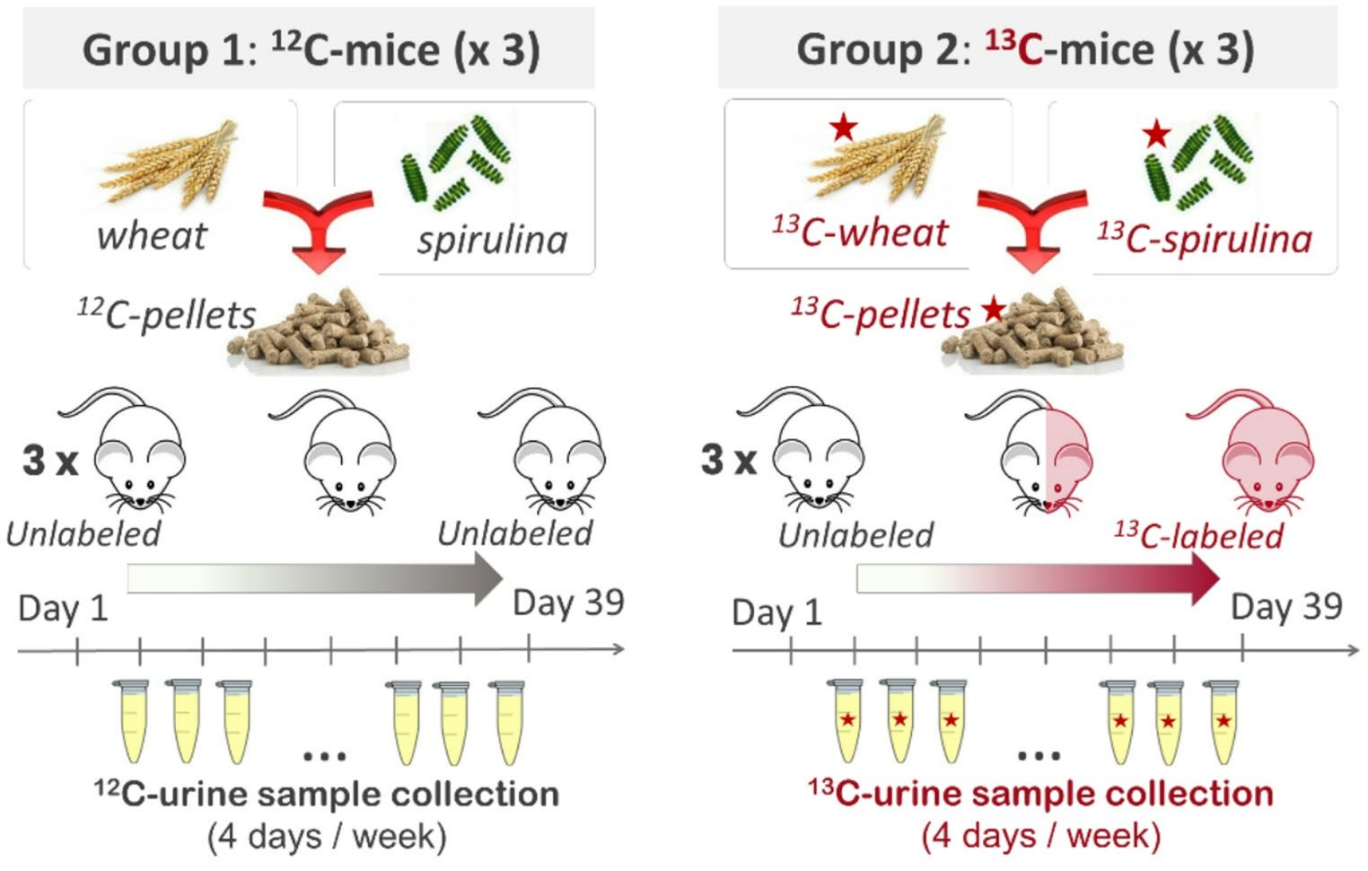
In vivo ^13^C-labeling protocol. Group 1 represent the control group and Group 2 consists of producing three ^13^C-labeled mice

**Fig. 2:**
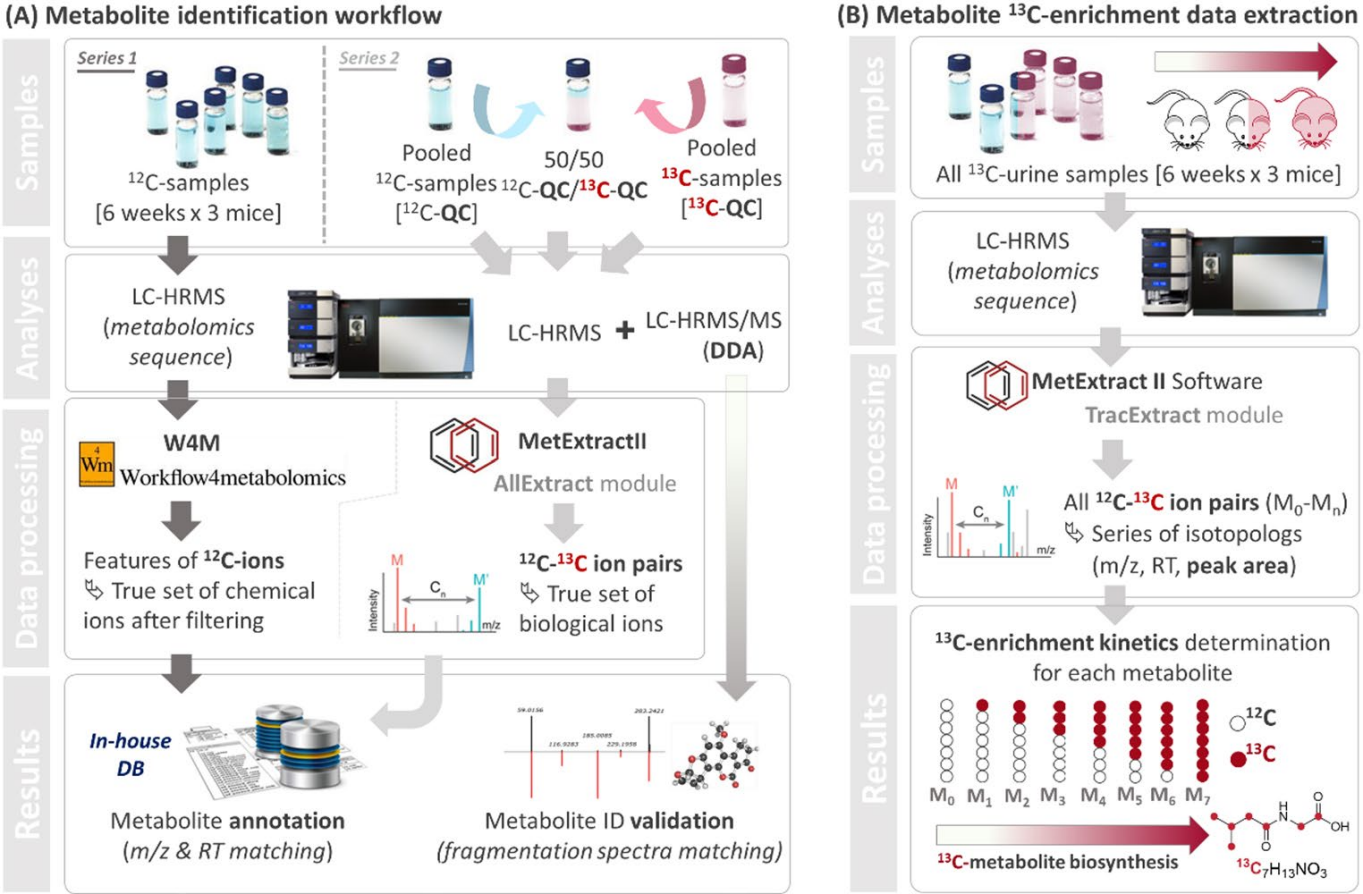
Non-targeted workflows applied for the LC-HRMS analysis and data pre-processing of the different mice urine samples. **A** W4M and MetExtract II workflows used for metabolite identification and full characterization; **B** Workflow specifically applied for the ^13^C-enrichment data extraction using MetExtract II as software, on all ^13^C-labeled mice urine samples

A group of three mice was fed a ^13^C-labeled diet consisting of a 78:22 (w/w) mixture of in-house produced ^13^C-labeled wheat and commercially available ^13^C-labeled spirulina with isotopic enrichment > 98%, as measured by isotope ratio mass spectrometry, IRMS (experimental details are described in the Supplementary file1). For the ^13^C-labeled wheat production, durum wheat was sown and grown in an air-tight chamber allowing for the injection of 99% pure atomic ^13^C as [^13^C]CO_2_ from the start of the crop (André et al., 1985; Matulova et al., 2005). The wheat was grown for six months, and ^13^C-labeling was measured at 97.2% in the 370 g of grain produced. As depicted in Fig. 1, a control group of mice (Group 1), also comprising three individuals, was monitored (Supplementary file2, FigS1) and fed the same diet in its natural non-enriched version as the above-mentioned group of mice (Group 2). Mice urine from Group 1 and Group 2 were collected four days a week for six weeks which represents a total collection of 51.1 mL of labeled urine and an average volume per animal per day of almost 0.7 mL. Elemental analysis of isotopic carbon in all urine samples from group 2 were measured by the National Analysis Service AQui (Montpellier, France, Supplementary file2, FigS2). The urine samples were aliquoted in batches of 100 µL and stored at −80 °C prior to LC-HRMS analysis.

### Sample preparation and LC-HRMS profiling of mouse urine

5 µL of each urine sample was diluted in 45 µL of a 95:5 (v/v) H_2_O/CH_3_CN mixture containing 0.1% formic acid. The resulting samples were centrifuged at 20,000 g for 15 min at 4 °C. The supernatants were transferred into vials for injection and LC-HRMS analyses. Quality control sample (QC) preparation of ^12^C, ^13^C and mixed ^12^C/^13^C-urines is detailed in the Supplementary file1. For the LC-HRMS acquisitions, chromatographic separation was carried out on a Dionex Ultimate 3000 system (Thermo Fisher Scientific) equipped with a C18 Hypersil Gold column 2.1 × 150 mm, 1.9 μm, 175 Å (Thermo Fisher Scientific). It was coupled to an Orbitrap™ Tribrid™ Fusion™ (Thermo Fisher Scientific, Courtaboeuf, France) equipped with a heated electrospray ionization source operated in the positive or negative mode at a high resolution of 240,000 (at *m/z* 200) for HRMS profiling and at a resolution of 30,000 for MS/MS acquisitions in data-dependent acquisition mode (DDA). Detailed elution conditions and mass spectrometer settings are reported in the Supplementary Information (Supplementary file1).

### Data processing

**Fig. 3:**
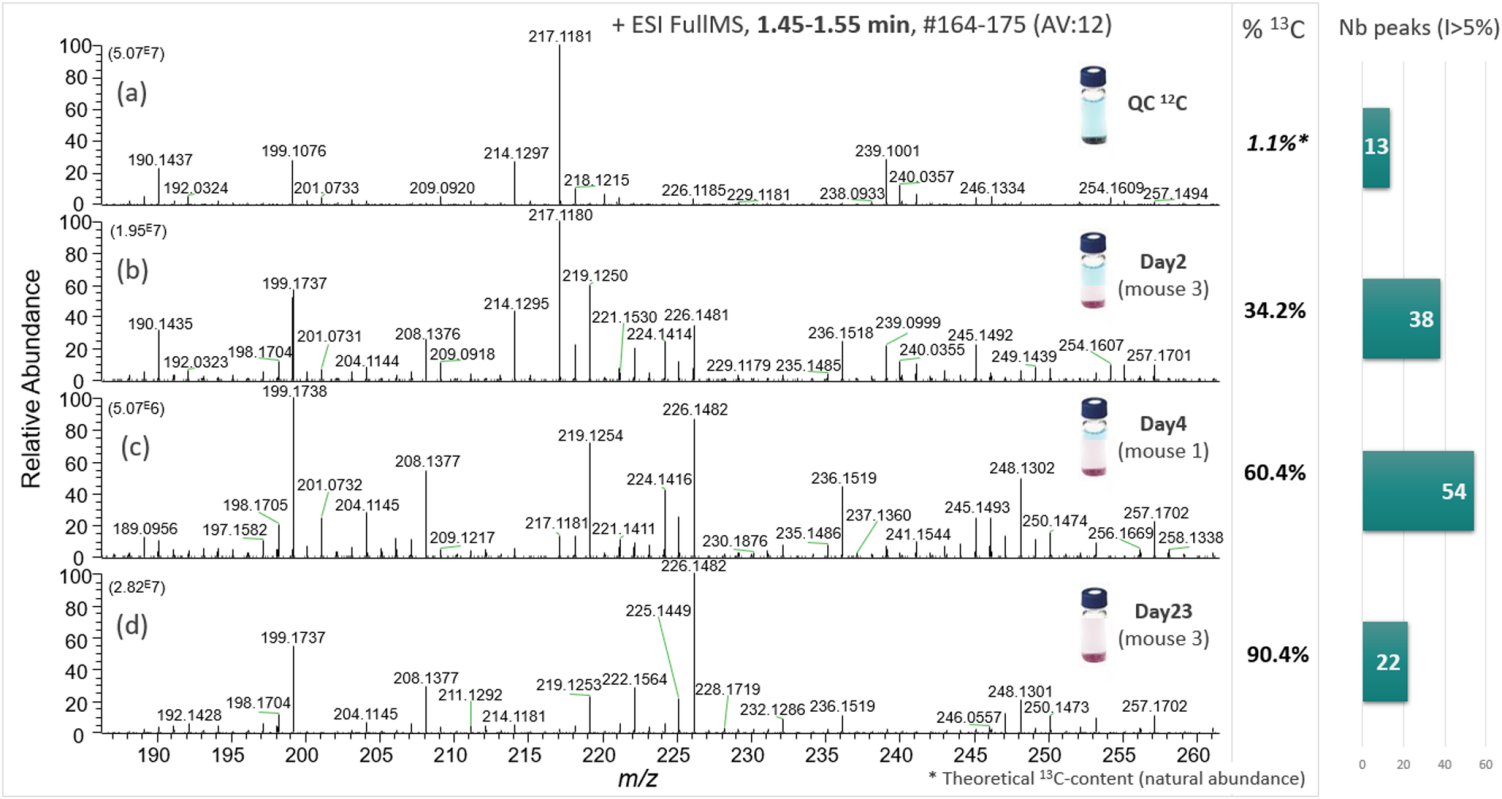
Overview of LC-HRMS data as a function of the ^13^C-enrichment stage. Mass spectra obtained in the positive ion mode for four different samples in the retention time window 1.45–1.55 min zoomed in the *m/z* range 186–262. **a** quality control sample from ^12^C-mice from group 1; **b** sample collected on day 2 from ^13^C-mouse 3; **c** sample collected on day 4 from ^13^C-mouse 1; **d** sample collected on day 23 from ^13^C-mouse 3. The ^13^C-fraction in the sample, expressed in %, is indicated on the right-hand side of each mass spectra (data obtained from the elemental analysis, Supplementary file2, FigS2). The number of mass spectral peaks with a relative intensity greater than 5% detected for each of the four samples is also reported

All the data were processed using open access softwares. In brief, LC-HRMS.raw data were first converted into centroid mzxml files using MSConvert. Two specific workflows, shown in Fig. 2, were applied depending on whether the data was enriched or not.^12^C-sample data were processed using a dedicated classical metabolomics workflow on the Workflow4Metabolomics (W4M) platform (Giacomoni et al., 2015). ^12^C-QC, ^13^C-QC, mixed ^12^C/^13^C-QC and daily ^13^C-sample data were processed using the MetExtract II open access software (Bueschl et al., 2017) dedicated to the extraction of unlabeled/labeled ion pairs (Supplementary File2, FigS3). The full isotopic distribution of target metabolites were extracted from the daily ^13^C-samples LC-HRMS profiles at each time point with the TracExtract module of MetExtract II. The data processing parameters applied are reported in the Supporting Information (Supplementary File1). The MetExtract software does not automatically take into account the natural abundance of ¹³C. Authentic isotopic profiles, as appearing in mass spectra, were reported as histograms for each identified metabolite in the Supplementary File4. These isotope distributions were then used to calculate the total ^13^C-content at each time point (see Sect. 2.5 below).

### Metabolite identification

**Fig. 4:**
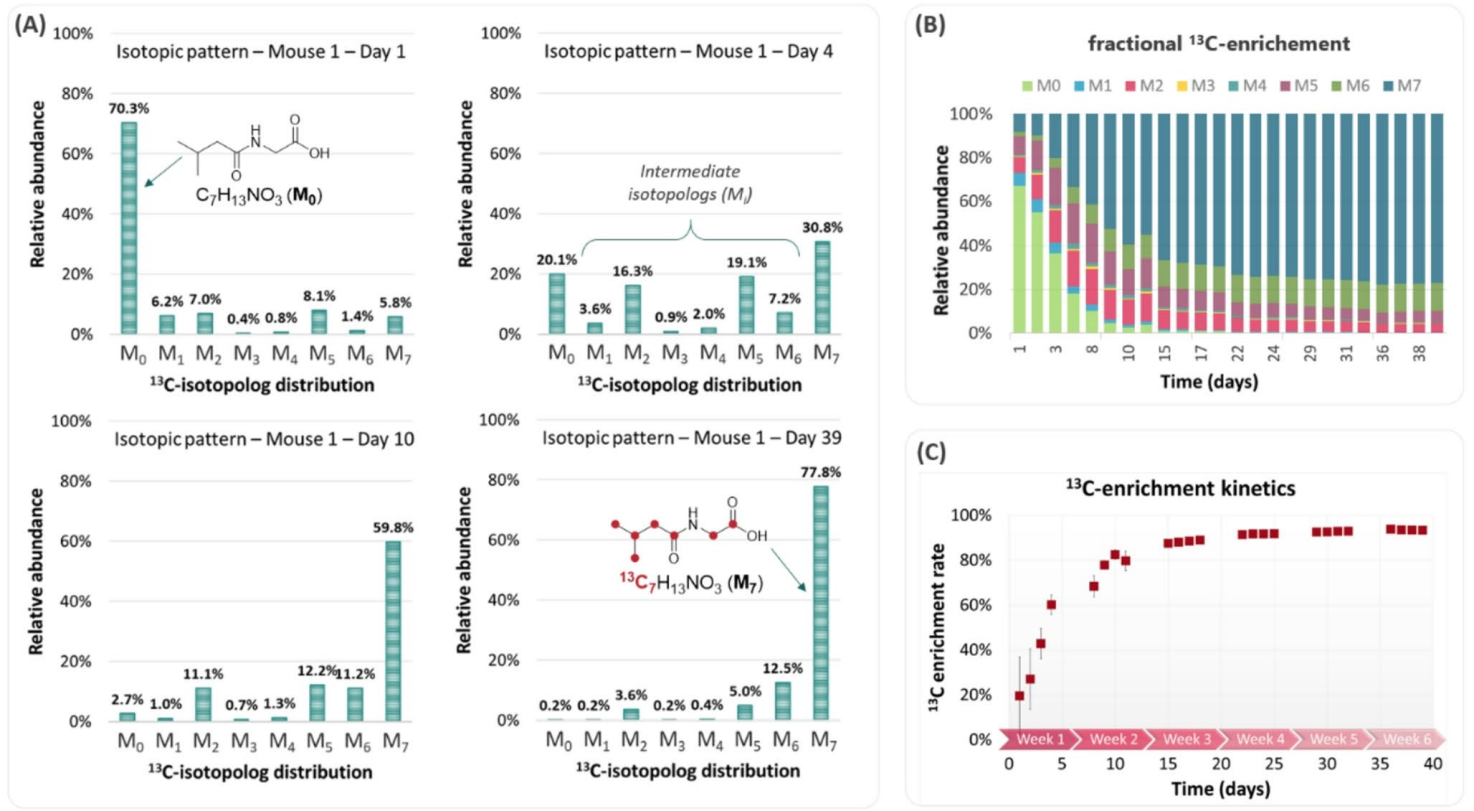
^13^C-enrichement data in ^13^C-labeled mice urine and ^13^C-metabolite kinetics measurement. **A** Reconstruction of four isotopic patterns obtained with the TracExtract data for isovaleroylglycine in the ^13^C-urine sample of mouse 1 at day 1, day 4, day 10 and day 39 during the in vivo ^13^C-enrichement process; **B** Fractional ^13^C-enrichement for isovaleroylglycine over the 39 days (average results for the 3 mice); **C** ^13^C-enrichement kinetic curve obtained for isovaleroylglycine (average results for the 3 mice)

**Fig. 5:**
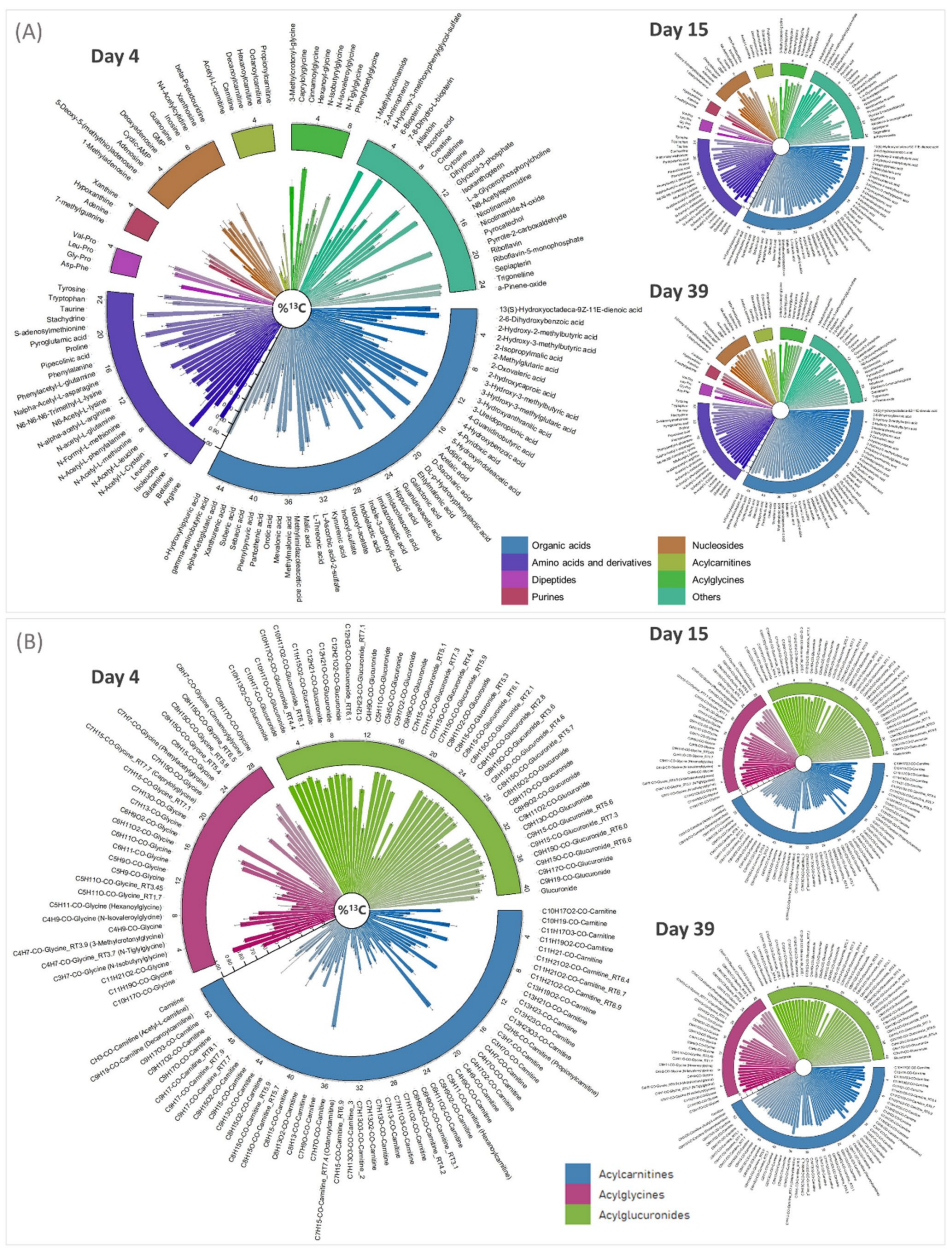
Circos plot view of the identified molecular species ^13^C-content. **A** representation for the 128 “level 1” identified metabolites at early (day 4), intermediate (day 15) and final (day 39) times and, **B** representation for the acyl-derivatives at early, intermediate and final times

*Level 1 identification*. N°44: (Sumner et al., 2007) the W4M variable dataset and the unlabeled ions of the MetExtract II variable dataset were annotated, based on their *m/z* and retention time values, using an in-house spectral library that includes more than 1,200 metabolites (Boudah et al., 2014). Unambiguous identifications were then validated by comparing the fragmentation data to those of the authentic standards. The ^13^C-labeled ion was also checked to confirm carbon stoichiometry and, when available, the fragmentation profile was manually compared to the unlabeled one and annotated with elemental composition (the example of pantothenic acid identification is presented in the Supplementary file2, FigS4A). *Level 2 identification*. An in silico library of three different chemical families, namely acyl-carnitines, acyl-glucuronides and acyl-glycines, was designed based on acyl-species already encountered in human urine (Yan et al., 2020). Based on their elemental composition (chemical formula), their [M + H]^+^ and [M-H]^−^ *m/z* values were calculated and used as an in-house database for annotating the unlabeled LC-HRMS dataset obtained from the profiling of group 1 mice (Supplementary file3, Table S1). Identification validation of the matching signals was achieved by checking in the fragmentation spectra the presence of fragment ions and neutral losses characteristic of each chemical family which are presented in the Supplementary file2, FigS5.

### ^13^C-enrichment calculation and ^13^C-labeling kinetic curves

The daily ^13^C-enrichment rate (i.e., the proportion of carbon-13 in the total amount of carbon into the molecule) could be calculated from each isotopic distribution and for each identified metabolite, according to the following equation.

$$\:\mathrm{\%}{}^{13}C={\sum\:}_{i=0}^{n}\frac{i}{n}\times\:{I}_{i}$$

Where *i* is the number of ^13^C atoms in the isotopolog *M*_*i*_, *n* is the total number of carbon atoms in the molecular structure and *I*_*i*_ is the relative intensity of each isotopolog *M*_*i*_ in the isotopic distribution. ^13^C-labeling kinetic curves were plotted by reporting the ^13^C-content (%^13^C) as a function of time (39 days) for each molecule.

## Results

### Design of in vivo ^13^C-labeling and general outcome

Three mice were fed for six weeks with small pellets composed of a mixture of ^13^C-wheat and ^13^C-spirulina (Fig. 1). The latter component of the pellets was selected based on its exceptional nutritional content, composed of proteins (50–75%), lipids (5–10%), carbohydrates (10–20%) and other essential nutrients among which vitamins (e.g., B1, B2, B12, Folic acid), phytopigments (e.g., carotenoids) and minerals (e.g., Ca, K, Fe, Se) (Abreu et al., 2023; Shioji et al., 2021). The limited quantity of ^13^C-wheat produced defined the number of mice to be included in the protocol, to be able to feed the animals for six weeks and thus obtain sufficient ^13^C-enrichment to monitor the kinetics of metabolite labeling over time. This time period was based on a review of the literature (Gregg et al., 1973; Wu et al., 2004), which demonstrates the possibility of slow turnover of certain metabolites. Since repeated blood sampling over such an extended duration was clearly not feasible without compromising animal welfare, our initial objective was to characterize the kinetics of ¹³C-labeling in mice urine over time. Besides, all organs and blood were collected at the end of the study (upon sacrifice of the animals) and stored at −80 °C for further studies. Note that urine is representative of the whole-body metabolism, except for those compounds that are not filtered by the kidney. Lipids fall into this category, but we expected a number of polar compounds (carnitine, glucuronic acid, etc.) to be excreted in the urine after conjugation with fatty acids, mirroring part of the lipid metabolism.

In this experiment, we first confirmed that carbon-13 labeling had no apparent impact on the health of mice as demonstrated by comparing phenotypes and characteristics of the ^13^C-mice group (Group 2, Fig. 1) to a group of control mice (Group 1, Fig. 1). For instance, in Group 2, the mice did not show any abnormal behaviour nor loss of weight (Sup file2, FigS1). Secondly, the level of carbon-13 incorporation, measured by elemental analysis of isotopic carbon in all urine samples from Group 2, showed a rapid increase since it reaches a steady level > 90% already after 22 days. It indicates an excellent global incorporation of carbon-13 into metabolites or small peptides filtered by the kidney (Supplementary file2, FigS2).

In order to obtain a more detailed view of the ^13^C-content at the molecular level, the collected urine samples were then analyzed by high-resolution mass spectrometry coupled to liquid chromatography (LC-HRMS).

### Profiling of mouse urinary ^12^C- and ^13^C-metabolomes

LC-HRMS analysis of ^13^C-enriched mouse urine samples provided much more complex profiles than unlabeled urine, due to the progressive incorporation of carbon-13 into metabolites. Figure 3 shows how spectral complexity increases with time: mass spectra taken at four different time points during the labeling process show how the content of carbon-13 increases from 1.1% (no labeling) to 34.2% (day 2), 60.4% (day 4) and 90.4% (day 23). This multiplication of signals reflects the presence of additional ^13^C-isotopic analogs (isotopologs). Visual inspection of the mass spectrum in Fig. 3 indicates that, for some enriched samples, the number of mass peaks with a relative intensity greater than 5% compared with the base peak can more than quadruple compared with the natural sample (i.e., ^12^C-QC). At the same time, as the number of peaks increases, the absolute intensity of each peak decreases (i.e., signal dilution).

For non-targeted analysis, such a multiplication of signals requires the implementation of efficient data processing workflows adapted to the extraction and grouping of ^12^C- and ^13^C-signals derived from the same metabolite. Thus, two untargeted analytical and pre-processing workflows were set up (Fig. 2) and evaluated for their ability to (1) extract, identify and characterize, both with ^12^C and ^13^C-data, urinary metabolites in mice; and (2) to decipher their ^13^C-enrichment kinetics over time through the analysis of their carbon isotopologs.

### Specific identification of biologically relevant metabolite features via extraction of ^12^C/^13^C-ion pairs

Following a first workflow (Fig. 2A), two distinct series of samples were prepared to conduct LC-HRMS(/MS) experiments. Series 1 consisted of all Group 1 samples and, as such, included ^12^C-urine samples at each day of the collection process for each ^12^C-mouse of the control group (i.e., 72 samples). Series 2 consisted of three mixes: (1) a mix of pooled ^12^C-urine samples (^12^C-QC); (2) a mix of pooled ^13^C-urine samples (^13^C-QC); and (3) an equivolumic mixture of the two previous QCs (^12^C/^13^C). Urinary metabolites were separated using a reverse phase column and detected with a high-resolution Orbitrap^™^ Tribrid^™^ Fusion^™^ mass spectrometer. Both mass spectra and fragmentation spectra were collected for the different samples for individual and mixed ^12^C- and ^13^C-pooled QC samples. Then, two different suites of software tools were used to extract the LC-HRMS signals in an untargeted manner. W4M (Giacomoni et al., 2015) was used, as traditionally conducted in metabolomics, to extract signals from the ^12^C-sample LC-HRMS profiles. While MetExtract II, specifically designed for isotopic data extraction (Bueschl et al., 2017), was used to process the second set of acquisitions that included ^13^C-labeled ions. The latter software is a combination of bioinformatics tools assembled to allow the detection of ^12^C/^13^C ion pairs. In this series, we used the AllExtract module of the software, which only retains extreme ion pairs (Supplementary file2, FigS3) (Bueschl et al., 2017). The main difference between these two extraction methods is that in one case, the processing gives a set of robust ions, whereas in the second case, the detection of ion pairs guarantees their biological origin. In fact, not only does the detection of ^12^C/^13^C ion pairs exclude all unlabeled species (e.g., contaminants, artifacts) but also, when metabolite labeling is complete, it enables carbon number determination. Table 1 summarizes the number of features extracted according to the data extraction method and the ionization mode. The filtration steps applied to the extracted features in the end of the W4M process is used to retain only analytically robust variables (Supplementary file1).

**Table 1:** Number of extracted features according to the data processing protocol

Extracted features	Samples	Positive ion mode	Negative ion mode
W4M – No filters	Series 1	9016	11,662
W4M – Robust features	Series 1	5401	7388
AllExtract ^12^C/^13^C-ion pairs	Series 2	2046	1710

The use of an isotope-assisted processing workflow enabled the detection of 2,046 ^12^C/^13^C-ion pairs in the positive ion mode (Supplementary file3, Table S2) and 1,710 in the negative ion mode (Supplementary file3, Table S3), all of them being associated to ions derived from biologically relevant compounds as evidenced by ^13^C-incorporation.

The lower number of signals obtained with AllExtract can be mainly explained by the fact that: (1) unlabeled species and notably chemical contaminants or exogenous compounds are eliminated; (2) isotopic dilution in series 2 leads to lower feature detection sensitivity. At this stage, signal redundancy remains since adduct ions and in-source fragments were not filtered out. Thus, considering that one single metabolite can produce more than 10 signals under ESI conditions (Brown et al. 2009), a rough calculation indicates that our data from ^13^C-labeled mice support previously reported data from ^13^C-labeled bacteria and yeast indicating that less than 10% of features detected by LC-HRMS correspond to unique metabolites (Mahieu and Patti 2017; L. Wang et al. 2019).

### Metabolomic landscape of mouse urine

After annotation of the variable dataset obtained with both W4M and MetExtract II workflows, metabolite identification was fully confirmed with ^12^C- and ^13^C-ion fragmentation data provided by the MS/MS experiments (see Methods and Supplementary file2, FigS4A). In total, 76 metabolites were identified in the positive ion mode and 84 in the negative ion mode either with W4M or AllExtract as data processing tool (Supplementary file2, FigS4Ba). Only 32 metabolites could be identified in both ionization modes (Supplementary file2, FigS4Bb), giving a total of 128 unique metabolites unambiguously identified and referred to as “level 1”-identified according to the Sumner metabolite description scale (Sumner et al., 2007). These compounds (1–128) are listed in Table S4 (Supplementary file3) along with their identifiers (HMDB, CheBi and KEGG IDs, etc.) and corresponding LC-MS data (*m/z* unlabeled, *m/z* labeled, retention time, intensity). More than half of them correspond to organic acids or amino acids and derivatives. Their chemical class repartition is presented in Supplementary file2, FigS4Bc.

To extend metabolic coverage to less polar compounds, and in particular to study short- and medium-chain fatty acid derivatives, three chemical families - acyl-carnitines, acyl-glucuronides and acyl-glycines - were considered. These compounds are well resolved on reverse phase columns and were consistently detected across the samples. Since only a few representatives of each class was available as standards, they were identified first on the basis of their *m/z* values in relation to their generic chemical formula (Supplementary file3, Table S1), and then based on their typical fragmentation pattern (Supplementary file2, FigS5) (Guo et al., 2022; Yan et al., 2020). Thus, formal identification was achieved using the fragmentation data generated from the MS/MS experiments on ^12^C-QCs and ^13^C-QCs. Indeed, acyl-carnitine fragmentation gives characteristic product ions at *m/z* 85.0284 and 60.0808 in the positive ion mode (*m/z* 89.0418 and 63.0908, in ^13^C-labeled version), with neutral losses of either 59.0735 Da corresponding to NMe_3_, or 77.0840 reflecting the same loss plus water (*m/z* 62.0836 and 80.0941, in ^13^C-labeled version) (Yan et al., 2020). In the case of acyl-glycines, characteristic fragment ions are found at *m/z* 76.0390 in the positive mode and *m/z* 74.0247 in the negative mode (*m/z* 78.0460 and 76.0315 respectively in ^13^C-labeled version) with a typical neutral loss of 75.0320 Da in both modes corresponding to the release of the glycine moiety (*m/z* 77.0387 for ^13^C_2_-glycine). For acyl-glucuronides, *m/z* 175.0248, 113.0244 and 85.0295 are characteristic product ions obtained in the negative ionization mode (*m/z* 181.0450, 118.0412 and 89.0429 respectively in ^13^C-labeled version), along with a typical neutral loss of 176.0326 Da corresponding to the glucuronide moiety (*m/z* 182.0528 for ^13^C_6_H_8_O_6_) (Supplementary file2, FigS5) (Guo et al., 2022). Based on these typical fragmentation behaviours, 55 acyl-carnitines, 29 acyl-glycines and 40 acyl-glucuronides were identified and are listed in the supplementary material (Supplementary file3, Table S5) with their respective *m/z* ratio and retention time. They are referred to as “level 2” identified metabolites, as no correspondence with an available authentic standard could be established (Schymanski et al., 2014), with the exception of fourteen which had already been identified and listed as level 1 (N-Isobutyrylglycine **51**, Carnitine **56**, 3-Methylcrotonyl-glycine **63**, N-Isovaleroylglycine **65**, N-Tiglylglycine **66**, Hexanoyl-glycine **73**, Acetyl-carnitine **78**, Propionylcarnitine **107**, Phenylacetylglycine **108**, Capryloylglycine **111**, Cinnamoylglycine **116**, Hexanoylcarnitine **120**, Octanoylcarnitine **124** and Decanoylcarnitine **126**). The latter molecules are reported again in this second table (Supplementary file3, Table S5) to facilitate inter-comparison within the studied chemical families. In conclusion of the identification part, 128 metabolites were level 1 identified (**1**-**128**) and additionally, 110 level 2 acyl-metabolites were found and characterized in ^12^C and ^13^C-mice urine (**129**–**238**).

### ^13^C-labeling kinetics

Following the workflow depicted in Fig. 2B, carbon isotopolog ions of all identified metabolites could be extracted. As an example, Fig. 4A displays the isotopic distribution of isovaleroylglycine at four time points. M_0_ represents the full ^12^C-ion and M_7_ the full ^13^C-labeled ion. We observed the formation of two main intermediate isotopologs, M_2_ and M_5_, during the enrichment process (day 4 and day 10). These intermediates reflect the biosynthesis of the molecule: M_2_ corresponds to ¹³C₂-glycine combined with unlabeled isovaleroic acid, while M_5_ corresponds to ¹³C₅-isovaleroic acid combined with unlabeled glycine. Interestingly, the contribution of isotopolog M_6_ remains significant and almost constant beyond day 10 (~ 11 to 13% from day 10 to day 39). The corresponding isotopolog ^12^C^13^C_6_H_13_NO_3_ represents the carbon-12 contribution originating in part from the labeled diet (> 97% ^13^C). Graphs presenting the average fractional ^13^C-content (for 3 mice) were also constructed to give a more compact view of each isotopolog contribution over time (Fig. 4B as an illustration for isovaleroylglycine). This information highlights the stepwise labeling of some identified metabolites. Additionally, after calculating the daily ^13^C-enrichment rate (see the Methods section for a formula) for each identified metabolite, their ^13^C-labeling kinetic curves were plotted over the 39 days (Fig. 4C as an illustration for isovaleroylglycine). This process was applied to the 238 formally identified metabolites including acyl-glycines, acyl-carnitines and acyl-glucuronides. For some compounds whose isotopic ions were not sufficiently intense to be detected automatically, a manual search was carried out and the data collected at five different times (day 1, day 4, day 15, day 25 and day 39). This information is included in the Supplementary file3, Tables S6 and S7 as a summary of the ^13^C-content of each 238 mice urine metabolites at early (day 4), intermediate (day 15 and day 25) and final times (day 39). Individual enrichment kinetics sheets are available for each level 1 identified metabolite in the Supplementary file4.

**Fig. 6:**
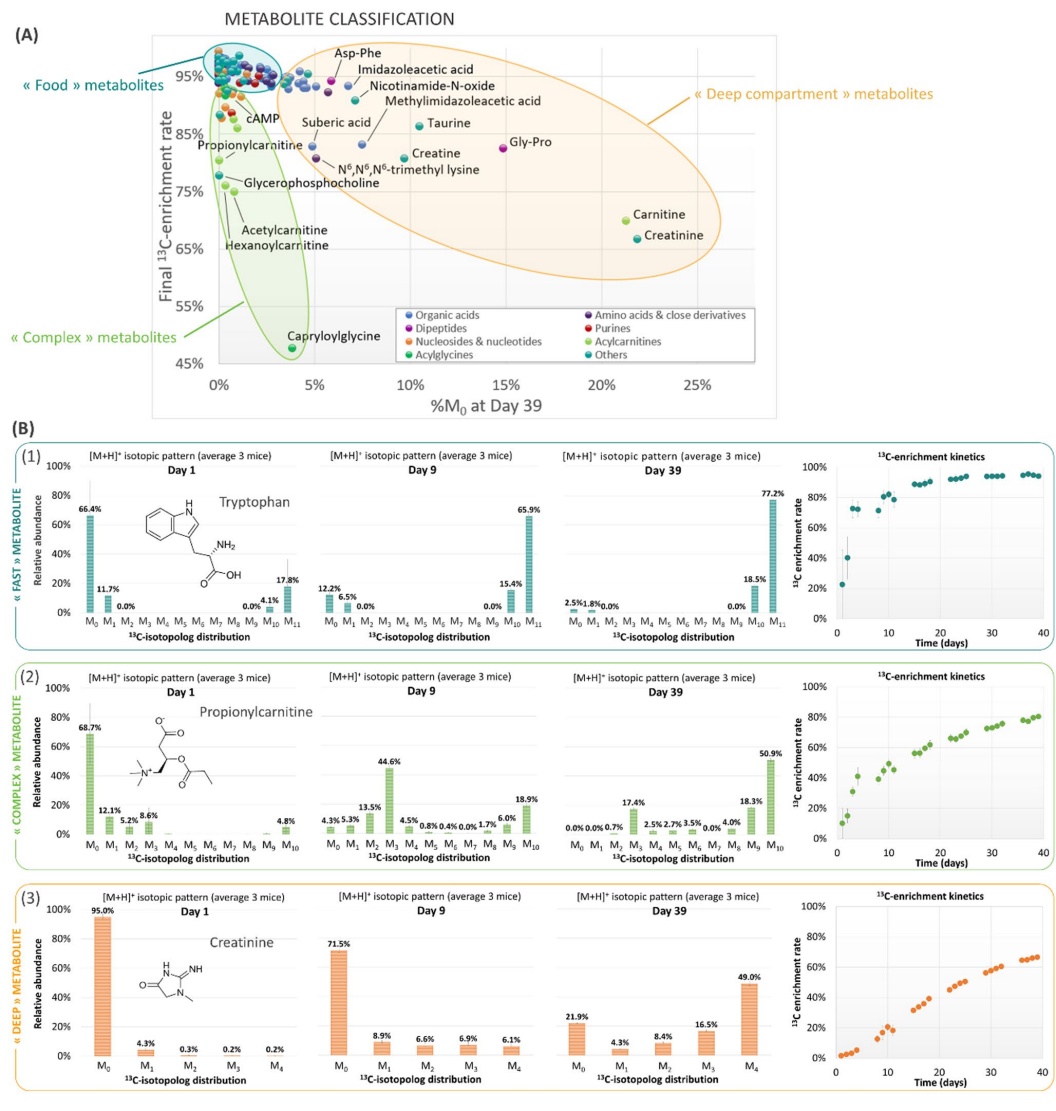
Kinetic trends of ^13^C-labeling for the 128 unambiguously identified metabolites. **A** Classification of the metabolites in three “metabolic” groups evidenced by ploting the final ^13^C-enrichement rate against the final amount of remaining unlabeled isotopolog (M_0_) in the isotopic distribution of each metabolite; **B** Illustration of three typical ^13^C-enrichment profiles with tryptophan, propionylcarnitine and creatinine as representative examples. Isotopic pattern reconstructions are average data for the three mice

As a summary, the ^13^C-enrichment results are also presented as Circos Plots in Fig. 5A for the 128 “level 1” metabolites at three different days (early, medium and late) and in Fig. 5B for the 110 acyl-derivatives. Additional Circos plots are available in the Supplementary file2, FigS6-S13 for all 238 identified metabolites. Review of the enrichment data led to the first relevant observation that inter-animal variability is exceptionally low, especially at day 39 with a standard deviation (SD) below 5% in the final ^13^C-enrichment rates (Supplementary file3, Table S6 and S7). Secondly, when comparing the data at early and late times, almost all studied metabolites showed a rapid ^13^C-incorporation with 109 out of the 128 compounds identified at level 1 presenting a final ^13^C-enrichment > 90%, while only six metabolites (creatinine **10**, carnitine **56**, glycerophosphorylcholine **75**, acetylcarnitine **78**, capryloylglycine **111** and hexanoylcarnitine **120**) did not reach 80% of ^13^C-content at the last time point. Note that, 124 metabolites out of 128 were more than 50% labeled at day 15, demonstrating that, for this set of molecules, the average t_1/2_ was below 2 weeks. Among the short- and medium- chain fatty acid conjugates, 64 compounds did not reach a 90% ^13^C-enrichment rate at the final time point (Fig. 5B and Supplementary file2, FigS10, S11, S12 and S13). Most of them belong to the acylcarnitine family. This lack of ^13^C-incorporation is probably linked to the slow labeling of the carnitine (**56**) itself, which is only ~ 70% enriched by day 39. Moreover, capryloylglycine (**111**) and five other metabolites (**141**, **165**, **167**, **186** and **191**) show less than 50% ^13^C-enrichment at day 39. These metabolites share a C_8_-acyl chain, saturated (C_7_H_15_-CO-) for five of them (**111**, **141**, **165**, **167** and **191**) and mono-unsaturated (C_7_H_13_-CO-) for the sixth (**186**). This low rate of ^13^C-incorporation is due to the absence of labeling of the acyl side chain throughout the six weeks (except for capryloylglycine whose acyl moiety is partly labeled), while the polar head progressively incorporates carbone-13. This observation shows that some of the fatty acids released in mouse urine in the form of glycine, carnitine or glucuronide conjugates originate essentially from storage compartments such as adipose tissues. These fatty acids are neither supplied by the diet nor metabolically produced by the mice on this 6-week diet. However, not all octanoic and octenoic acid isomers (i.e., C_7_H_15_-CO_2_H or C_7_H_13_-CO_2_H) are concerned, since other conjugates with C_8_-acyl chains show ^13^C-incorporation (e.g., **124**, **139**, **140**, **142**, **166**, **168**, **173**, **174**, **183**, **184**, **187**–**190**).

## Discussion

We have seen that, although the overall enrichment of metabolites is rapid and most often reaches a final level of over 90%, some compounds appear to stand out and exhibit slower ^13^C-enrichment kinetics and a more limited final ^13^C-content. In an attempt to identify groups of metabolites exhibiting similar behavior, and to characterize in greater detail the reasons for these slower enrichment rates, we have placed the metabolites on a graph showing their final ^13^C-enrichment as a function of their percentage of residual unlabeled isotopolog M_0_ (Fig. 6A).

**Fig. 7:**
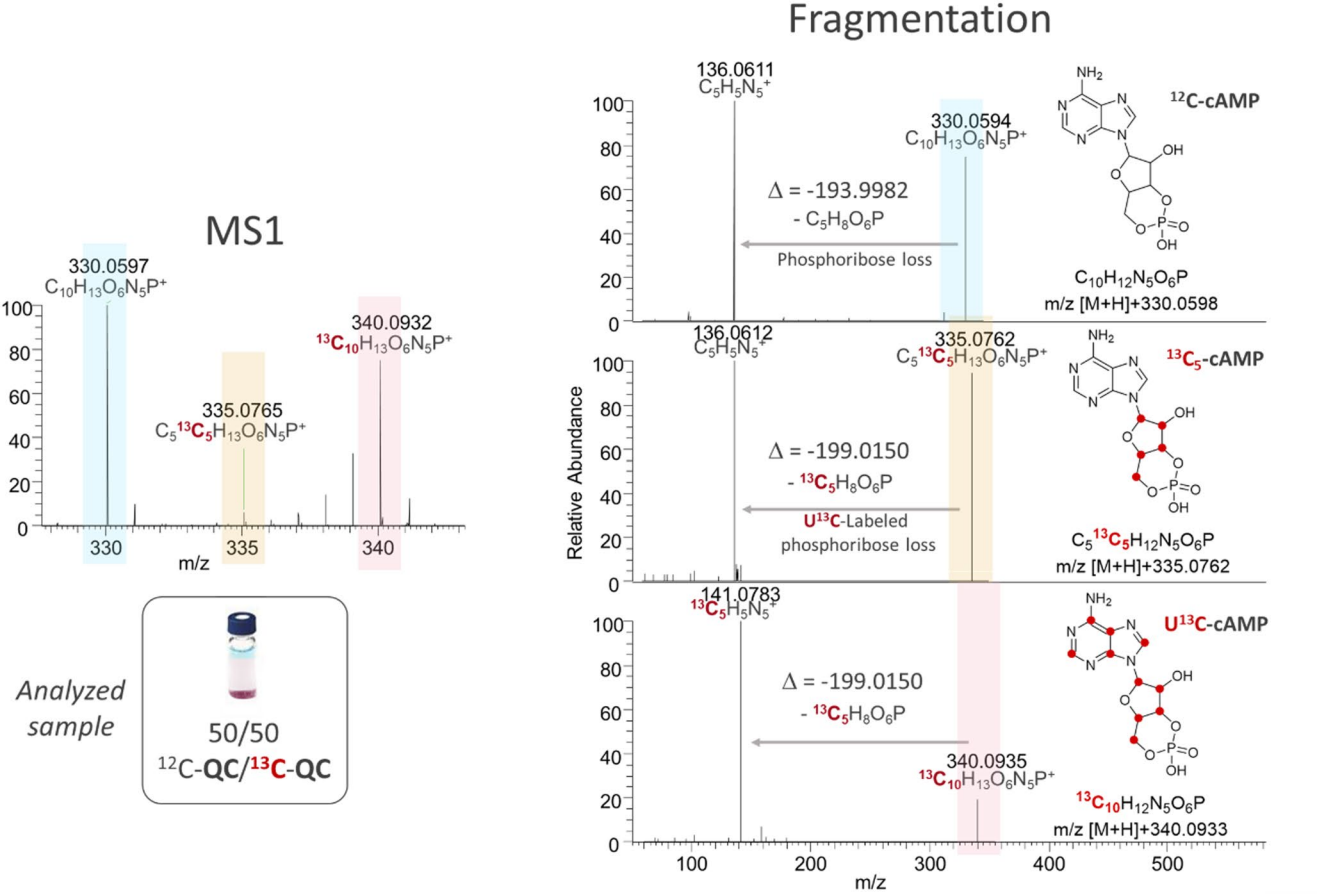
Mass spectrometry data and ^13^C-tracing of cAMP in the mix ^12^C/^13^C-QC sample. (A) Mass spectra (zoomed in between *m/z* 328 and *m/z* 343) and (B) fragmentation spectra of three isotopic versions (fully-unlabeled ^12^C-cAMP at *m/z* 330.0594, partially-labeled ^13^C_5_-cAMP at *m/z* 335.0762 and uniformly-labeled U^13^C-cAMP at *m/z* 340.0935) of the nucleotide cAMP

In this representation, we were able to identify three groups of compounds released in the urine of mice: (I) A first group (blue circle, Fig. 6A) includes molecules that display rapid and almost complete ^13^C-labeling after six weeks (i.e., ^13^C-content > 95%). They are grouped together at the top left of the chart. This group contains mainly amino and organic acids, small peptides but also nucleic acid metabolites, which are directly associated to fast central metabolism through the tricarboxylic acid cycle or glucose degradation and are referred to as “central metabolites” (Hui et al., 2017, 2020; Ragavan et al., 2021). Tryptophan (Fig. 6B(1) is an illustrative example of this class of molecules with rapid labeling. Although these metabolites quickly reach almost complete labeling, the isotopic profiles obtained at each stage of the labeling process (Supplementary file4) make it possible to highlight the stages of biosynthesis for some of these compounds, as illustrated above with isovaleroylglycine (Fig. 4). (II) The second group (green circle, Fig. 6A) includes partially labeled metabolites having variable final ^13^C-enrichment level (ranging from ~ 50 to ~ 90%) but characterized by no significant amount of remaining unlabeled isotopolog (M_0_ < 4%). They are gathered on the left-hand side of Fig. 6A and are referred to as “complex metabolites” or “multi-blocks metabolites”. Indeed, for these compounds, the ^13^C-enrichment defect is not due to the presence of remaining M_0_, but to a slow labeling step of one of the biosynthesis intermediates. Interestingly, most of the metabolites present in this group contain small or medium size fatty acid moieties, which are not excreted as such in the urine but can be tracked via their polar acyl-conjugates capable of filtering through the kidneys. Propionylcarnitine is one representative example of this class of molecules. As shown on Fig. 6B(2), its isotopic pattern at day 9 highlights the formation of a partially labeled version (M_3_) that corresponds to [^13^C_3_]-propionylcarnitine where the propionyl moiety is fully labeled while the carnitine part remains unlabeled. Conversely, some glucuronide, glycine and carnitine conjugates have a final ^13^C-enrichment limited by the fatty chain as already discussed for some octanoyl and octenoyl derivatives (Supplementary file3, Table S7). Such partial labelings highlight the different levels of ^13^C-incorporation, characteristic of multi-step biosynthesis processes, and reflect the slow turnover of fatty acids in adipose storage compartments, which can reach several months in mammals (P. Arner et al. 2019; Peter Arner et al. 2011; Palacios-Marin et al. 2023; Spalding et al. 2017). These metabolites are composed of at least two subunits or “building blocks” that embody ^13^C at significantly different rates in vivo. The 3’,5’-cyclic adenosine monophosphate (cAMP) nucleotide also belongs to this group even if it reaches a high final ^13^C-enrichment rate, close to 92%. In Fig. 7, the mass spectra of cAMP (*m/z* 330.0597, M_0_) in the ^12^C/^13^C-QC sample shows the formation of an intermediate isotopic version (partially labeled cAMP) at *m/z* 335.0765 (M_5_) before reaching a full incorporation of carbon-13 at *m/z* 340.0932 (M_10_). Comparison of the fragmentation spectra of M_0_, M_5_ and M_10_ shows that the M_5_ intermediate is composed of a uniformly ^13^C-labeled (U^13^C-) phosphoribosyl moiety and an unlabeled nucleobase detected at *m/z* 136.0611 (protonated ^12^C_5_-adenine) in both fragmentation spectra of M_0_ and M_5_ ions. This demonstrates a much faster in vivo ^13^C-labeling of the ribose subunit compared to the adenine moiety that results from a more complex biosynthesis process involving various amino acids, including glycine.

(III) In the third group (orange circle, Fig. 6A), the metabolites have the particularity of presenting a non-negligible and persistent proportion of unlabeled species (4% < M_0_ < 22%) in their isotopic pattern at the final time, responsible for an incomplete global ^13^C-enrichment ranging from 66 to 96%. We define this group as “deep compartment metabolites” because most of them probably originate from storage compartments such as adipose tissues or from the slow degradation of endogenous proteins. Carnitine and creatinine (Fig. 6B(3) are extreme examples of this category with 21 and 22% of remaining M_0_ for 69.9 and 66.6% of ^13^C-incorporation at day 39, respectively (Supplementary file3, Table S6). Some other metabolites belonging to this last category are linked to specific proteins with very slow catabolism. For instance, the dipeptide Gly-Pro which is a repeated sequence of collagen, an exceptionally long-lived protein in cartilage (Kobak et al., 2022), is found at 14.9% unlabeled in urine after 39 days. Similar observation is made with *N*^*6*^,*N*^*6*^,*N*^*6*^-trimethyl-lysine (TML, M_0_ of 5.1% at day 39) which is a conserved motif of histones whose turnover in the liver and brain of mice is 4 to 12 months (Commerford et al., 1982). Similarly, carnitine, a metabolite of TML, remains unlabeled at 21.3% on day 39, reflecting its specific biological role. Indeed, carnitine acts as a transporter of fatty acids across the mitochondrial membrane and is both derived from the diet and de novo biosynthesized from lysine and methionine to maintain its homeostasis. It is distributed into two kinetically defined compartments: one relatively small with rapid turnover (i.e., liver, kidney, and other tissues) and the other large with slow turnover (e.g., muscles), with a half-life greater than 40 days, resulting in a continuous release of ^12^C-species in the urine (Rebouche, 2004; Tsai et al., 1975). In addition to compounds derived directly from protein metabolism, we have also confirmed the persistence of unlabeled metabolites previously associated with slow endogenous turnover; creatine and its metabolite creatinine fall into this category of metabolites (Supplementary file3, Table S6). The typical creatine content of skeletal muscle is 1% of dry muscle mass, and only 1–2% of intramuscular creatine is degraded per day, which is reflected by the presence of a significant amount of full ^12^C-isotopolog M_0_ (5.7 and 21.9% for creatine and creatinine, respectively). Taurine with an elevated level of unlabeled form (10.5%) at day 39 is another example. This metabolite is not incorporated into proteins, but is naturally derived from cysteine or produced by a trans-sulfuration pathway converting homocysteine into hypotaurine. Taurine regulates cell volume and is necessary for normal skeletal muscle functioning. Its slow elimination from intracellular pools has been demonstrated in rodents due to low mobility in the cytosol, resulting in a body half-life of 15 days (Huxtable, 1981; Macaione et al., 1975; Sved et al., 2007).

Interestingly, we also found that a great number of other metabolites remains unlabeled (5 to 8% of the total content), and among them numerous low molecular weight acidic metabolites (Asp-Phe, oxovaleric acid, pipecolinic acid, imidazole acetic acid, nicotinamide-N-oxide and methylimidazoleacetic acid). At this stage, we have no clear explanation for this, as it cannot be explained by a known storage in deep compartments, nor by the slowness of the corresponding biosynthetic pathways.

In fine, although overall urine labeling was rapid and quickly reached a high level of ^13^C-content (Supplementary file2, FigS2), isotope monitoring by LC-HRMS(/MS) of each identified molecular species throughout the 39 days of ^13^C-diet (Group 2) demonstrated that ^13^C-enrichment kinetics are considerably variable between metabolites (Supplementary file3, Table S6 and S7) and reflects their biological function, origin and biosynthesis rhythm in vivo.

## Conclusion

This proof-of-concept study explores broad metabolome dynamics in a mammalian species under a highly ¹³C-enriched diet. It demonstrates that mice can safely tolerate > 97% ¹³C-enriched diets without compromising growth or welfare. Establishing this tolerance is essential for consideration in in vivo metabolic flux analyses and comprehensive stable-isotope labeling studies in mammals. A large set of metabolite-derived signals (> 3,700 features) detected as  ^12^C/^13^C-ion pairs has been evidenced in mouse urine under the current LC-HRMS conditions. Among these features, 128 metabolites were unambiguously identified, including amino and organic acids, small peptides, nucleosides, and 110 more lipophilic species belonging to acyl-carnitine, acyl-glycine and acyl-glucuronide classes, were identified as “level 2” molecules. All of them provided broad coverage and fulfilled our goal of highlighting metabolites at the crossroads of multiple pathways with potential pathway-specific ¹³C-incorporation kinetics. By deciphering their labeling kinetics over a 39-days period, it was shown that despite the overall rapid turnover of metabolites through urinary excretion, a diversity of dynamic profiles could be observed, reflecting the complex interplay between biosynthesis and the slow release of metabolites from deep compartments.

We are expecting further applications of this work. The collected spectral data (LC-MS and LC-MS/MS, Supplementary file3, Tables S4 and Table S5) will constitute a relevant database for further annotation of ^13^C-labeled materials, as well as for the resolution of unknown human metabolites due to the high metabolome homology between the two species. Together with the recent work of Gao et al. (Gao et al., 2025) based on isotopolog similarity networks, our data could provide new insights into metabolic pathways in mice and potentially in humans. As organs and blood were also collected at the end of the study, analyses of tissue labeling are planned to complement urine observations, using also complementary chromatographic platforms, such as HILIC, to further expand the current metabolome coverage. Finally, this approach using fully labeled ^13^C-mice could be extended to monitor metabolite dynamics following nutritional or pharmacological interventions, as well as during disease progression.

## Supplementary Information

Below is the link to the electronic supplementary material.

Supplementary Material 1

Supplementary Material 2

Supplementary Material 3

Supplementary Material 4

## Data Availability

All processed data are available in the article, in the Supplementary file3 (Tables S2, S3, S4, S5, S6 and S7) and Supplementary file4.
