# Supplementary material for: Urinary metabolome dynamics in 13C-labeled mice
Source: Metabolomics. 2025 Dec 29;22(1):15. doi: 10.1007/s11306-025-02391-4 (PMC12748121; doi:10.1007/s11306-025-02391-4)
Supplement: Supplementary file 1 — Supplementary Material 1 [file 11306_2025_2391_MOESM1_ESM.docx]

Supplementary file 1: Supporting Methods

**Content**

Materials and Methods – Experimental details

1. *In vivo* ^13^C-labeling and sample collection
   1. ^13^C-material
   2. ^13^C-labeled wheat production, collection and storage
   3. ^13^C-labeled mice
2. Profiling of mouse urinary ^12^C- and ^13^C-metabolomes
   1. Chemicals
   2. Urine sample preparation for LC-HRMS analysis
   3. Analytical sequences
   4. Chromatographic separation conditions
   5. High-resolution mass spectrometry (HRMS) instrumentation and settings
   6. Data processing
3. Metabolomic landscape of mouse urine
   1. Level 1 identification
   2. Acyl-carnitines, acyl-glucuronides and acyl-glycines level 2 identification

*1. In vivo* ^13^C-labeling

- 1. **^13^C-material**

[^13^C]CO_2_ as well as spirulina and ^13^C-spirulina were purchased from Eurisotop (France).

- 1. **^13^C-labeled wheat production, collection and storage**

Durum wheat (cv. Caphorn) was sown and grown in an air-tight chamber (Phytotec platform) which allows accurate regulation of atmospheric gas composition and environmental parameters.(André et al. 1985; Matulova et al. 2005) Photon flux rate 400 µmol/m²/s. Gradual photoperiod (8-14h) during 90 days, and then, 14h photoperiod. Gradual daytime temperature (6 to 21°C) during 90 days, and then, 21°C constant temperature. Gradual night temperature (6 to 18°C) during 90 days, and then, 18°C constant temperature. Relative humidity 70%. Watering with half diluted Hoagland’s nutrient solution. [^13^C]CO_2_ concentration was maintained at 420 µL/L. Regulation was achieved by automatic injection of pure (99% atom13C) starting from the beginning of the culture. The partial pressure in the chamber was continuously monitored by Near Infrared Spectroscopy. Wheat culture duration: 188 days. ^13^C labeling was measured to 97.2% in the 370 g of grain produced.

- 1. **^13^C-labeled mice**

Ethical approval: This project was submitted to and received a favorable opinion from the Ethics Committee n°044. It has been authorized by the French Ministry of Research and Higher Education under reference APAFIS#8926. Work involving the use of animals was carried out in the laboratory approved under reference C300816.

Three 5-week-old male bALB/cJ mice were fed a diet consisting of 360 g ^13^C-wheat per 100 g ^13^C-spirulina. For four days a week, the animals were kept in metabolism cages, and urine was collected and stored at -80°C. For three days a week (including holidays and weekends), the animals were kept in standard cages (1/cage), still on a ^13^C-labeled diet. This diet was followed for 39 days, after which the animals were sacrificed and all organs were collected and stored at -80°C.

2. Profiling of mouse urinary ^12^C- and ^13^C-metabolomes

2.1 Chemicals

Metabolite standards were purchased from Sigma-Aldrich (Saint Quentin Fallavier, France). All extraction and HPLC solvents (HPLC grade water and acetonitrile) as well as formic acid (≥ 98.0 % purity) as mobile phase additive, were purchased from Sigma-Aldrich.

**2.2 Urine sample preparation for LC-HRMS analysis**

*Sample preparation for identification and kinetics data*

Urine samples were stored at -80°C immediately after collection, then thawed and aliquoted in batches of 100 microliters. 5 µL of each sample was also taken before re-freezing the aliquots to prepare the LC-HRMS injection vials. Sample preparation was as follows: each 5 µL urine sample was diluted in 45 µL of a 95/5 H_2_O/CH_3_CN mixture containing 0.1% formic acid (sample recovery solution, S_A_). The resulting samples were centrifuged at 20,000 g for 15 min at 4°C. The supernatants were transferred into vials for injection and LC-HRMS analyses.

Quality control samples (QC) for the control group were obtained following these successive dilutions: 10 µL of each remaining control urine supernatants were pooled (QC dil 1). 100µL of QC dil 1 was diluted in 100µL of S_A_ (QC dil 2). 100µL of QC dil 2 was diluted in 100µL of S_A_ (QC dil 4). 100µL of QC dil 4 was diluted in 100µL of S_A_ (QC dil 8). QC for the ^13^C-labeled group were obtained using the same process with ^13^C-labeled urine supernatants.

*Sample preparation for identification and DDA experiments*

For each group, 5 µL of the urines collected on the first day, Day 11, Day 25, and the final day of the experiment for the three mice were pooled and diluted in 180 µL of S_A_. Moreover, 5 µL of the urines collected on the final day of the experiments (3 urines for the control group and 3 urines for the ^13^C-labeled group) were pooled. 10 µL of this ^12^C-QC/^13^C-QC was then diluted in 90 µL of S_A_. The resulting samples (^12^C-QC, ^13^C-QC, and ^12^C-QC/^13^C-QC) were centrifuged at 20,000 g for 15 min at 4°C. The supernatants were transferred into vials for injection and LC-HRMS/MS analyses.

**2.3 Analytical sequences**

Daily and QC samples

Samples were analyzed using a common workflow in metabolomics consisting of successive diluted QC samples followed by randomized biological samples.

^12^C-QC, ^13^C-QC, ^12^C-QC/^13^C-QC

Samples were analyzed in triplicates.

**2.4 Chromatographic separation conditions**

Chromatographic separation was carried out on a Dionex Ultimate 3000 system (Thermo Fisher Scientific) equipped with a C18 Hypersil Gold column 2.1 x 150 mm, 1.9 µm, 175 Å (Thermo Fisher Scientific) kept at 30°C applying elution conditions as follows: mobile phase A: 0.1 % formic acid in water; mobile phase B: 0.1 % formic acid in acetonitrile. The following gradient was applied at a flow rate of 500 µL/min: t = 0 min, 5% B; t = 2 min, 5% B; t = 13 min, 100% B; t = 25.5 min, 100% B; t = 25.51 min, 5% B; t = 30 min, 5% B.

**2.5 High-resolution mass spectrometry (HRMS) instrumentation and settings**

HRMS/MS analyses were performed on an Orbitrap Tribrid Fusion (Thermo Fisher Scientific, Courtaboeuf, France) equipped with a heated electrospray ionization source operated in the positive or negative mode. Source parameters were set as follows:

Ion transfer parameters were set as follows: mass range at “normal”, lens RF level at 60 %.

For LC-HRMS-only experiments

Scan parameters applied were as follows: acquisition time from 0 to 20 min, positive or negative ionization mode, micro scan at “1”, data type at “profile”, AGC target at 5e4 (12.5 %). The data were acquired using the Orbitrap analyzer, operated with a resolution of 240k (FWHM) at *m/z* 200; maximum injection time was set at 400 ms, and the scan range (*m/z*) from *m/z* 85 to 1000.

For LC-HRMS/MS experiments with a DDA - Top 10 method

*
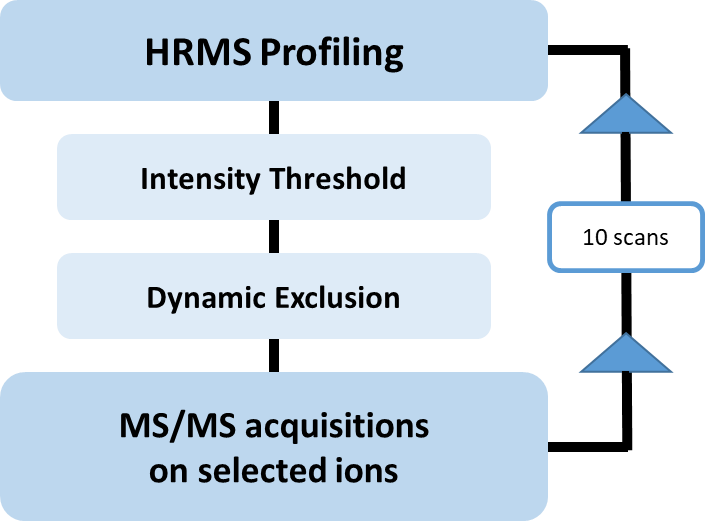
*

*HRMS acquisitions*

Scan parameters applied were as follows: acquisition time from 0 to 20 min, positive or negative ionization mode, micro scan at “1”, data type at “profile”, AGC target at 5e4 (12.5 %). The data were acquired using the Orbitrap analyzer, operated with a resolution of 120k (FWHM) at *m/z* 200; maximum injection time was set at 200 ms, and the scan range (*m/z*) from *m/z* 75 to 1000.

*HRMS/MS acquisitions*

A dynamic exclusion of filter was applied with parameters set as follows: Exclude during 5 s if seen 3 times within 4s, mass tolerance at ± 5 ppm, and exclude isotope at “false”. An intensity threshold filter was also implemented at 5.0E+04. The MS/MS activation parameters were set as follows: Isolation in the Quadrupole with an isolation window (m/z) at 0.8, HCD activation type with a stepped normalized collision energy at 30±20 %. The data were acquired using the Orbitrap analyzer, operated with a resolution of 15k (FWHM) at m/z 200; maximum injection time was set on “Auto” mode, AGC Target on “Standard”, micro scan at “1” and a “profile” data type was used.

**2.6. Data processing**

*Data conversion to open format*: for automated data pre-processing, LC-HRMS .raw data were first converted into centroid mzxml files using MSConvert (ProteoWizard). Conversion settings were 64 bit and peak picking was used as first filter. The mzxml files were then processed using either the Workflow4metabolomics (W4M) Galaxy instance or MetExtract II open-source software.

*W4M processing of the ^12^C-sample data (Series 1, Fig. 2A)*

A workflow combining the XCMS modules from the W4M platform(Giacomoni et al. 2015) was used to perform peak extraction and retention time alignment. The CAMERA module was also implemented in the workflow to annotate isotopes and adducts. The peak list was then filtered according to three criteria: (i) chromatographic peak areas ratio between biological and blank samples > 3, (ii) coefficient of variation (CV) of metabolites in the QC samples < 30%, (iii) correlation between QC dilution factors and areas of chromatographic peaks > 70%. Overall, 9016 (*m/z*; RT) features in the positive ionization mode and 11662 in the negative mode could be obtained. After filtering the data (CV QCs < 30%; Bio Area /Bk Area > 3; QCdil correlation), 5401 and 7488 signals remained that were considered as “real” chemical signals.

*MetExtract II processing of* ^12^C-QC, ^13^C-QC and ^12^C-QC/^13^C-QC data *(Series 2, Fig. 2A)*

For the set of LC-HRMS data obtained from series 2 in the workflow presented in Fig. 2A, the following parameters were applied in the AllExtract module of MetExtract II, for the processing of the mzxml converted files:

*MetExtractII data processing of daily* ^13^C-urine samples *(Fig. 2B)*

Note that to determine the kinetic of ^13^C-incorporation into metabolites, untargeted LC-HRMS-based profiling was performed on each individual ^13^C-urine samples obtained from the group of three mice fed with ^13^C-nutrients for 6 weeks (Group 2). The workflow depicted in Fig. 2B was applied to specifically follow the evolution of the isotopic enrichment of the murine metabolites over time. The same parameters as above (AllExtract module of MetExtract II) were applied but this time with the TracExtract module of MetExtract II, for the processing of the mzxml converted files.

The objective was to extract the carbon isotopic pattern of each biological signal and determine the *in vivo* ^13^C-labeling kinetics of the corresponding mice metabolites. The acquired LC-HRMS data were processed in an untargeted manner using MetExtract II software, but this time using the TracExtract module to specifically extract and cluster all isotopic versions (isotopologs) of each molecular species. As a result, an isotopic distribution for each metabolite signal and at each time point could be extracted and plotted to visualise the gradual incorporation of carbon-13.

**3. Metabolomic landscape of mouse urine**

**3.1. Level 1 identification**

The W4M peak list and the unlabeled ions of the MetExtract II – AllExtract peak list were annotated using an in-house spectral library that includes more than 1,200 metabolites. The two annotated peak lists were merged, and the annotations were curated using the carbon stoichiometry provided by MetExtract II when available. Annotations were then manually checked using the Qualbrowser module of Xcalibur (version 4.1, Thermo Fisher Scientific). If no labeled counterpart had been automatically extracted for a signal (W4M extraction only), the carbon stoichiometry suggested by the annotation was considered to calculate the expected m/z of the labeled counterpart. For each signal, the unlabeled and ^13^C-labeled forms were then inspected. For unlabeled ions: retention time (ΔRT < 0.1 min), m/z and MS/MS data were compared to those of available standards. For ^13^C-labeled ions: m/z were compared to the unlabeled counterpart (∆m/z_12C-13C_ = n*1.0033 ± 0.0003, with n the carbon stoichiometry), ions of the pairs had to co-elute, the presence of the M_n-1_ isotopolog (∆m/z_12C-13C_ = (n-1)*1.0033 ± 0.0003) and absence of M_n+1_ isotopolog (∆m/z_12C-13C_ = (n+1)*1.0033 ± 0.0003) were checked. When available, the MS/MS fragmentation profile was compared to the unlabeled one. The resulting list of 128 identified metabolites are summarized in Sup. file3, TableS4 with their corresponding LC-MS data (*m/z* unlabeled, *m/z* labeled, retention time, intensity, etc.)

**3.2. Acyl-carnitines, acyl-glucuronides and acyl-glycines level 2 identification**

An in-silico library of compounds was first designed based on the following generic chemical formulas: C_n_H_m_O_x_-CO-carnitine (carnitine = C_7_H_14_NO_3_), C_n_H_m_O_x_-CO-glycine (with glycine = C_2_H_4_NO_2_) and C_n_H_m_O_x_-CO-glucuronide (with glucuronide = C_6_H_9_O_7_), with C_n_H_m_O_x_ (n, m and x integers) being the acyl-chain attached to the polar head via the carbonyl (C=O) of an ester or amide linkage. The CnHmOx formulas of the apolar tail of the derivatives were limited to acyl-chain already encountered in acyl-carnitine analogs from human urine.(Yan et al. 2020) Based on their elemental formula, their [M+H]^+^ and [M-H]^-^ *m/z* values were calculated and used as an in-house dataset for annotating the unlabeled LC-HRMS profiles. For this, the Bank-in-house (BiH) W4M tool was used. After annotation, all matching signals were checked for availability of a fragmentation spectra in both the DDA dataset obtained from the ^12^C-mice urines (^12^C-QCs) and the ^13^C-mice urines (^13^C-QCs). Validation of the compounds as belonging to the above-mentioned chemical families was carried out according to the presence of fragment ions and losses characteristic of each family, as shown in Sup. file2, FigS5. The resulting list of 124 identified metabolites within this chemical families are summarized in Sup. file3, TableS5 with their corresponding LC-MS data (*m/z* unlabeled, *m/z* labeled, retention time, intensity, etc.).

**SI References**

André, M., Daguenet, A., Massimino, D., & Gerbaud, A. (1985). The C₂3A System, an Example of Quantitative Control of Plant Growth Associated with a Data Base. *SAE Transactions*, *94*, 467–476.

Giacomoni, F., Le Corguillé, G., Monsoor, M., Landi, M., Pericard, P., Pétéra, M., et al. (2015). Workflow4Metabolomics: a collaborative research infrastructure for computational metabolomics. *Bioinformatics*, *31*(9), 1493–1495. https://doi.org/10.1093/bioinformatics/btu813

Matulova, M., Nouaille, R., Capek, P., Péan, M., Forano, E., & Delort, A.-M. (2005). Degradation of wheat straw by Fibrobacter succinogenes S85: a liquid- and solid-state nuclear magnetic resonance study. *Applied and Environmental Microbiology*, *71*(3), 1247–1253. https://doi.org/10.1128/AEM.71.3.1247-1253.2005

Yan, X., Markey, S. P., Marupaka, R., Dong, Q., Cooper, B. T., Mirokhin, Y. A., et al. (2020). Mass Spectral Library of Acylcarnitines Derived from Human Urine. *Analytical Chemistry*, *92*(9), 6521–6528. https://doi.org/10.1021/acs.analchem.0c00129
