# Supplementary material for: Urinary metabolome dynamics in 13C-labeled mice
Source: Metabolomics. 2025 Dec 29;22(1):15. doi: 10.1007/s11306-025-02391-4 (PMC12748121; doi:10.1007/s11306-025-02391-4)
Supplement: Supplementary file 2 — Supplementary Material 2 [file 11306_2025_2391_MOESM2_ESM.docx]

Supplementary file 2: Supporting Figures

**List of Supporting Figures**

FigS1. Mouse weight curves

FigS2. Elemental analysis of isotopic carbon in all urine samples from Group 2

FigS3. Illustration of the extraction of tryptophan ^12^C-^13^C extreme ion pairs with MetExtract II in a ^13^C-QC sample - screenshot of software visualization

FigS4. Characterization and repartition of 128 “level 1”-identified metabolites

FigS5. Characteristic fragmentation patterns used to unambiguously identify 124 acyl-derivatives in mice urine including 14 metabolites already identified as level 1 metabolites

FigS6-S9. ^13^C-enrichment rates of the 128 “level 1” metabolites at day 4, day 15, day 25 and day 39 presented as a Circos plots.

FigS10-S13. ^13^C-enrichment data presented as Circos plots at day 4, day 15, day 25 and day 39 for acyl-glycine, acyl-carnitine and acyl-glucuronide series of compounds.


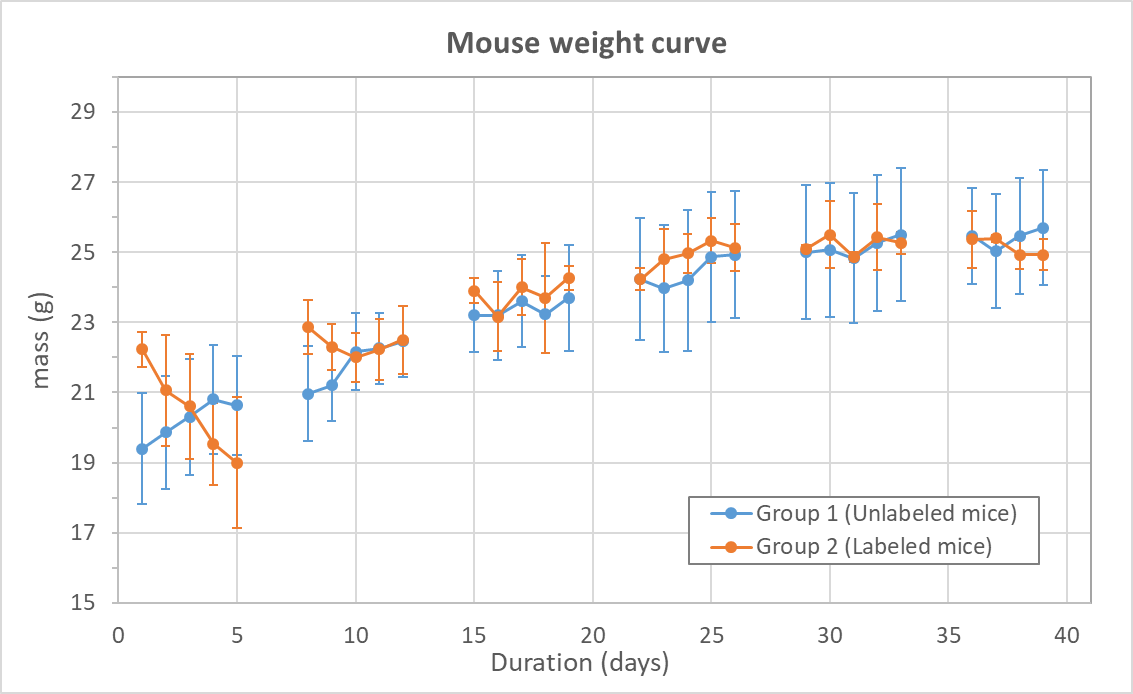


**Figure S1.** Mouse weight curves

|  | **Mouse 1** | **^13^C %** | **Mouse 2** | **^13^C %** | **Mouse 3** | **^13^C %** |
| --- | --- | --- | --- | --- | --- | --- |
|  | ***Sample*** |  | ***Sample*** |  | ***Sample*** |  |
| *Day 1* | 1-1 | **17.45%** | 2-1 | **4.59%** | 3-1 | **41.66%** |
| *Day 2* | 1-2 | **39.35%** | 2-2 | **13.38%** | 3-2 | **34.19%** |
| *Day 3* |  |  | 2-3 | **42.28%** |  |  |
| *Day 4* | 1-4 | **60.37%** | 2-4 | **61.68%** | 3-4 | **65.35%** |
| *Day 8* | 1-5 | **65.81%** | 2-5 | **78.68%** | 3-5 | **70.13%** |
| *Day 9* | 1-6 | **82.79%** | 2-6 | **79.80%** | 3-6 | **78.13%** |
| *Day 10* | 1-7 | **82.57%** | 2-7 | **85.89%** | 3-7 | **82.28%** |
| *Day 11* | 1-8 | **77.84%** | 2-8 | **83.49%** | 3-8 | **83.92%** |
| *Day 15* | 1-9 | **87.55%** | 2-9 | **87.96%** | 3-9 | **89.40%** |
| *Day 16* | 1-10 | **88.57%** | 2-10 | **86.76%** | 3-10 | **91.83%** |
| *Day 17* |  |  | 2-11 | **87.78%** |  |  |
| *Day 18* | 1-12 | **88.46%** | 2-12 | **88.30%** | 3-12 | **91.01%** |
| *Day 22* | 1-13 | **90.52%** | 2-13 | **91.79%** | 3-13 | **90.82%** |
| *Day 23* | 1-14 | **91.90%** | 2-14 | **90.76%** | 3-14 | **90.37%** |
| *Day 24* | 1-15 | **91.86%** | 2-15 | **91.36%** | 3-15 | **91.55%** |
| *Day 25* | 1-16 | **90.91%** | 2-16 | **91.24%** | 3-16 | **91.19%** |
| *Day 29* | 1-17 | **91.95%** | 2-17 | **87.72%** | 3-17 | **90.83%** |
| *Day 30* | 1-18 | **91.47%** | 2-18 | **91.06%** | 3-18 | **91.47%** |
| *Day 31* | 1-19 | **91.60%** | 2-19 | **91.38%** | 3-19 | **93.18%** |
| *Day 32* | 1-20 | **91.42%** | 2-20 | **91.19%** | 3-20 |  |
| *Day 36* | 1-21 | **92.89%** | 2-21 | **92.28%** | 3-21 |  |
| *Day 37* |  |  | 2-22 | **91.28%** |  |  |
| *Day 38* | 1-23 | **91.86%** | 2-23 | **87.44%** | 3-23 |  |
| *Day 39* | 1-24 | **91.88%** | 2-24 | **89.59%** | 3-24 | **91.37%** |

**
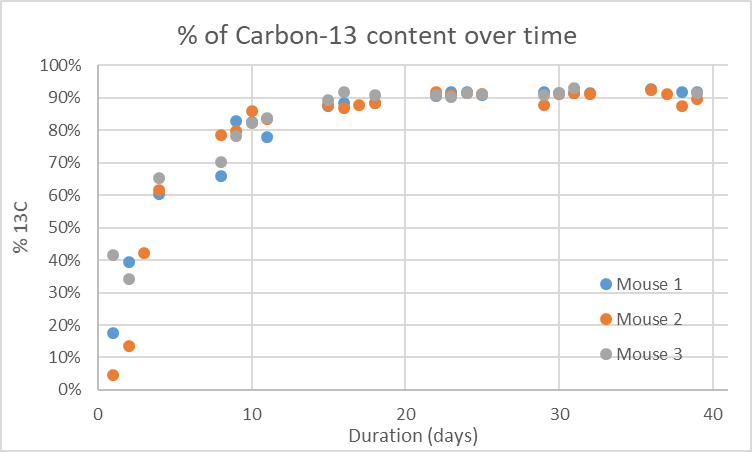
**

**Figure S2.** Elemental analysis* of isotopic carbon in all urine samples from Group 2

*Measurements were obtained from the national analysis service AQui (Atelier de Quantifications Isotopiques, INRAe, Montpellier, France, https://www1.montpellier.inra.fr/wp-inra/bpmp/plateformes-et-plateaux-techniques/ais/).

**
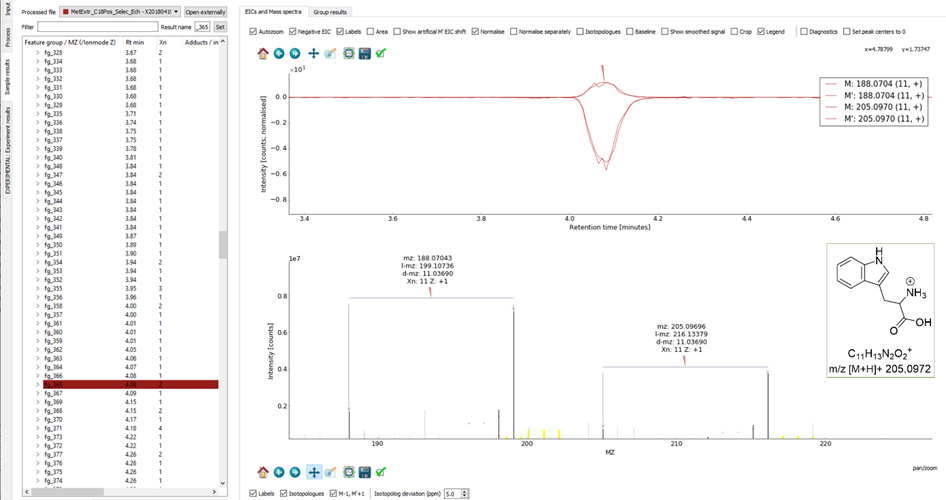
**

**Figure S3**. Illustration of the extraction of tryptophan ^12^C-^13^C extreme ion pairs with MetExtract II in a ^13^C-QC sample - screenshot of software visualization.


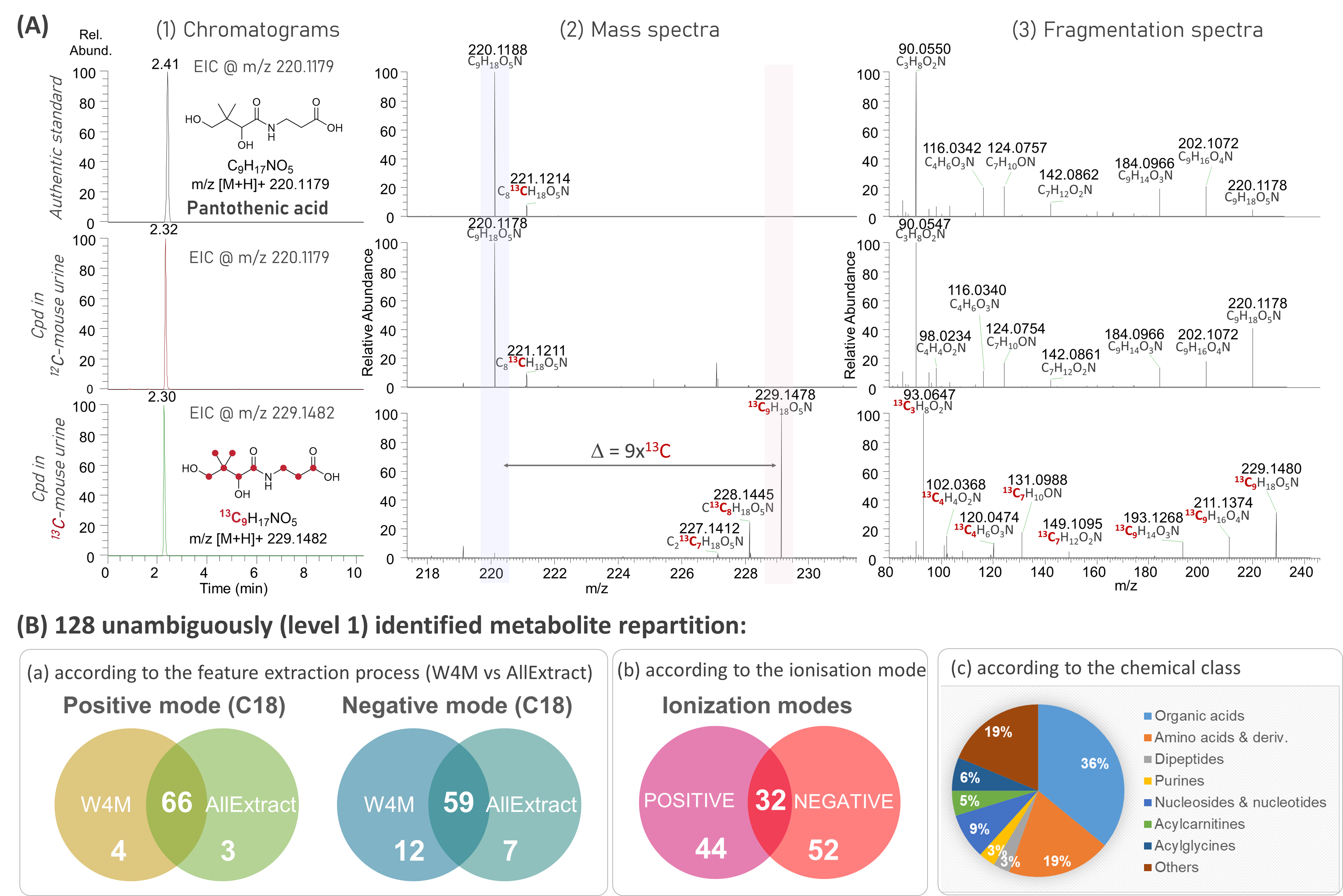


**Figure S4. Characterization and repartition of 128 “level 1”-identified metabolites** (listed in Sup. file3, Table S4) (A) Illustration of the metabolite identification and characterization process using pantothenic acid as an example in the positive ionization mode: comparison of LC-HRMS(/MS) data of an authentic standard, the unlabeled urinary metabolite and the ^13^C-fully-labeled urinary metabolite with respect to (1) C18 column coelution, (2) annotated mass spectra (MS1) and (3) annotated fragmentation spectra (MS2) concordance and coherence; (B) Number of unambiguously identified compounds according to (a) the feature extraction process (W4M vs AllExtract), (b) the ionization mode, (c) their chemical class repartition.


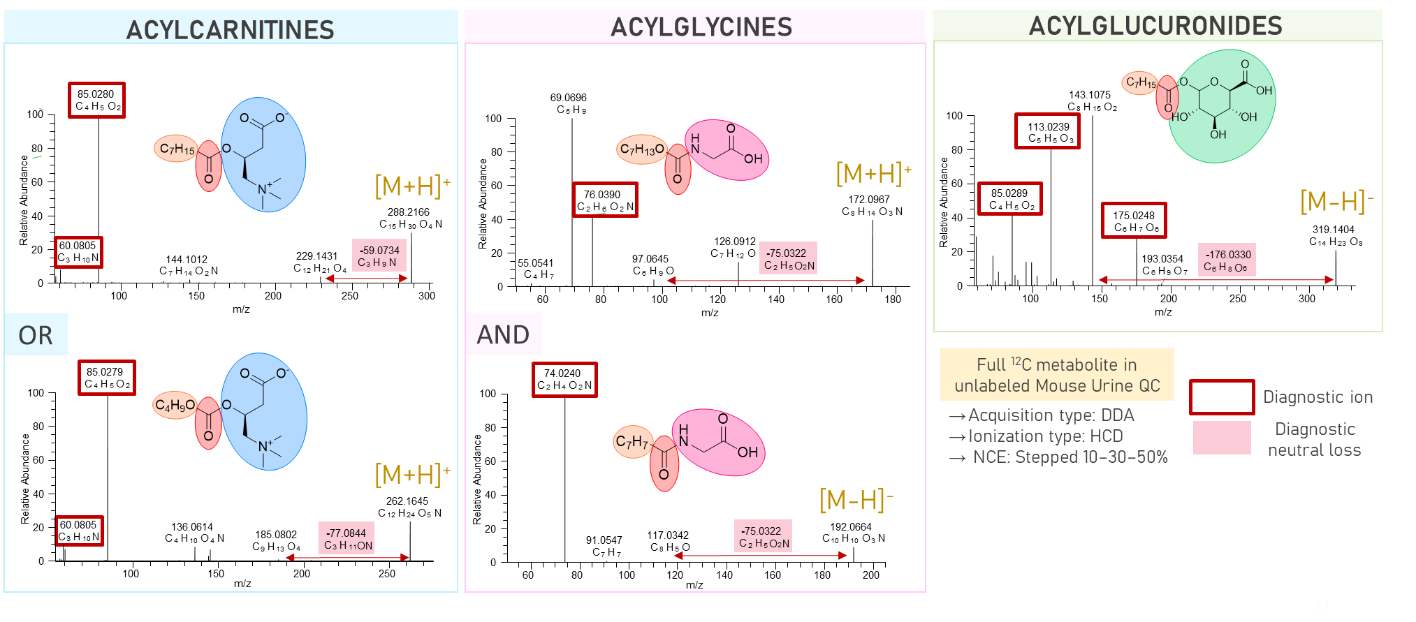


**Figure S5**. Characteristic fragmentation patterns used to unambiguously identify 124 acyl-derivatives in mice urine (Sup. file3, Table S5) including 14 metabolites already identified as level 1 metabolites.


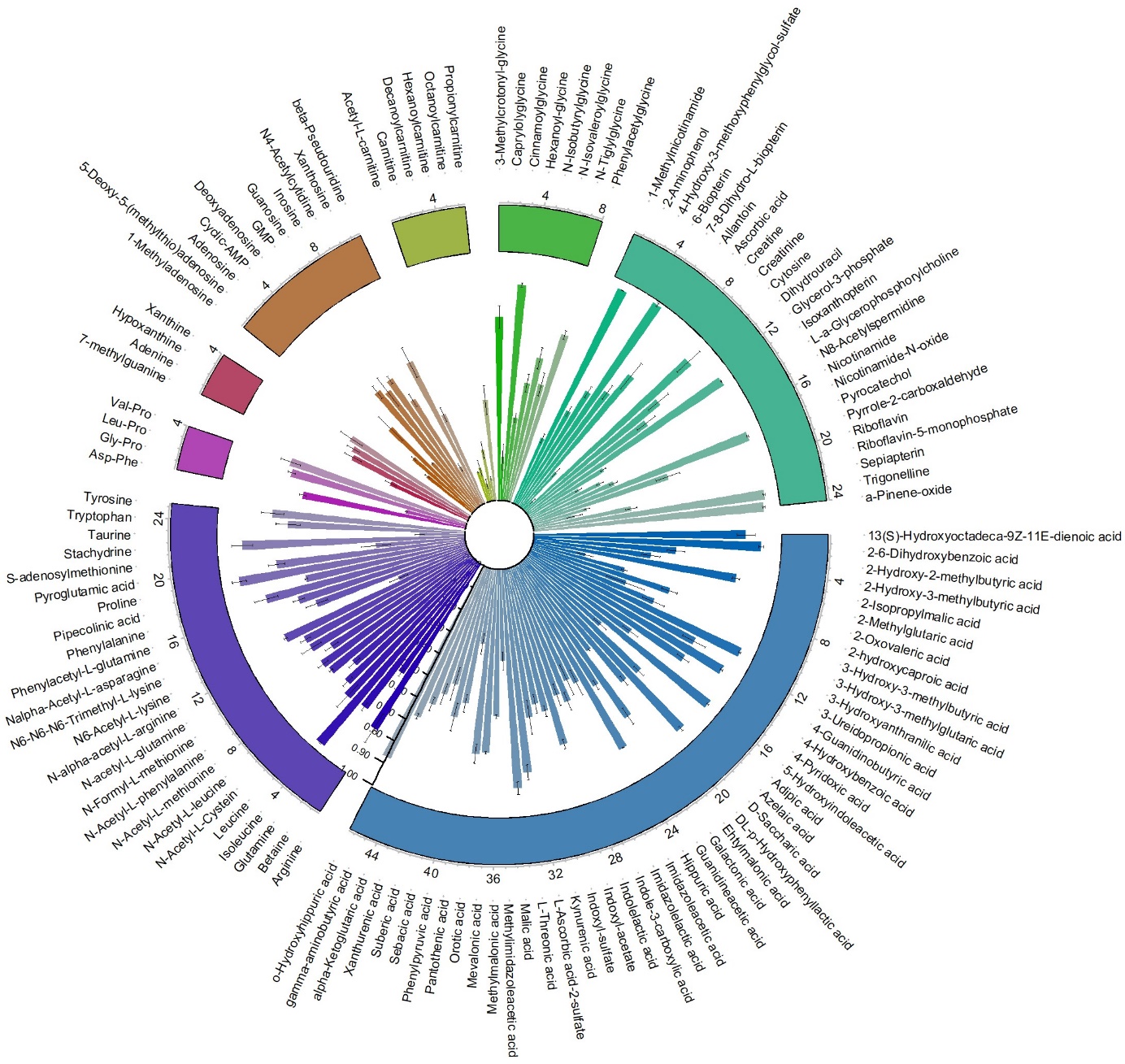
**Figure S6.** ^13^C-enrichment rates of the 128 “level 1” metabolites at day 4 presented as a Circos plot


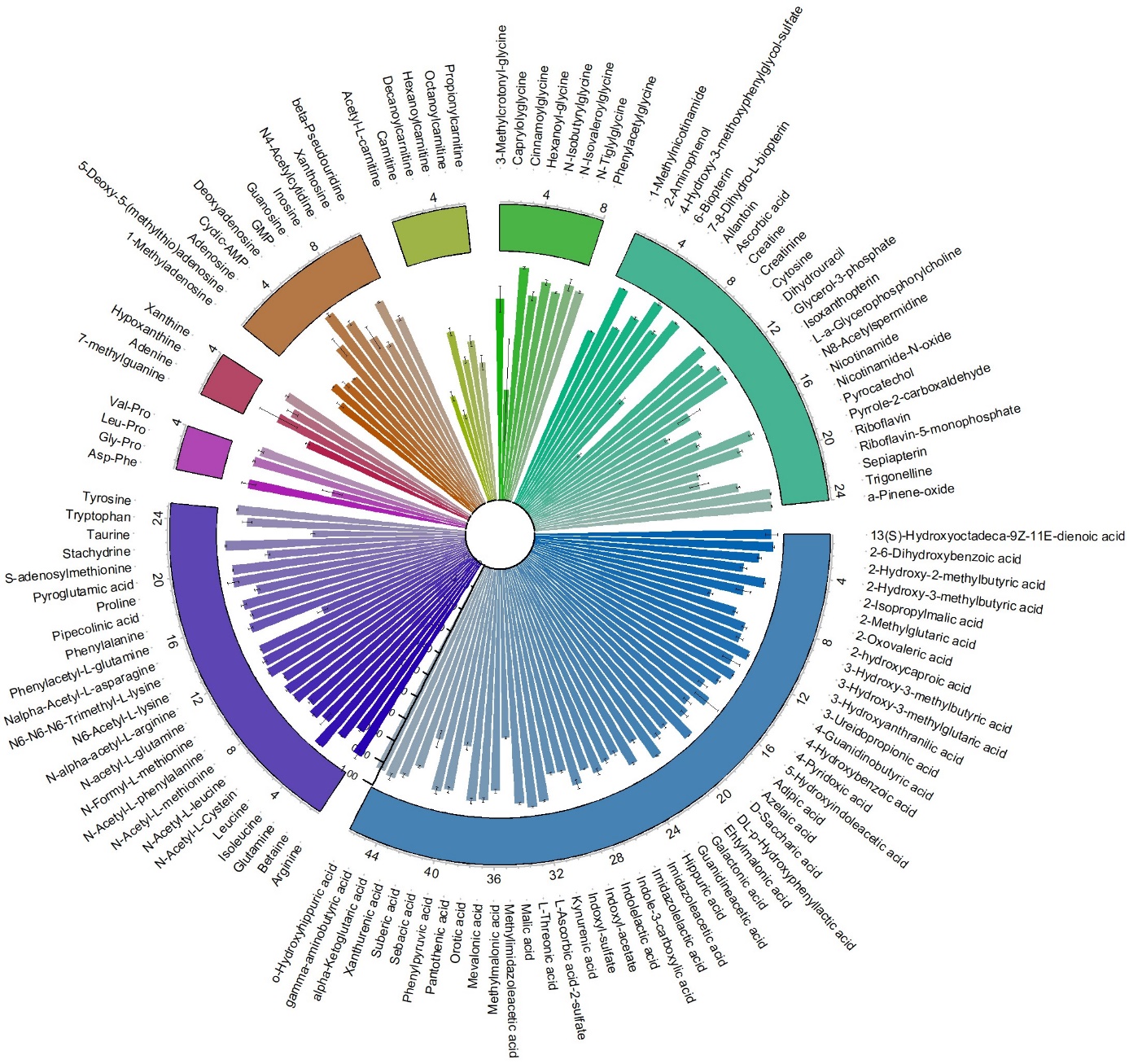
**Figure S7.** ^13^C-enrichment rates of the 128 “level 1” metabolites at day 15 presented as a Circos plot


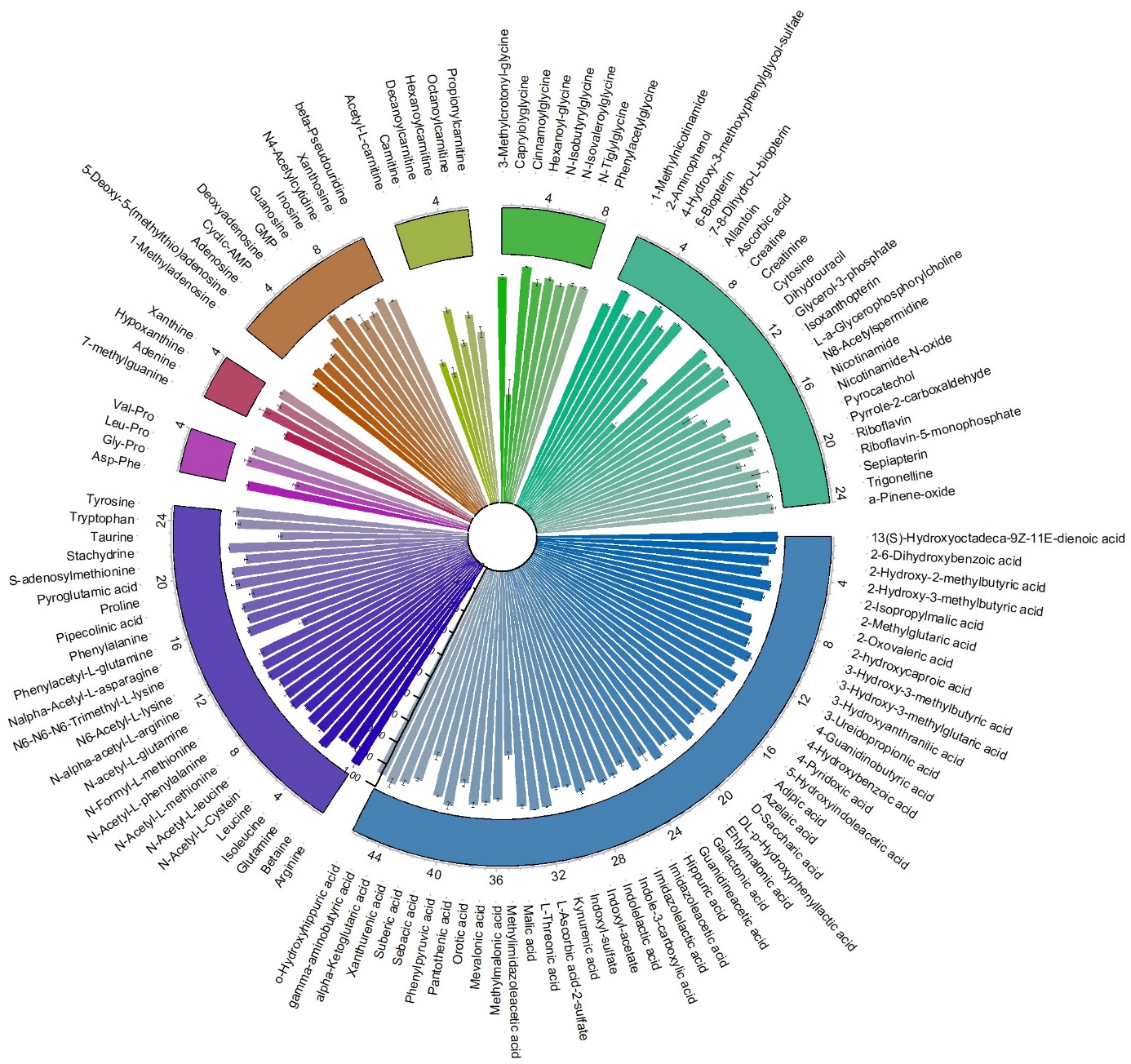
**Figure S8.** ^13^C-enrichment rates of the 128 “level 1” metabolites at day 25 presented as a Circos plot


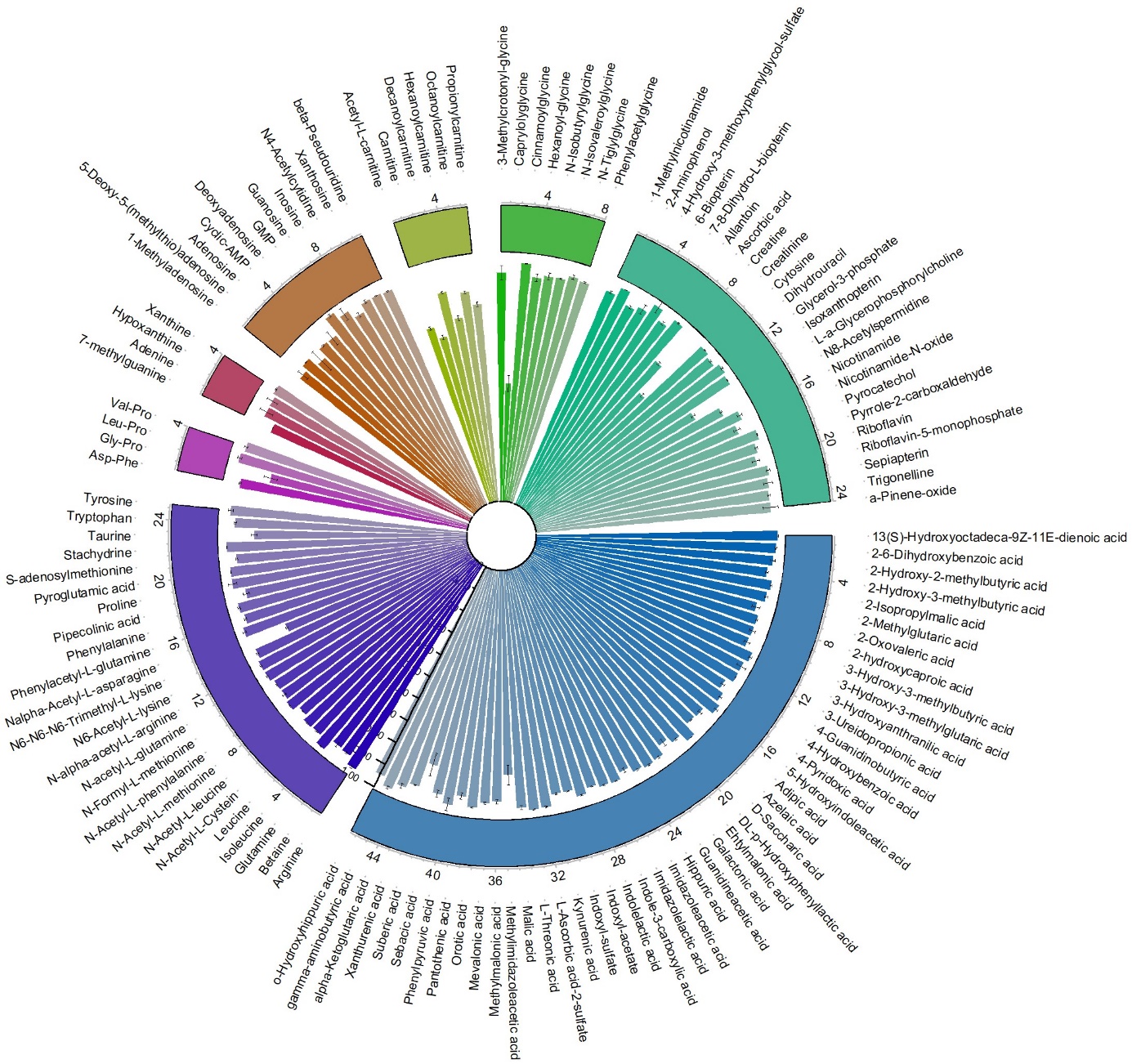
**Figure S9.** ^13^C-enrichment rates of the 128 “level 1” metabolites at day 39 presented as a Circos plot


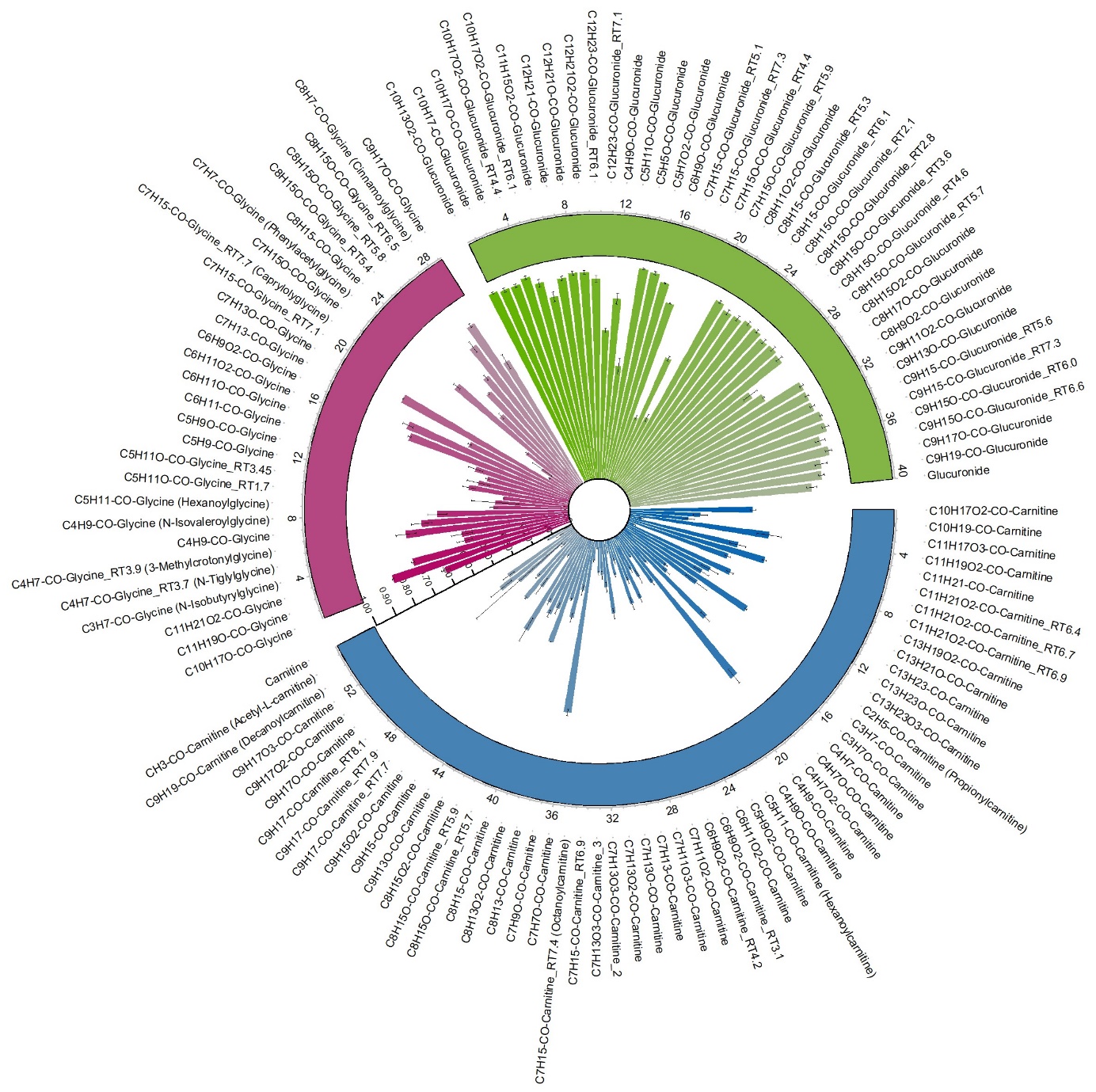
**Figure S10**. ^13^C-enrichment data presented as Circos plots at day 4 for acyl-glycine, acyl-carnitine and acyl-glucuronide series of compounds


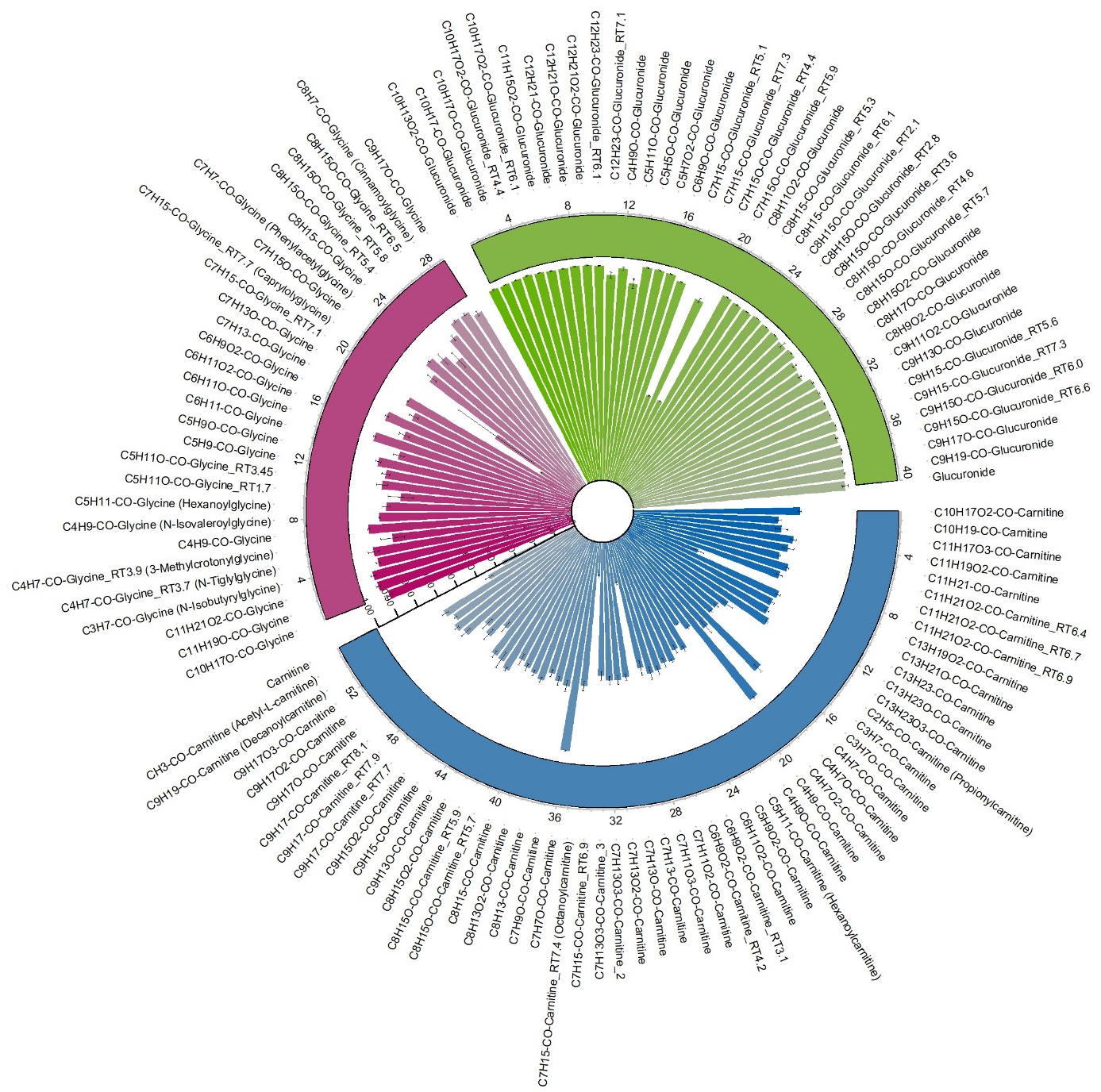
**Figure S11**. ^13^C-enrichment data presented as Circos plots at day 15 for acyl-glycine, acyl-carnitine and acyl-glucuronide series of compounds


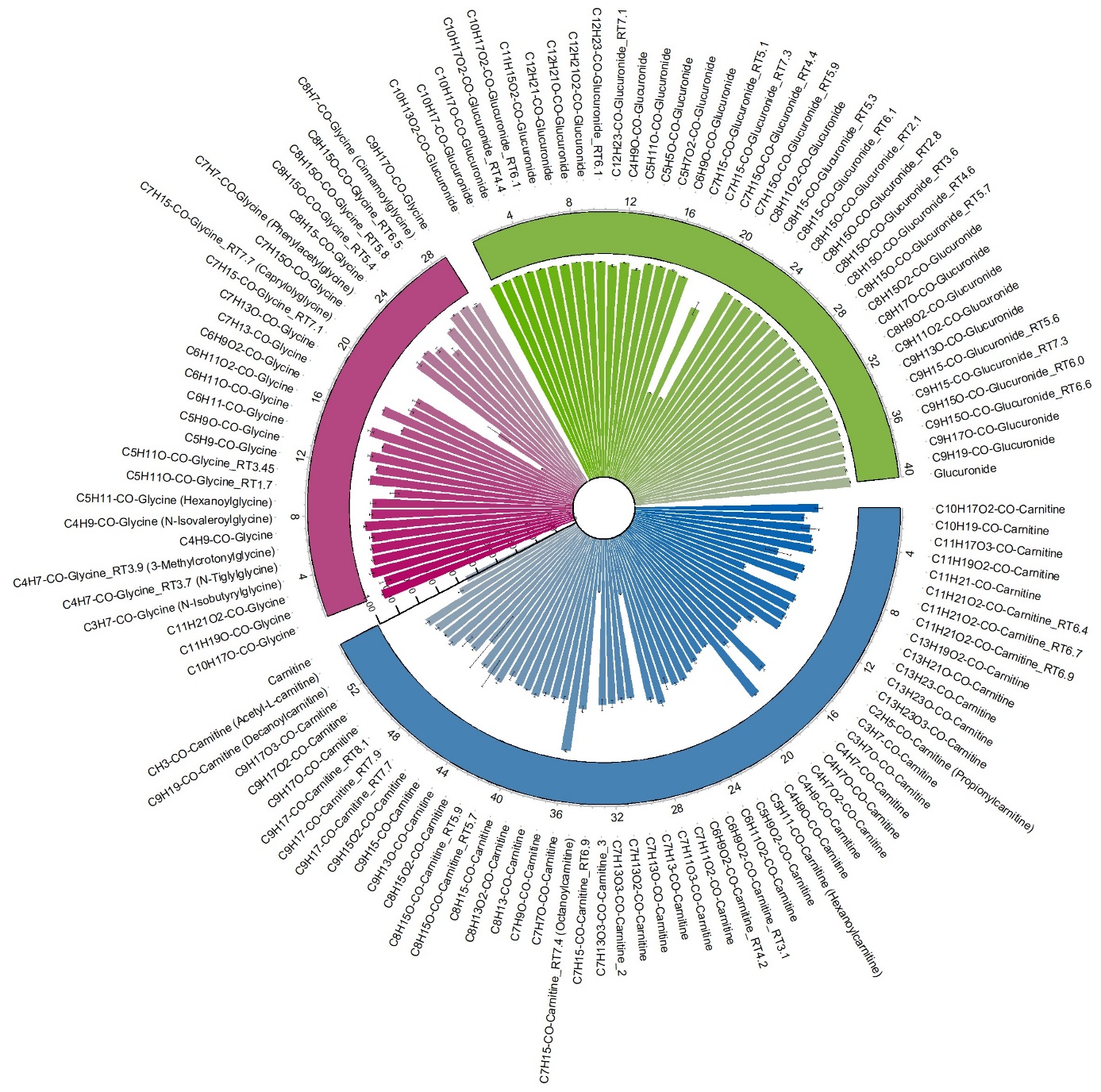
**Figure S12**. ^13^C-enrichment data presented as Circos plots at day 25 for acyl-glycine, acyl-carnitine and acyl-glucuronide series of compounds


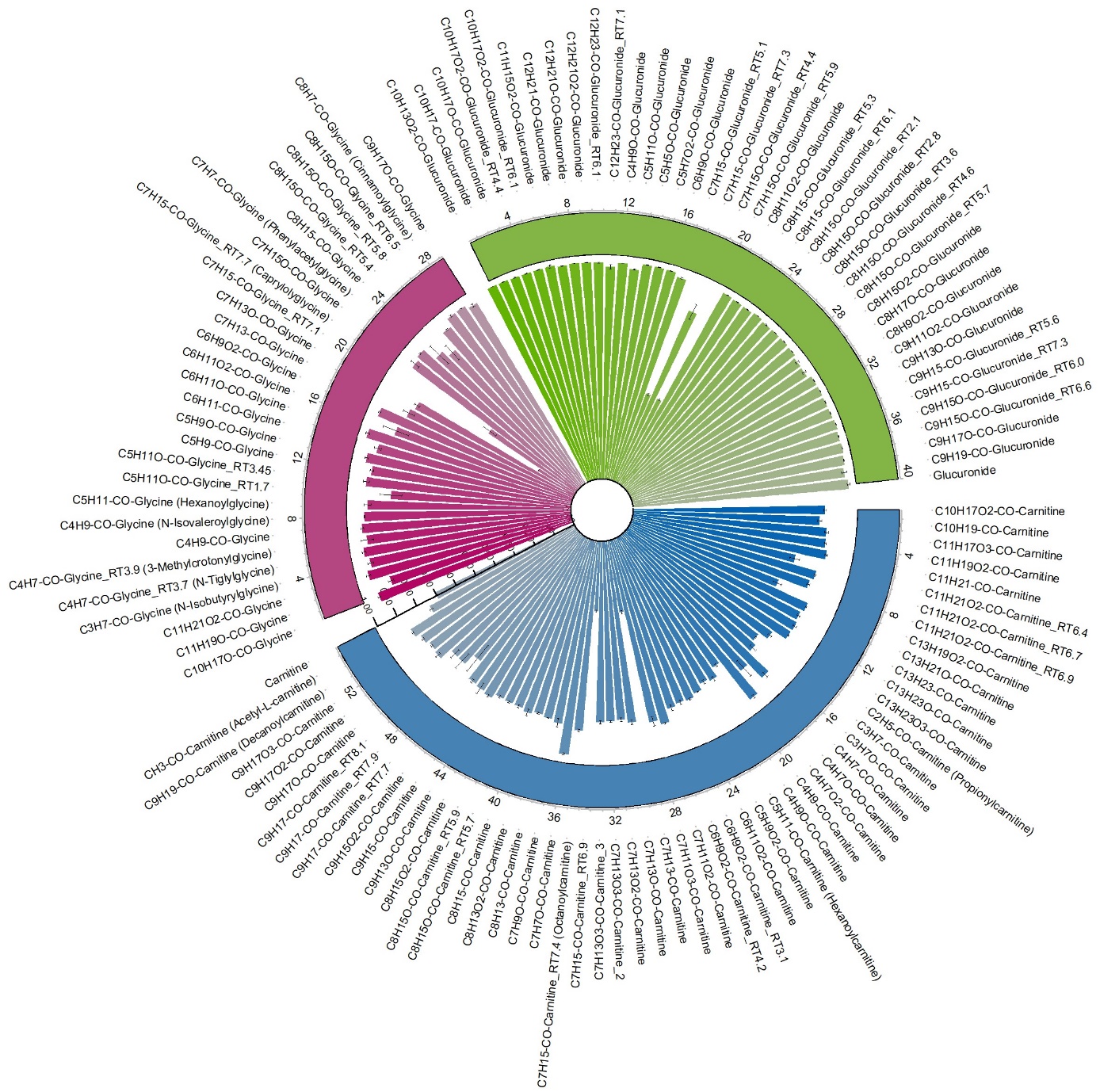
**Figure S13**. ^13^C-enrichment data presented as Circos plots at day 39 for acyl-glycine, acyl-carnitine and acyl-glucuronide series of compounds
