# Supplementary material for: Urinary metabolome dynamics in 13C-labeled mice
Source: Metabolomics. 2025 Dec 29;22(1):15. doi: 10.1007/s11306-025-02391-4 (PMC12748121; doi:10.1007/s11306-025-02391-4)

# Identified metabolites in murine urine

*Metabolites from the chemical library*

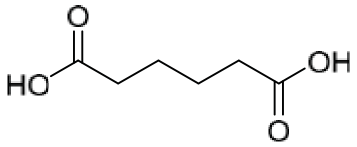


| Metabolite | Adipic acid |
|---|---|
| **Formula** | C6H10O4 |
| **Exact mass** | 146.0579 |

| Ion type | [M-H]- |
|---|---|
| **m/z** | 145.0506 |

## Isotopic patterns

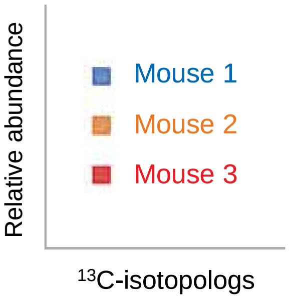


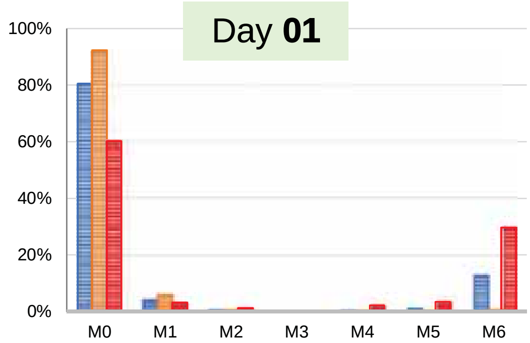


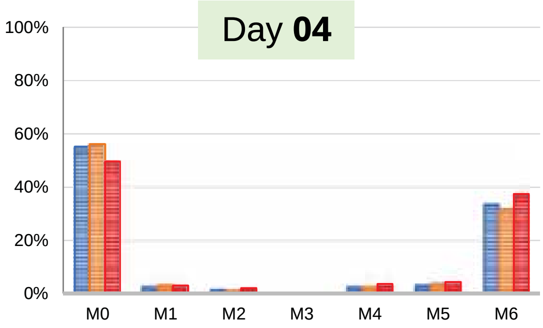


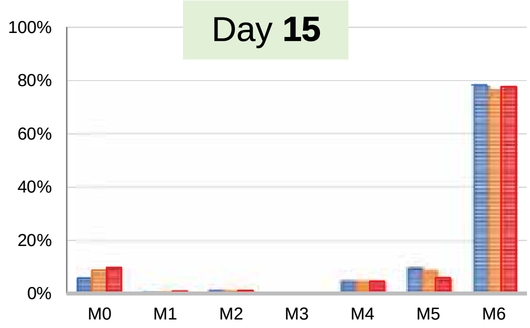


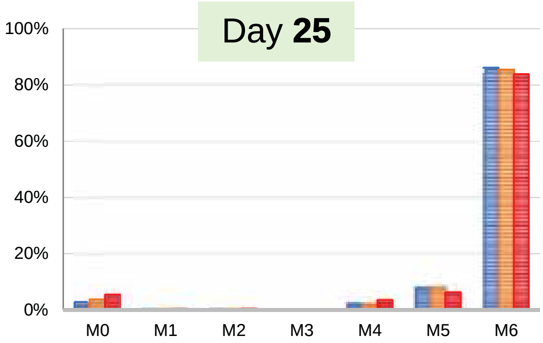


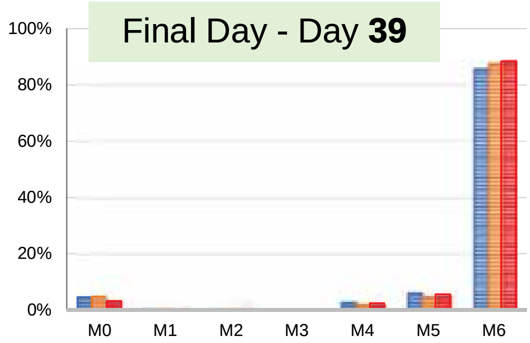


## Fractional $^{13}$C-enrichment

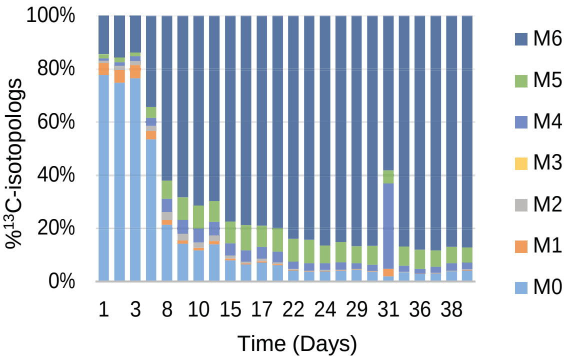


## $^{13}$C-enrichment kinetics

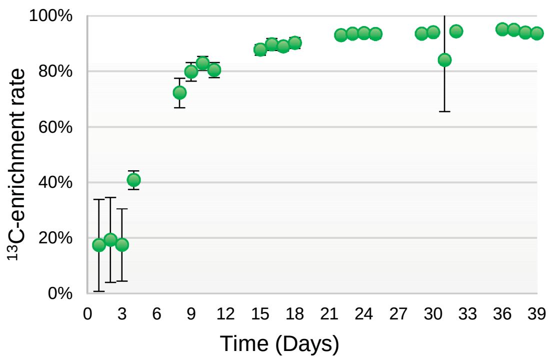

# Identified metabolites in murine urine

*Metabolites from the chemical library*

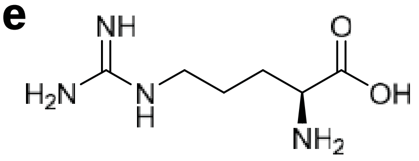

| Metabolite | Arginine |
|---|---|
| Formula | C6H14N4O2 |
| Exact mass | 174.1117 |

| Ion type | [M+H]+ |
|---|---|
| m/z | 175.119 |

## Isotopic patterns

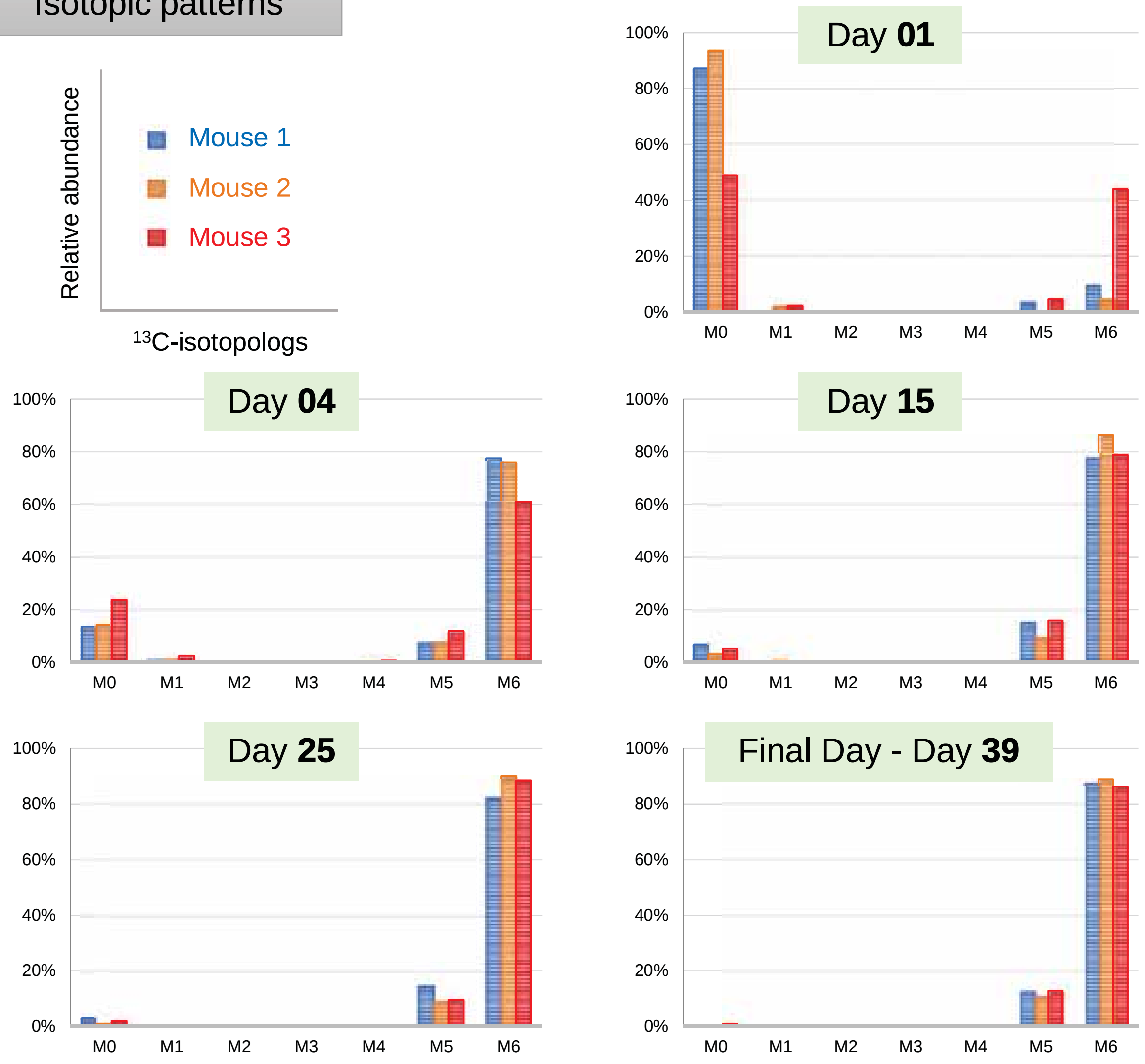

# Identified metabolites in murine urine

*Metabolites from the chemical library*

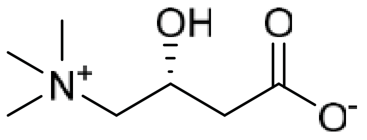


| | |
|---|---|
| **Metabolite** | **Carnitine** |
| **Formula** | C7H15NO3 |
| **Exact mass** | 161.1052 |

| | |
|---|---|
| **Ion type** | [M+H]+ |
| **m/z** | 162.1125 |

## Isotopic patterns

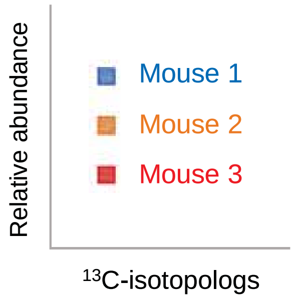


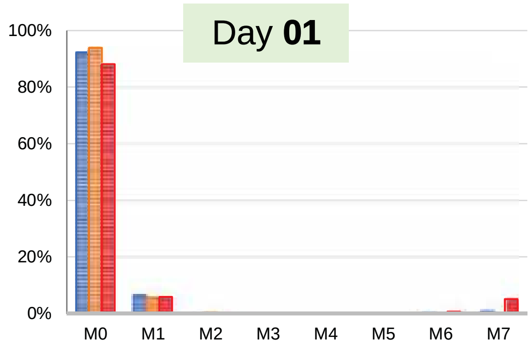


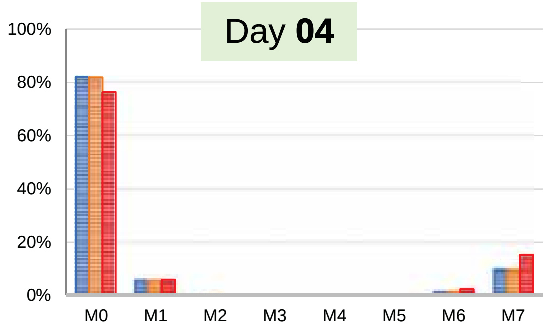


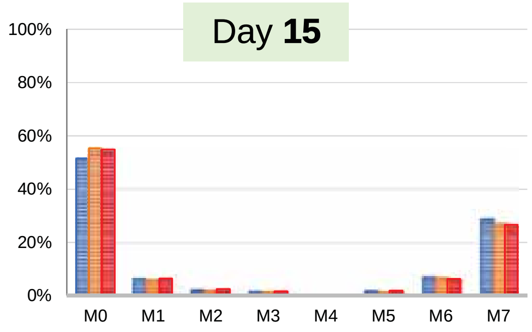


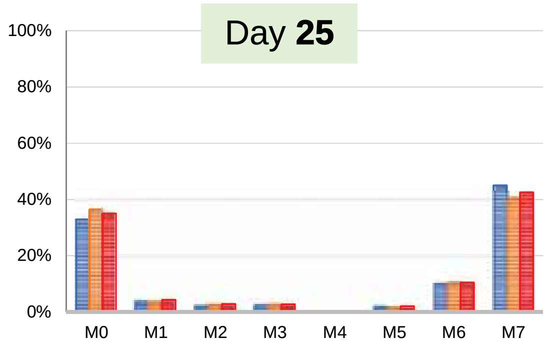


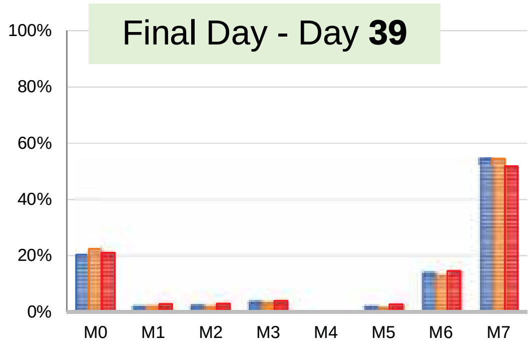


## Fractional 13C-enrichment

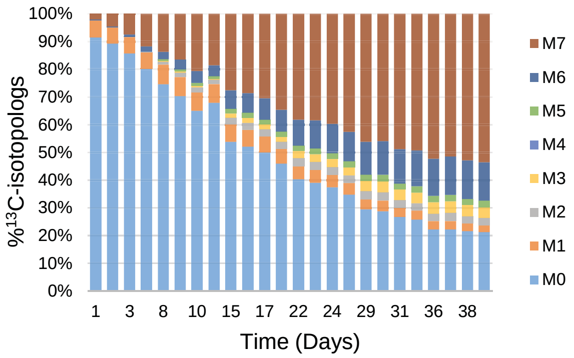


## 13C-enrichment kinetics

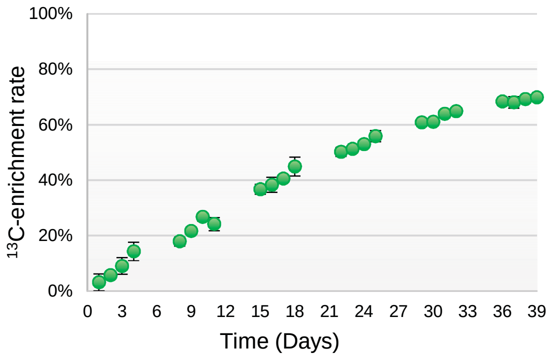

# Identified metabolites in murine urine

*Metabolites from the chemical library*

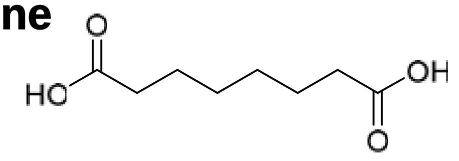


| Metabolite | Suberic acid |
|---|---|
| Formula | C8H14O4 |
| Exact mass | 174.0892 |

| Ion type | [M-H]- |
|---|---|
| m/z | 173.0819 |

## Isotopic patterns

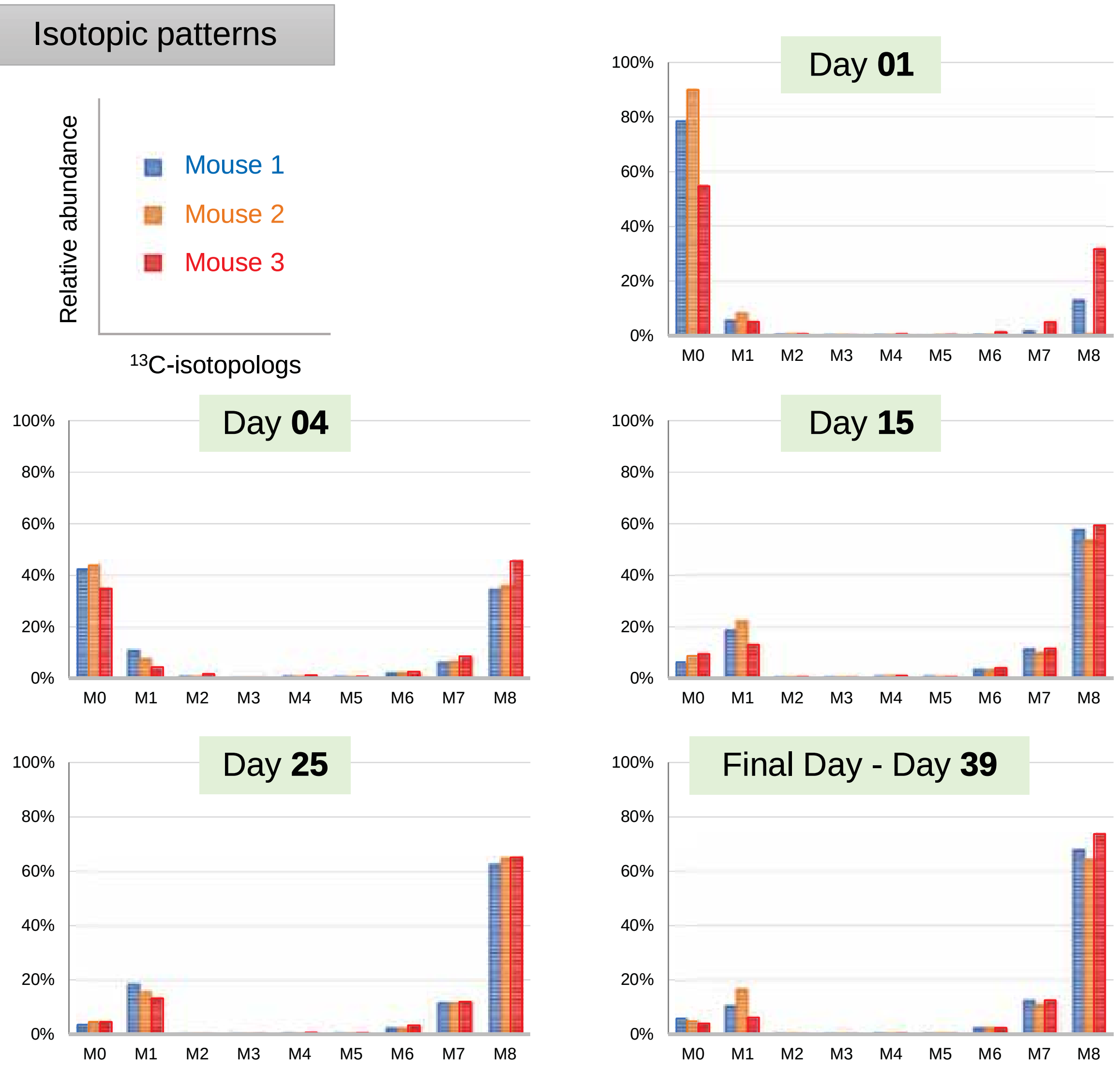

# Identified metabolites in murine urine

*Metabolites from the chemical library*

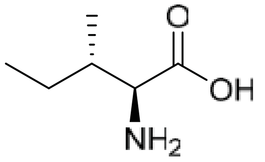


| | |
|---|---|
| **Metabolite** | **Isoleucine** |
| **Formula** | C6H13NO2 |
| **Exact mass** | 131.0946 |

| | |
|---|---|
| **Ion type** | [M-H]- |
| **m/z** | 130.0874 |

## Isotopic patterns

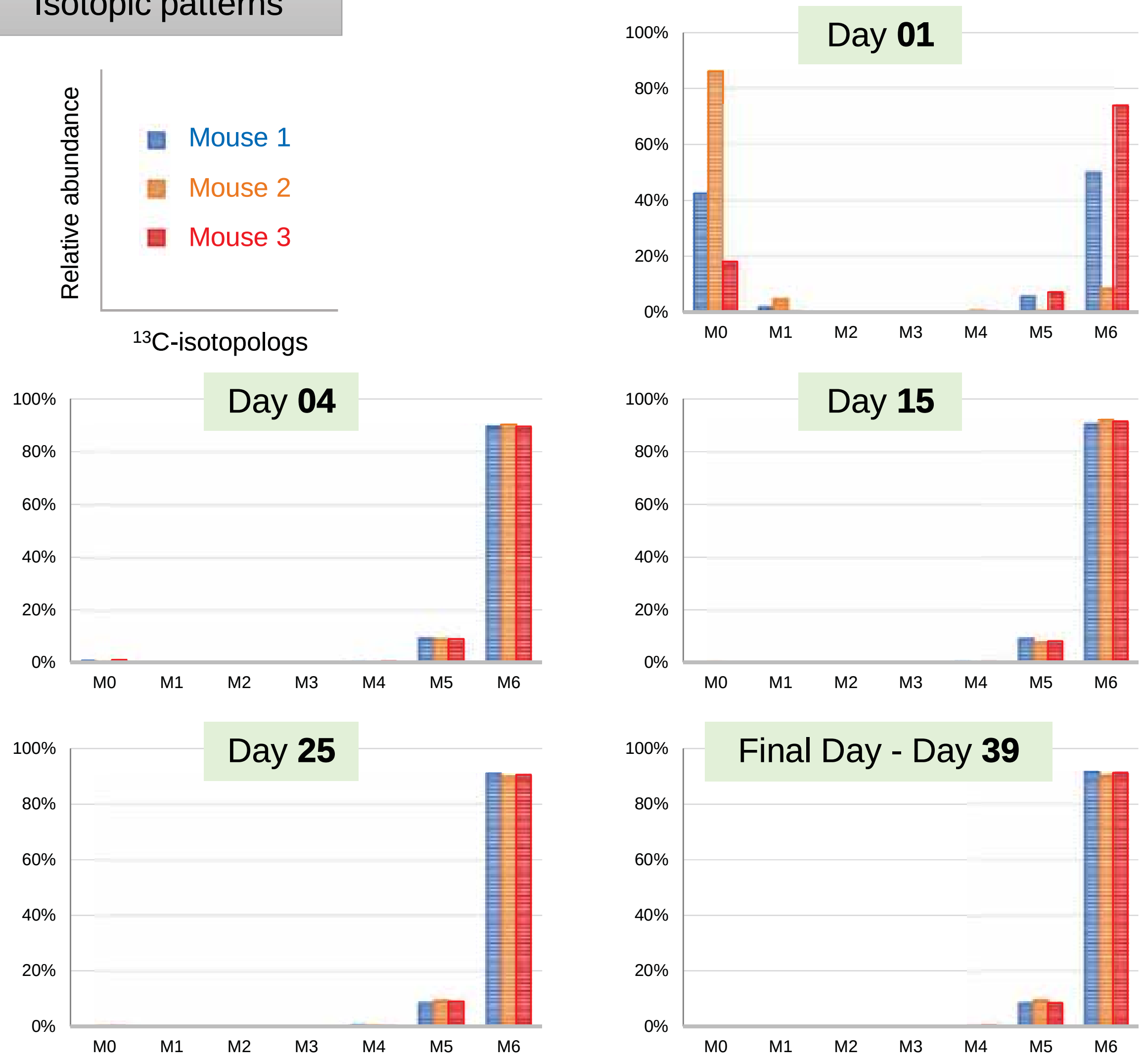

# Identified metabolites in murine urine

*Metabolites from the chemical library*

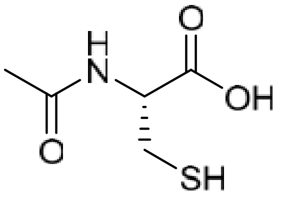


| Metabolite | N-Acetyl-L-Cystein |
|---|---|
| Formula | C5H9NO3S |
| Exact mass | 163.0303 |

| Ion type | [M-H]- |
|---|---|
| m/z | 162.023 |

## Isotopic patterns

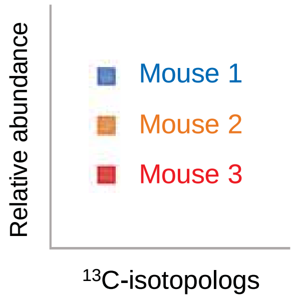


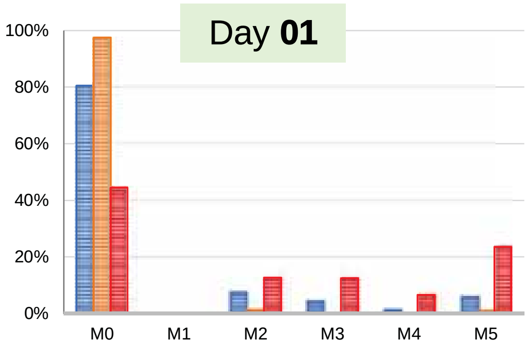


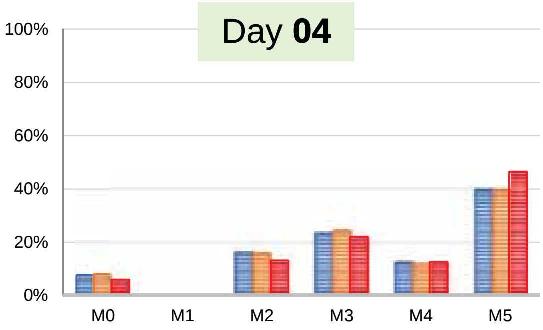


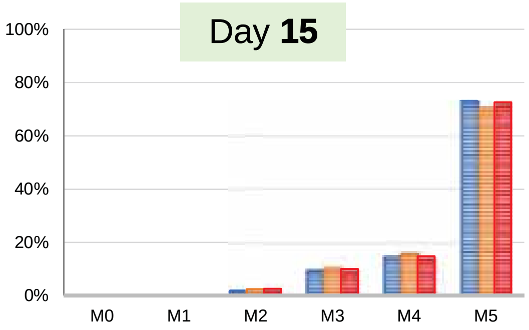


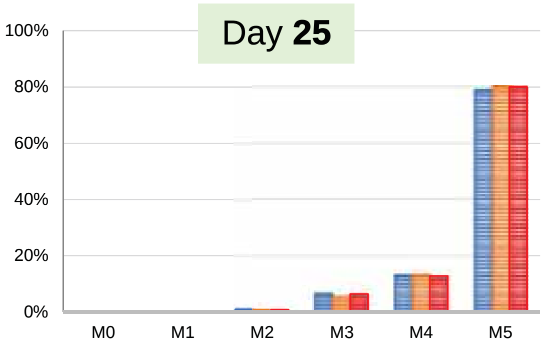


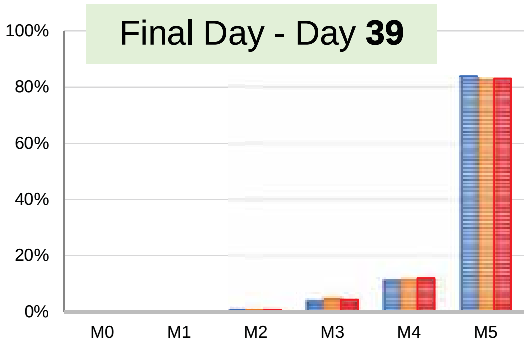


## Fractional $^{13}$C-enrichment

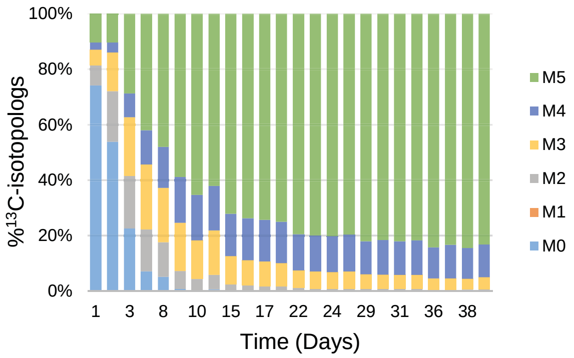


## $^{13}$C-enrichment kinetics

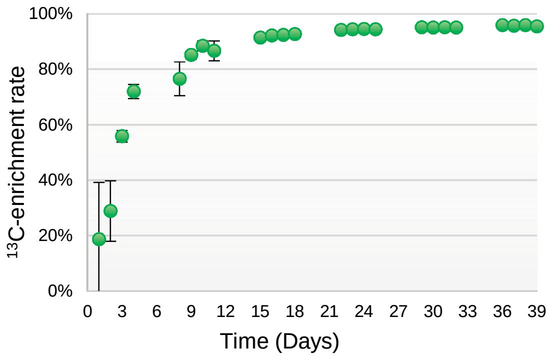

# Identified metabolites in murine urine

*Metabolites from the chemical library*

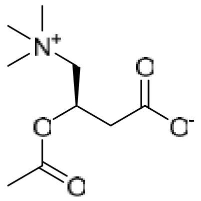

| | |
|---|---|
| **Metabolite** | **Acetyl-L-carnitine**<br>CH3-CO-Carnitine |
| **Formula** | C9H17NO4 |
| **Exact mass** | 203.1157 |

| | |
|---|---|
| **Ion type** | [M+H]+ |
| **m/z** | 204.123 |

## Isotopic patterns

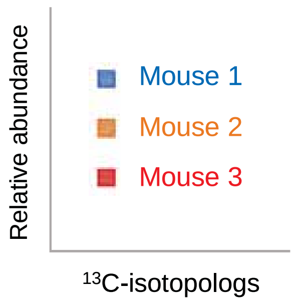


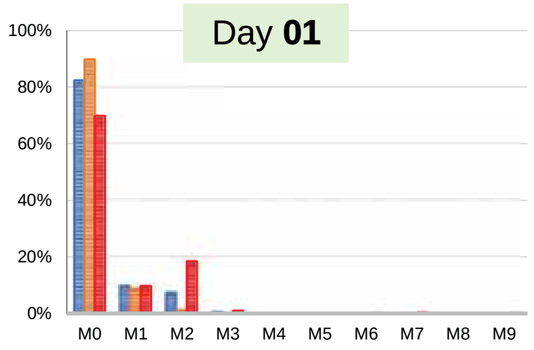


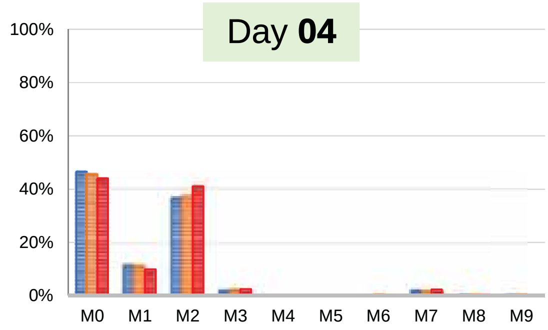


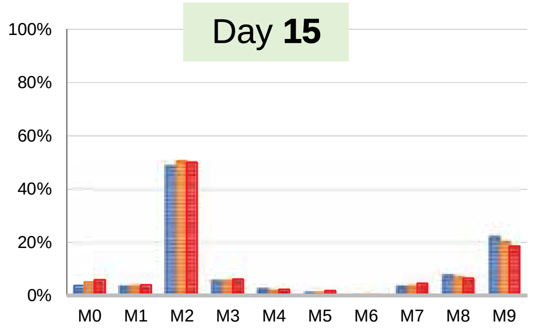


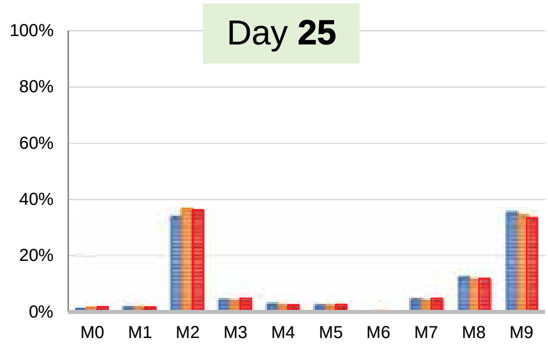


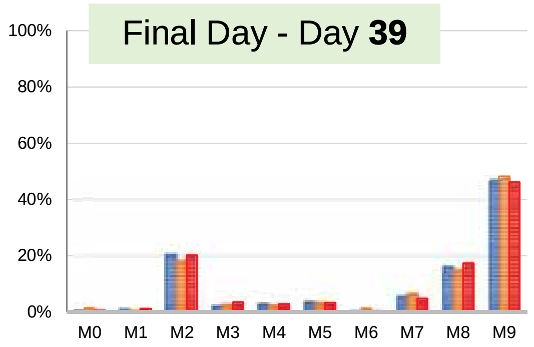


## Fractional $^{13}$C-enrichment

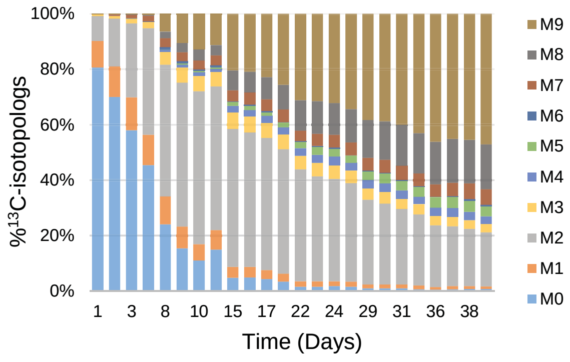


## $^{13}$C-enrichment kinetics

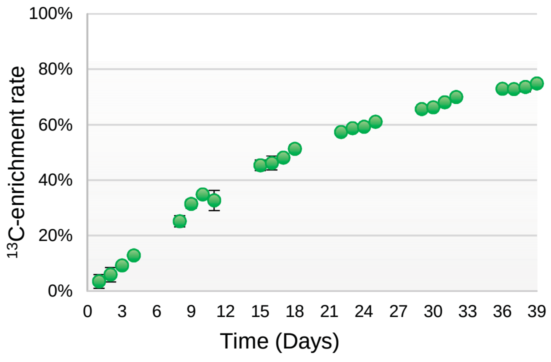

# Identified metabolites in murine urine

*Metabolites from the chemical library*

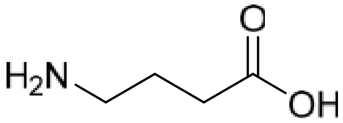


| Metabolite | gamma-aminobutyric acid |
|---|---|
| Formula | C4H9NO2 |
| Exact mass | 103.0633 |

| Ion type | [M+H]+ |
|---|---|
| m/z | 104.0706 |

## Isotopic patterns

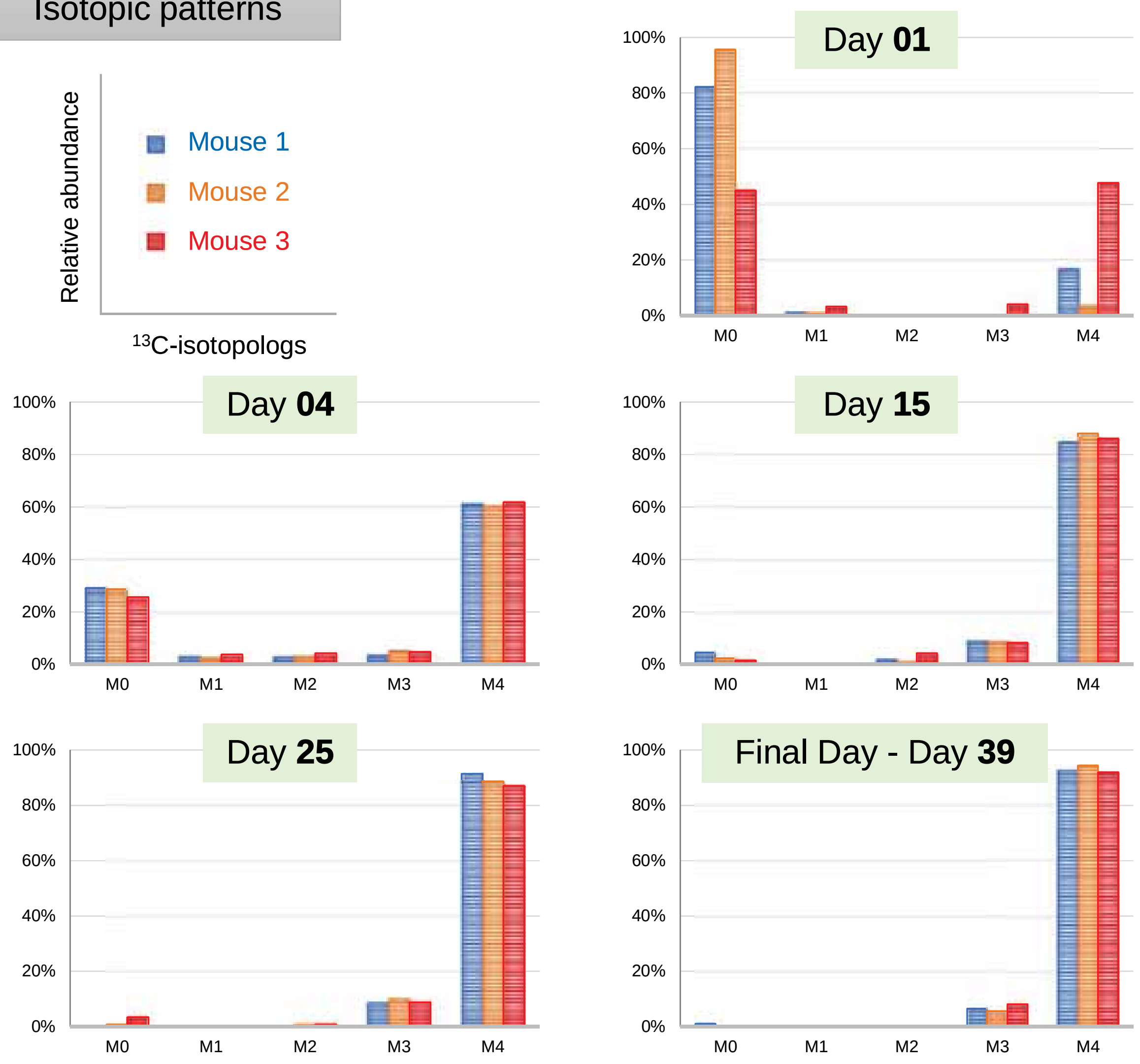

# Identified metabolites in murine urine

*Metabolites from the chemical library*

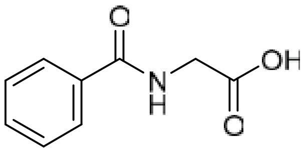


| Metabolite | Hippuric acid |
|---|---|
| Formula | C9H9NO3 |
| Exact mass | 179.0582 |

| Ion type | [M-H]- |
|---|---|
| m/z | 178.051 |

## Isotopic patterns

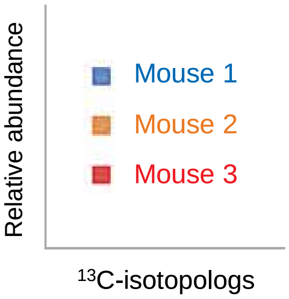


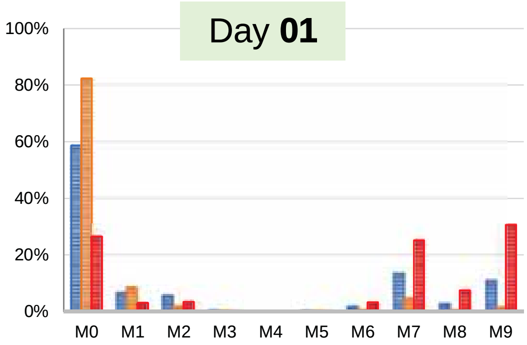


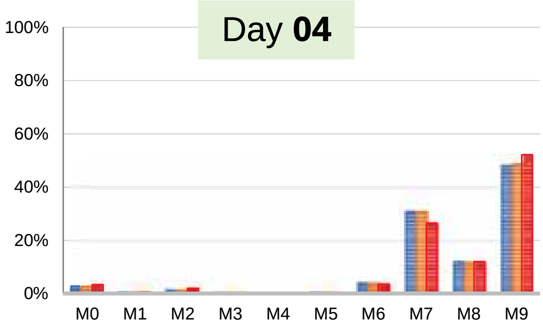


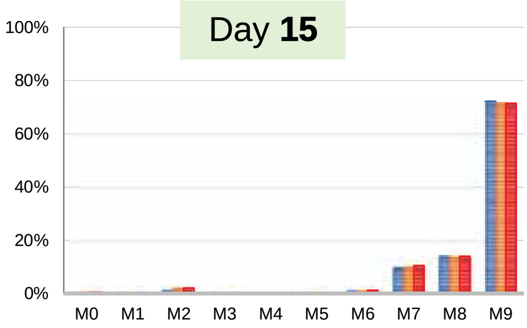


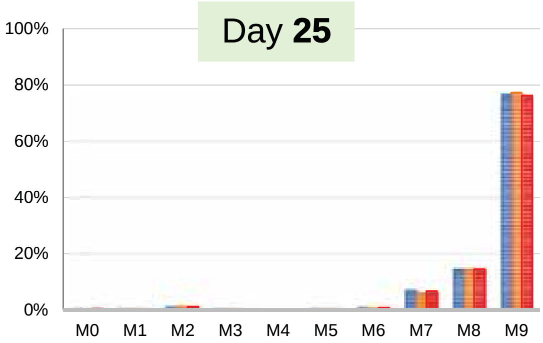


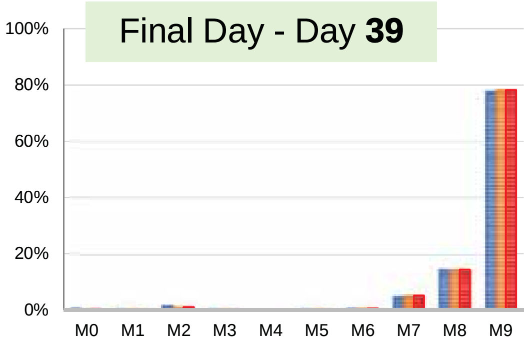


## Fractional $^{13}$C-enrichment

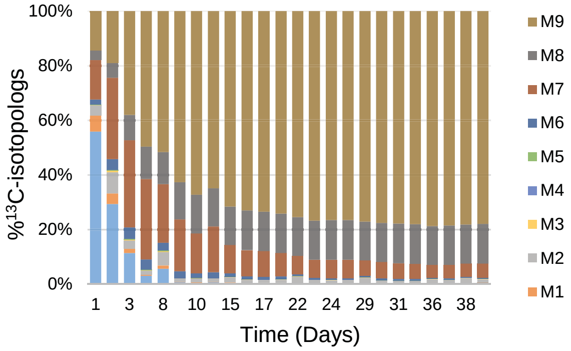


## $^{13}$C-enrichment kinetics

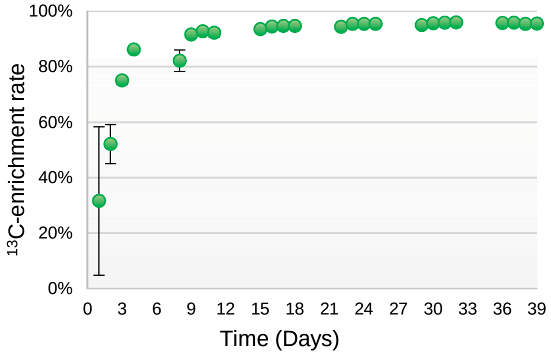

# Identified metabolites in murine urine

*Metabolites from the chemical library*

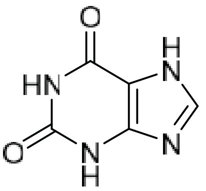

| Metabolite | Xanthine |
|---|---|
| **Formula** | C5H4N4O2 |
| **Exact mass** | 152.0334 |

| Ion type | [M+H]+ |
|---|---|
| **m/z** | 153.0407 |

## Isotopic patterns

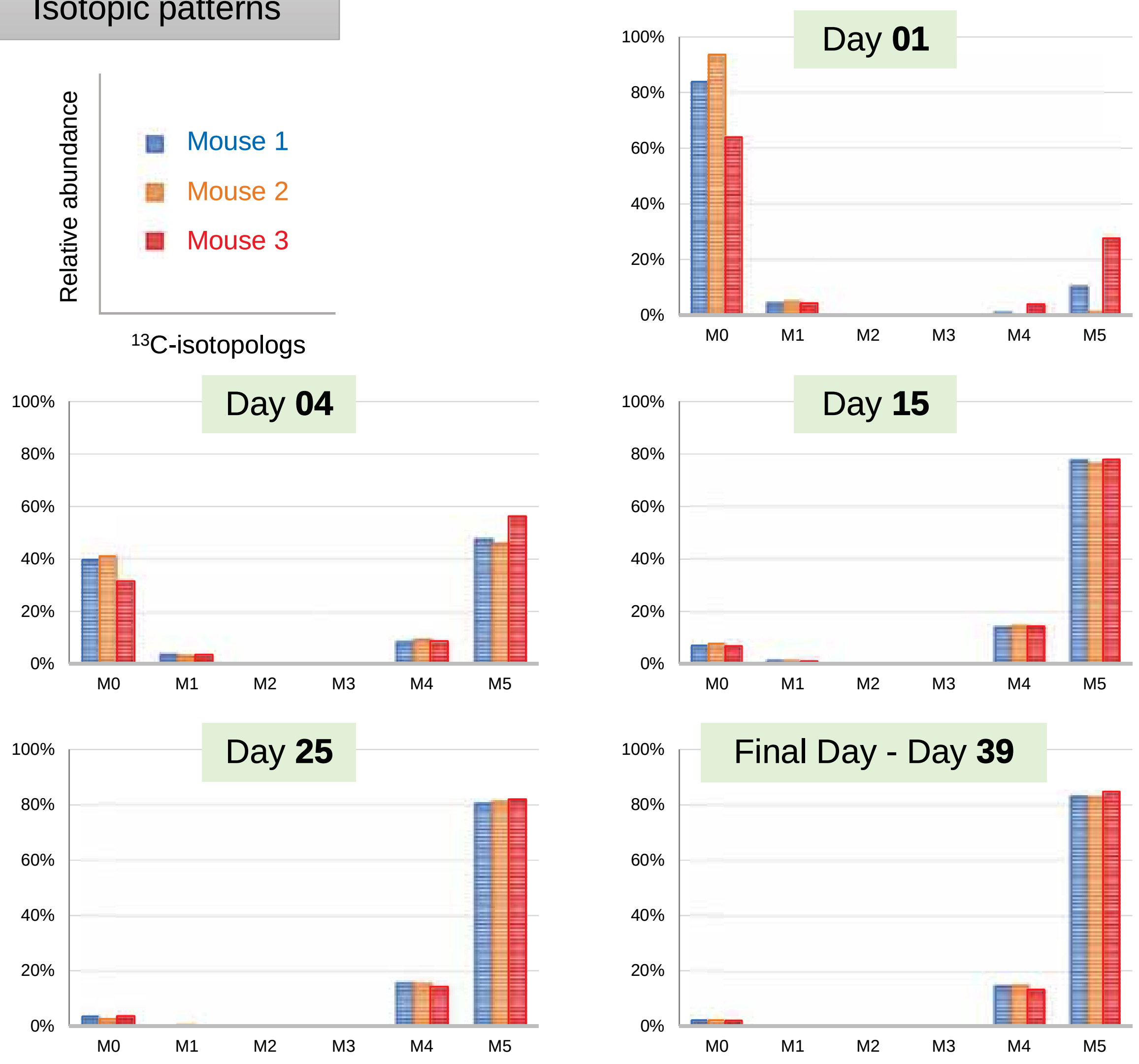


## Fractional $^{13}$C-enrichment

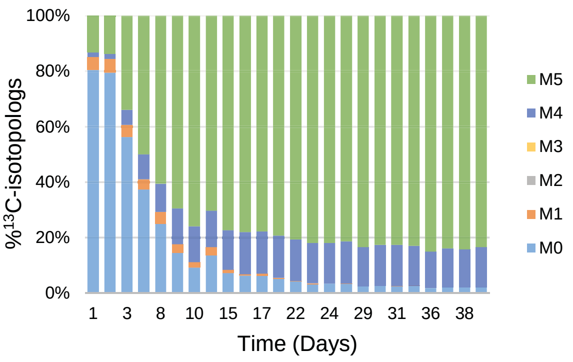


## $^{13}$C-enrichment kinetics

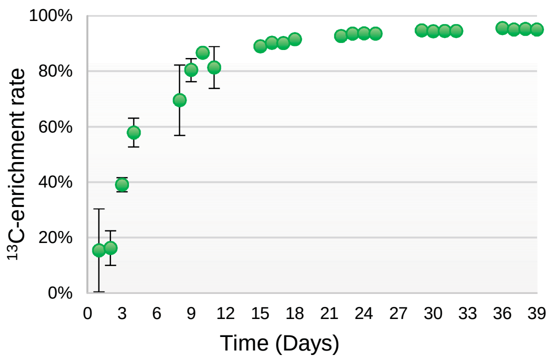

# Identified metabolites in murine urine

*Metabolites from the chemical library*

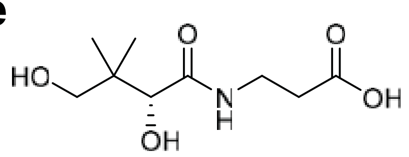


| Metabolite | Pantothenic acid |
|---|---|
| Formula | C9H17NO5 |
| Exact mass | 219.1107 |

| Ion type | [M+H]+ |
|---|---|
| m/z | 220.118 |

## Isotopic patterns

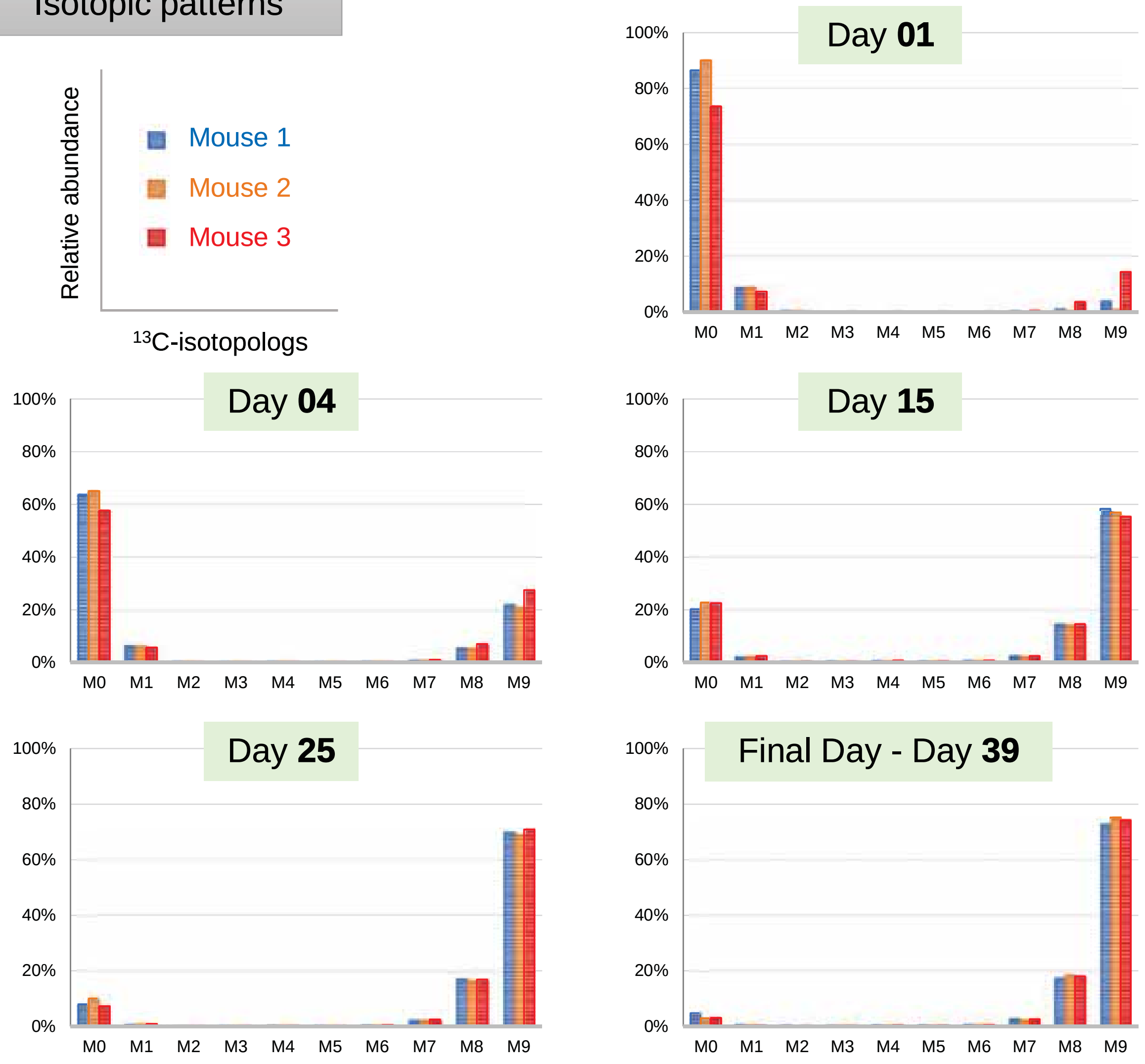


## Fractional $^{13}$C-enrichment

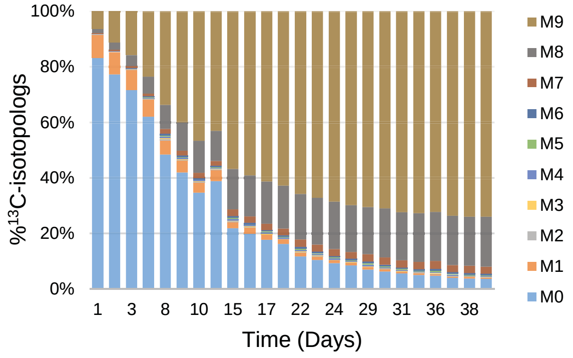


## $^{13}$C-enrichment kinetics

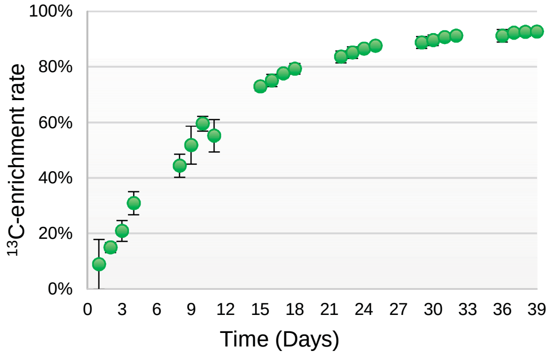

# Identified metabolites in murine urine

*Metabolites from the chemical library*

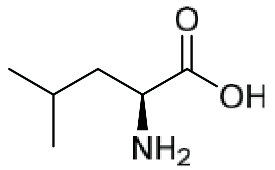


| Metabolite | Leucine |
|---|---|
| Formula | C6H13NO2 |
| Exact mass | 131.0946 |

| Ion type | [M+H]+ |
|---|---|
| m/z | 132.1019 |

## Isotopic patterns

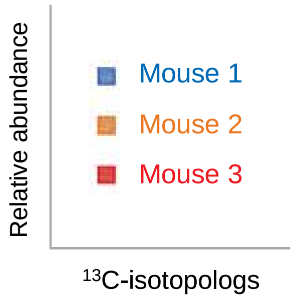


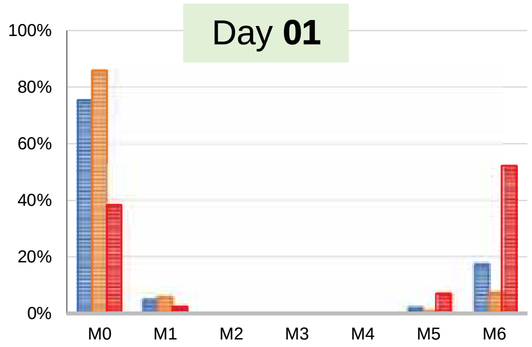


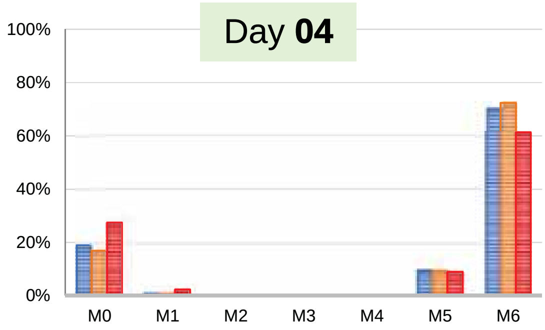


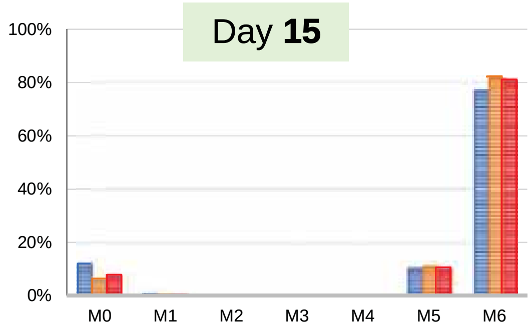


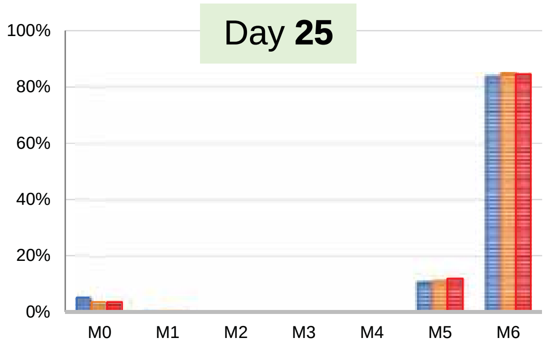


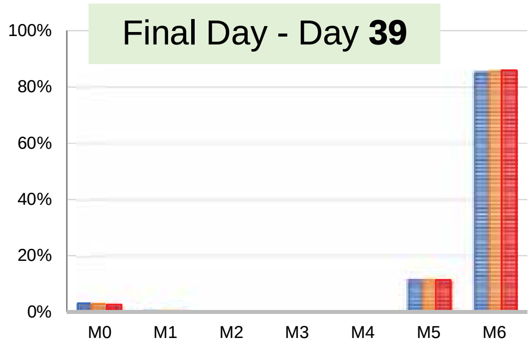


## Fractional $^{13}$C-enrichment

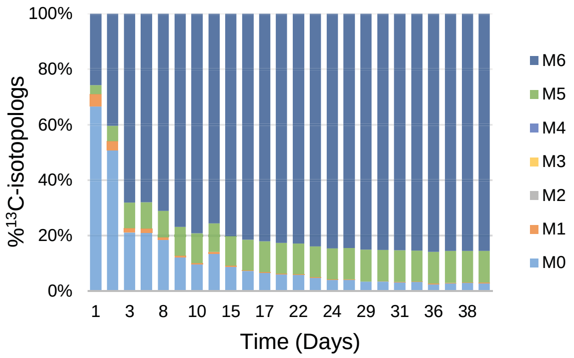


## $^{13}$C-enrichment kinetics

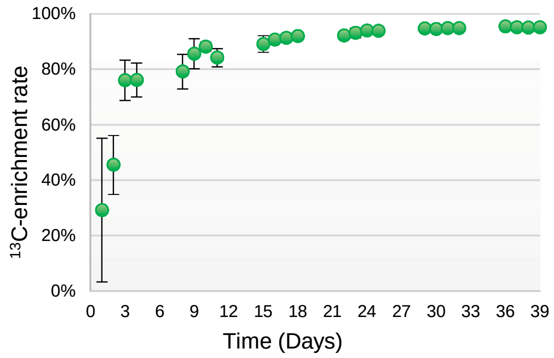

# Identified metabolites in murine urine

*Metabolites from the chemical library*

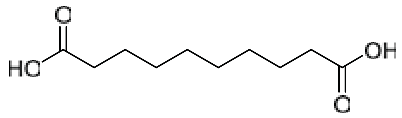


| Metabolite | Sebacic acid |
|---|---|
| Formula | C10H18O4 |
| Exact mass | 202.1205 |

| Ion type | [M-H]- |
|---|---|
| m/z | 201.1132 |

## Isotopic patterns

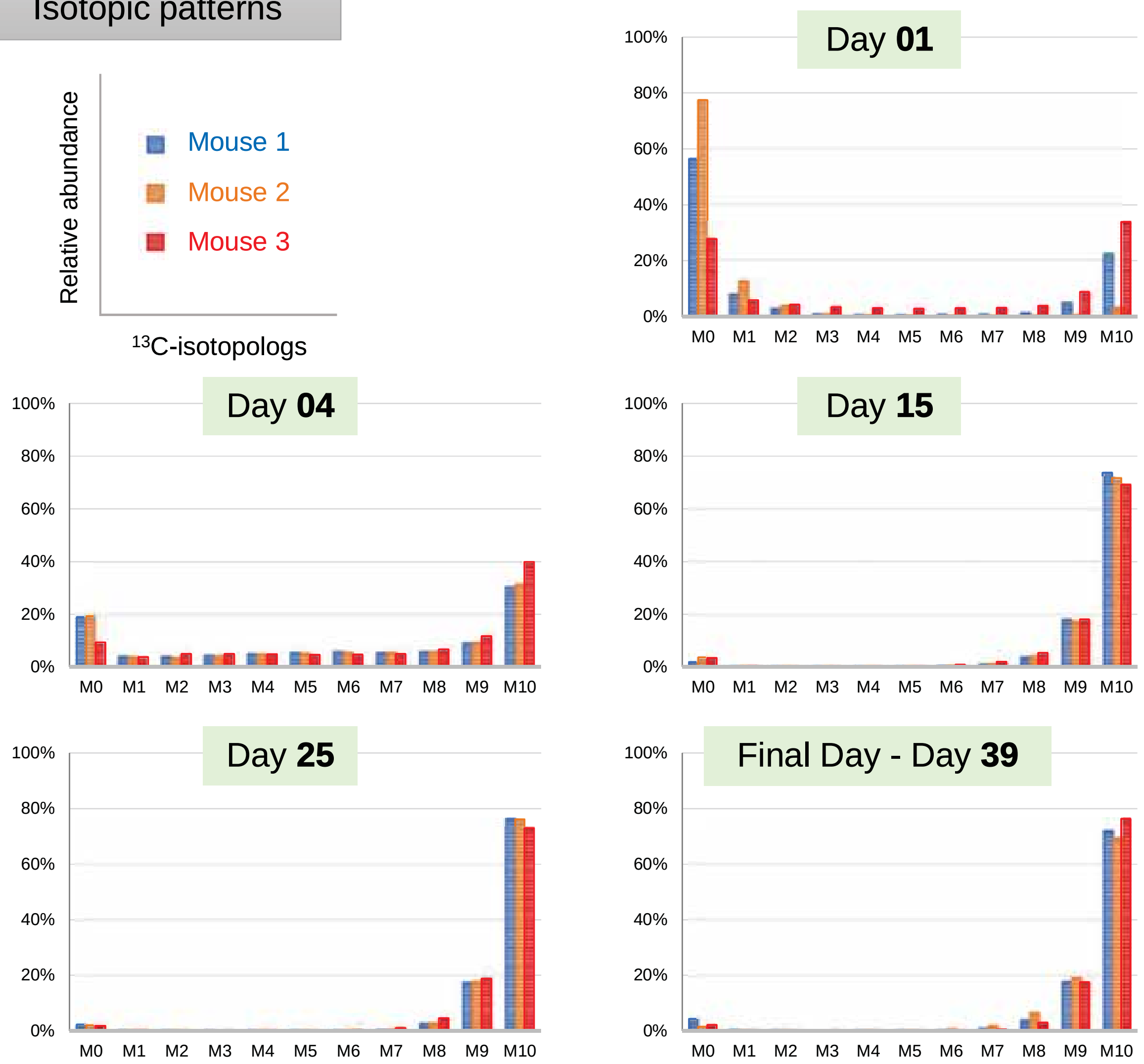


## Fractional $^{13}$C-enrichment

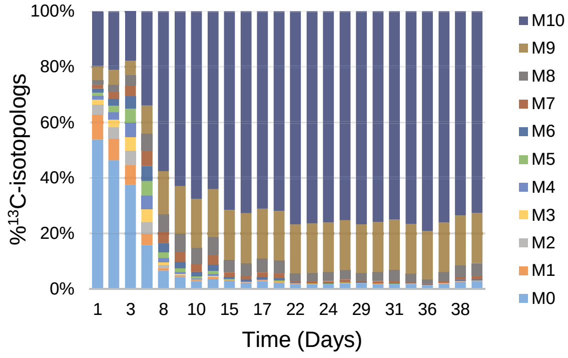


## $^{13}$C-enrichment kinetics

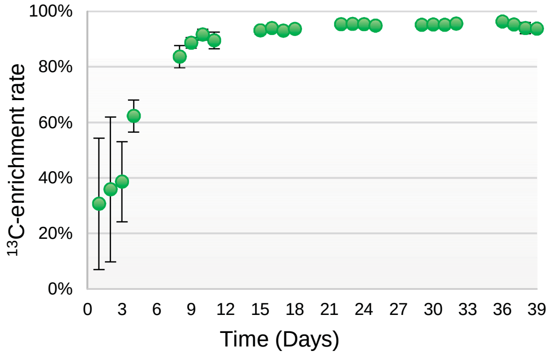

# Identified metabolites in murine urine

*Metabolites from the chemical library*

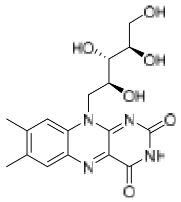

| Metabolite | Riboflavin |
|---|---|
| Formula | C17H20N4O6 |
| Exact mass | 376.1383 |

| Ion type | [M+H]+ |
|---|---|
| m/z | 377.1456 |

## Isotopic patterns

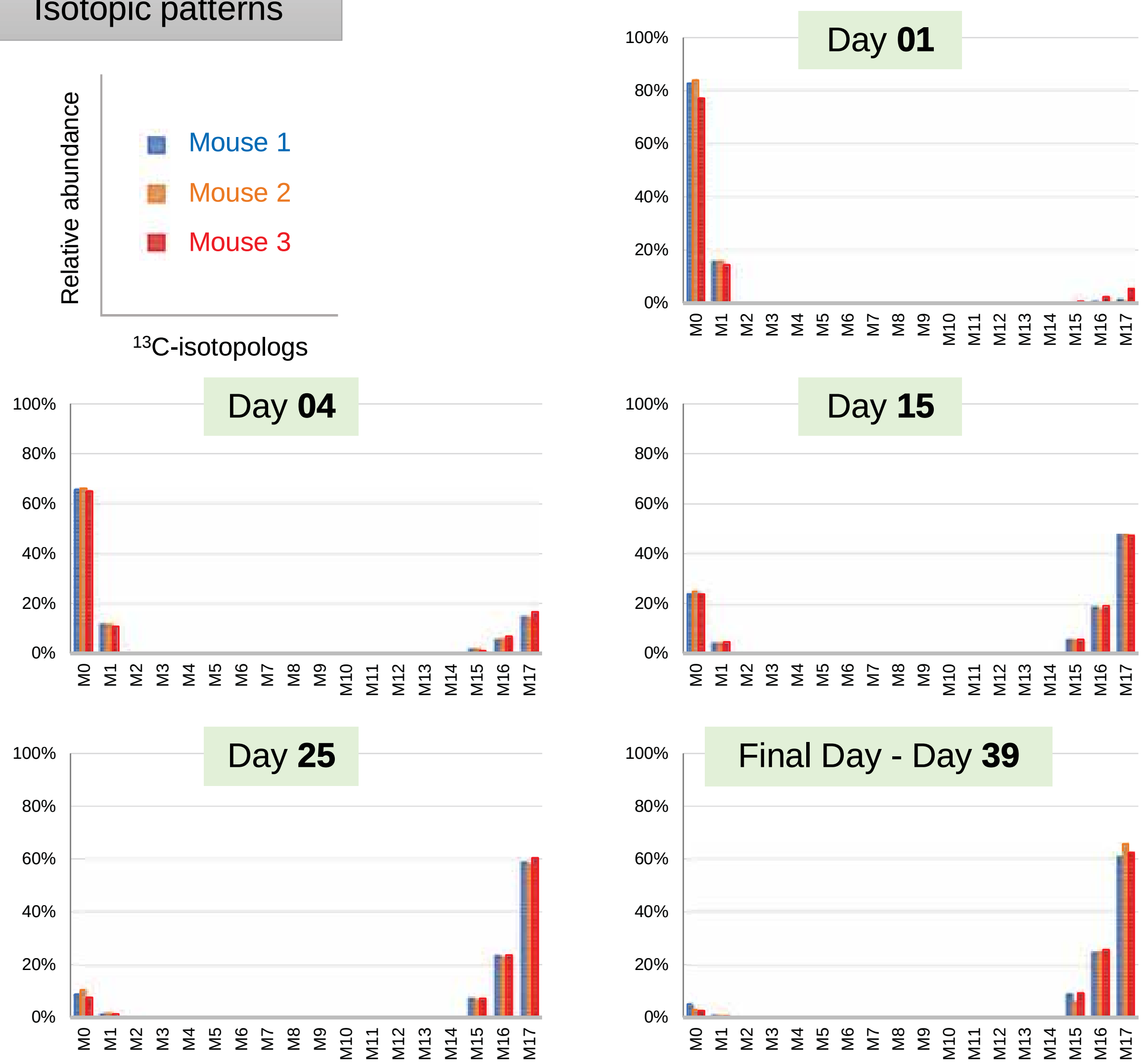


## Fractional $^{13}$C-enrichment

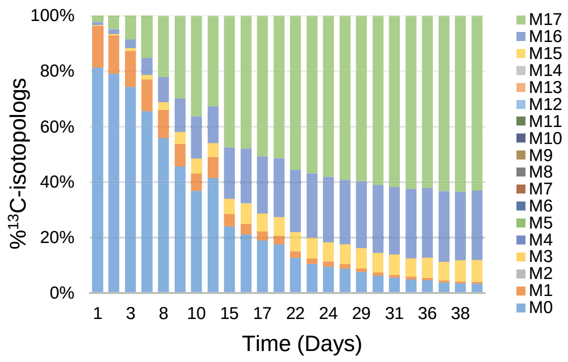


## $^{13}$C-enrichment kinetics

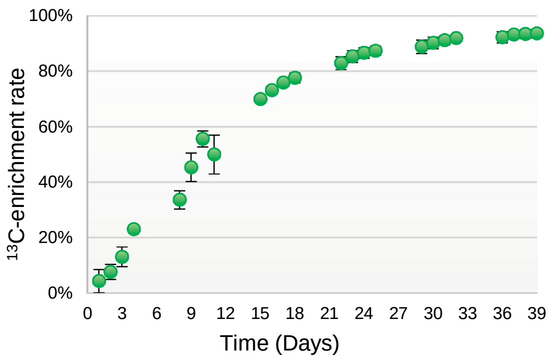

# Identified metabolites in murine urine

*Metabolites from the chemical library*

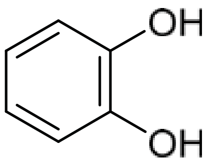


| | |
|---|---|
| **Metabolite** | **Pyrocatechol** |
| **Formula** | C6H6O2 |
| **Exact mass** | 110.0368 |

| | |
|---|---|
| **Ion type** | [M-H]- |
| **m/z** | 109.0295 |

## Isotopic patterns

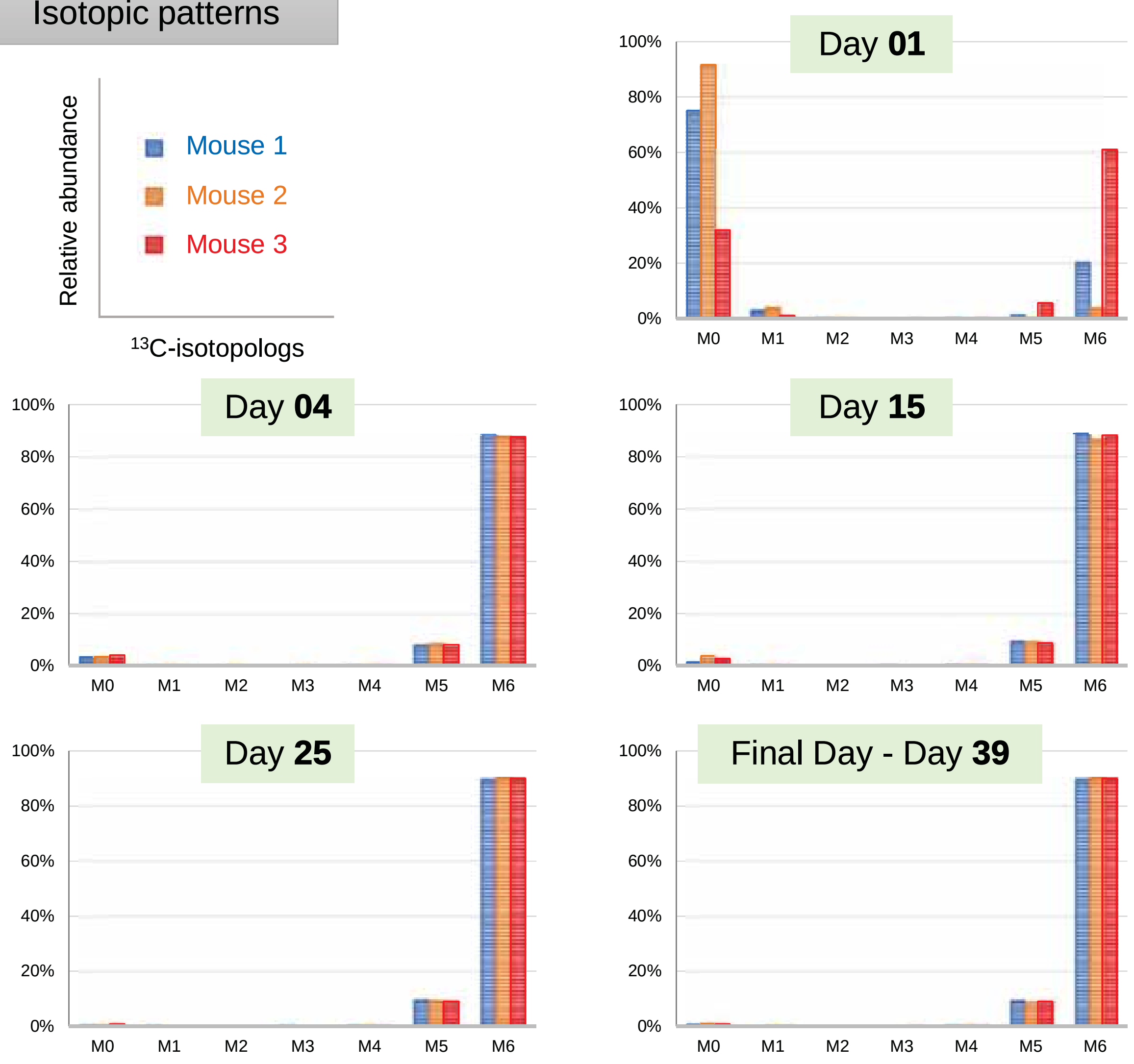

# Identified metabolites in murine urine

*Metabolites from the chemical library*

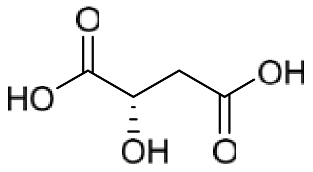


| Metabolite | Malic acid |
|---|---|
| Formula | C4H6O5 |
| Exact mass | 134.0215 |

| Ion type | [M-H]- |
|---|---|
| m/z | 133.0142 |

## Isotopic patterns

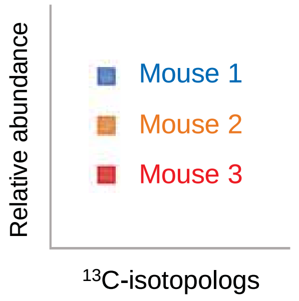


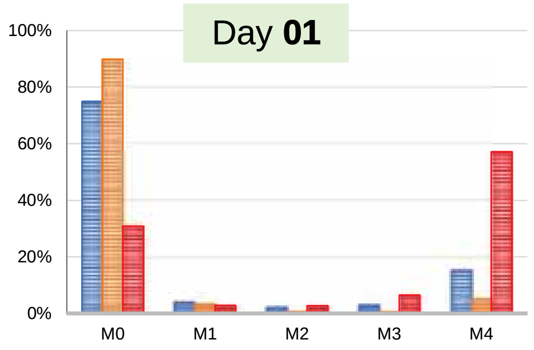


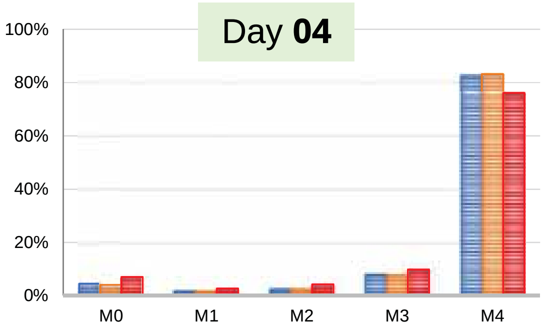


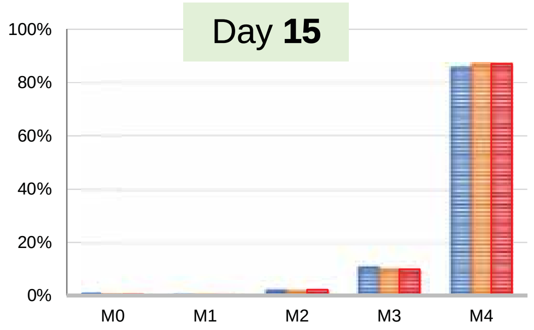


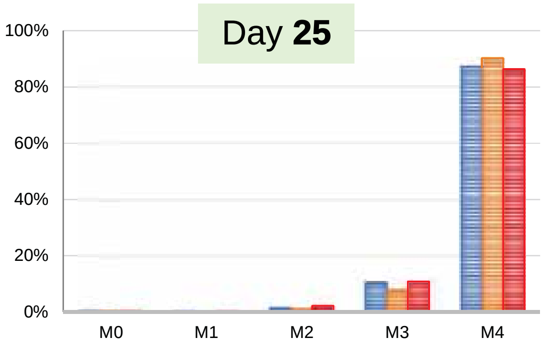


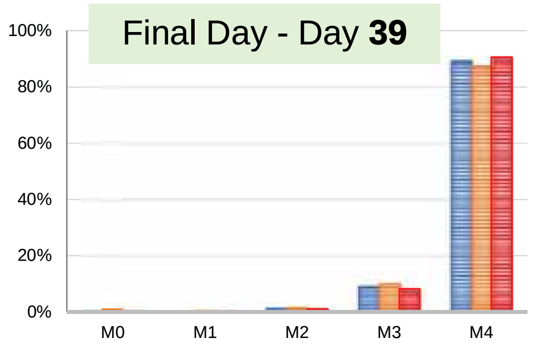


## Fractional $^{13}$C-enrichment

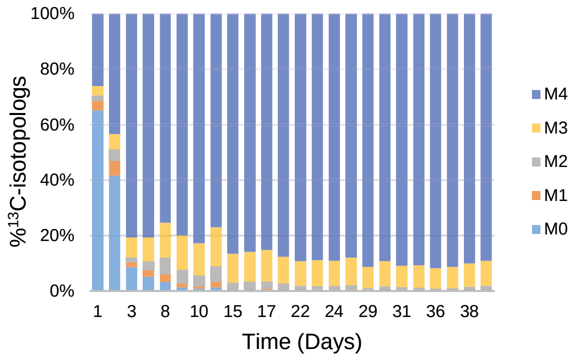


## $^{13}$C-enrichment kinetics

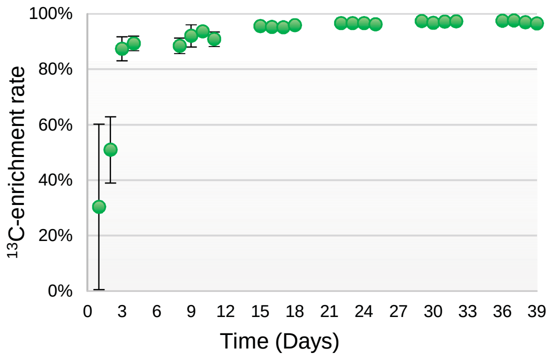

# Identified metabolites in murine urine

*Metabolites from the chemical library*

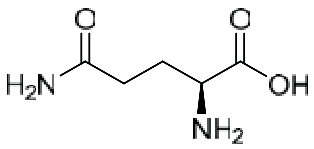


| **Metabolite** | **Glutamine** |
|---|---|
| **Formula** | C5H10N2O3 |
| **Exact mass** | 146.0691 |

| **Ion type** | [M+H]+ |
|---|---|
| **m/z** | 147.0764 |

## Isotopic patterns

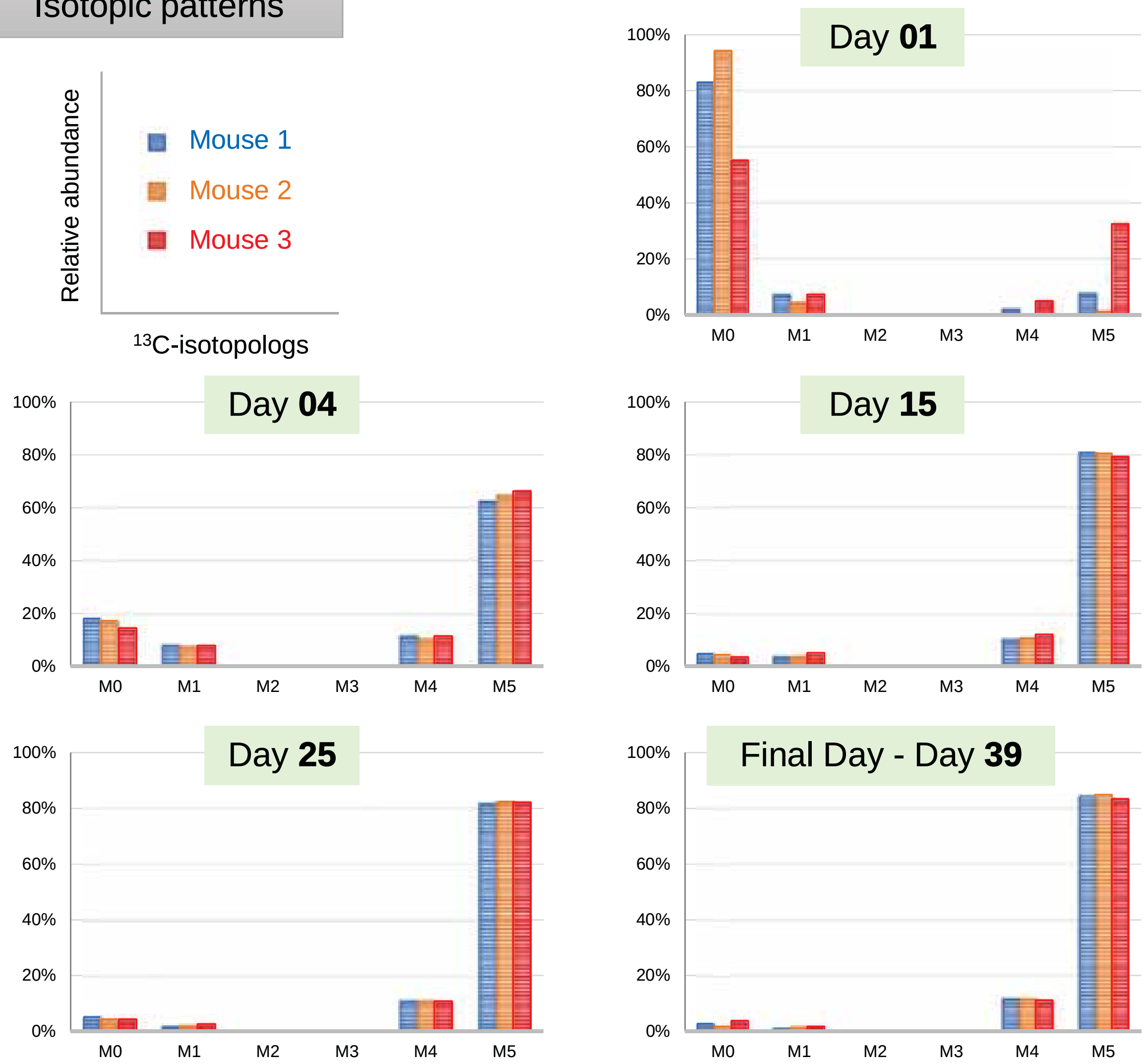


## Fractional $^{13}$C-enrichment

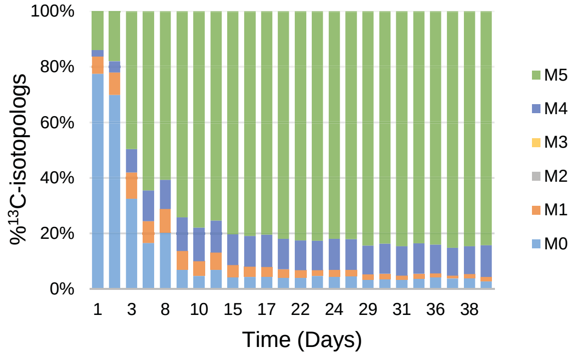


## $^{13}$C-enrichment kinetics

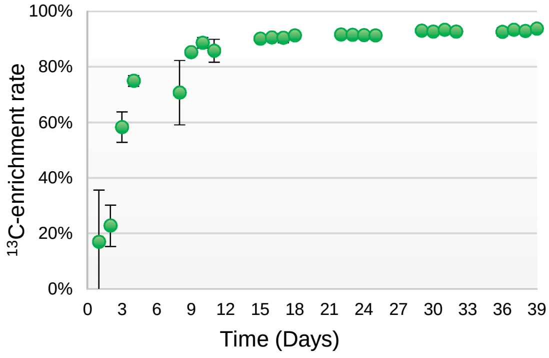

# Identified metabolites in murine urine

*Metabolites from the chemical library*

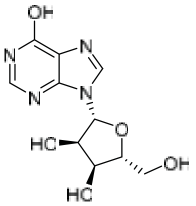

| Metabolite | Inosine |
|---|---|
| Formula | C10H12N4O5 |
| Exact mass | 268.0808 |

| Ion type | [M-H]- |
|---|---|
| m/z | 267.0735 |

## Isotopic patterns

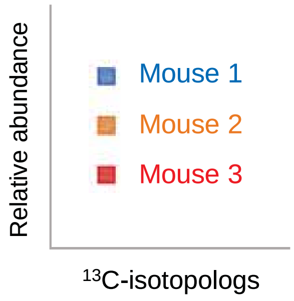


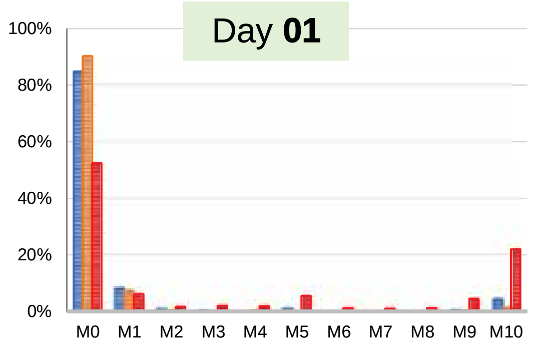


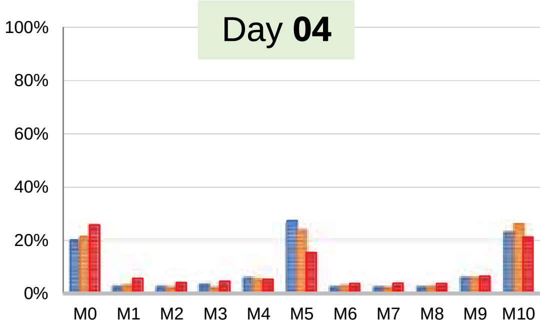


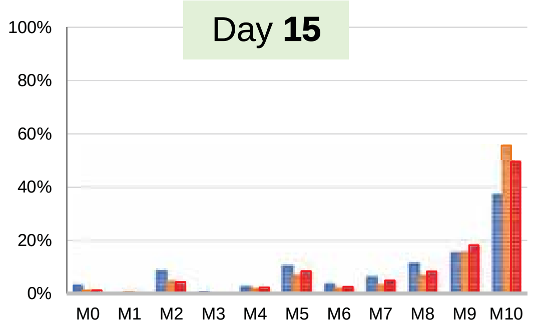


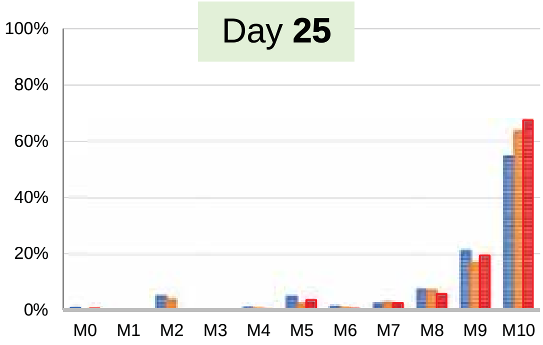


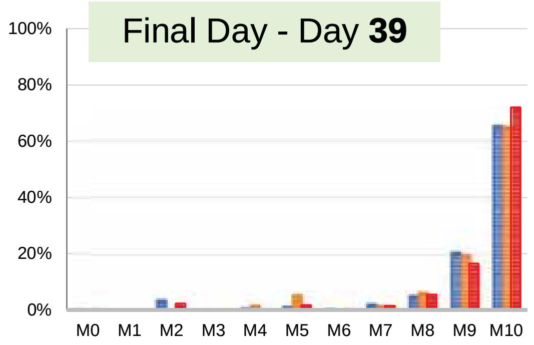

# Identified metabolites in murine urine

*Metabolites from the chemical library*

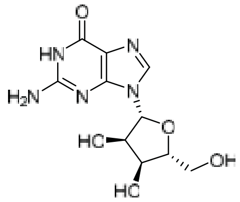

| Metabolite | Guanosine |
|---|---|
| Formula | C10H13N5O5 |
| Exact mass | 283.0917 |

| Ion type | [M+H]+ |
|---|---|
| m/z | 284.099 |

## Isotopic patterns

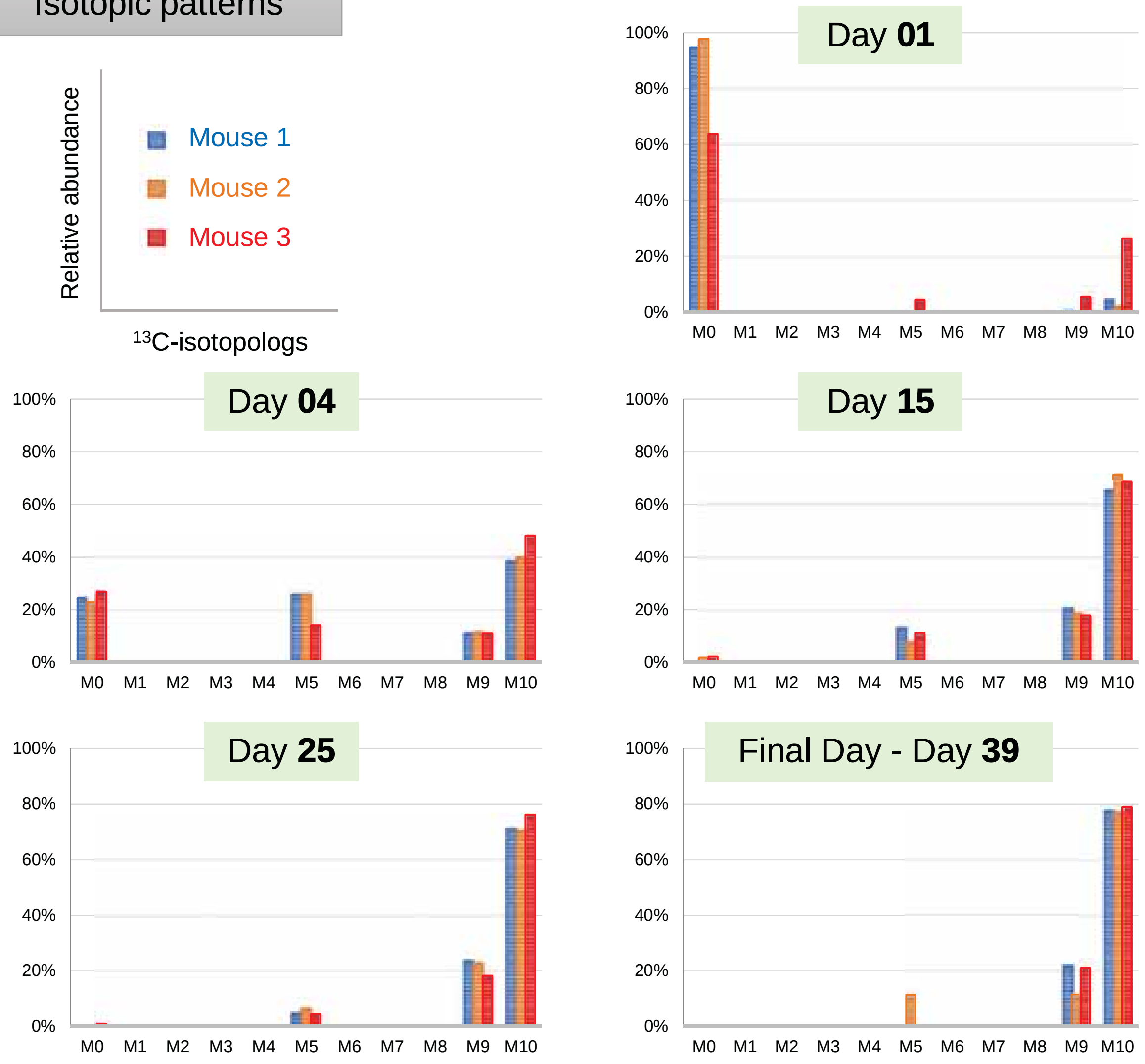


## Fractional $^{13}$C-enrichment

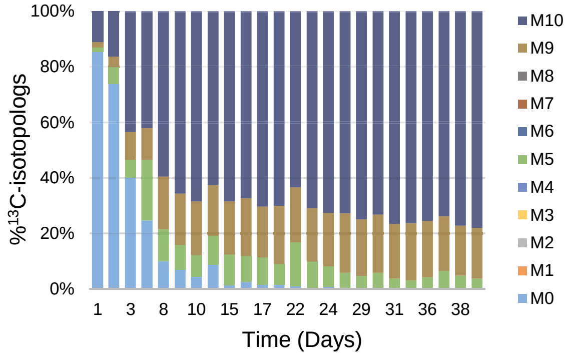


## $^{13}$C-enrichment kinetics

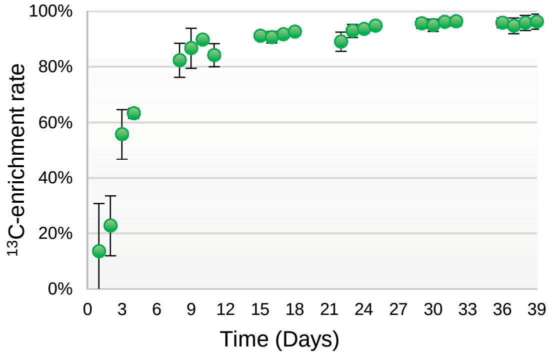

# Identified metabolites in murine urine

*Metabolites from the chemical library*

| Metabolite | Adenosine |
|---|---|
| **Formula** | C10H13N5O4 |
| **Exact mass** | 267.0967 |

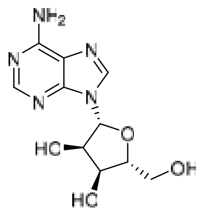


| Ion type | [M+H]+ |
|---|---|
| **m/z** | 268.104 |

## Isotopic patterns

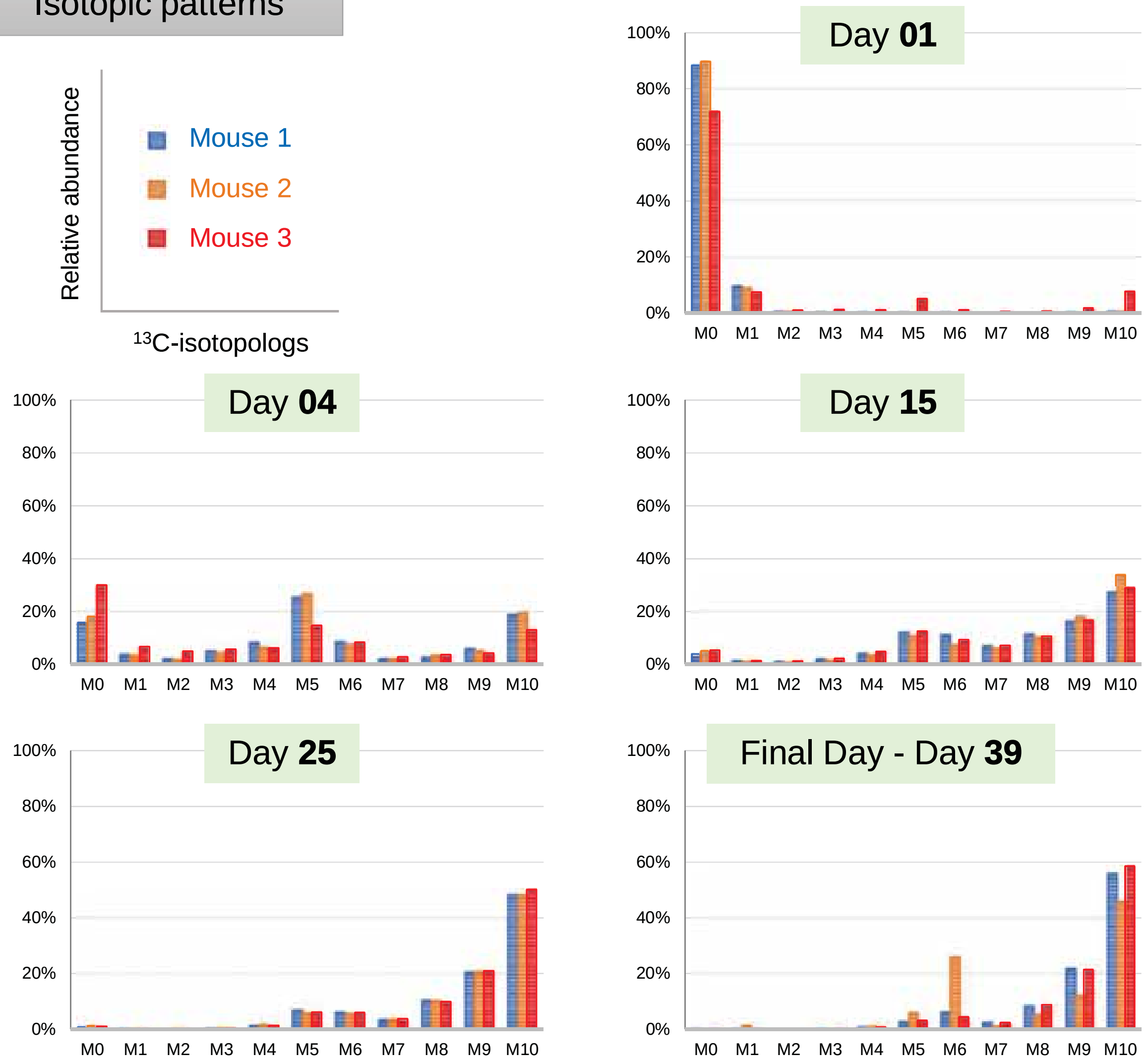


## Fractional $^{13}$C-enrichment

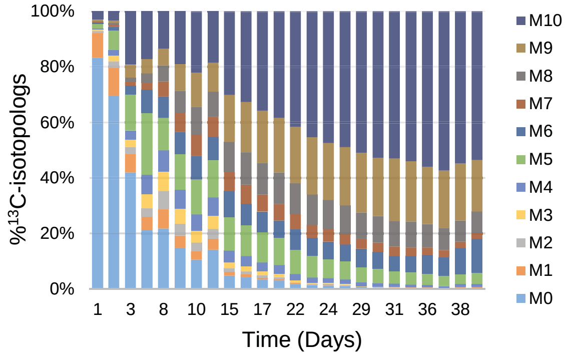


## $^{13}$C-enrichment kinetics

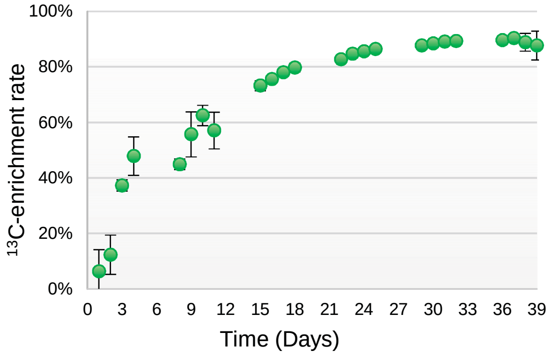

# Identified metabolites in murine urine

*Metabolites from the chemical library*

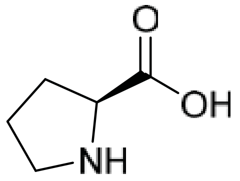


| | |
|---|---|
| **Metabolite** | **Proline** |
| **Formula** | C5H9NO2 |
| **Exact mass** | 115.0633 |

| | |
|---|---|
| **Ion type** | [M+H]+ |
| **m/z** | 116.0706 |

## Isotopic patterns

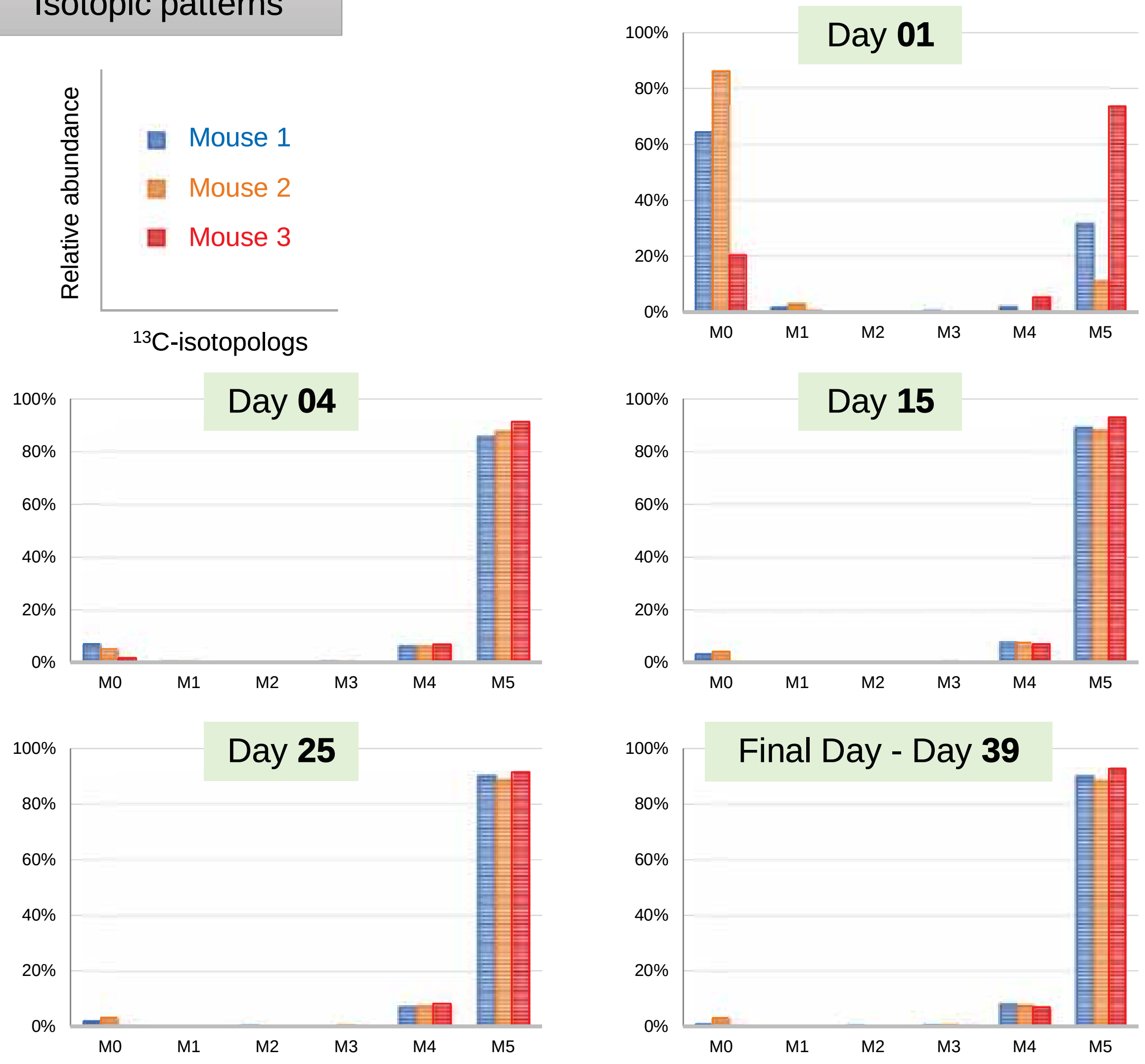

# Identified metabolites in murine urine

*Metabolites from the chemical library*

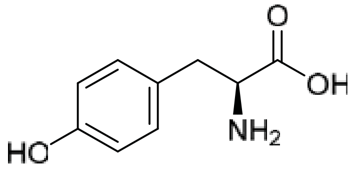


| Metabolite | Tyrosine |
|---|---|
| Formula | C9H11NO3 |
| Exact mass | 181.0739 |

| Ion type | [M+H]+ |
|---|---|
| m/z | 182.0812 |

## Isotopic patterns

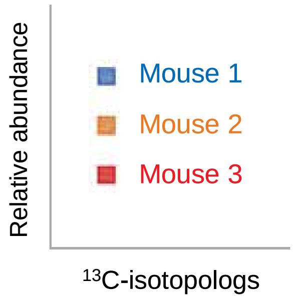


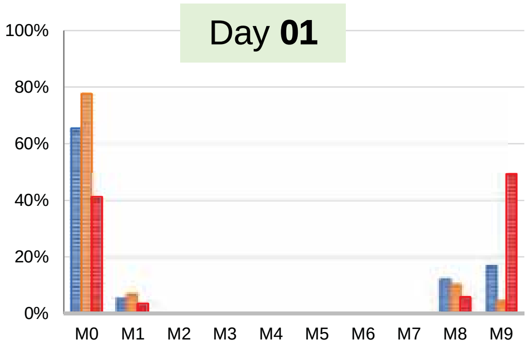


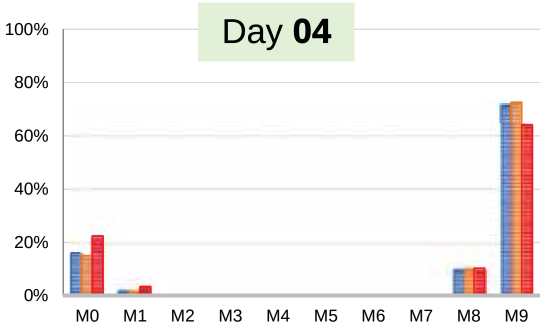


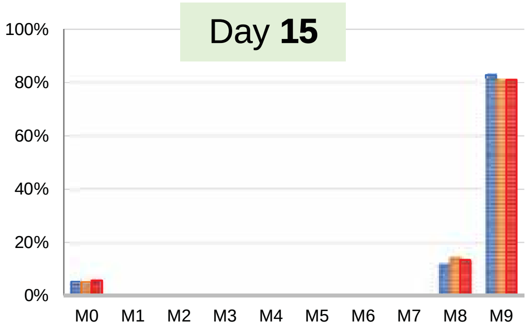


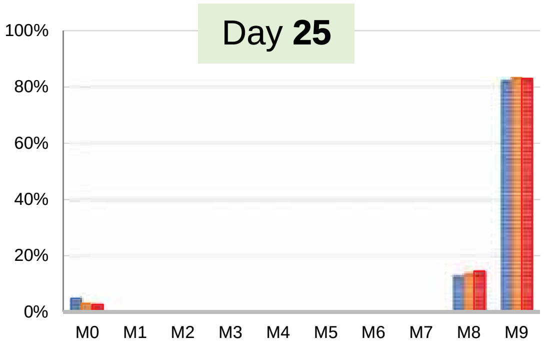


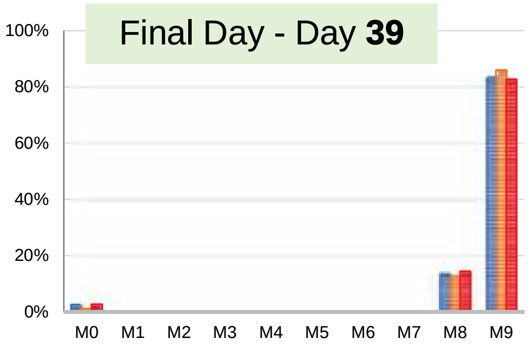


## Fractional $^{13}$C-enrichment

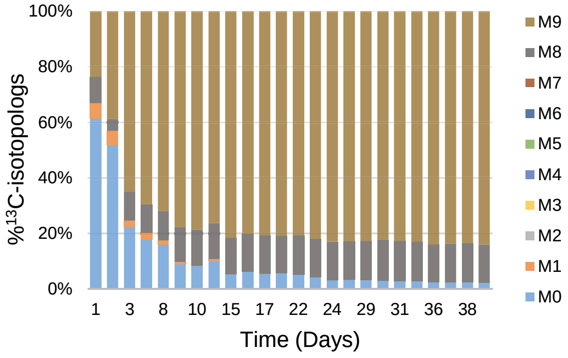


## $^{13}$C-enrichment kinetics

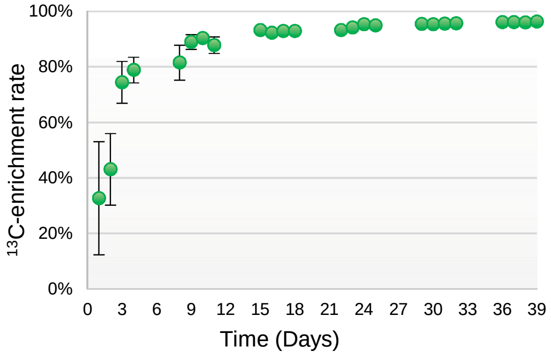

# Identified metabolites in murine urine

*Metabolites from the chemical library*

| Metabolite | Adenine |
|---|---|
| **Formula** | $C_5H_5N_5$ |
| **Exact mass** | 135.0545 |

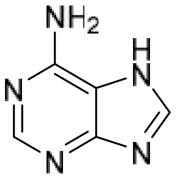


| Ion type | [M+H]+ |
|---|---|
| **m/z** | 136.0618 |

## Isotopic patterns

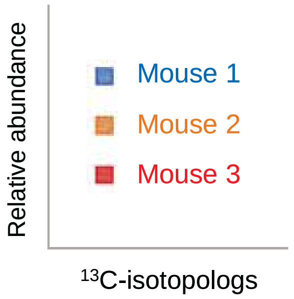


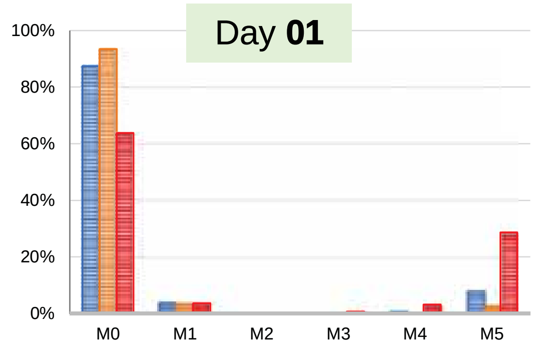


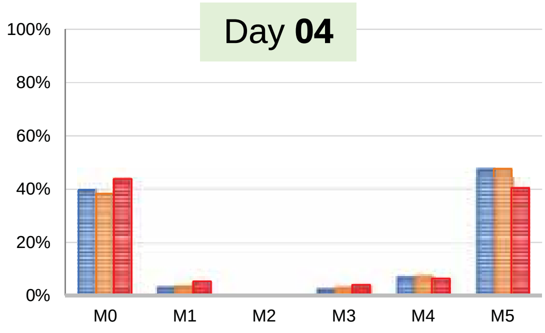


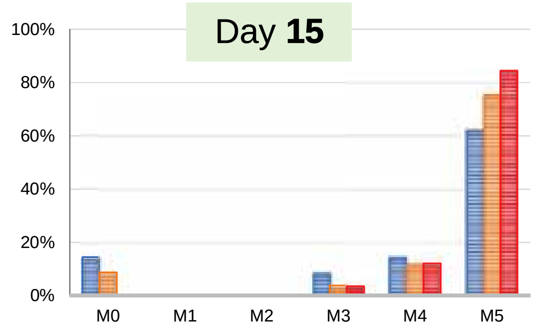


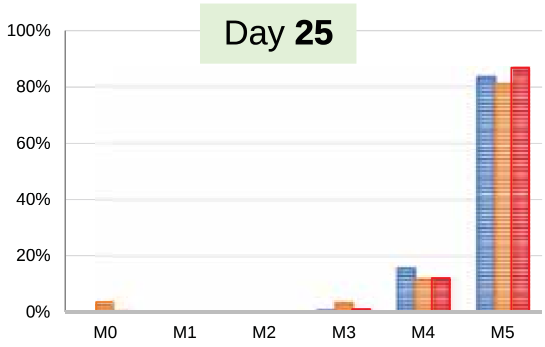


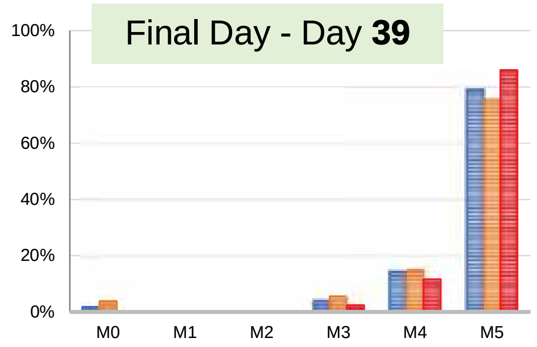


## Fractional $^{13}$C-enrichment

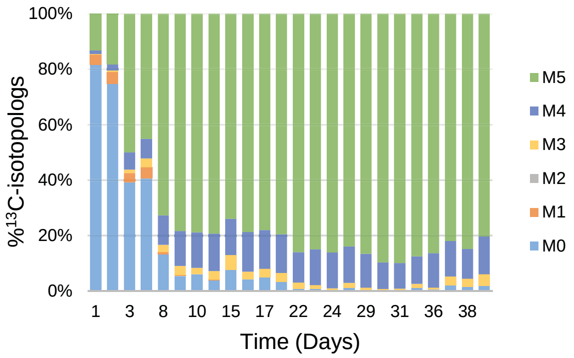


## $^{13}$C-enrichment kinetics

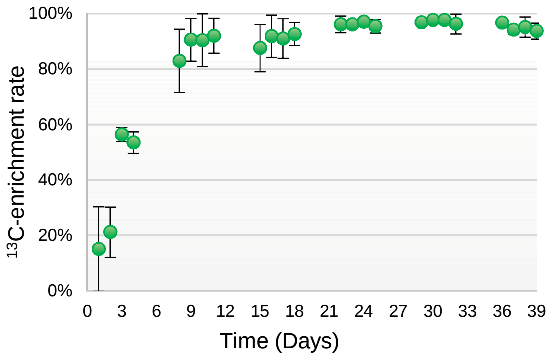

# Identified metabolites in murine urine

*Metabolites from the chemical library*

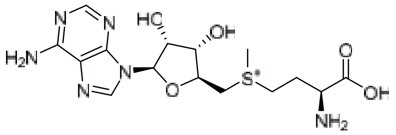

| Metabolite | S-adenosylmethionine |
|---|---|
| Formula | C15H22N6O5S |
| Exact mass | 398.1372 |

| Ion type | [M+H]+ |
|---|---|
| m/z | 399.1445 |

## Isotopic patterns

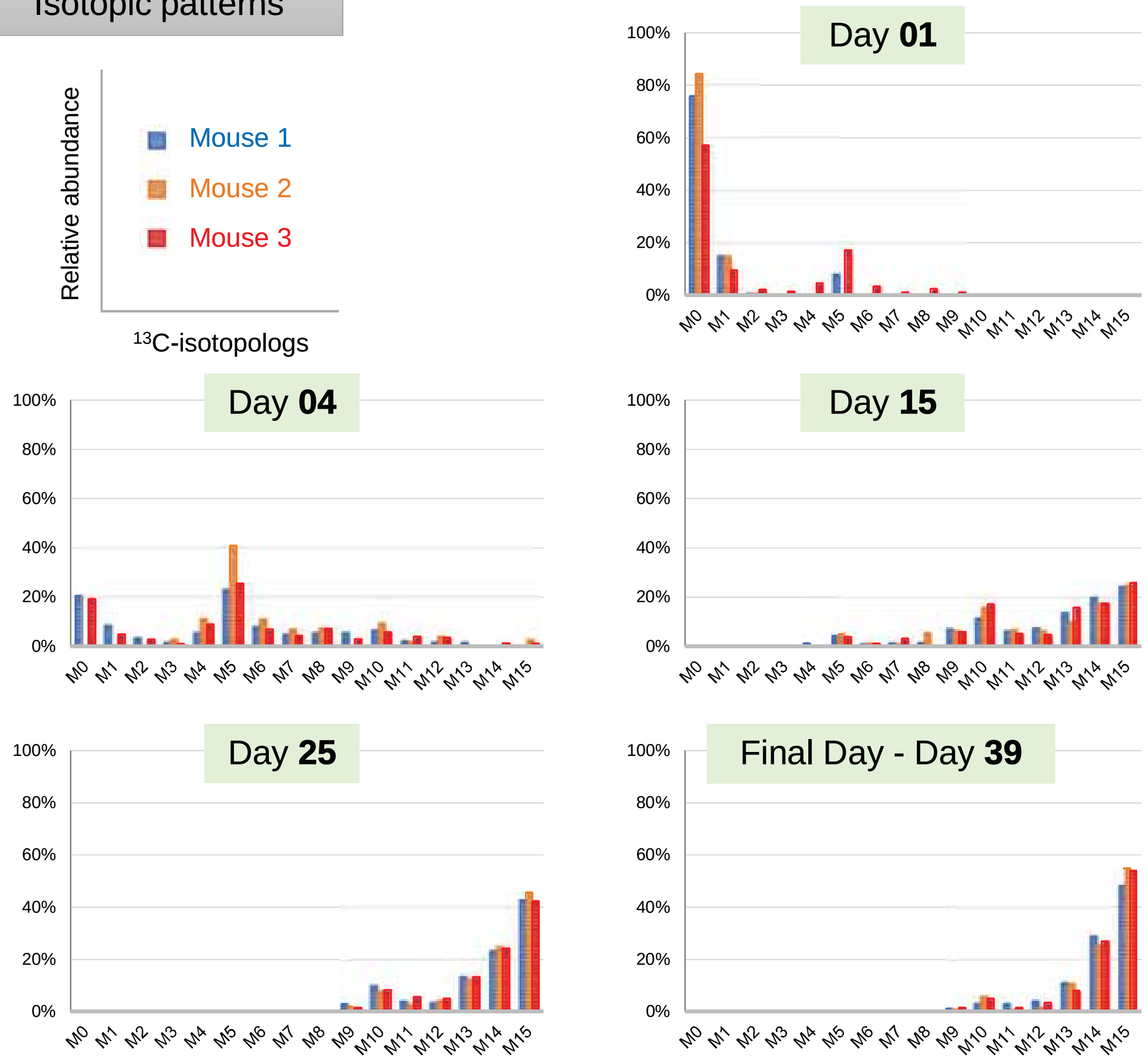

# Identified metabolites in murine urine

*Metabolites from the chemical library*

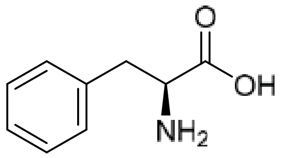


| Metabolite | Phenylalanine |
|---|---|
| **Formula** | C9H11NO2 |
| **Exact mass** | 165.079 |

| Ion type | [M+H]+ |
|---|---|
| **m/z** | 166.0863 |

## Isotopic patterns

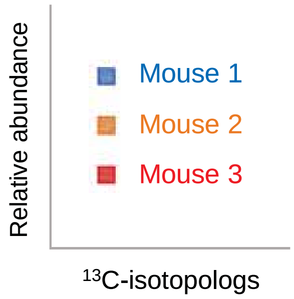


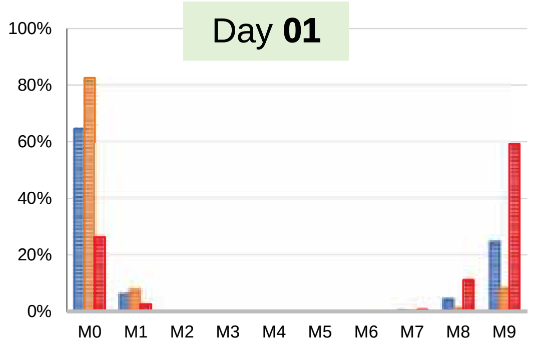


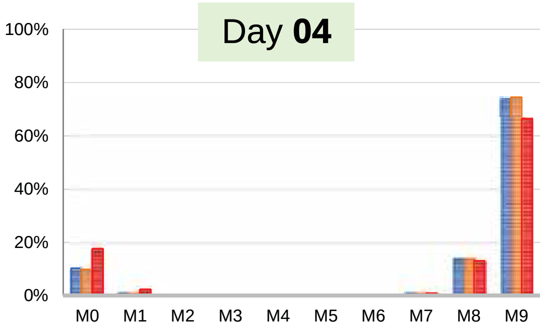


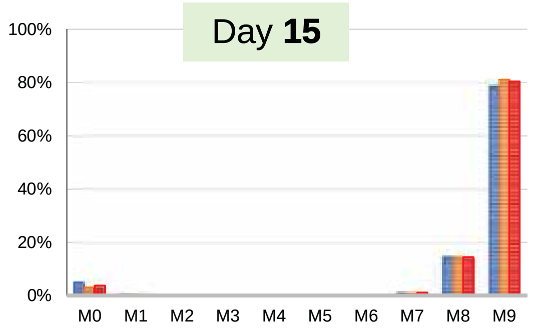


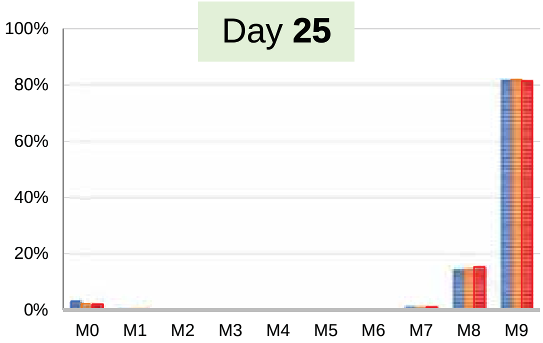


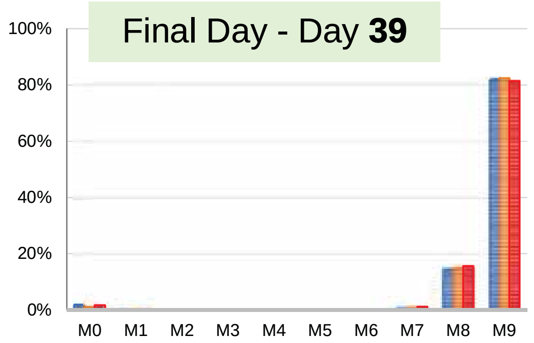


## Fractional $^{13}$C-enrichment

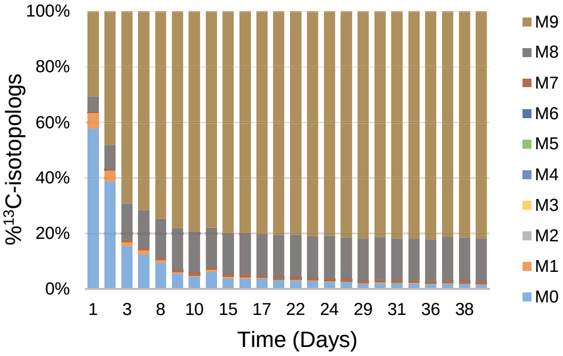


## $^{13}$C-enrichment kinetics

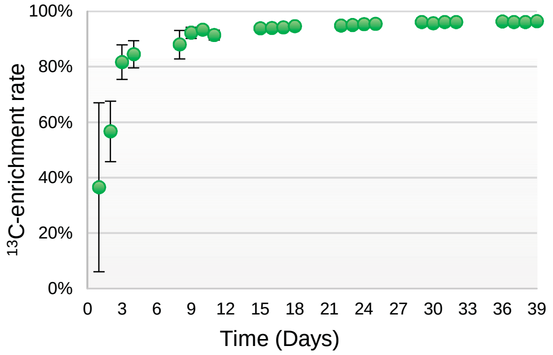

# Identified metabolites in murine urine

*Metabolites from the chemical library*

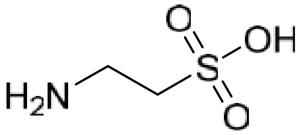


| Metabolite | Taurine |
|---|---|
| Formula | C2H7NO3S |
| Exact mass | 125.0147 |

| Ion type | [M-H]- |
|---|---|
| m/z | 124.0074 |

## Isotopic patterns

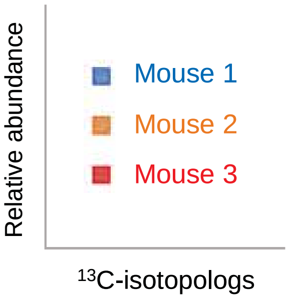


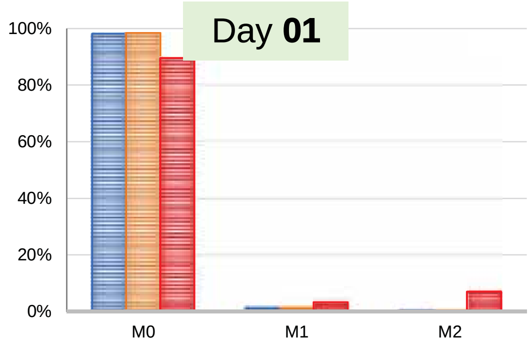


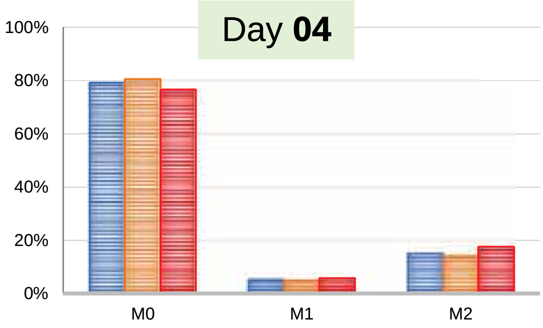


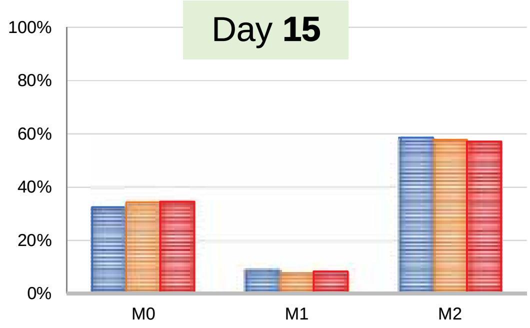


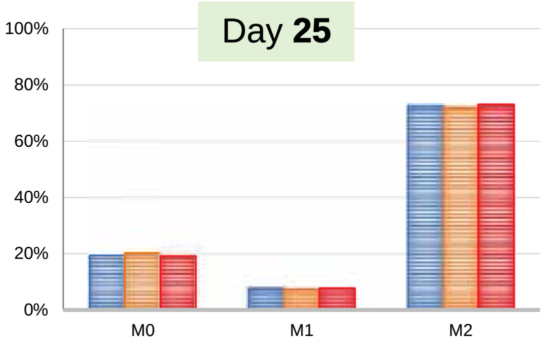


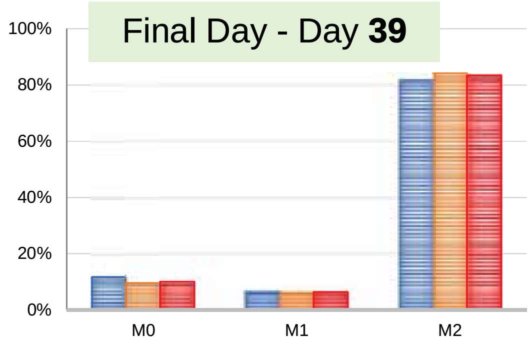


## Fractional $^{13}$C-enrichment

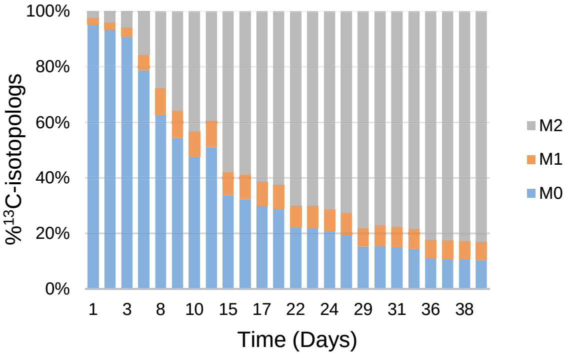


## $^{13}$C-enrichment kinetics

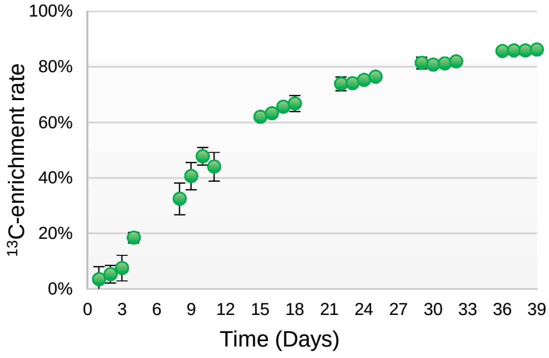

# Identified metabolites in murine urine

*Metabolites from the chemical library*

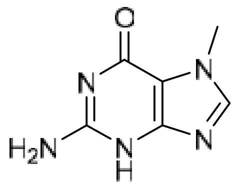


| Metabolite | 7-methylguanine |
| --- | --- |
| Formula | C6H7N5O |
| Exact mass | 165.0651 |

| Ion type | [M+H]+ |
| --- | --- |
| m/z | 166.0723 |

## Isotopic patterns

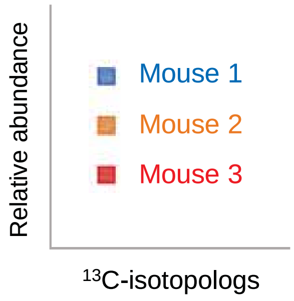


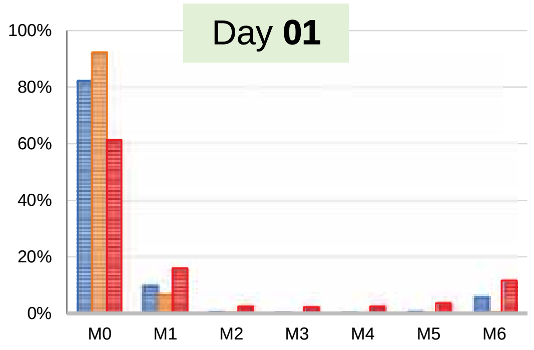


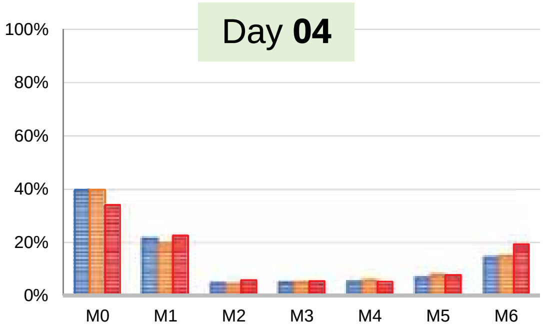


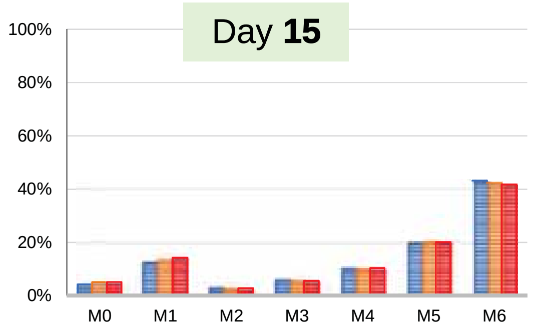


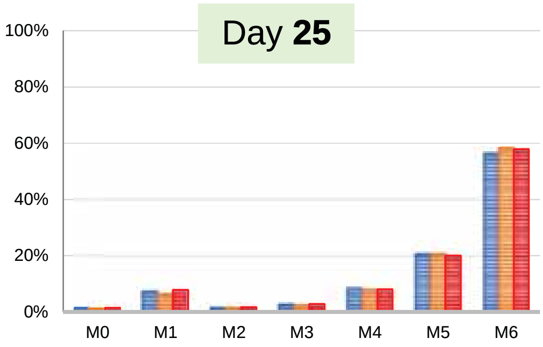


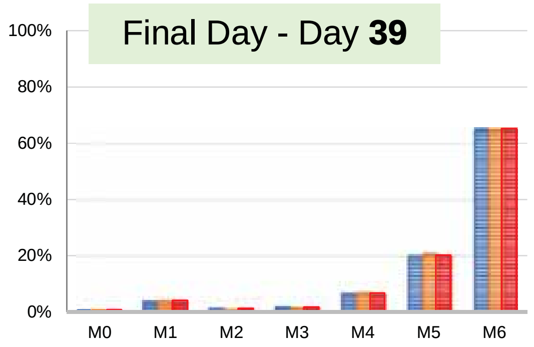


## Fractional $^{13}$C-enrichment

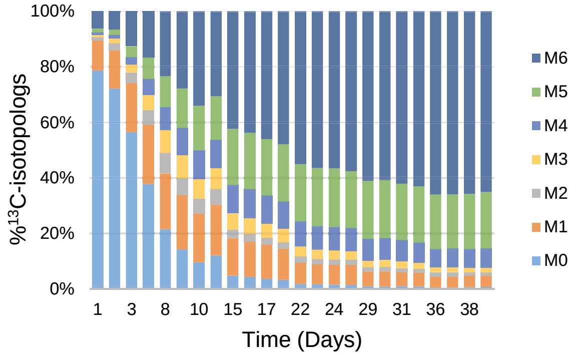


## $^{13}$C-enrichment kinetics

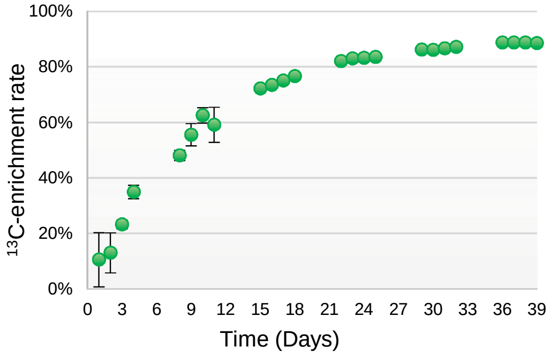

# Identified metabolites in murine urine

*Metabolites from the chemical library*

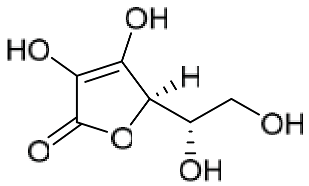


| Metabolite | Ascorbic acid |
|---|---|
| Formula | C6H8O6 |
| Exact mass | 176.0321 |

| Ion type | [M-H]- |
|---|---|
| m/z | 175.0248 |

## Isotopic patterns

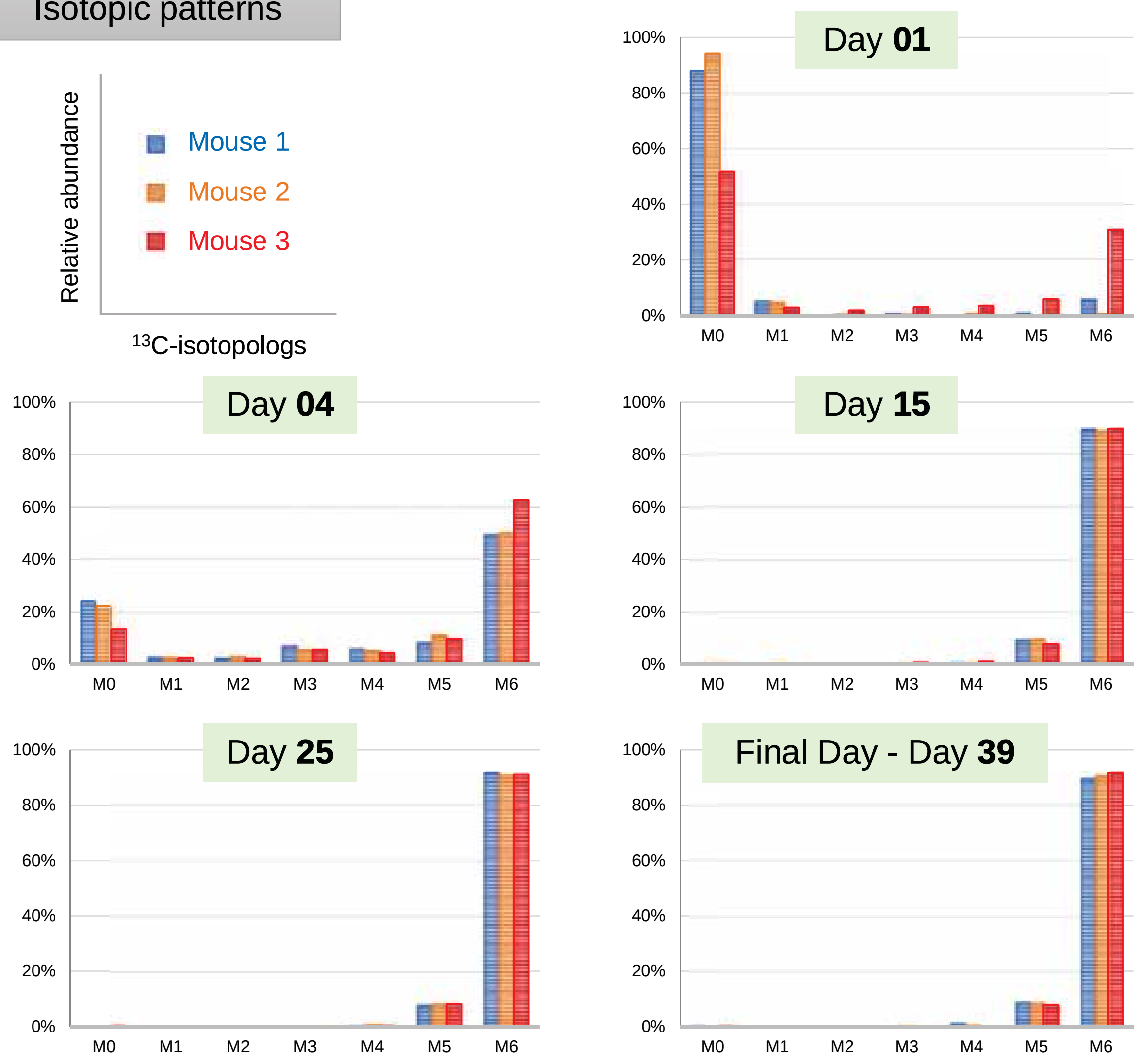

# Identified metabolites in murine urine

*Metabolites from the chemical library*

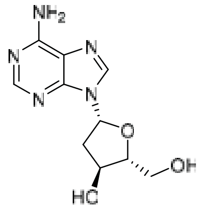


| Metabolite | Deoxyadenosine |
|---|---|
| **Formula** | C10H13N5O3 |
| **Exact mass** | 251.1018 |

| Ion type | [M+H]+ |
|---|---|
| **m/z** | 252.1091 |

## Isotopic patterns

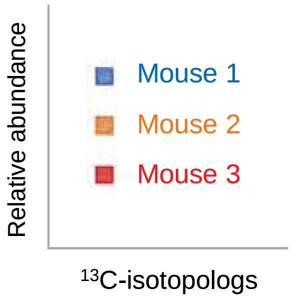


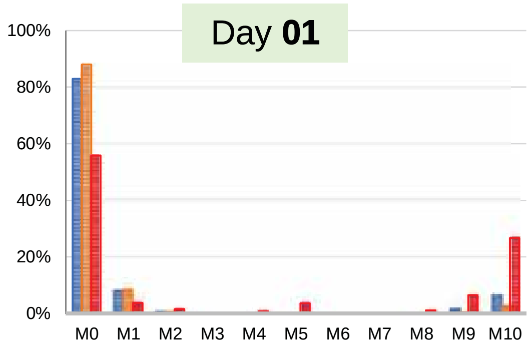


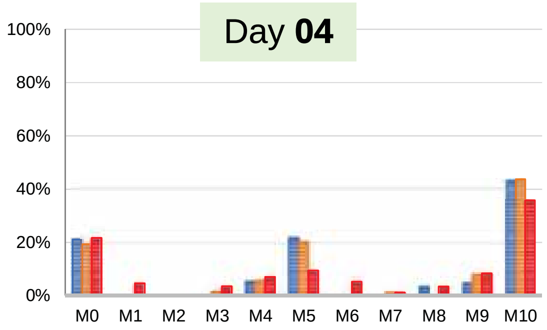


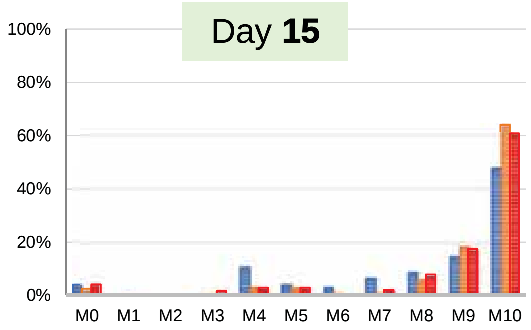


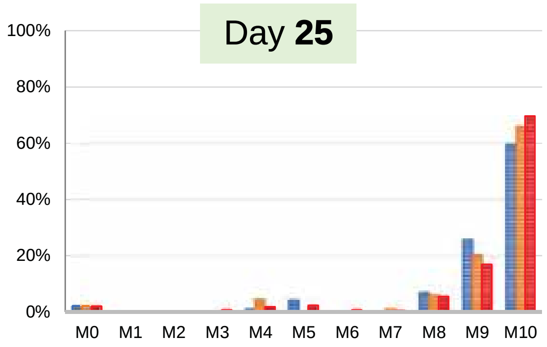


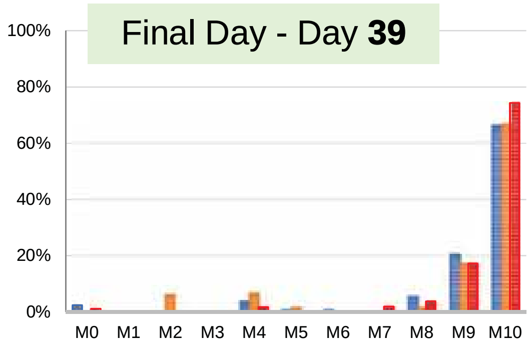

# Identified metabolites in murine urine

*Metabolites from the chemical library*

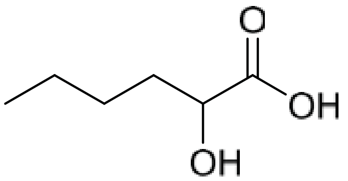


| Metabolite | 2-hydroxycaproic acid |
|---|---|
| Formula | C6H12O3 |
| Exact mass | 132.0786 |

| Ion type | [M-H]- |
|---|---|
| m/z | 131.0713 |

## Isotopic patterns

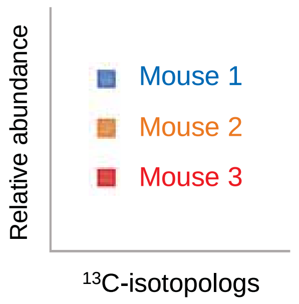


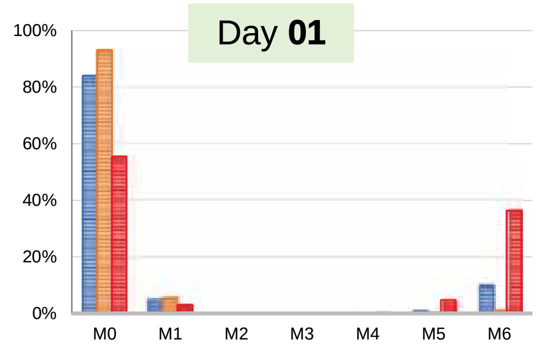


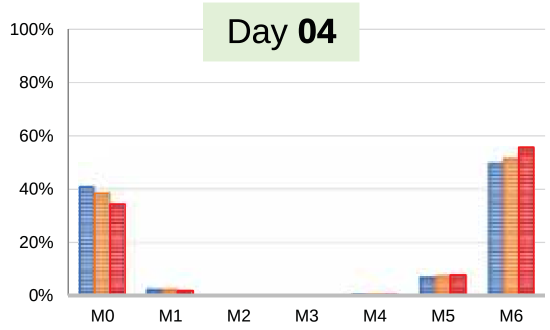


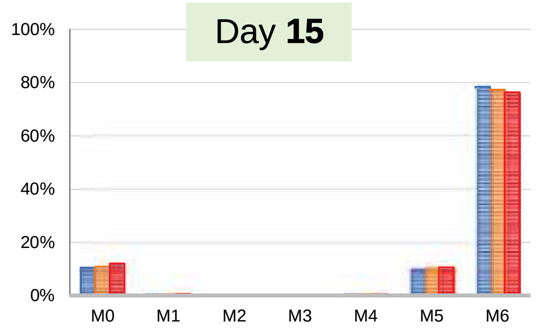


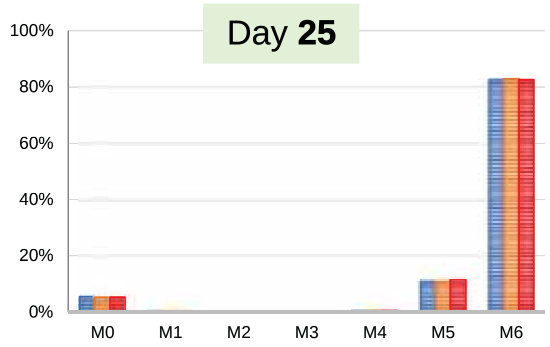


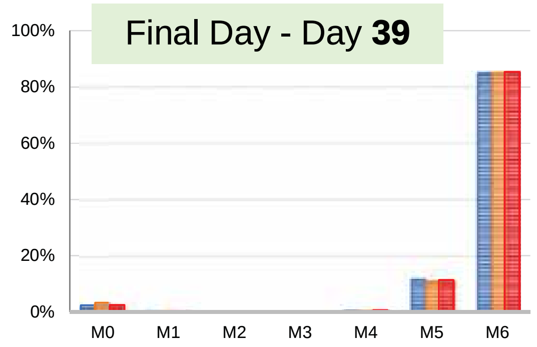


## Fractional $^{13}$C-enrichment

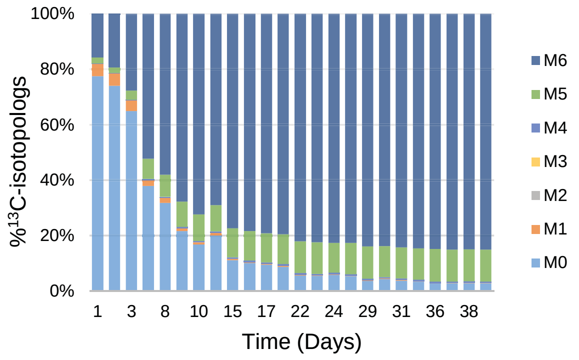


## $^{13}$C-enrichment kinetics

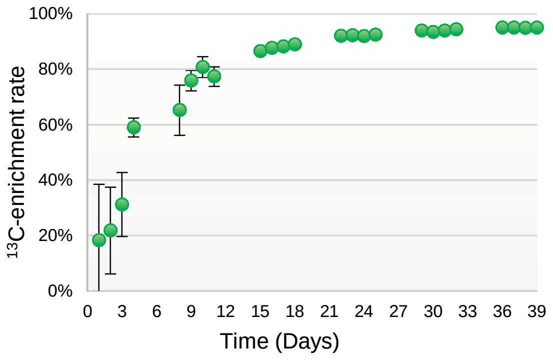

# Identified metabolites in murine urine

*Metabolites from the chemical library*

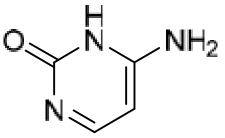


| Metabolite | Cytosine |
|---|---|
| Formula | C4H5N3O |
| Exact mass | 111.0433 |

| Ion type | [M+H]+ |
|---|---|
| m/z | 112.0505 |

## Isotopic patterns

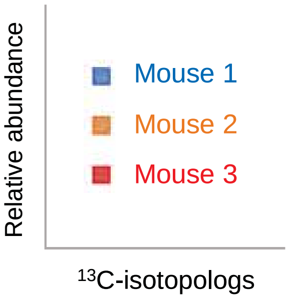


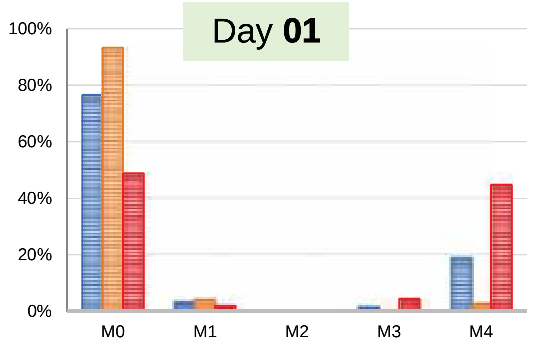


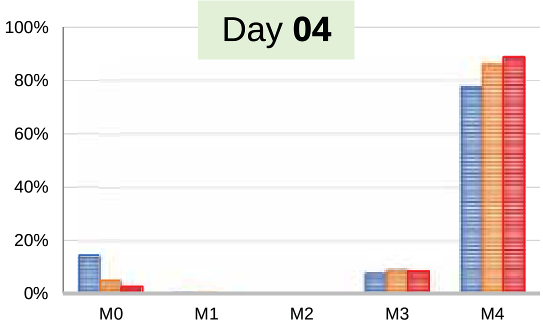


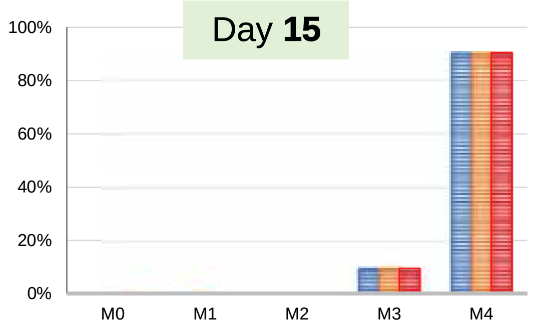


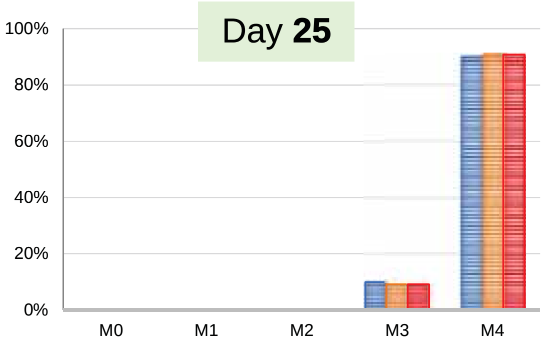


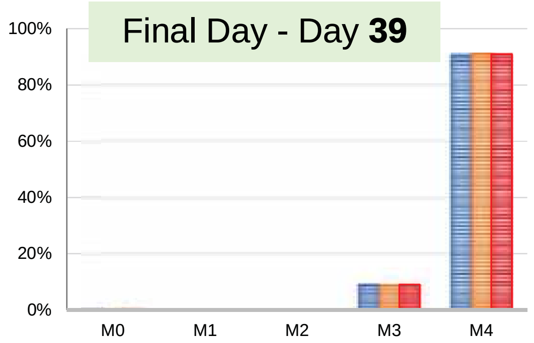


## Fractional $^{13}$C-enrichment

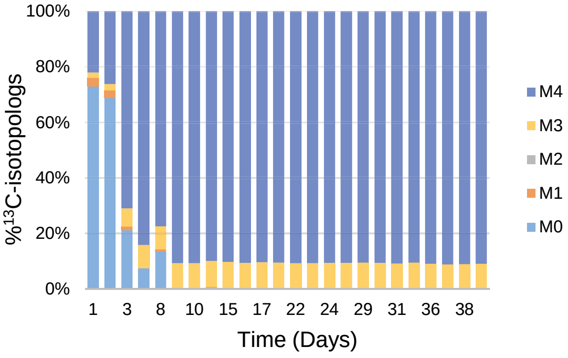


## $^{13}$C-enrichment kinetics

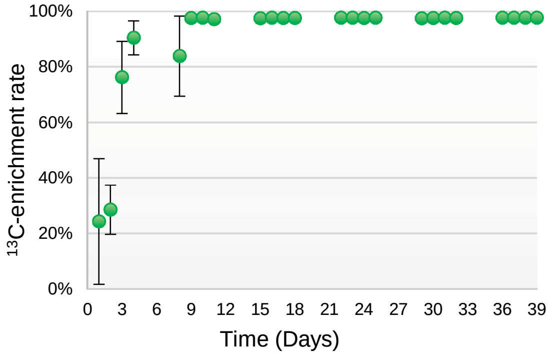

# Identified metabolites in murine urine

*Metabolites from the chemical library*

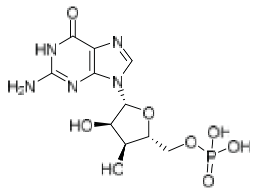


| Metabolite | GMP |
|---|---|
| Formula | C10H14N5O8P |
| Exact mass | 363.058 |

| Ion type | [M-H]- |
|---|---|
| m/z | 362.0507 |

## Isotopic patterns

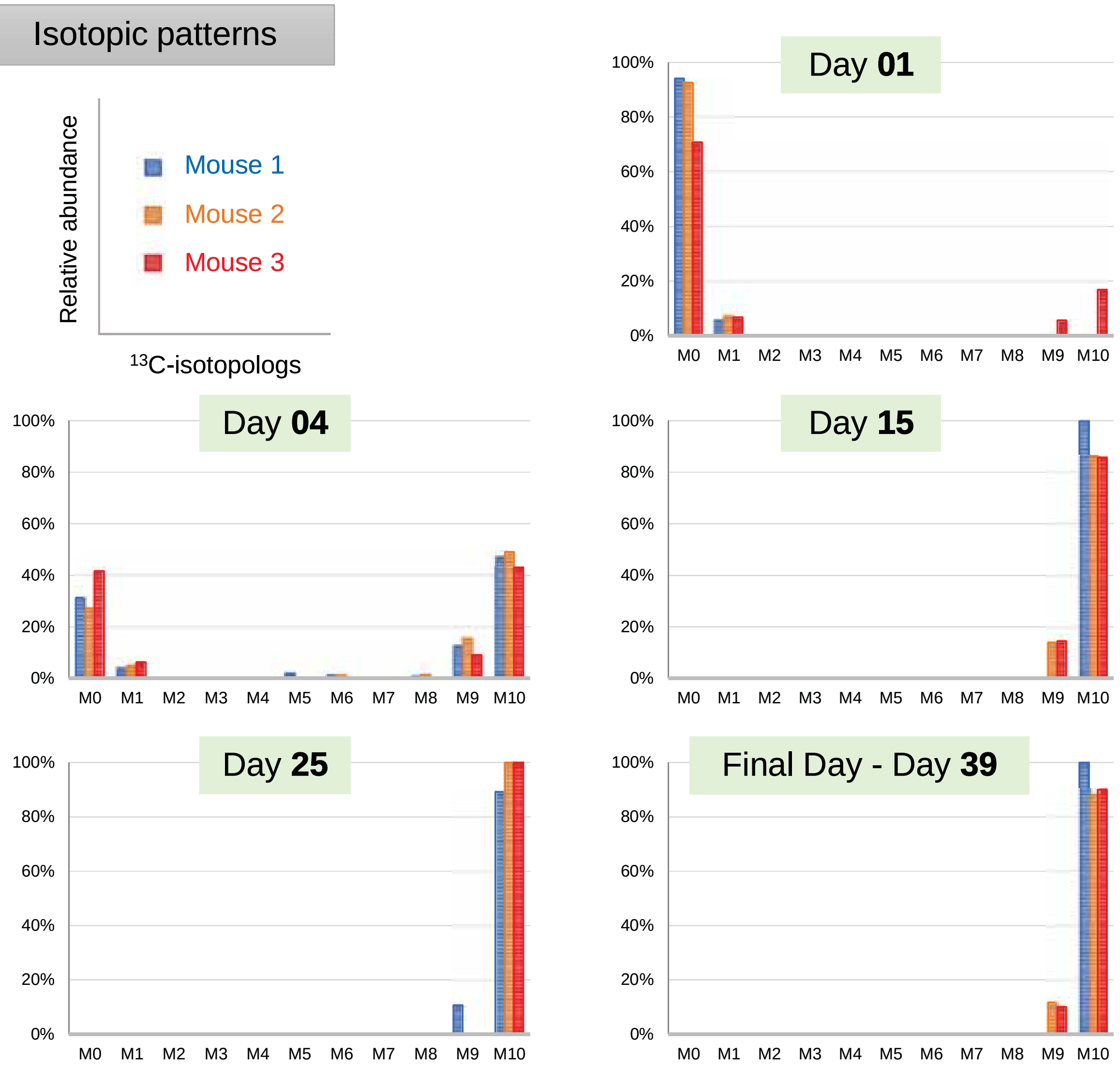

# Identified metabolites in murine urine

*Metabolites from the chemical library*

| Metabolite | 4-Hydroxybenzoic acid |
|---|---|
| Formula | C7H6O3 |
| Exact mass | 138.0317 |

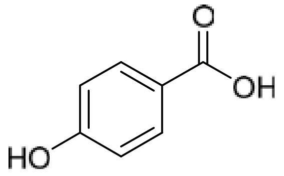


| Ion type | [M-H]- |
|---|---|
| m/z | 137.0244 |

## Isotopic patterns

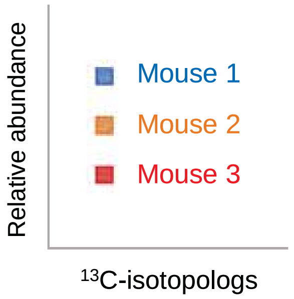


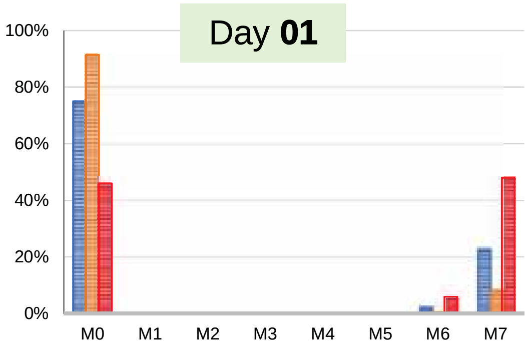


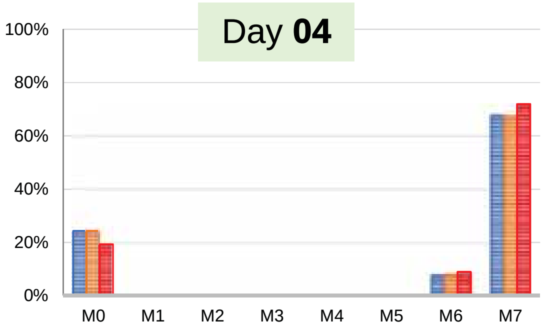


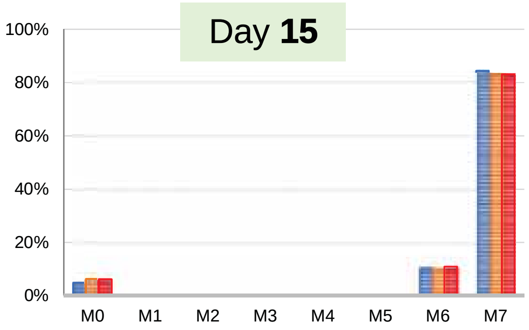


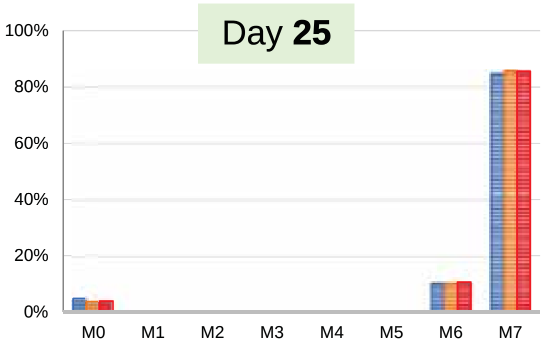


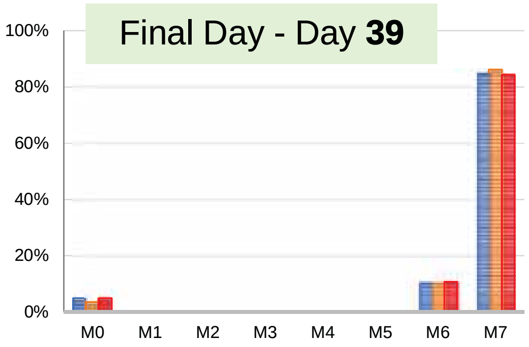


## Fractional $^{13}$C-enrichment

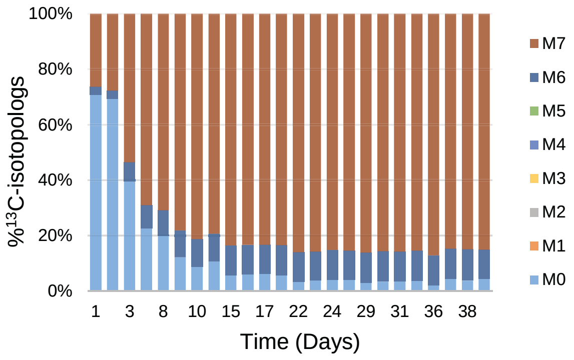


## $^{13}$C-enrichment kinetics

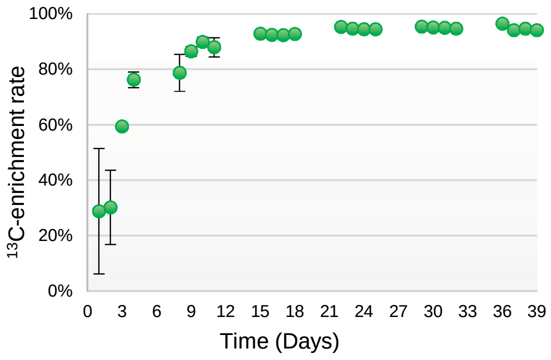

# Identified metabolites in murine urine

*Metabolites from the chemical library*

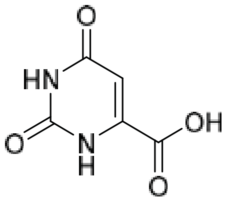


| Metabolite | Orotic acid |
|---|---|
| Formula | C5H4N2O4 |
| Exact mass | 156.0171 |

| Ion type | [M-H]- |
|---|---|
| m/z | 155.0098 |

## Isotopic patterns

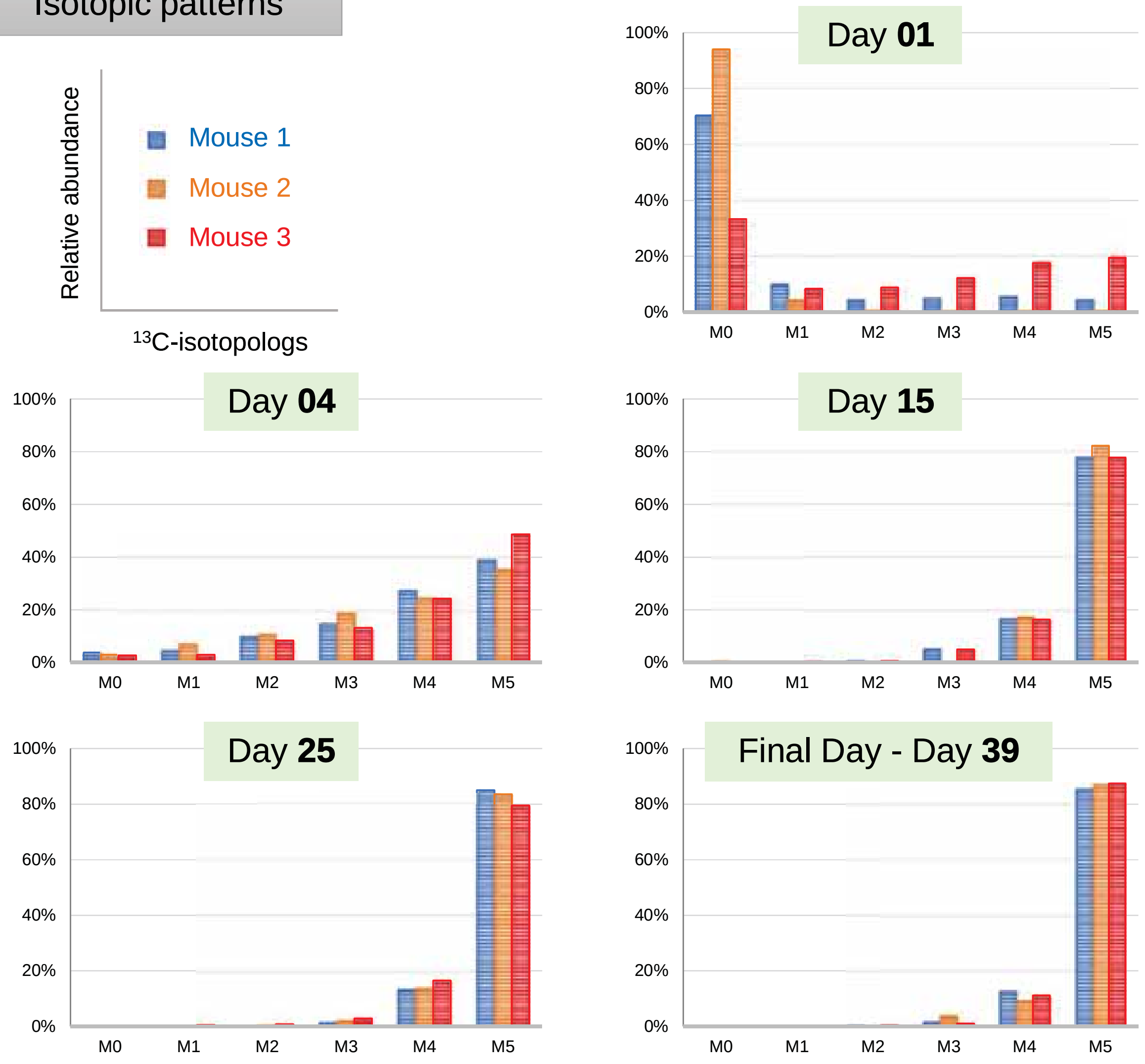

# Identified metabolites in murine urine

*Metabolites from the chemical library*

| Metabolite | Imidazoleacetic acid |
|---|---|
| Formula | C5H6N2O2 |
| Exact mass | 126.0429 |

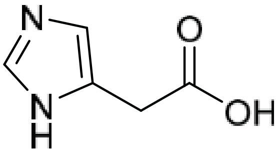

| Ion type | [M+H]+ |
|---|---|
| m/z | 127.0502 |

## Isotopic patterns

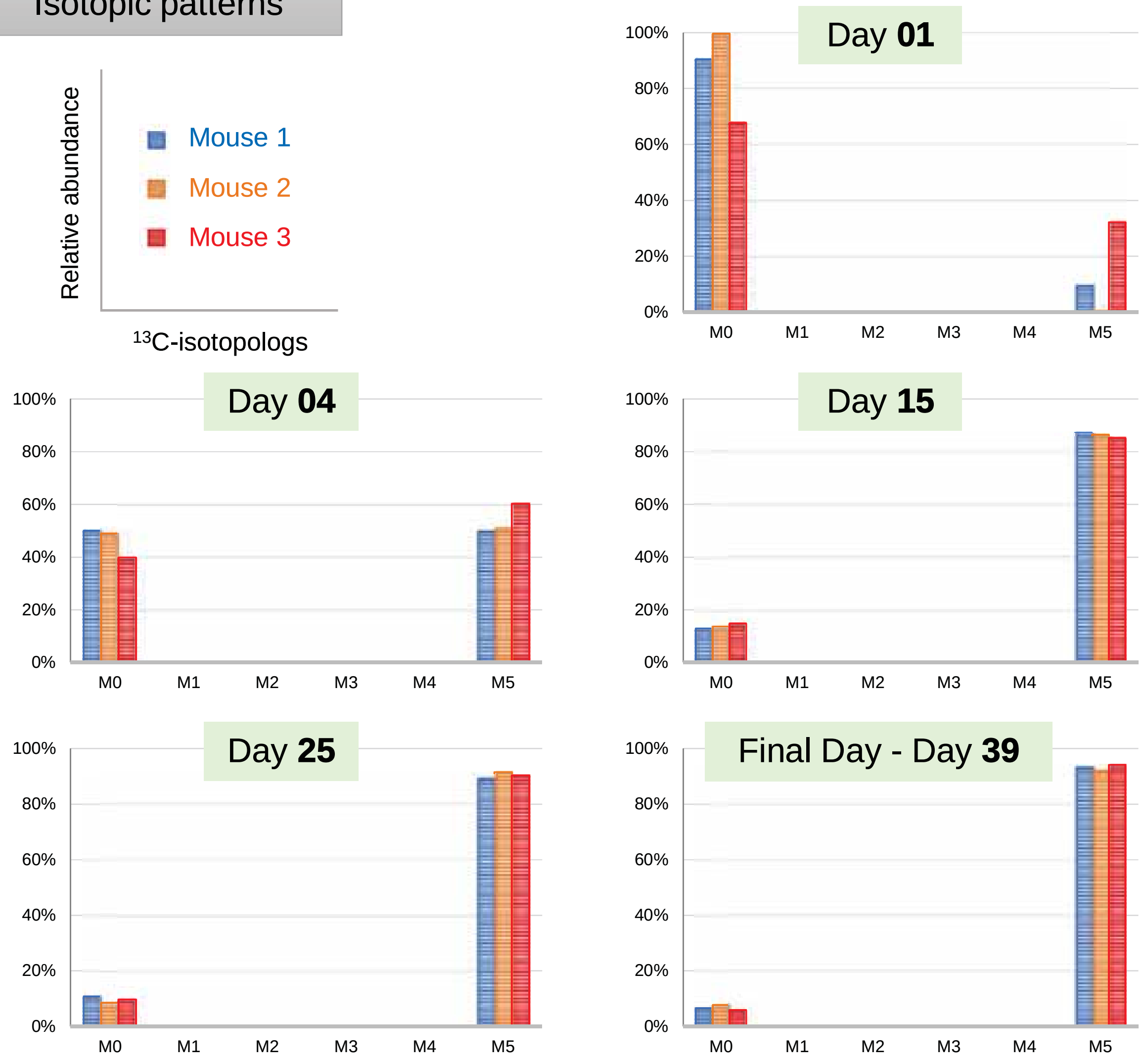


## Fractional $^{13}$C-enrichment

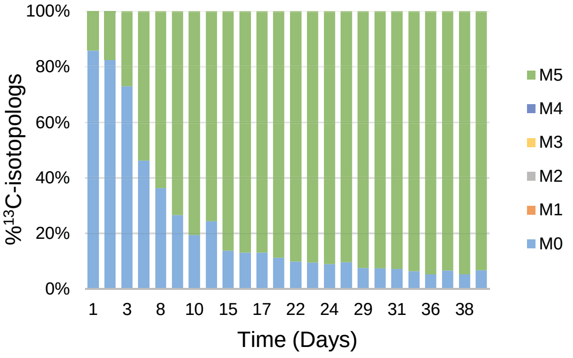


## $^{13}$C-enrichment kinetics

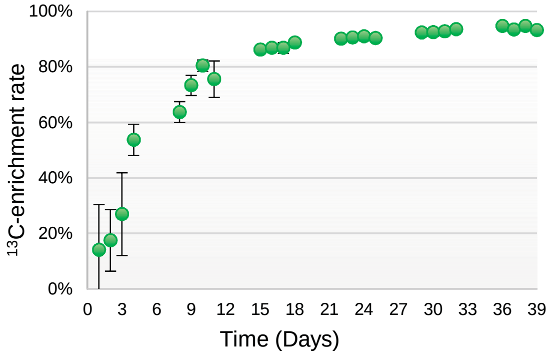

# Identified metabolites in murine urine

*Metabolites from the chemical library*

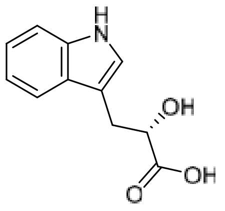


| Metabolite | Indolelactic acid |
|---|---|
| Formula | C11H11NO3 |
| Exact mass | 205.0739 |

| Ion type | [M-H]- |
|---|---|
| m/z | 204.0666 |

## Isotopic patterns

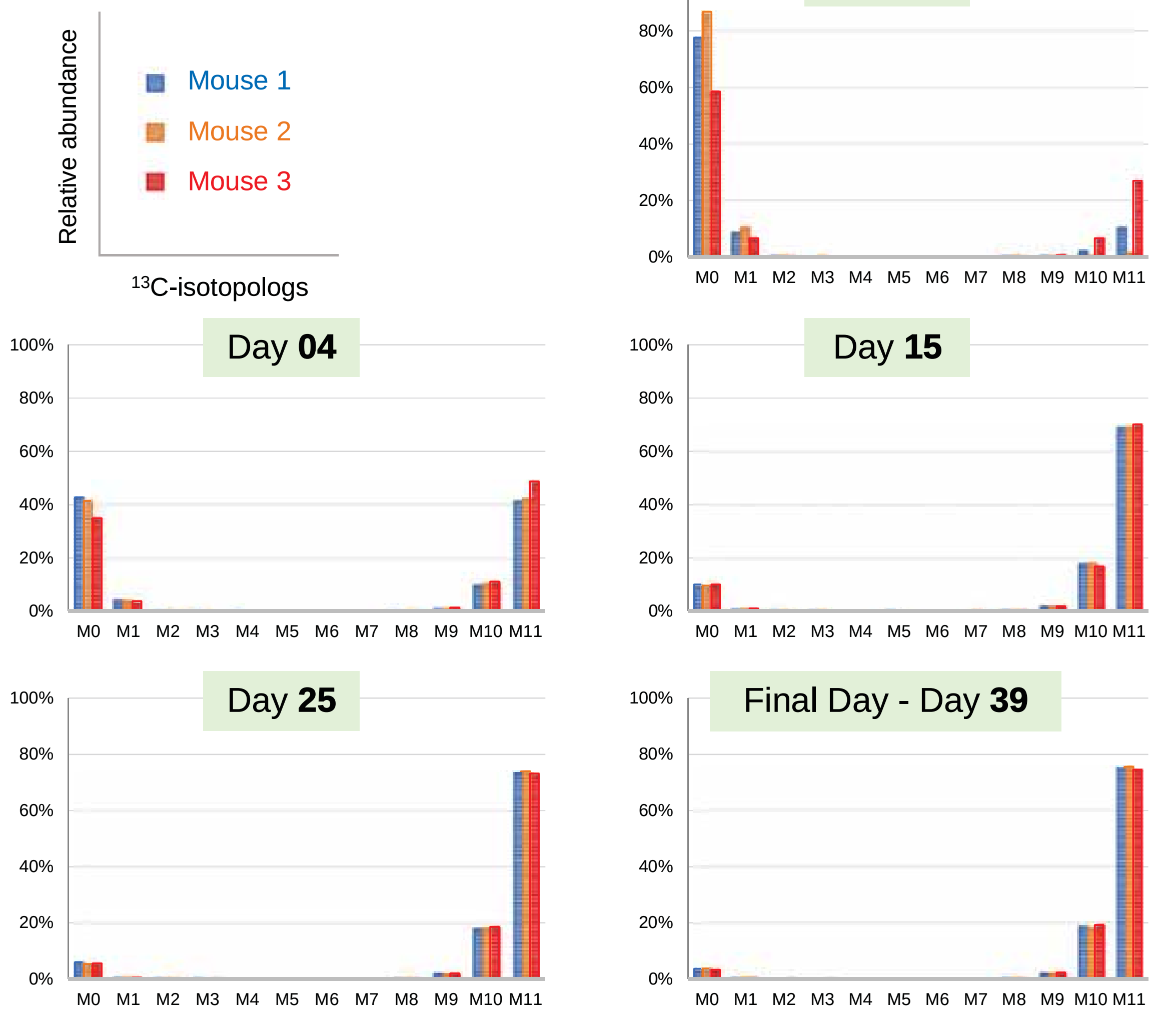

# Identified metabolites in murine urine

*Metabolites from the chemical library*

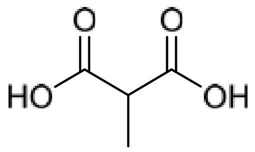

| **Metabolite** | **Methylmalonic acid** |
|---|---|
| **Formula** | C4H6O4 |
| **Exact mass** | 118.0266 |

| **Ion type** | [M-H]- |
|---|---|
| **m/z** | 117.0193 |

## Isotopic patterns

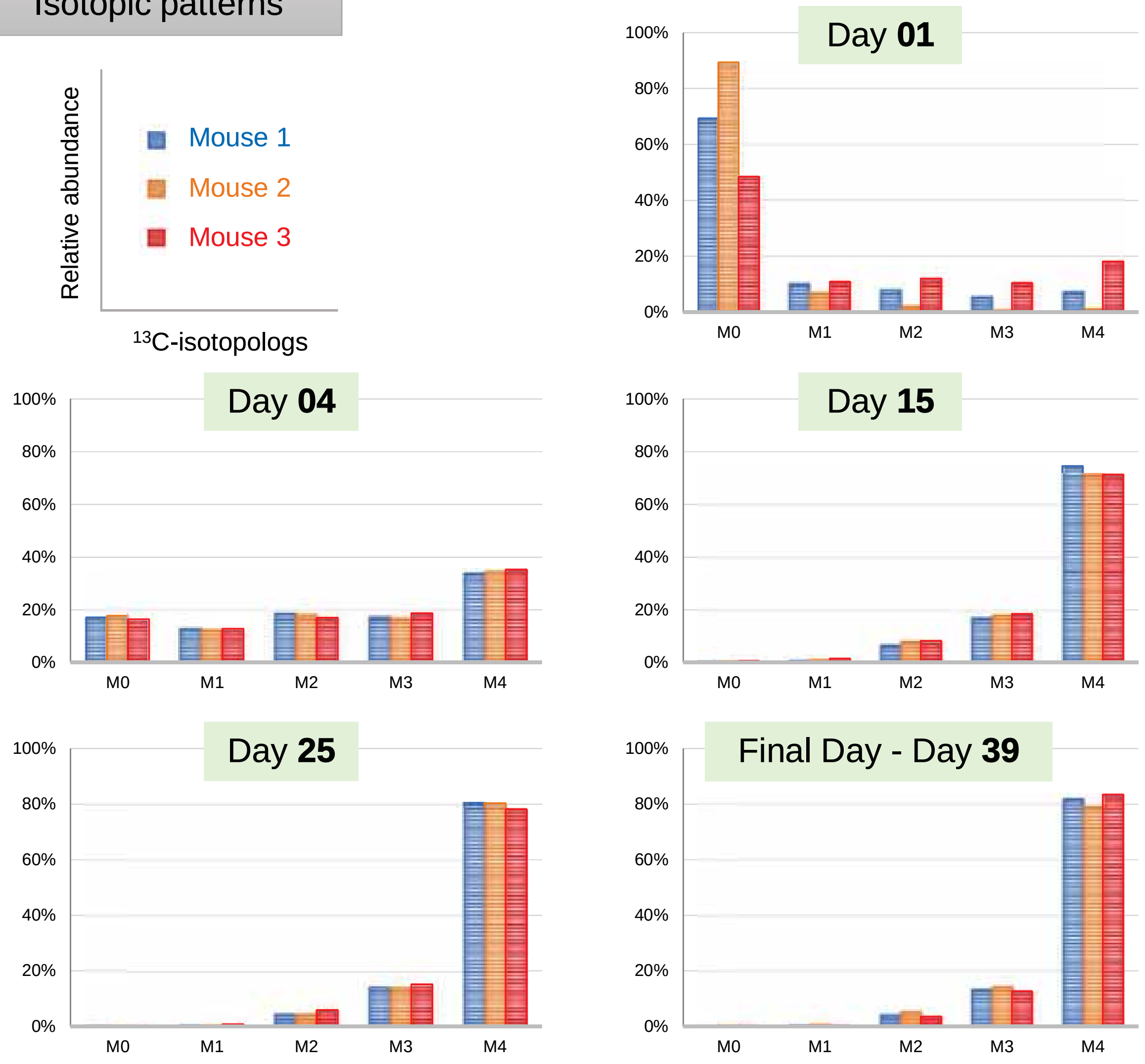


## Fractional 13C-enrichment

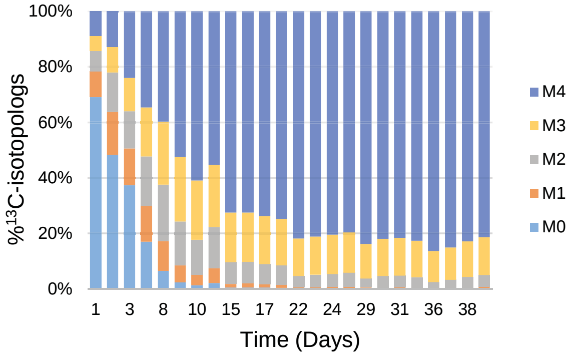


## 13C-enrichment kinetics

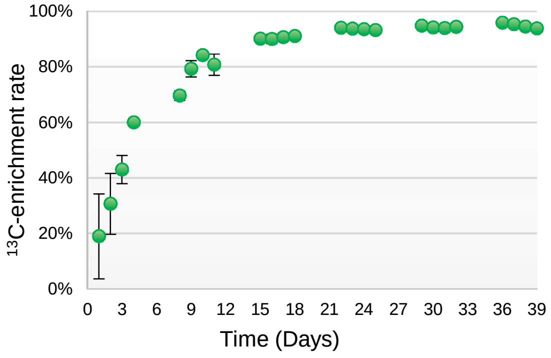

# Identified metabolites in murine urine

*Metabolites from the chemical library*

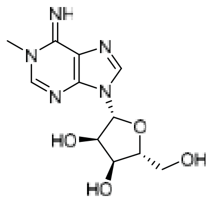


| Metabolite | 1-Methyladenosine |
|---|---|
| Formula | C11H15N5O4 |
| Exact mass | 281.1124 |

| Ion type | [M+H]+ |
|---|---|
| m/z | 282.1197 |

## Isotopic patterns

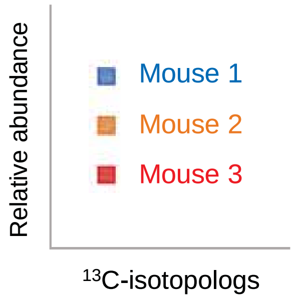


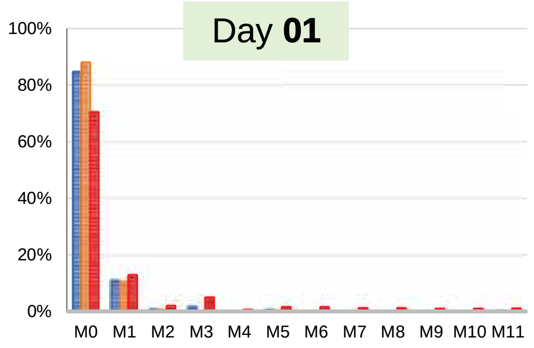


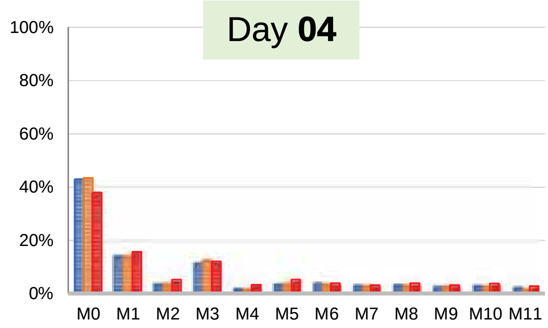


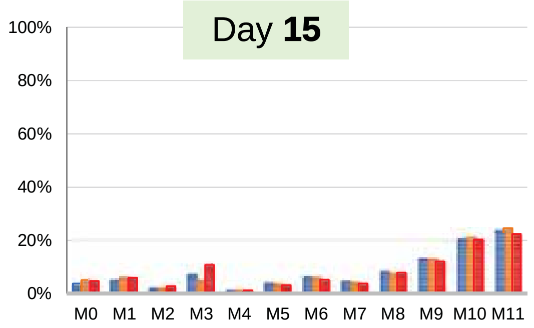


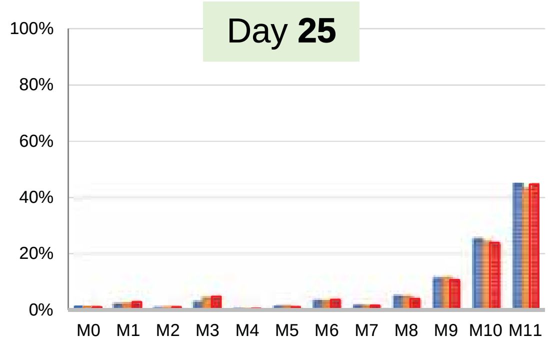


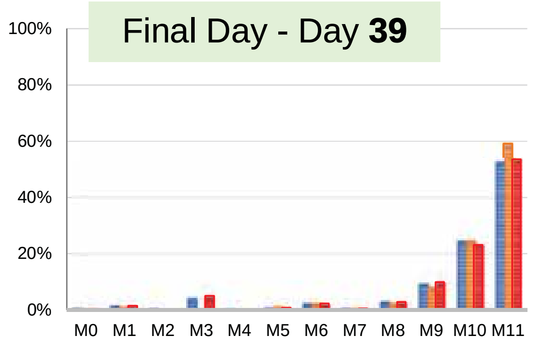


## Fractional $^{13}$C-enrichment

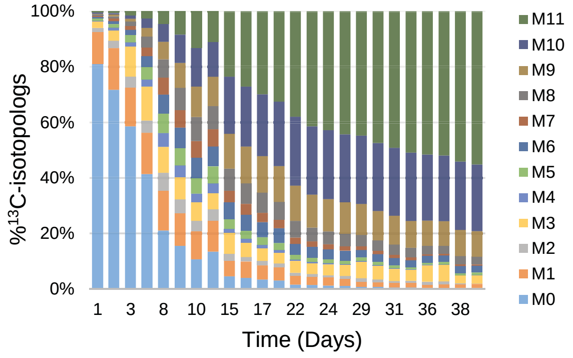


## $^{13}$C-enrichment kinetics

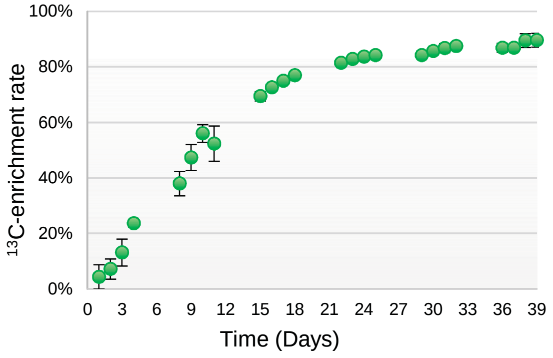

# Identified metabolites in murine urine

*Metabolites from the chemical library*

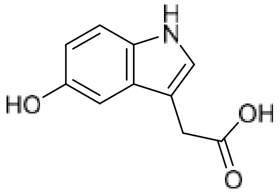


| **Metabolite** | **5-Hydroxyindoleacetic acid** |
|---|---|
| **Formula** | C10H9NO3 |
| **Exact mass** | 191.0582 |

| **Ion type** | [M-H]- |
|---|---|
| **m/z** | 190.0509 |

## Isotopic patterns

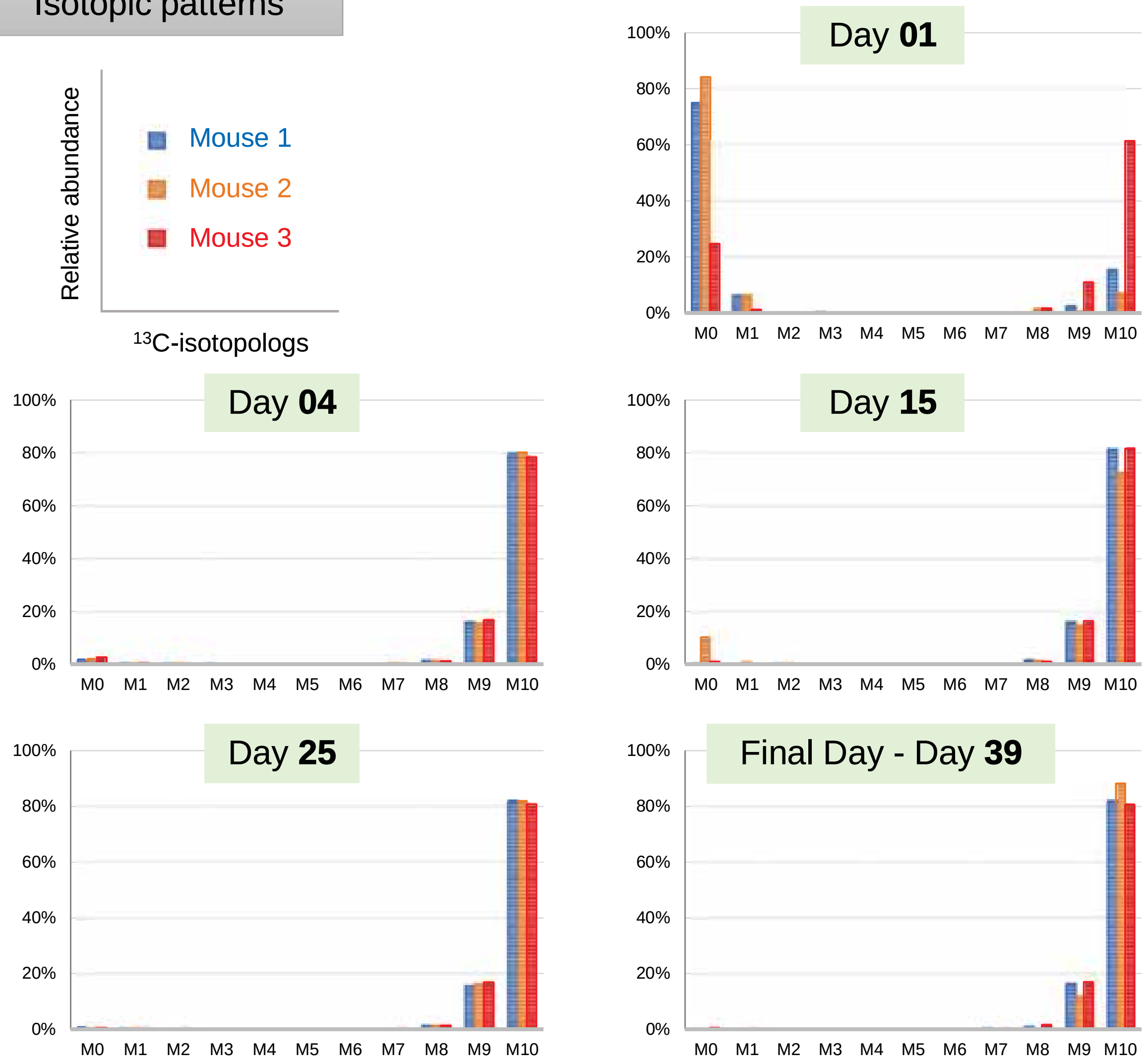

# Identified metabolites in murine urine

*Metabolites from the chemical library*

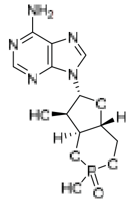

| Metabolite | Cyclic-AMP |
|---|---|
| Formula | C10H12N5O6P |
| Exact mass | 329.0525 |

| Ion type | [M+H]+ |
|---|---|
| m/z | 330.0598 |

## Isotopic patterns

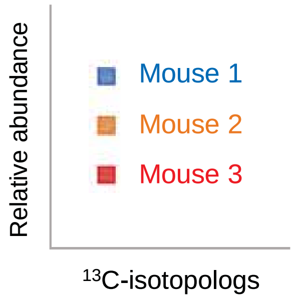


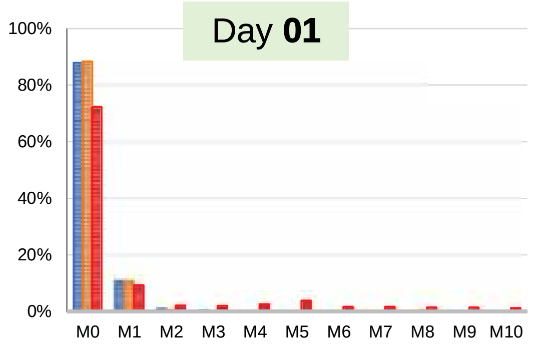


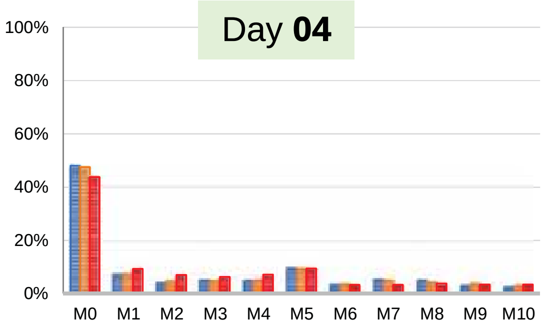


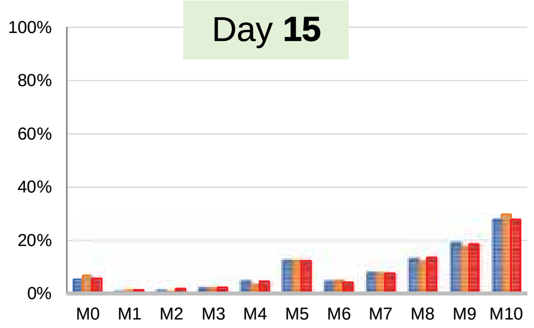


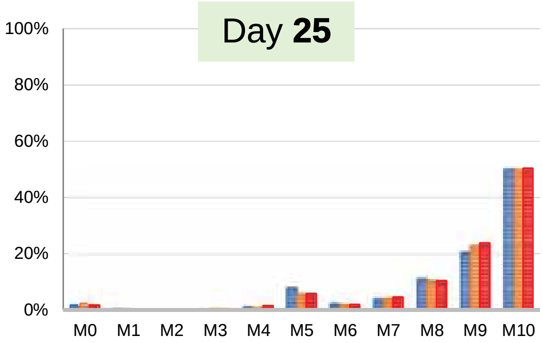


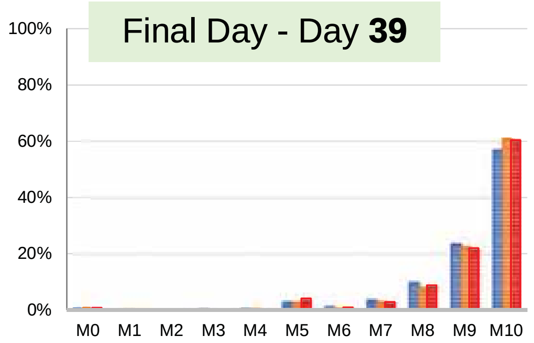


## Fractional $^{13}$C-enrichment

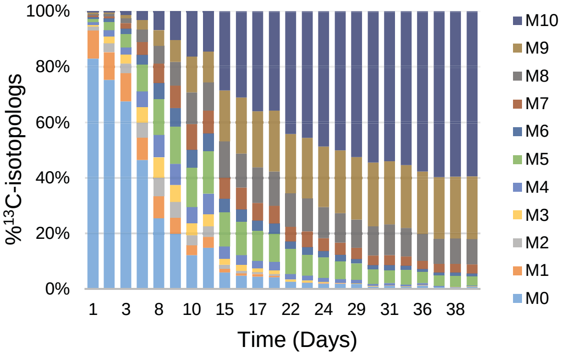


## $^{13}$C-enrichment kinetics

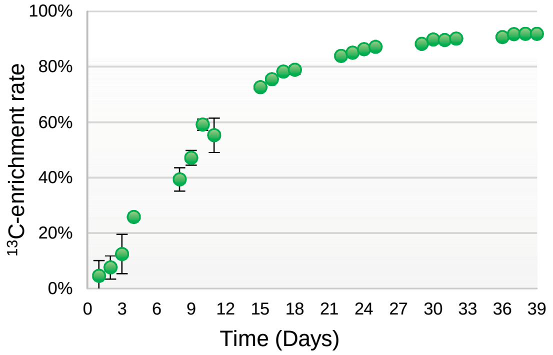

# Identified metabolites in murine urine

*Metabolites from the chemical library*

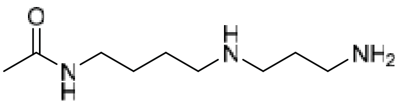


| Metabolite | N8-Acetylspermidine |
|---|---|
| Formula | C9H21N3O |
| Exact mass | 187.1685 |

| Ion type | [M+H]+ |
|---|---|
| m/z | 188.1757 |

## Isotopic patterns

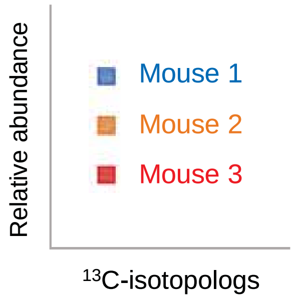


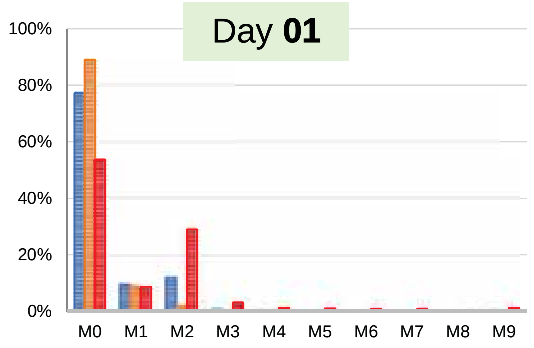


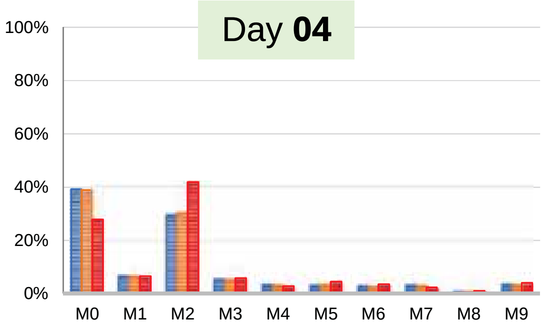


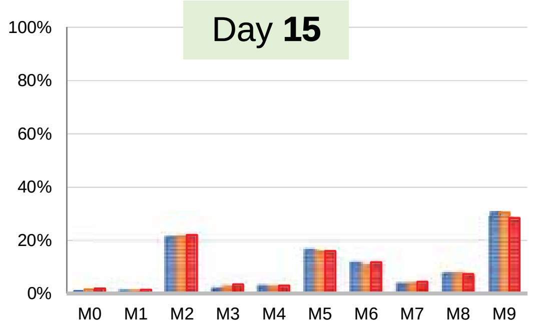


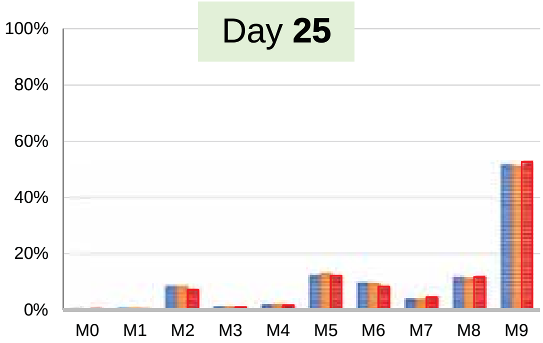


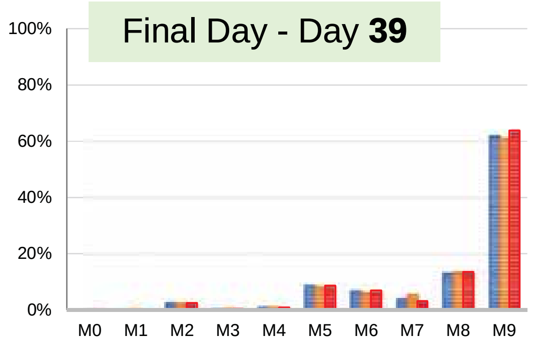


## Fractional $^{13}$C-enrichment

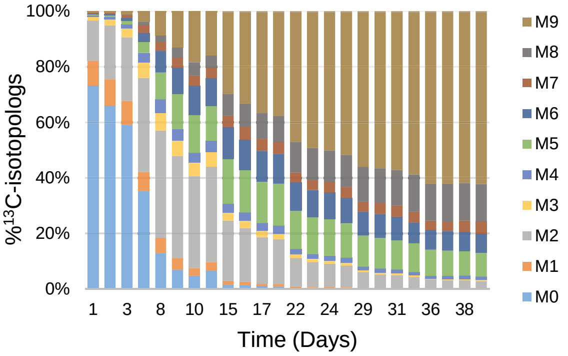


## $^{13}$C-enrichment kinetics

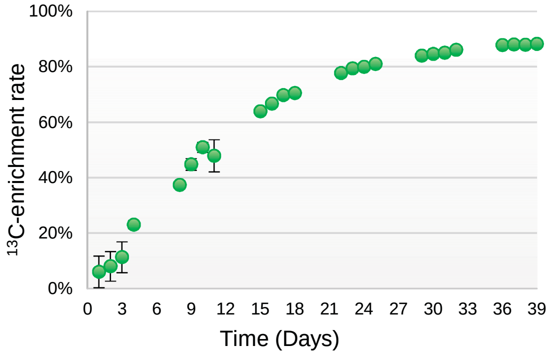

# Identified metabolites in murine urine

*Metabolites from the chemical library*

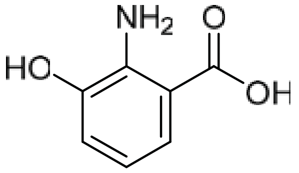


| Metabolite | 3-Hydroxyanthranilic acid |
|---|---|
| **Formula** | C7H7NO3 |
| **Exact mass** | 153.0426 |

| Ion type | [M-H]- |
|---|---|
| **m/z** | 152.0353 |

## Isotopic patterns

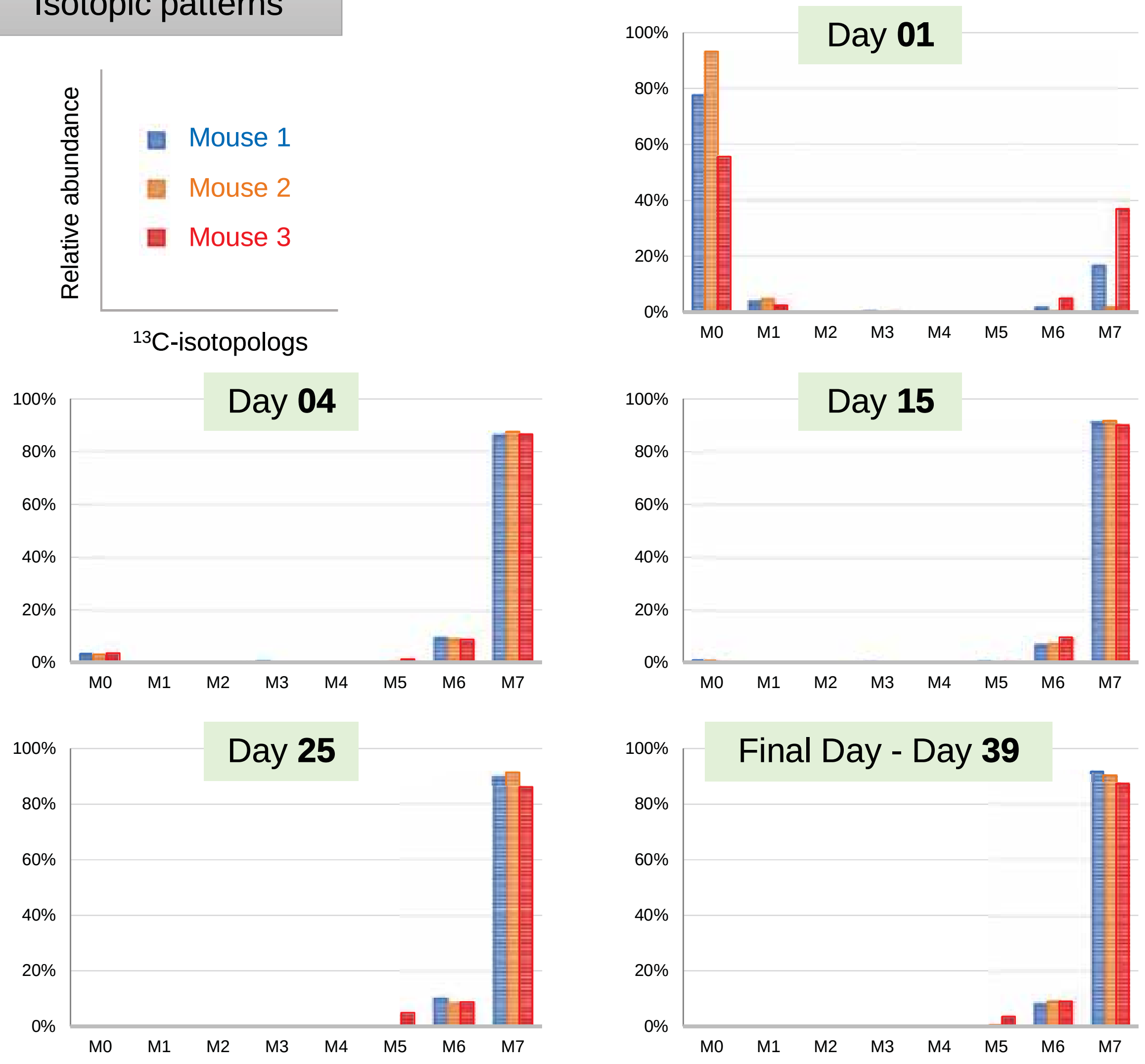

# Identified metabolites in murine urine

*Metabolites from the chemical library*

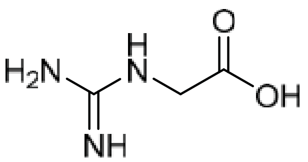


| **Metabolite** | **Guanidineacetic acid** |
|---|---|
| **Formula** | C3H7N3O2 |
| **Exact mass** | 117.0538 |

| **Ion type** | [M+H]+ |
|---|---|
| **m/z** | 118.0611 |

## Isotopic patterns

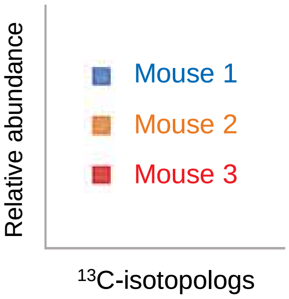


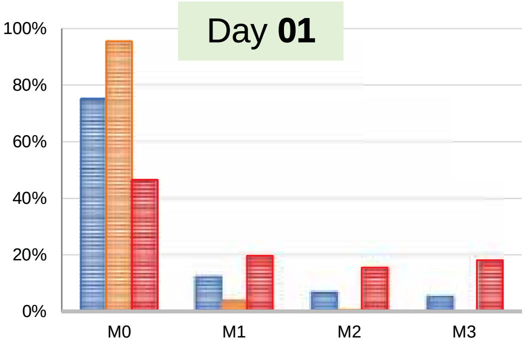


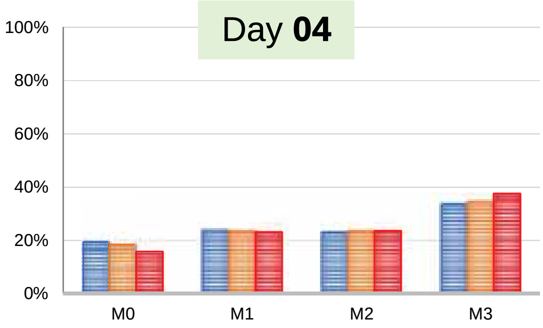


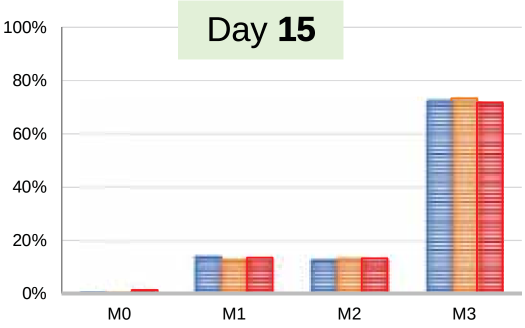


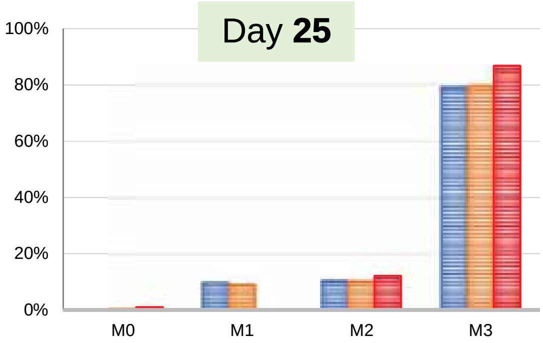


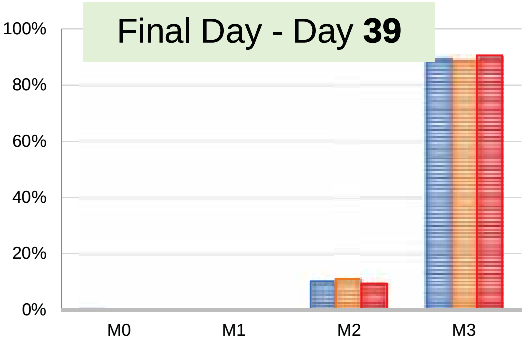


## Fractional $^{13}$C-enrichment

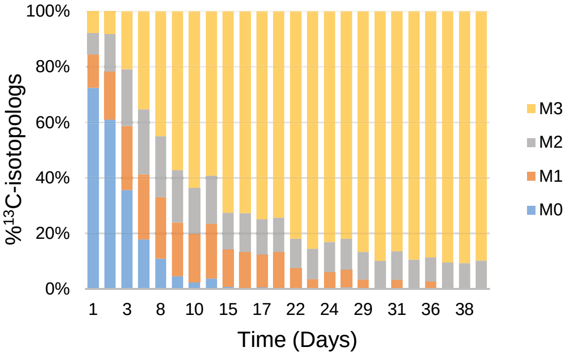


## $^{13}$C-enrichment kinetics

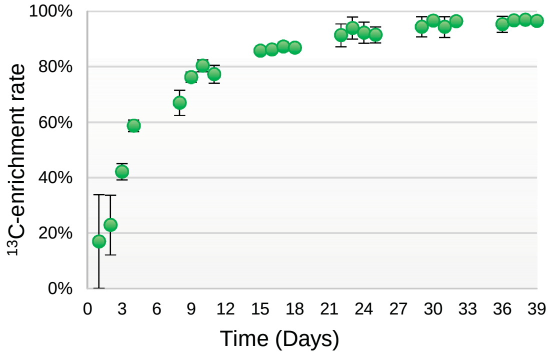

# Identified metabolites in murine urine

*Metabolites from the chemical library*

| Metabolite | Kynurenic acid |
|---|---|
| Formula | C10H7NO3 |
| Exact mass | 189.0426 |

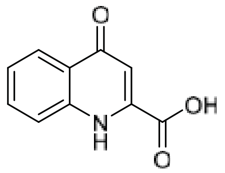


| Ion type | [M+H]+ |
|---|---|
| m/z | 190.0499 |

## Isotopic patterns

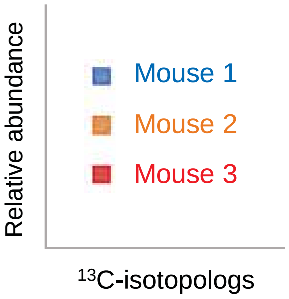


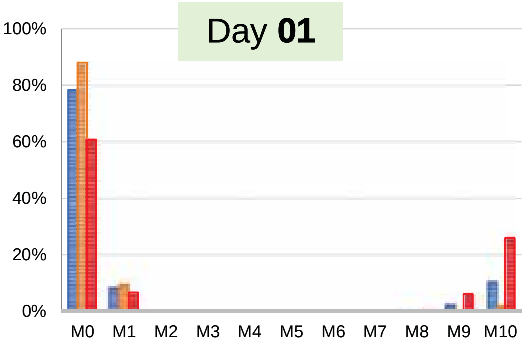


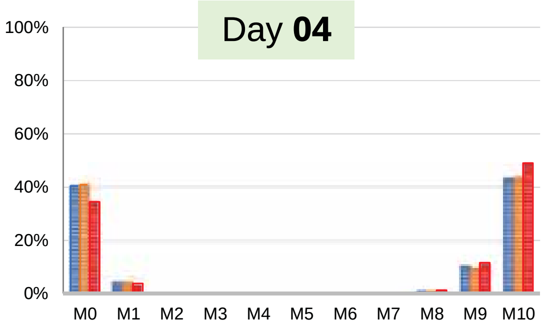


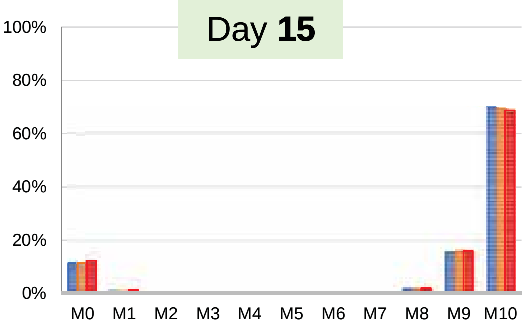


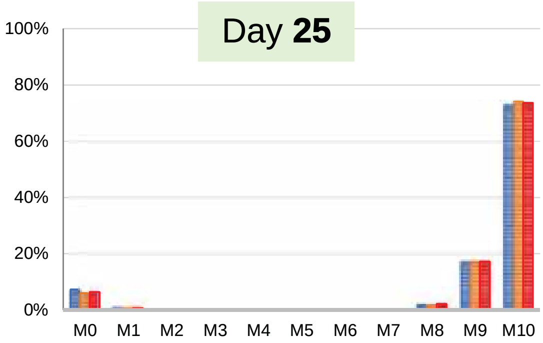


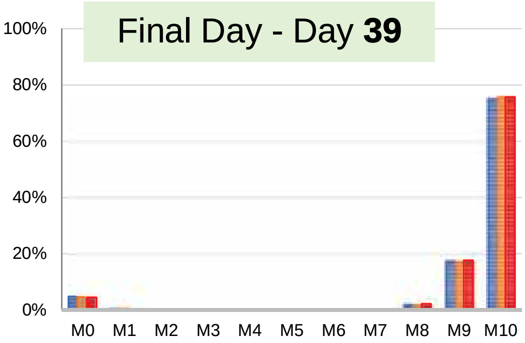


## Fractional $^{13}$C-enrichment

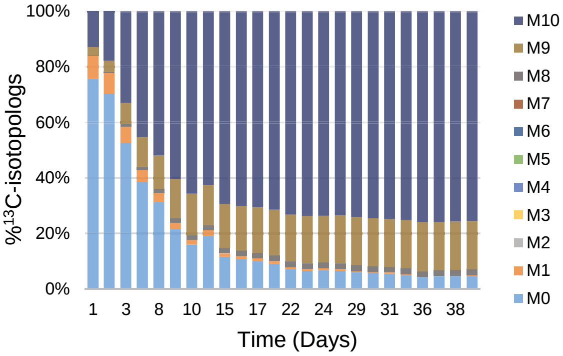


## $^{13}$C-enrichment kinetics

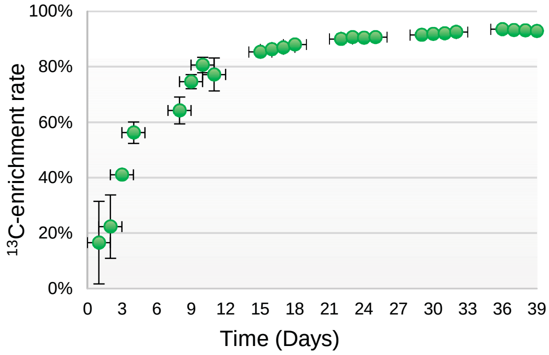

# Identified metabolites in murine urine

*Metabolites from the chemical library*

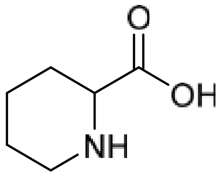


| Metabolite | Pipecolinic acid |
|---|---|
| **Formula** | C6H11NO2 |
| **Exact mass** | 129.079 |

| Ion type | [M+H]+ |
|---|---|
| **m/z** | 130.0863 |

## Isotopic patterns

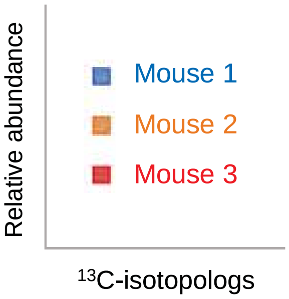


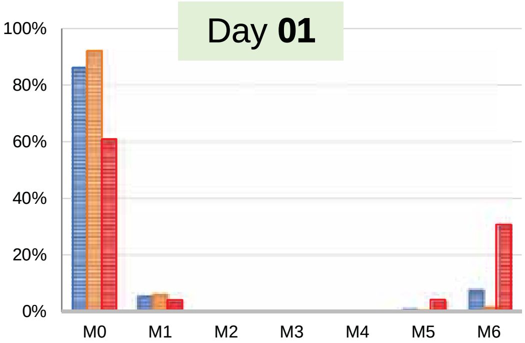


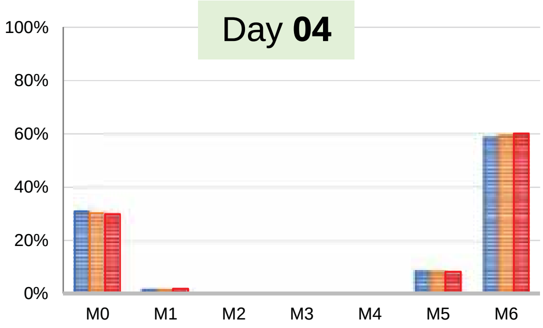


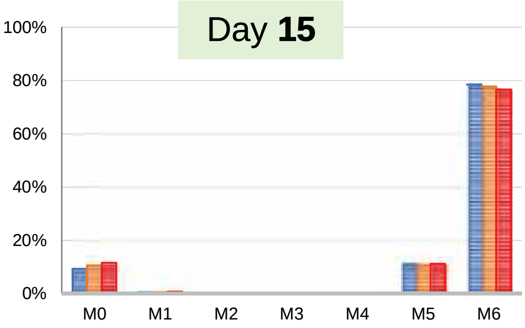


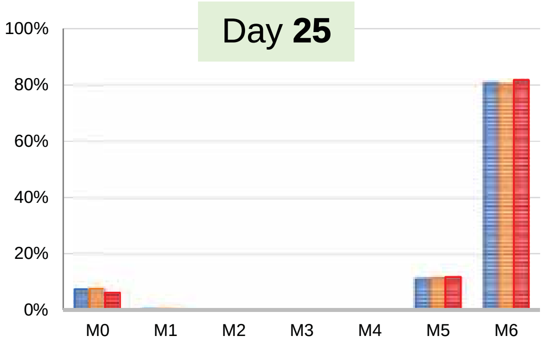


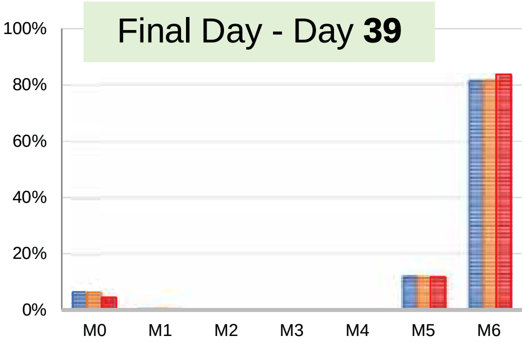


## Fractional $^{13}$C-enrichment

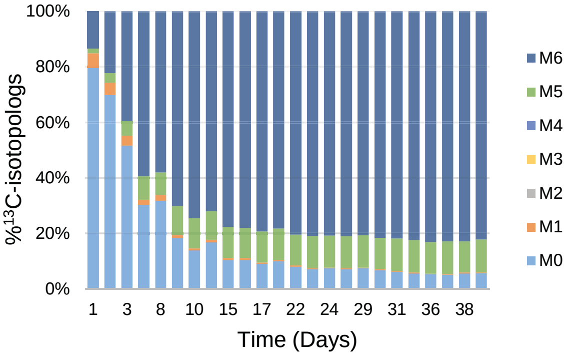


## $^{13}$C-enrichment kinetics

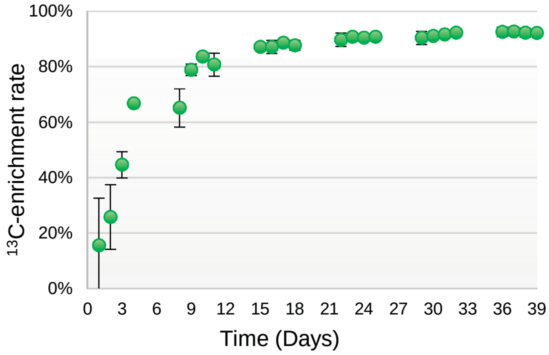

# Identified metabolites in murine urine

*Metabolites from the chemical library*

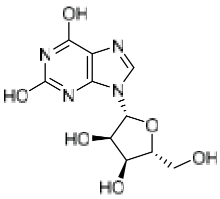


| Metabolite | Xanthosine |
|---|---|
| Formula | C10H12N4O6 |
| Exact mass | 284.0757 |

| Ion type | [M-H]- |
|---|---|
| m/z | 283.0684 |

## Isotopic patterns

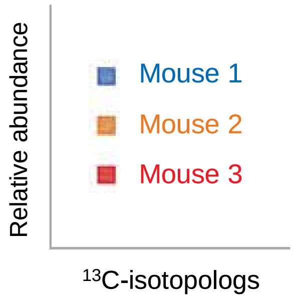


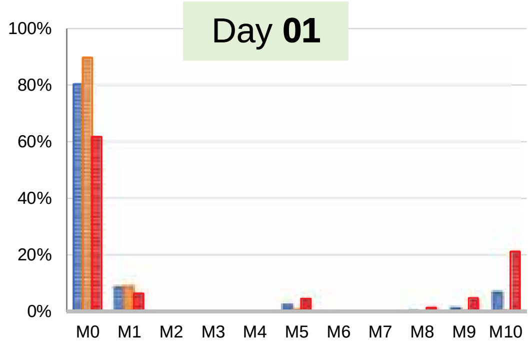


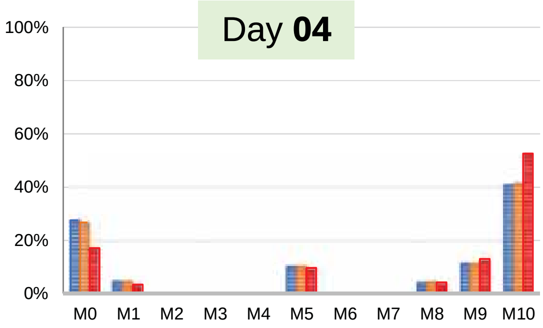


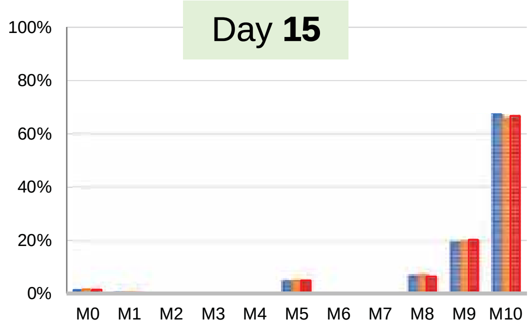


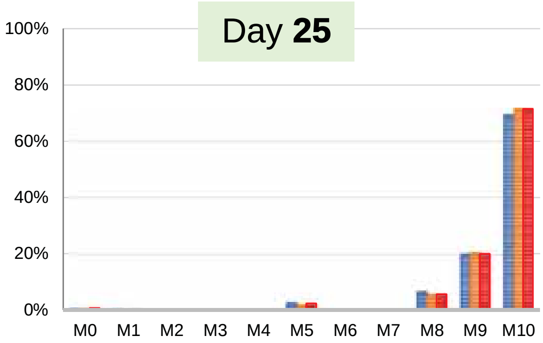


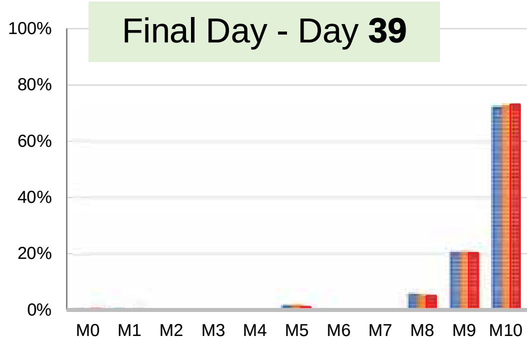


## Fractional $^{13}$C-enrichment

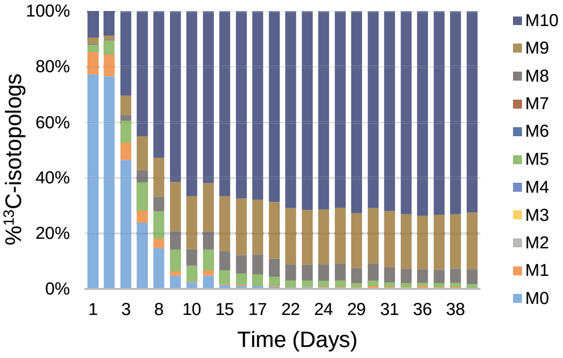


## $^{13}$C-enrichment kinetics

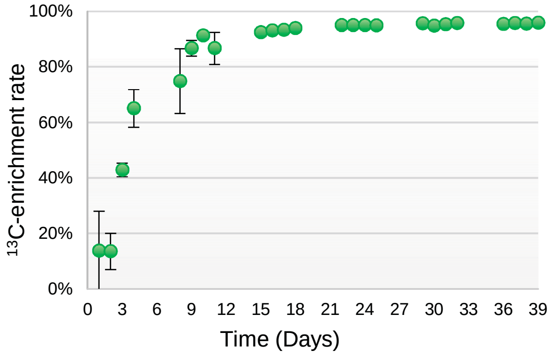

# Identified metabolites in murine urine

*Metabolites from the chemical library*

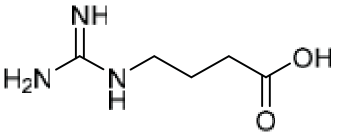


| Metabolite | 4-Guanidinobutyric acid |
|---|---|
| Formula | C5H11N3O2 |
| Exact mass | 145.0851 |

| Ion type | [M+H]+ |
|---|---|
| m/z | 146.0924 |

## Isotopic patterns

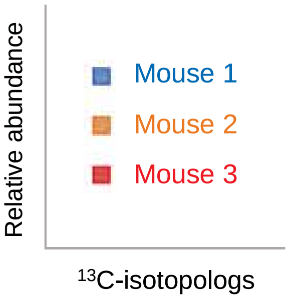


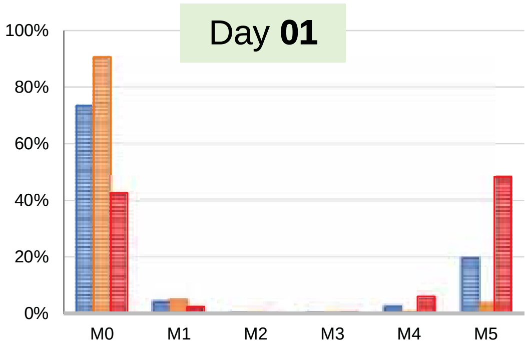


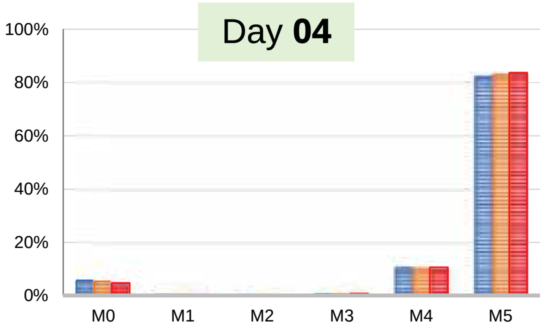


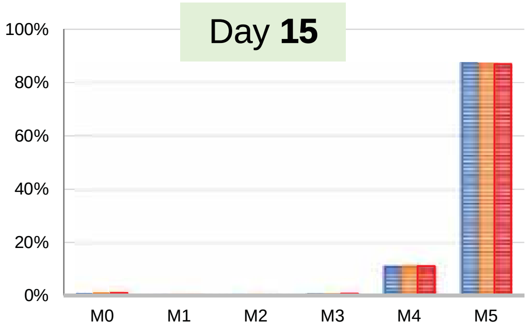


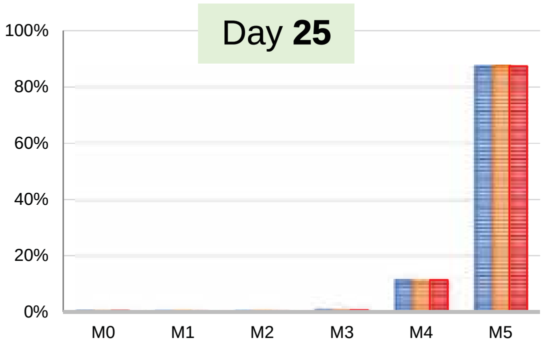


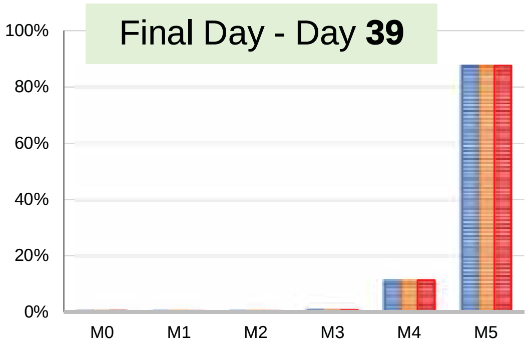


## Fractional $^{13}$C-enrichment

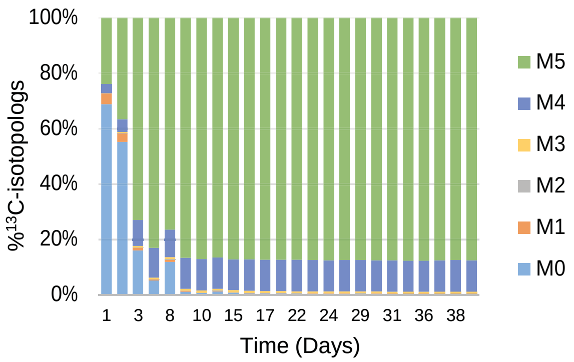


## $^{13}$C-enrichment kinetics

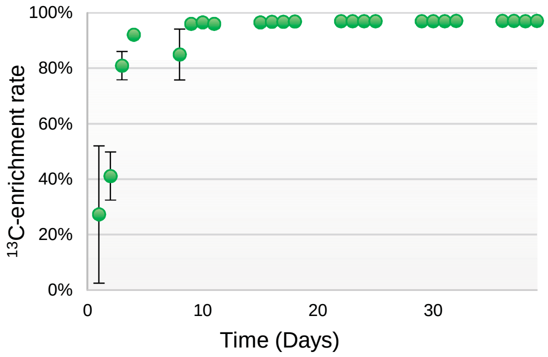

# Identified metabolites in murine urine

*Metabolites from the chemical library*

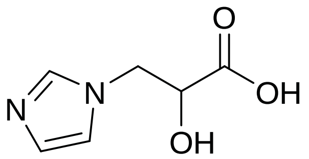


| **Metabolite** | **Imidazolelactic acid** |
|---|---|
| **Formula** | C6H8N2O3 |
| **Exact mass** | 156.0535 |

| **Ion type** | [M-H]- |
|---|---|
| **m/z** | 155.0462 |

## Isotopic patterns

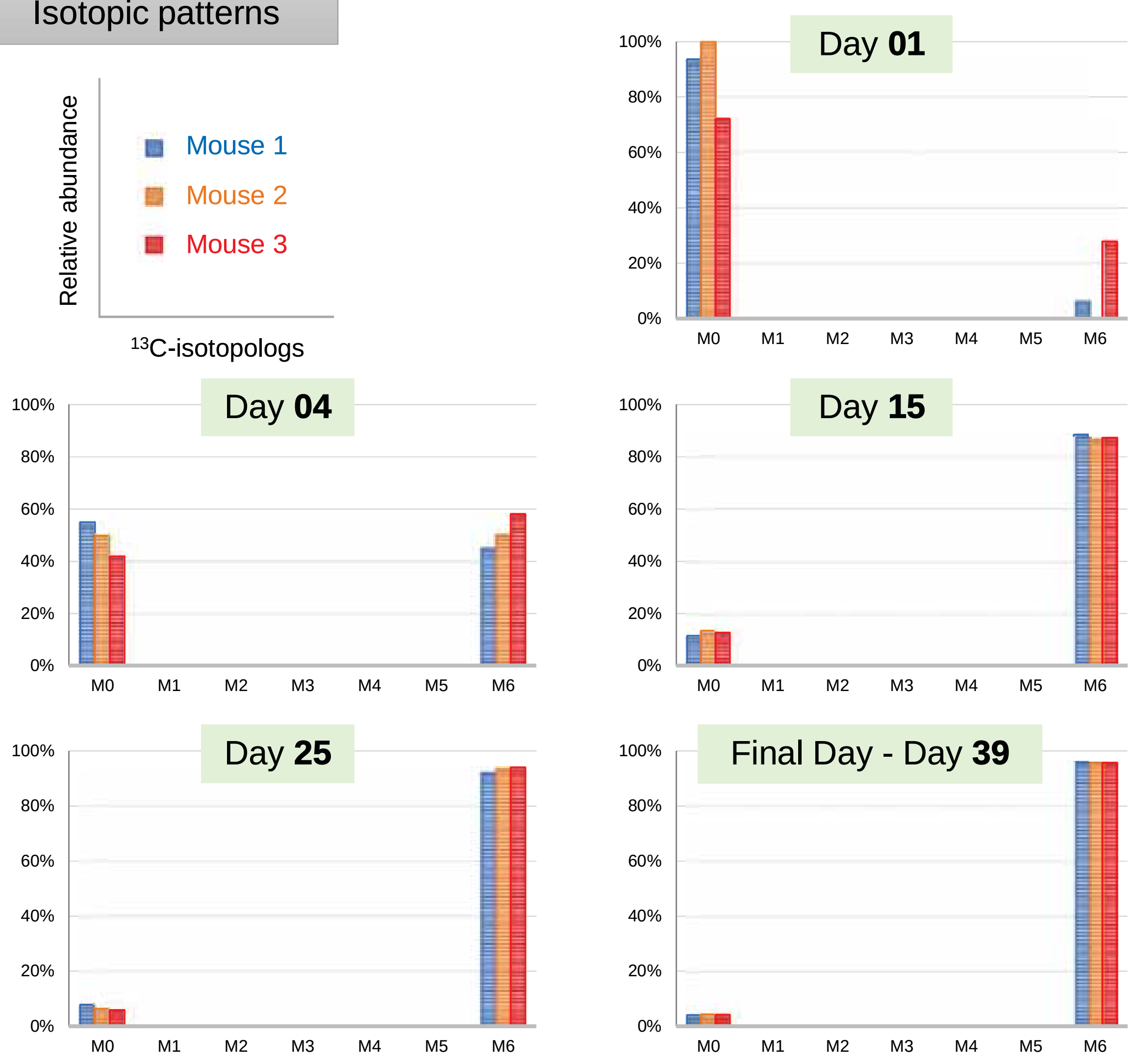


## Fractional $^{13}$C-enrichment

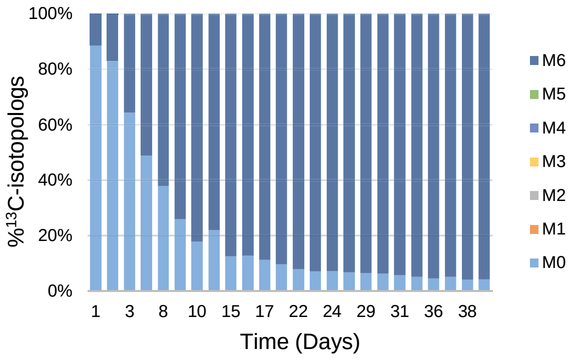


## $^{13}$C-enrichment kinetics

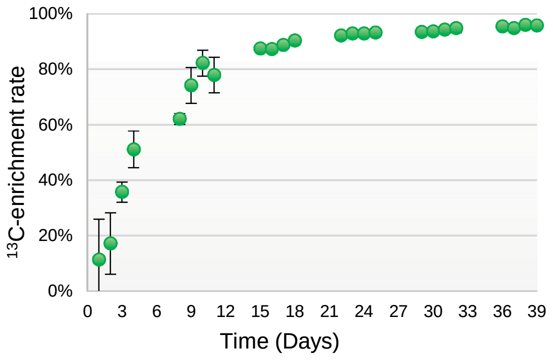

# Identified metabolites in murine urine

*Metabolites from the chemical library*

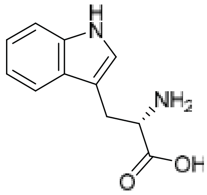


| **Metabolite** | **Tryptophan** |
|---|---|
| **Formula** | C11H12N2O2 |
| **Exact mass** | 204.0899 |

| **Ion type** | [M+H]+ |
|---|---|
| **m/z** | 205.0972 |

## Isotopic patterns

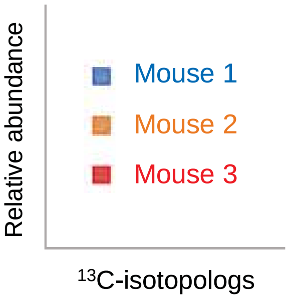


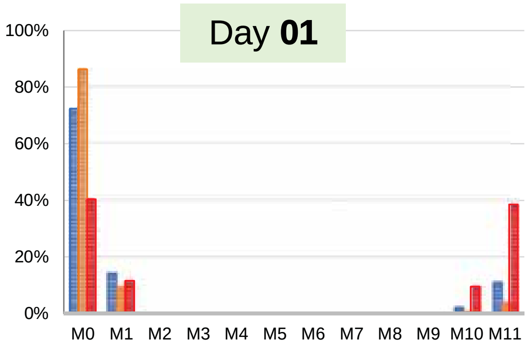


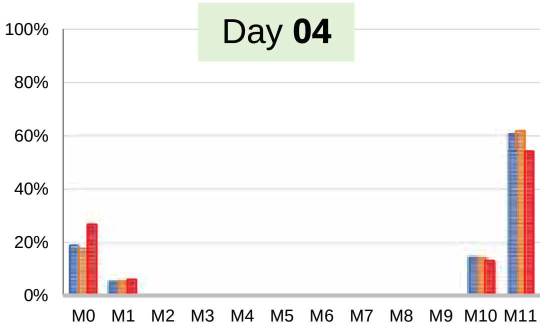


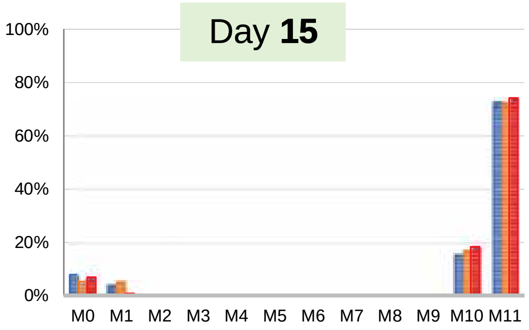


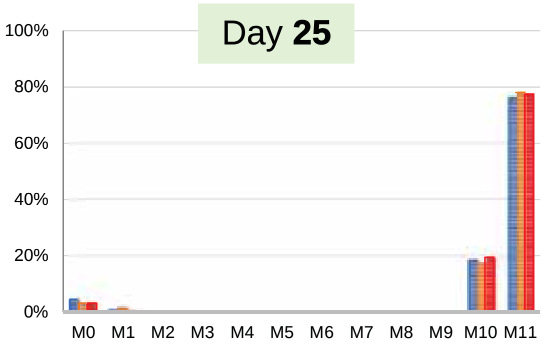


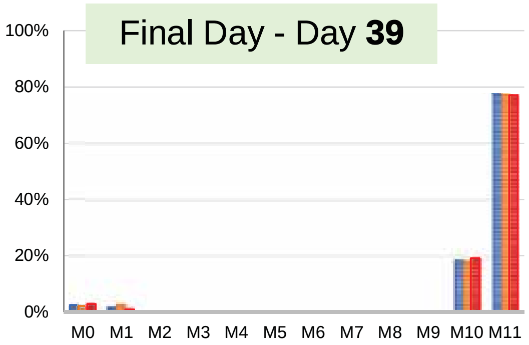


## Fractional $^{13}C$-enrichment

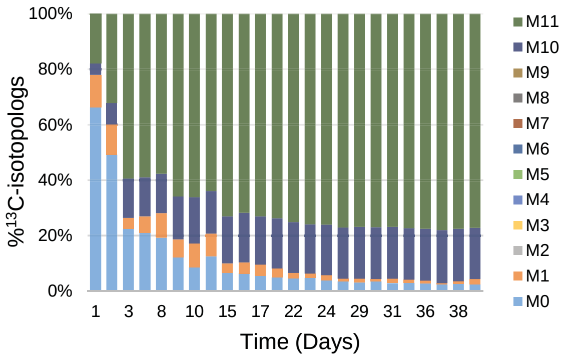


## $^{13}C$-enrichment kinetics

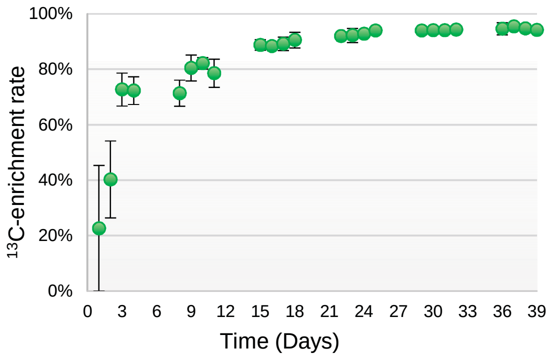

# Identified metabolites in murine urine

*Metabolites from the chemical library*

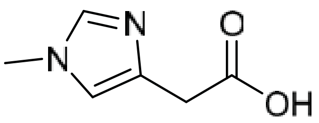

| Metabolite | Methylimidazoleacetic acid |
|---|---|
| Formula | C6H8N2O2 |
| Exact mass | 140.0586 |

| Ion type | [M+H]+ |
|---|---|
| m/z | 141.0659 |

## Isotopic patterns

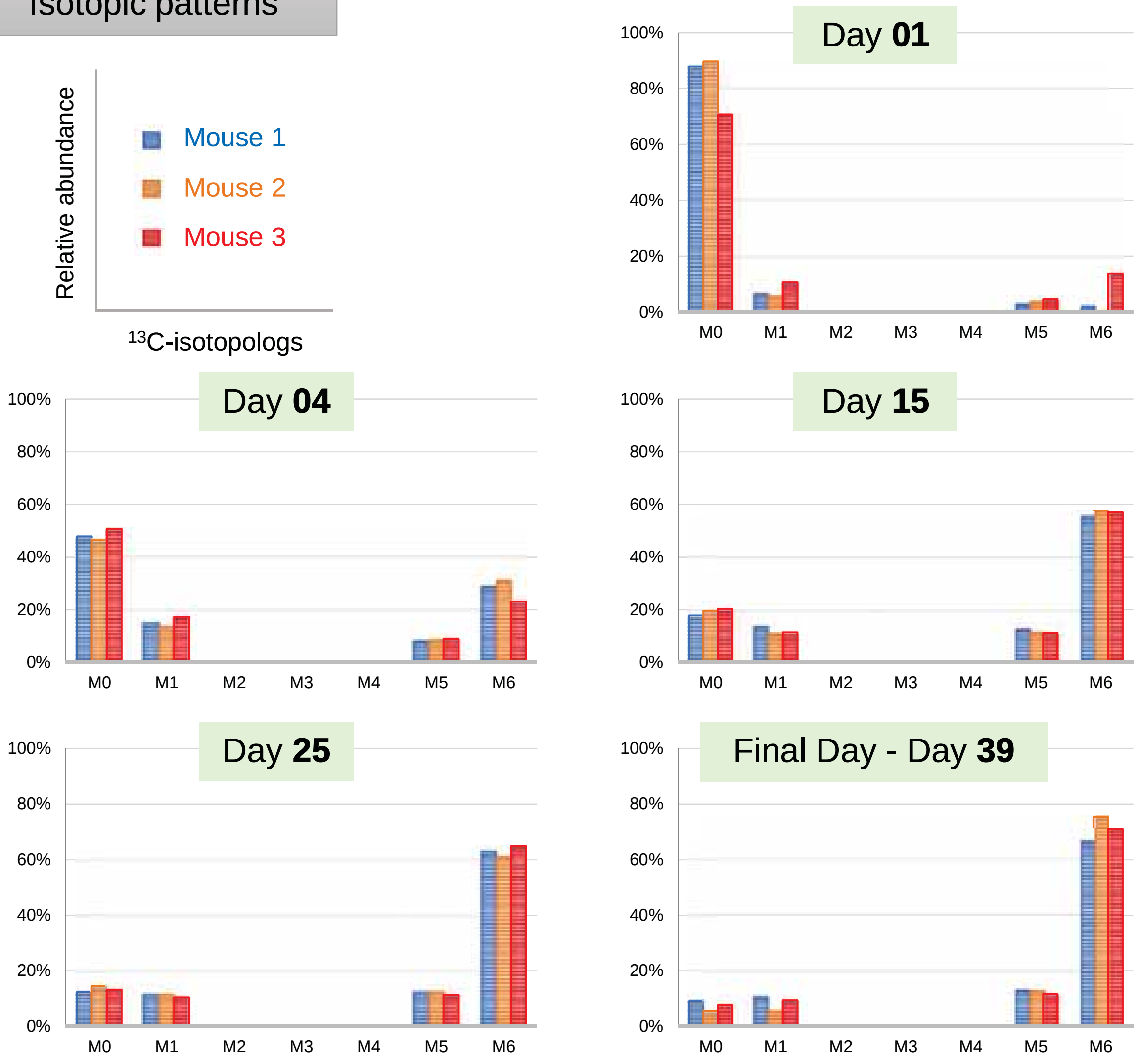


## Fractional $^{13}$C-enrichment

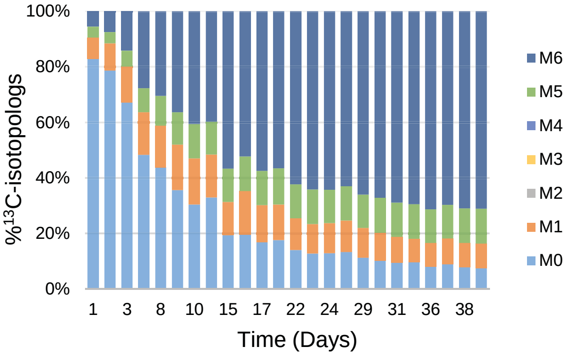


## $^{13}$C-enrichment kinetics

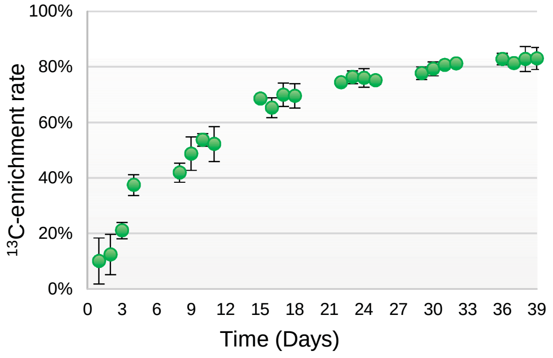

# Identified metabolites in murine urine

*Metabolites from the chemical library*

| Metabolite | Azelaic acid |
|---|---|
| Formula | $C_9H_{16}O_4$ |
| Exact mass | 188.1049 |

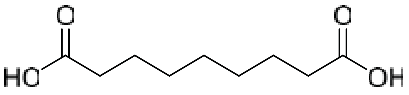


| Ion type | [M-H]- |
|---|---|
| m/z | 187.0976 |

## Isotopic patterns

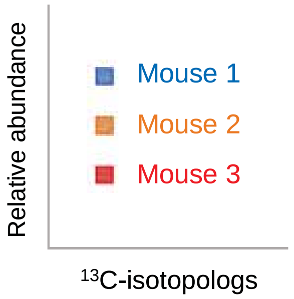


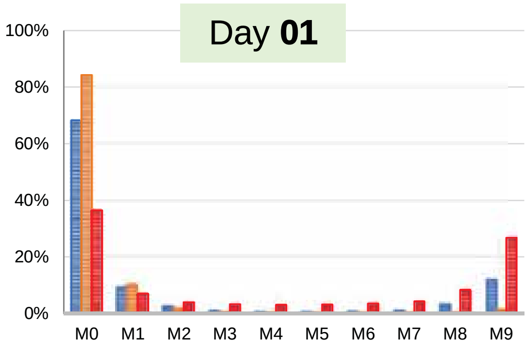


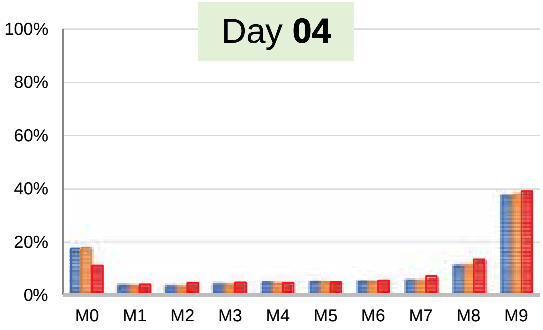


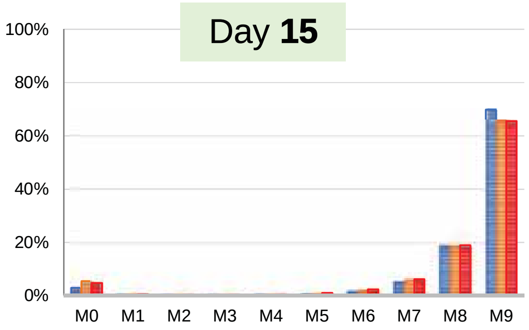


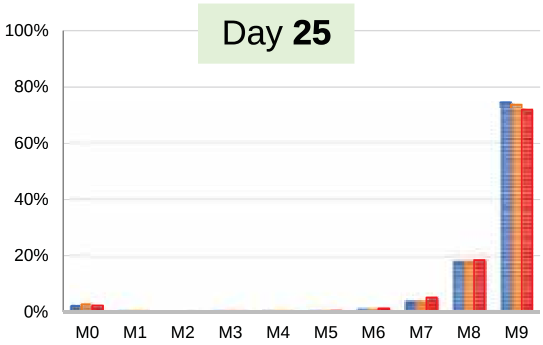


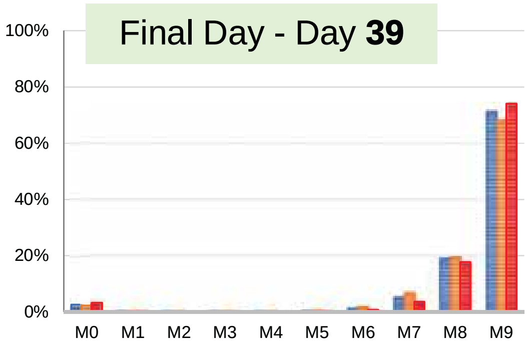


## Fractional $^{13}$C-enrichment

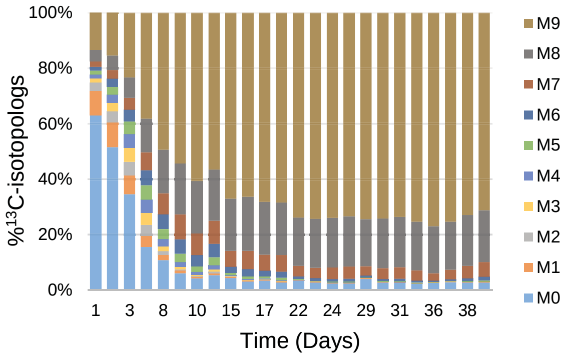


## $^{13}$C-enrichment kinetics

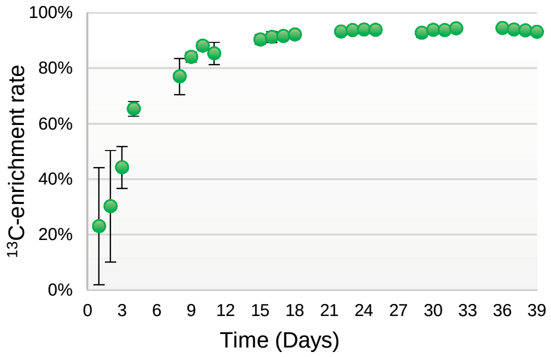

# Identified metabolites in murine urine

*Metabolites from the chemical library*

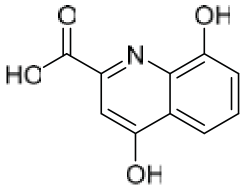


| **Metabolite** | **Xanthurenic acid** |
|---|---|
| **Formula** | C10H7NO4 |
| **Exact mass** | 205.0375 |

| **Ion type** | [M+H]+ |
|---|---|
| **m/z** | 206.0448 |

## Isotopic patterns

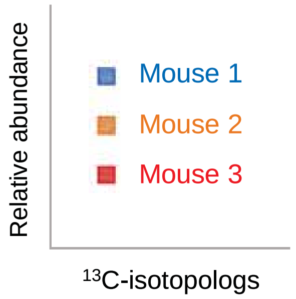


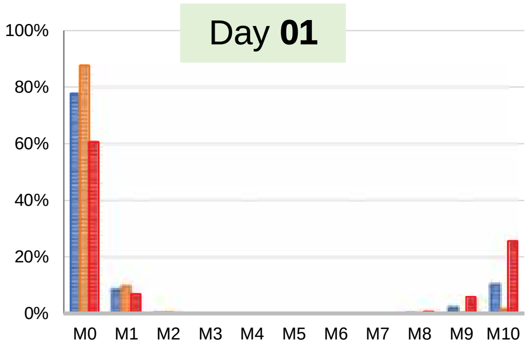


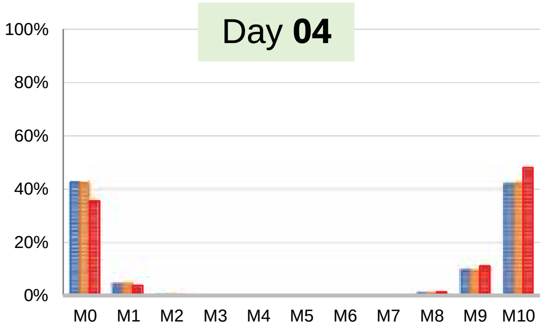


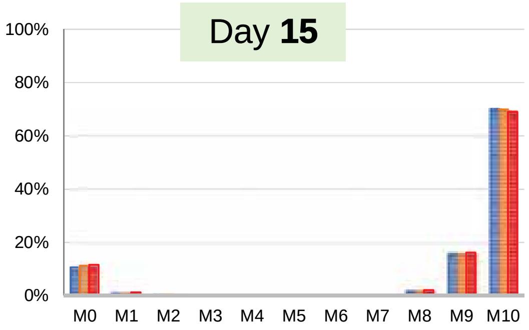


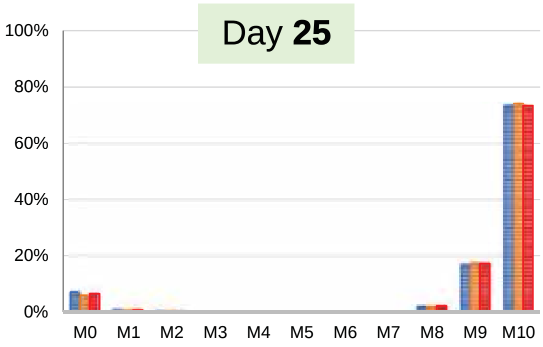


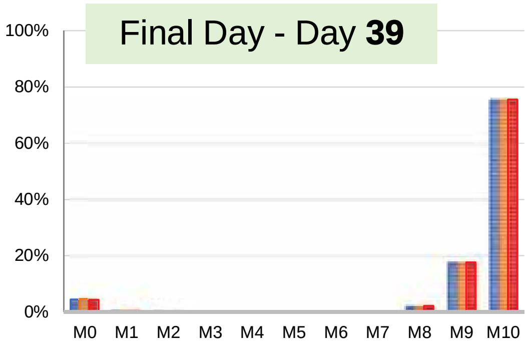


## Fractional $^{13}$C-enrichment

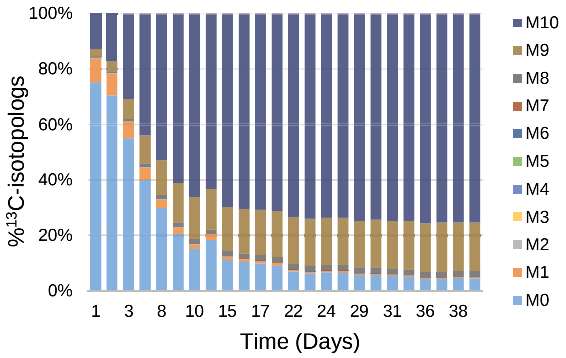


## $^{13}$C-enrichment kinetics

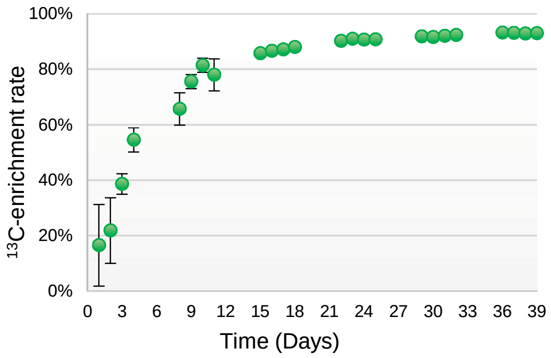

# Identified metabolites in murine urine

*Metabolites from the chemical library*

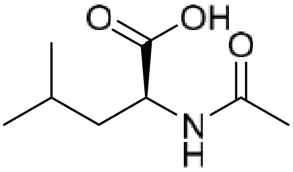


| Metabolite | N-Acetyl-L-leucine |
|---|---|
| Formula | C8H15NO3 |
| Exact mass | 173.1052 |

| Ion type | [M-H]- |
|---|---|
| m/z | 172.0979 |

## Isotopic patterns

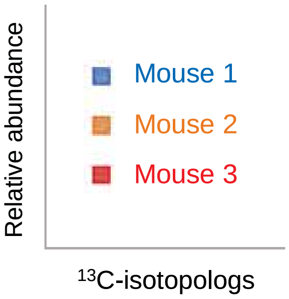


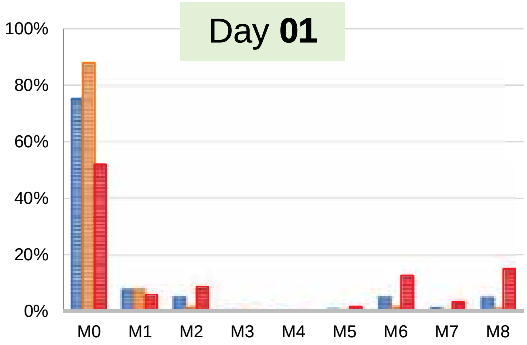


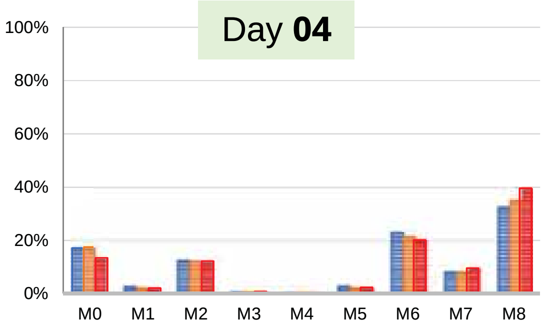


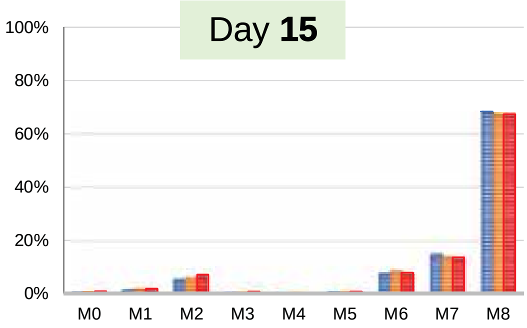


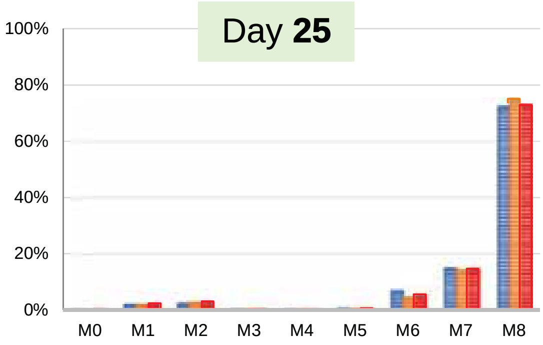


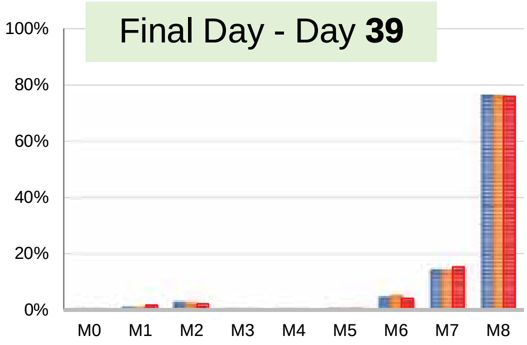

# Identified metabolites in murine urine

*Metabolites from the chemical library*

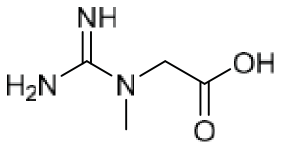

| Metabolite | Creatine |
|---|---|
| Formula | C4H9N3O2 |
| Exact mass | 131.0695 |

| Ion type | [M+H]+ |
|---|---|
| m/z | 132.0768 |

## Isotopic patterns

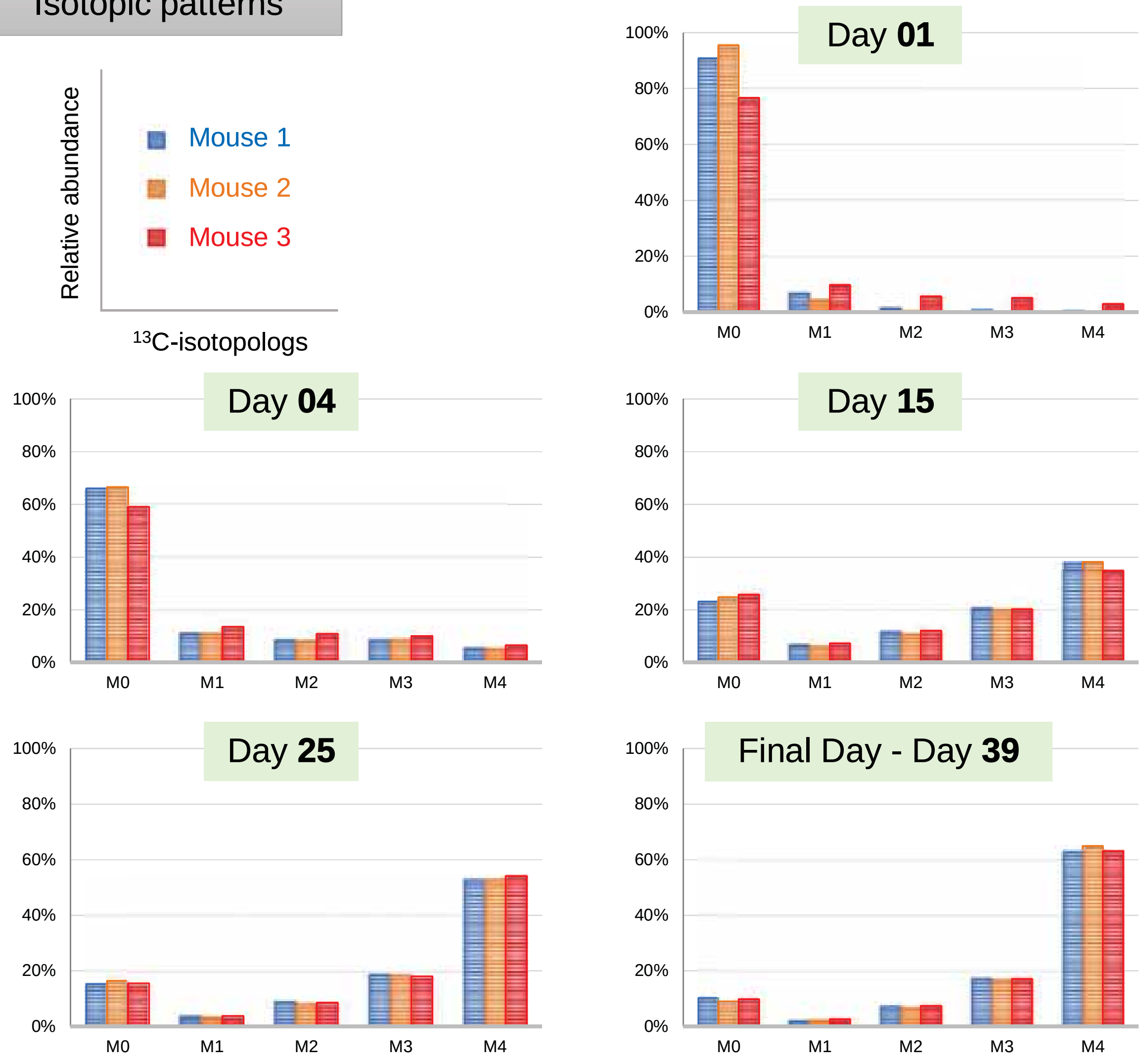


## Fractional $^{13}$C-enrichment

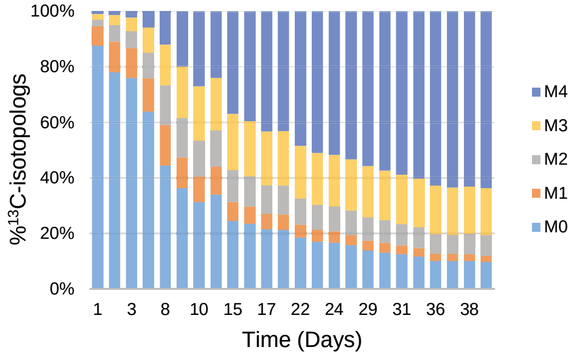


## $^{13}$C-enrichment kinetics

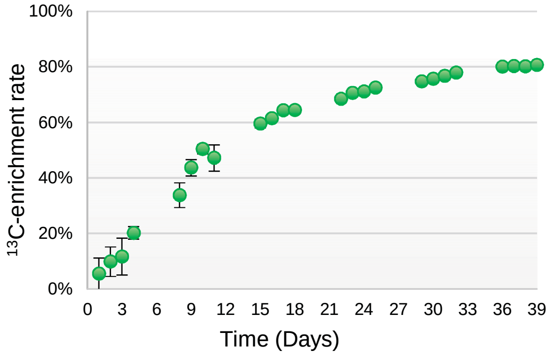

# Identified metabolites in murine urine

*Metabolites from the chemical library*

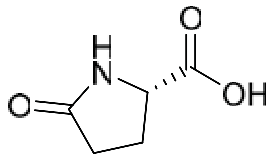

| Metabolite | Pyroglutamic acid |
|---|---|
| Formula | C5H7NO3 |
| Exact mass | 129.0426 |

| Ion type | [M+H]+ |
|---|---|
| m/z | 130.0499 |

## Isotopic patterns

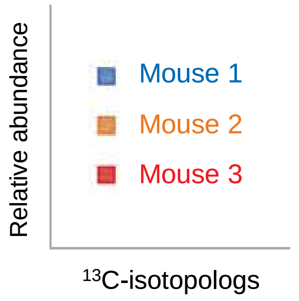


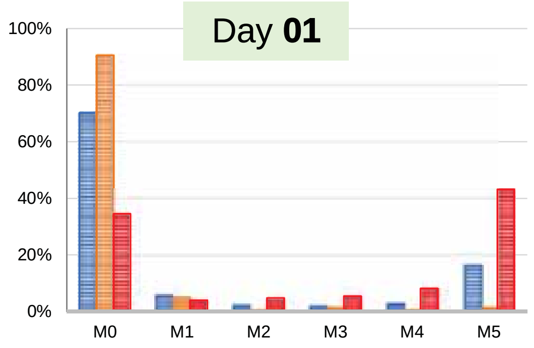


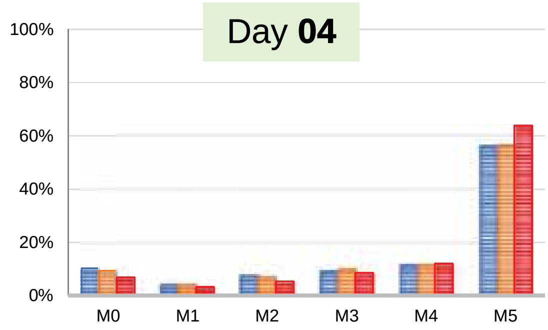


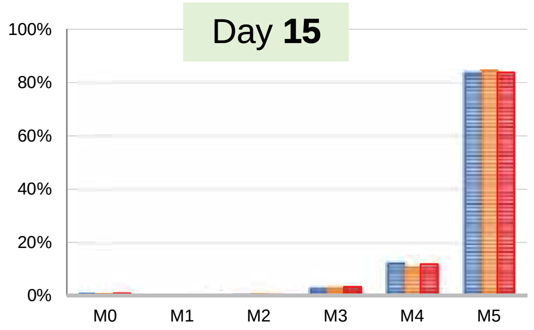


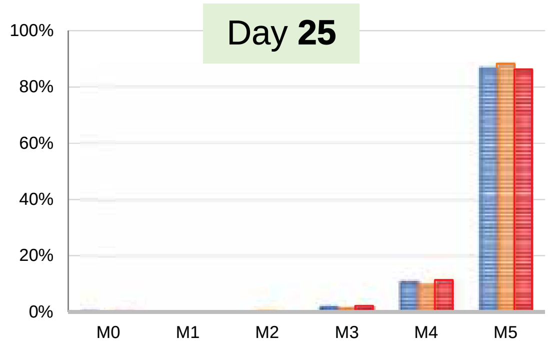


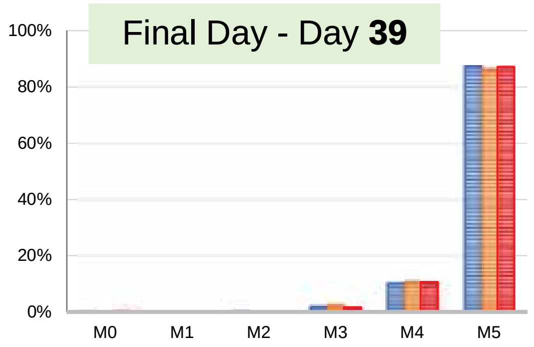


## Fractional $^{13}$C-enrichment

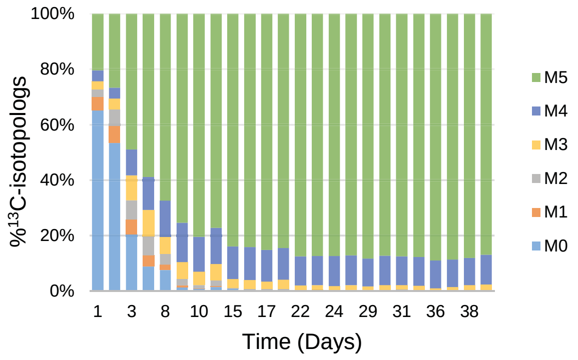


## $^{13}$C-enrichment kinetics

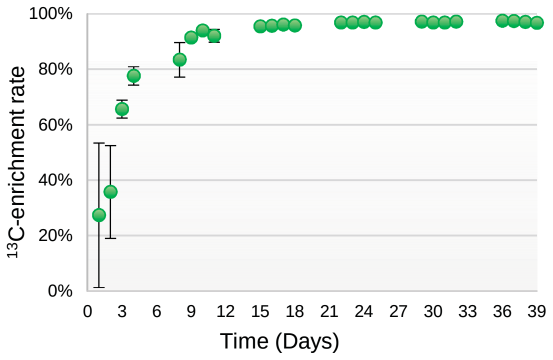

# Identified metabolites in murine urine

*Metabolites from the chemical library*

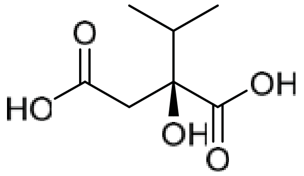


| **Metabolite** | **2-Isopropylmalic acid** |
|---|---|
| **Formula** | C7H12O5 |
| **Exact mass** | 176.0685 |

| **Ion type** | [M-H]- |
|---|---|
| **m/z** | 175.0612 |

## Isotopic patterns

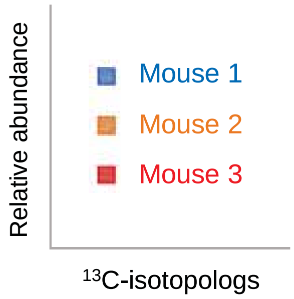


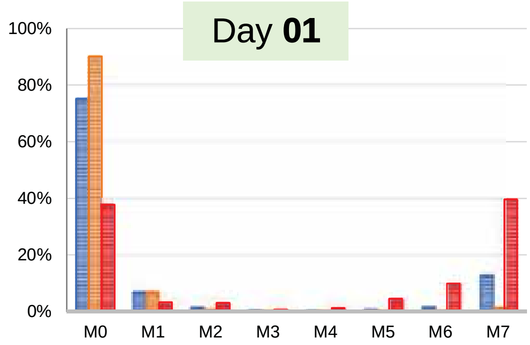


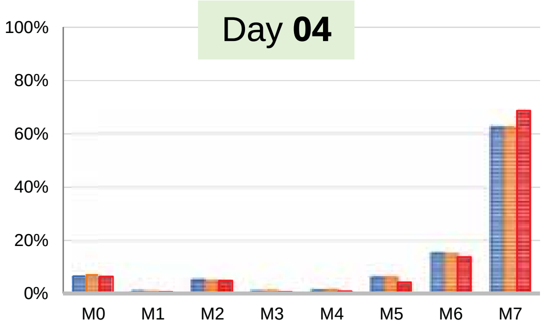


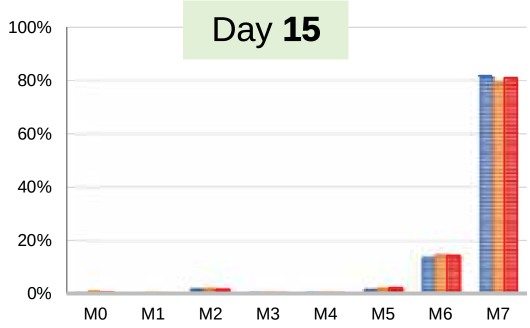


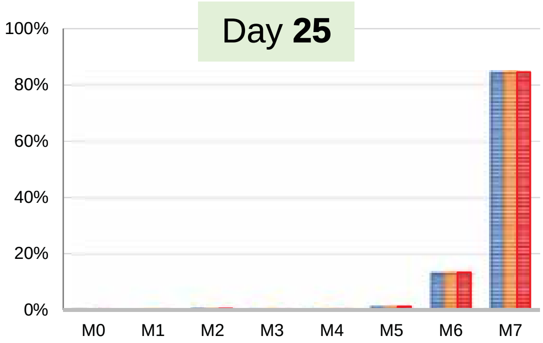


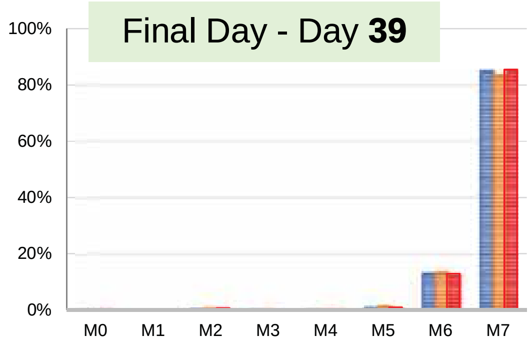

# Identified metabolites in murine urine

*Metabolites from the chemical library*

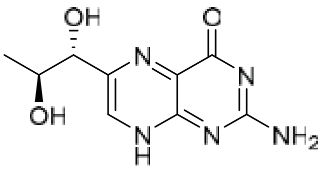

| Metabolite | 6-Biopterin |
|---|---|
| Formula | C9H11N5O3 |
| Exact mass | 237.0862 |

| Ion type | [M+H]+ |
|---|---|
| m/z | 238.0935 |

## Isotopic patterns

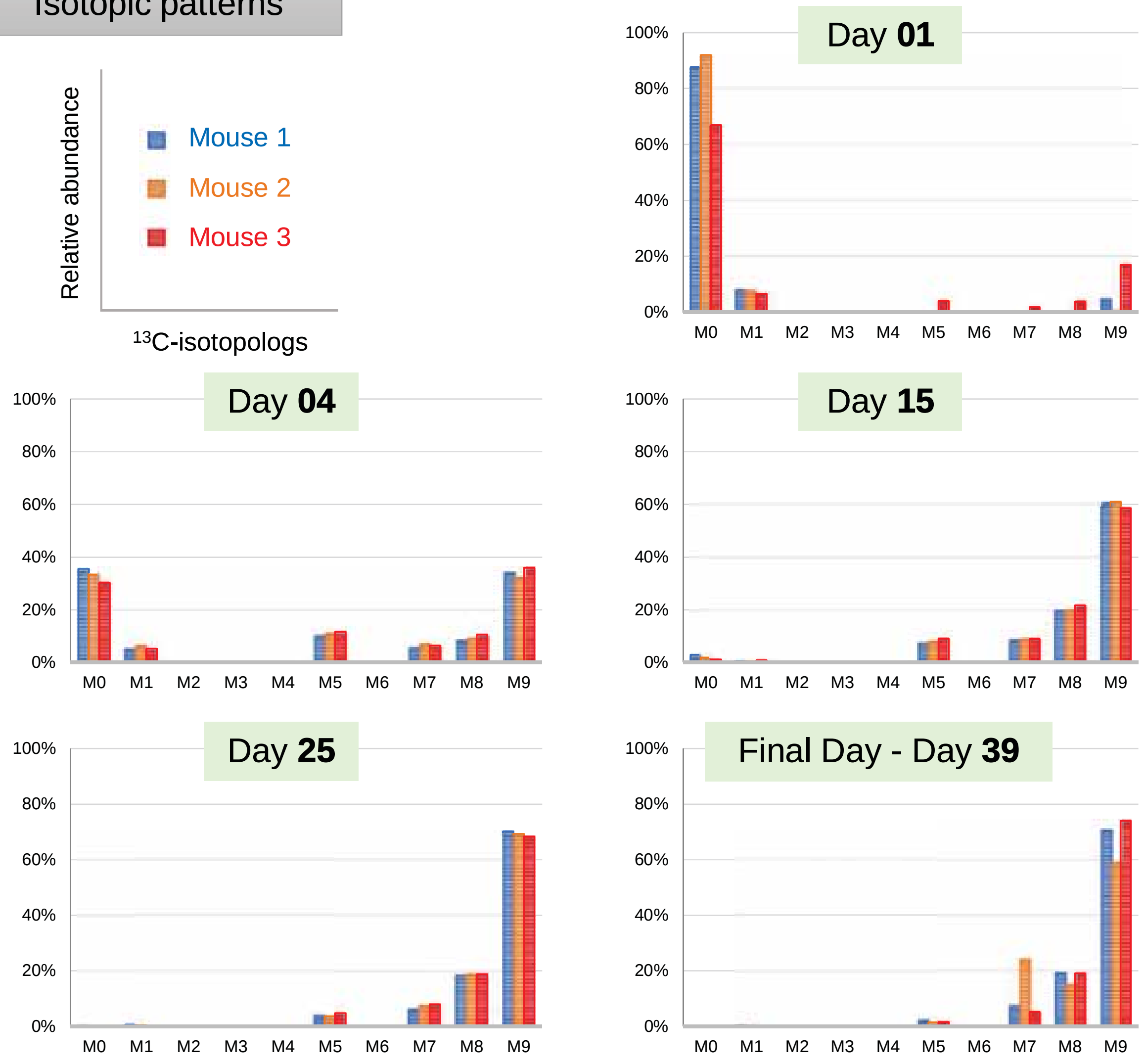


## Fractional $^{13}$C-enrichment

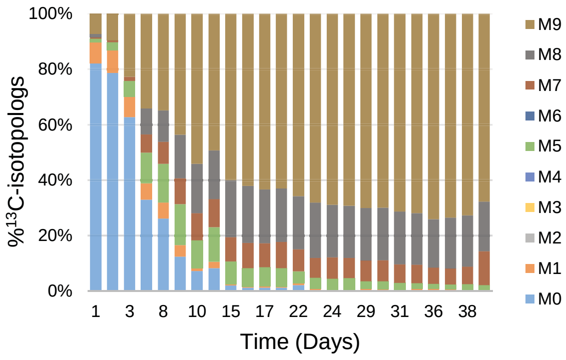


## $^{13}$C-enrichment kinetics

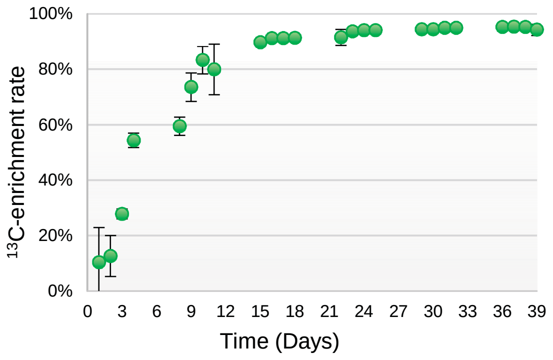

# Identified metabolites in murine urine

*Metabolites from the chemical library*

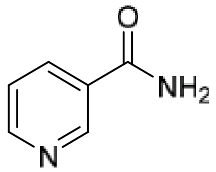


| **Metabolite** | **Nicotinamide** |
|---|---|
| **Formula** | C6H6N2O |
| **Exact mass** | 122.048 |

| **Ion type** | [M+H]+ |
|---|---|
| **m/z** | 123.0553 |

## Isotopic patterns

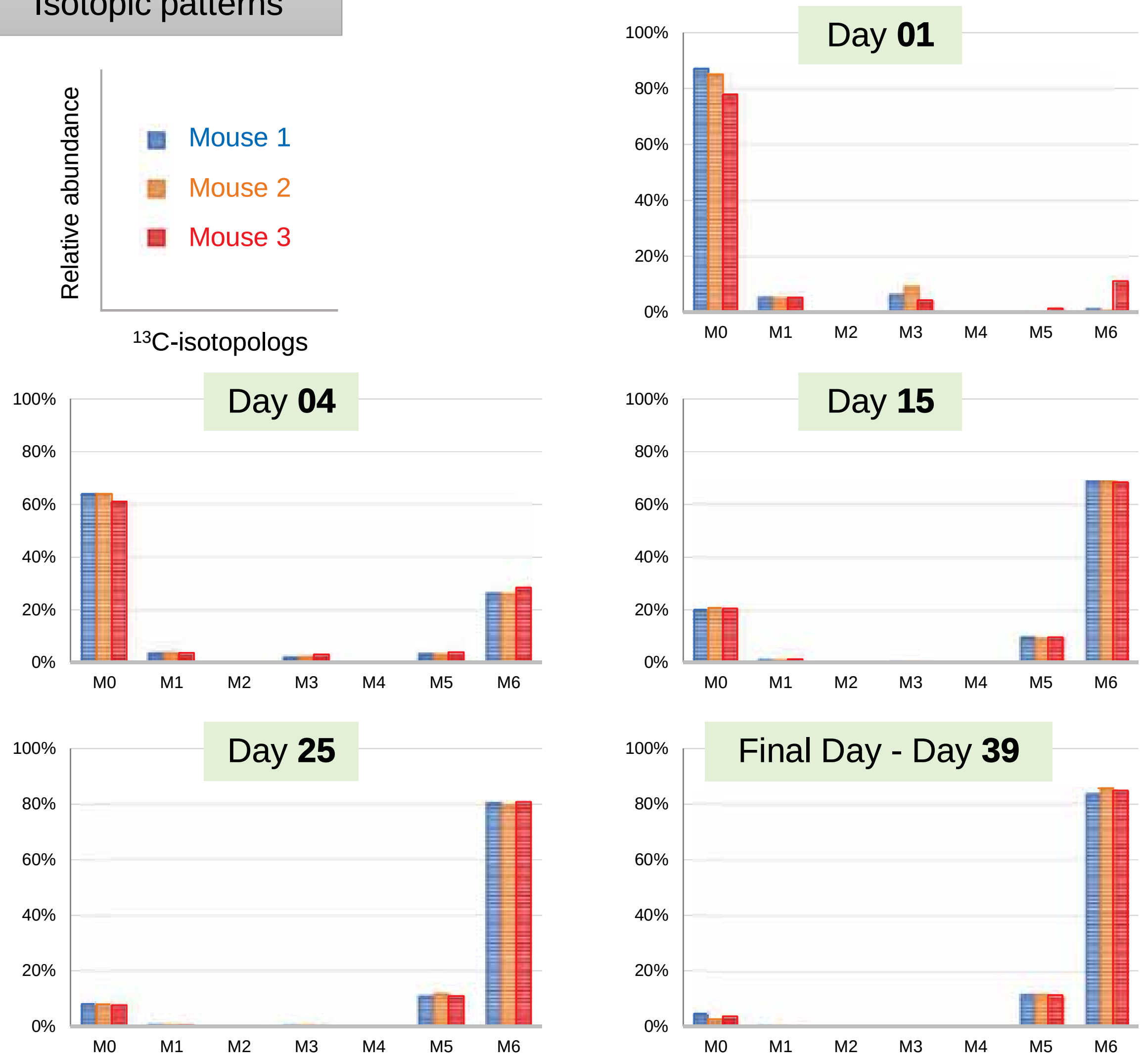


## Fractional $^{13}$C-enrichment

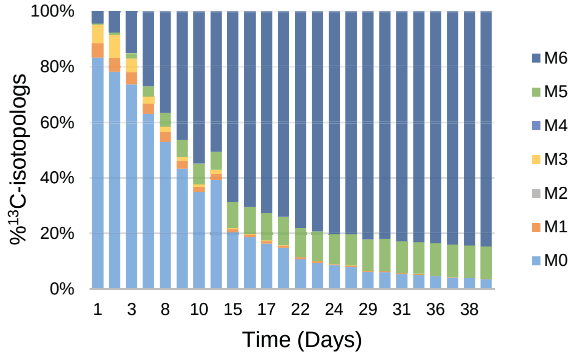


## $^{13}$C-enrichment kinetics

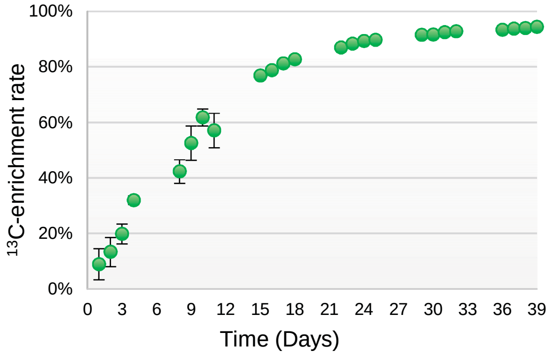

# Identified metabolites in murine urine

*Metabolites from the chemical library*

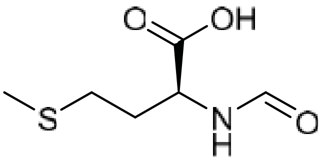


| **Metabolite** | **N-Formyl-L-methionine** |
|---|---|
| **Formula** | C6H11NO3S |
| **Exact mass** | 177.046 |

| **Ion type** | [M-H]- |
|---|---|
| **m/z** | 176.0387 |

## Isotopic patterns

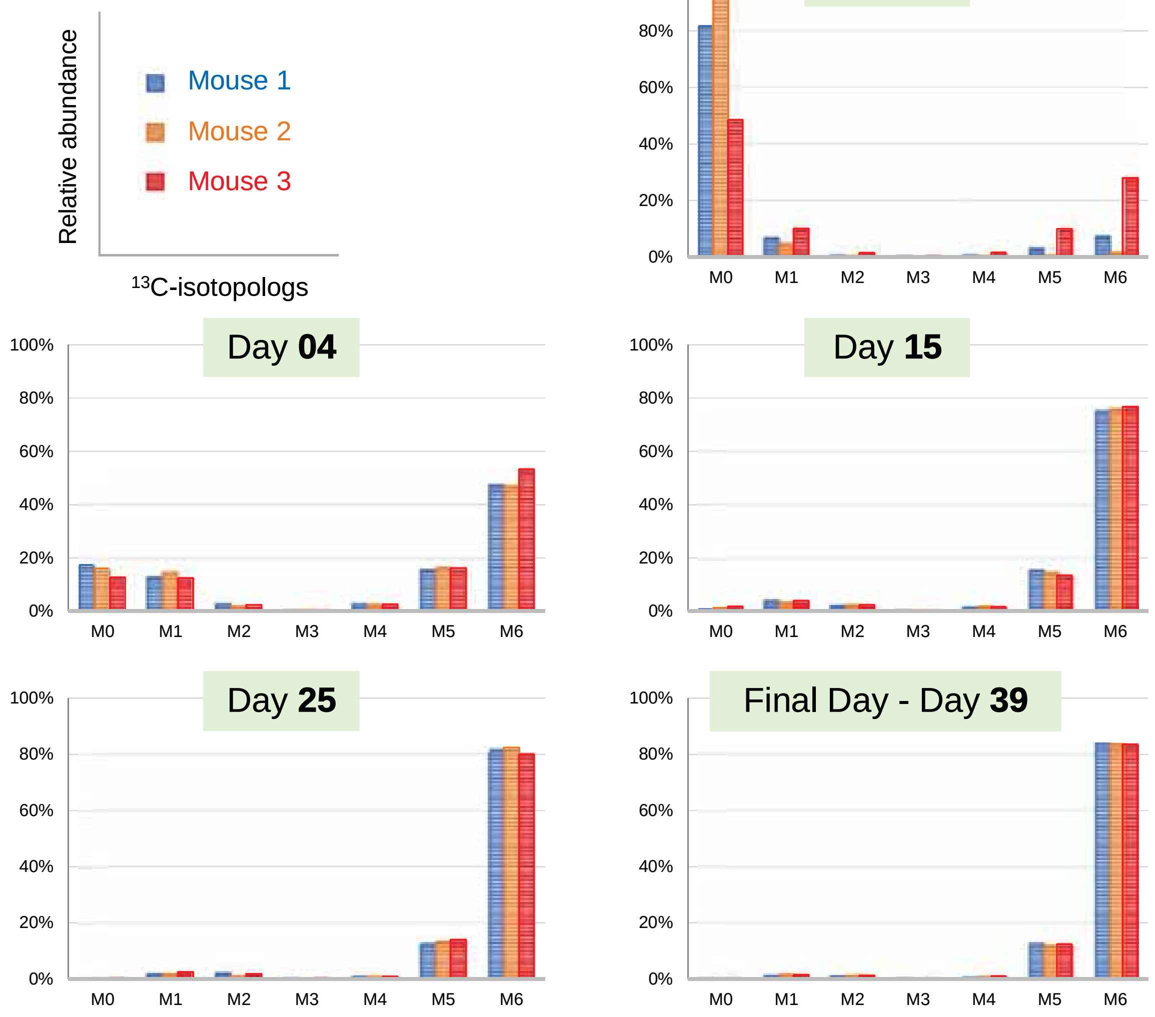

# Identified metabolites in murine urine

*Metabolites from the chemical library*

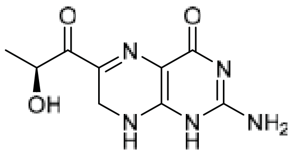


| **Metabolite** | **Sepiapterin** |
|---|---|
| **Formula** | C9H11N5O3 |
| **Exact mass** | 237.0862 |

| **Ion type** | [M+H]+ |
|---|---|
| **m/z** | 238.0935 |

## Isotopic patterns

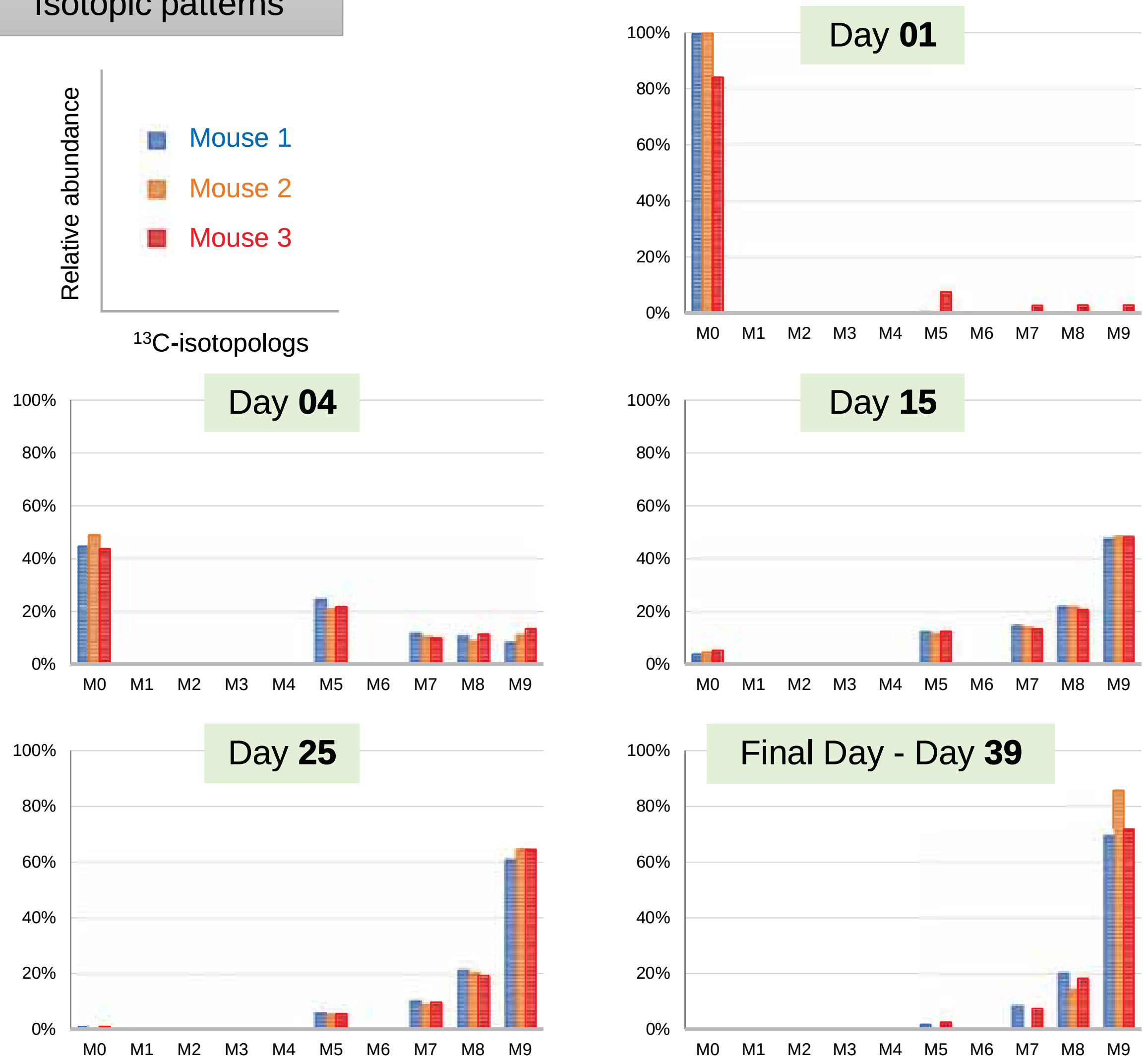


## Fractional $^{13}$C-enrichment

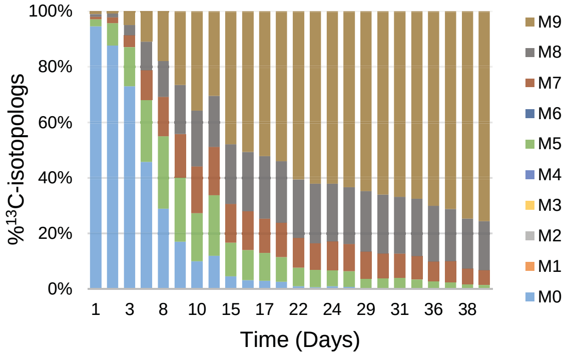


## $^{13}$C-enrichment kinetics

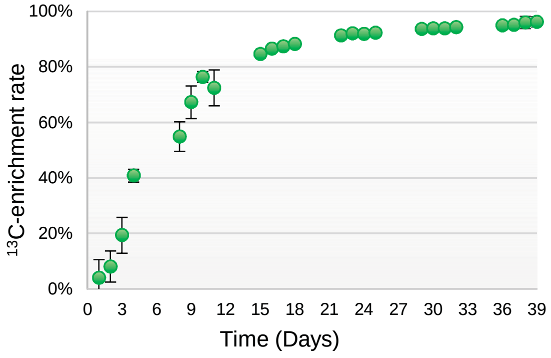

# Identified metabolites in murine urine

*Metabolites from the chemical library*

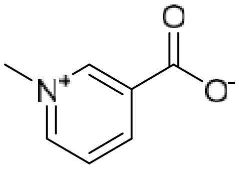


| Metabolite | Trigonelline |
|---|---|
| **Formula** | C7H7NO2 |
| **Exact mass** | 137.0477 |

| Ion type | [M+H]+ |
|---|---|
| **m/z** | 138.055 |

## Isotopic patterns

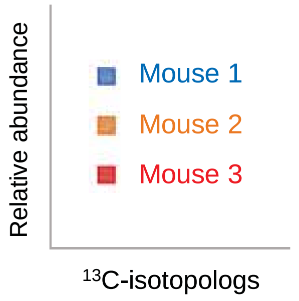


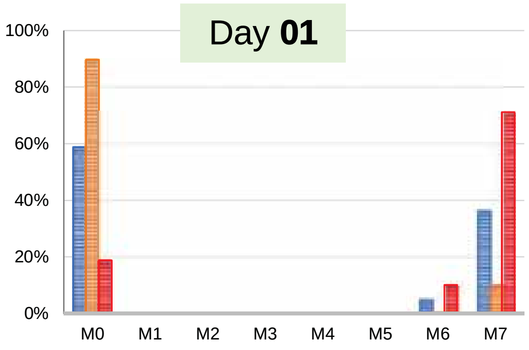


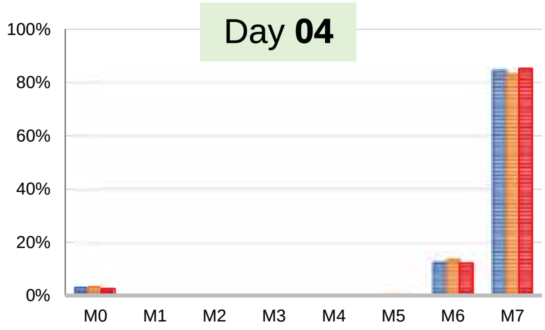


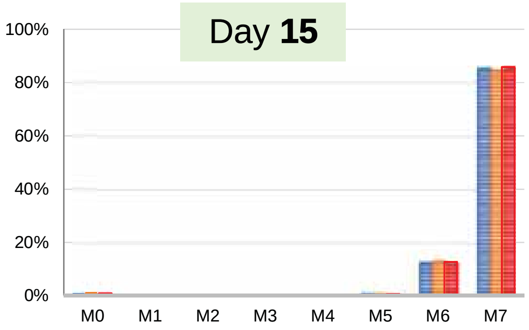


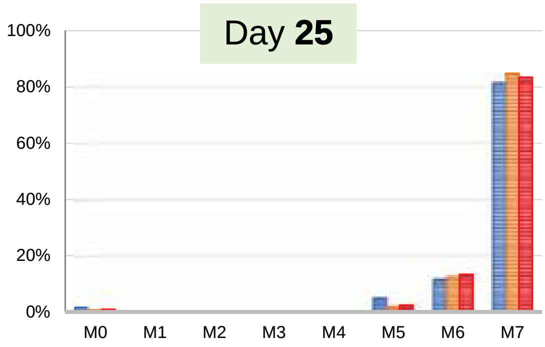


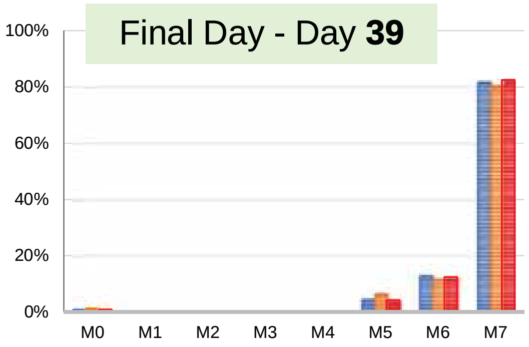


## Fractional $^{13}$C-enrichment

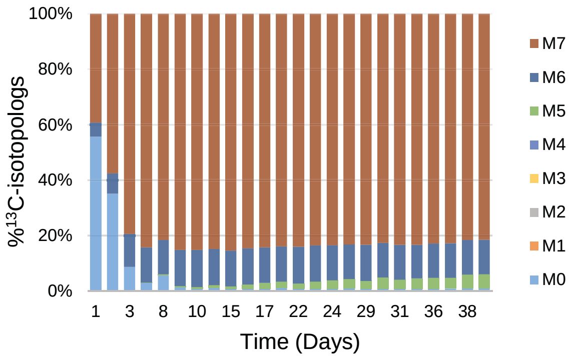


## $^{13}$C-enrichment kinetics

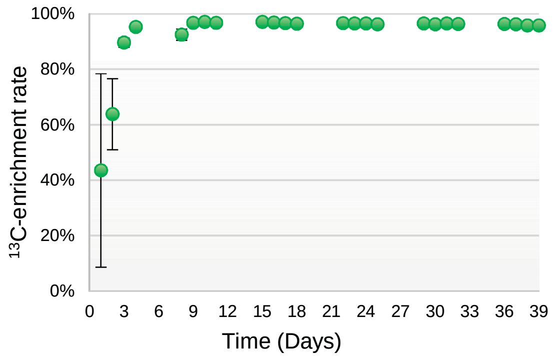

# Identified metabolites in murine urine

*Metabolites from the chemical library*

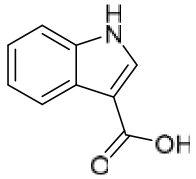


| Metabolite | Indole-3-carboxylic acid |
|---|---|
| Formula | C9H7NO2 |
| Exact mass | 161.0477 |

| Ion type | [M-H]- |
|---|---|
| m/z | 160.0404 |

## Isotopic patterns

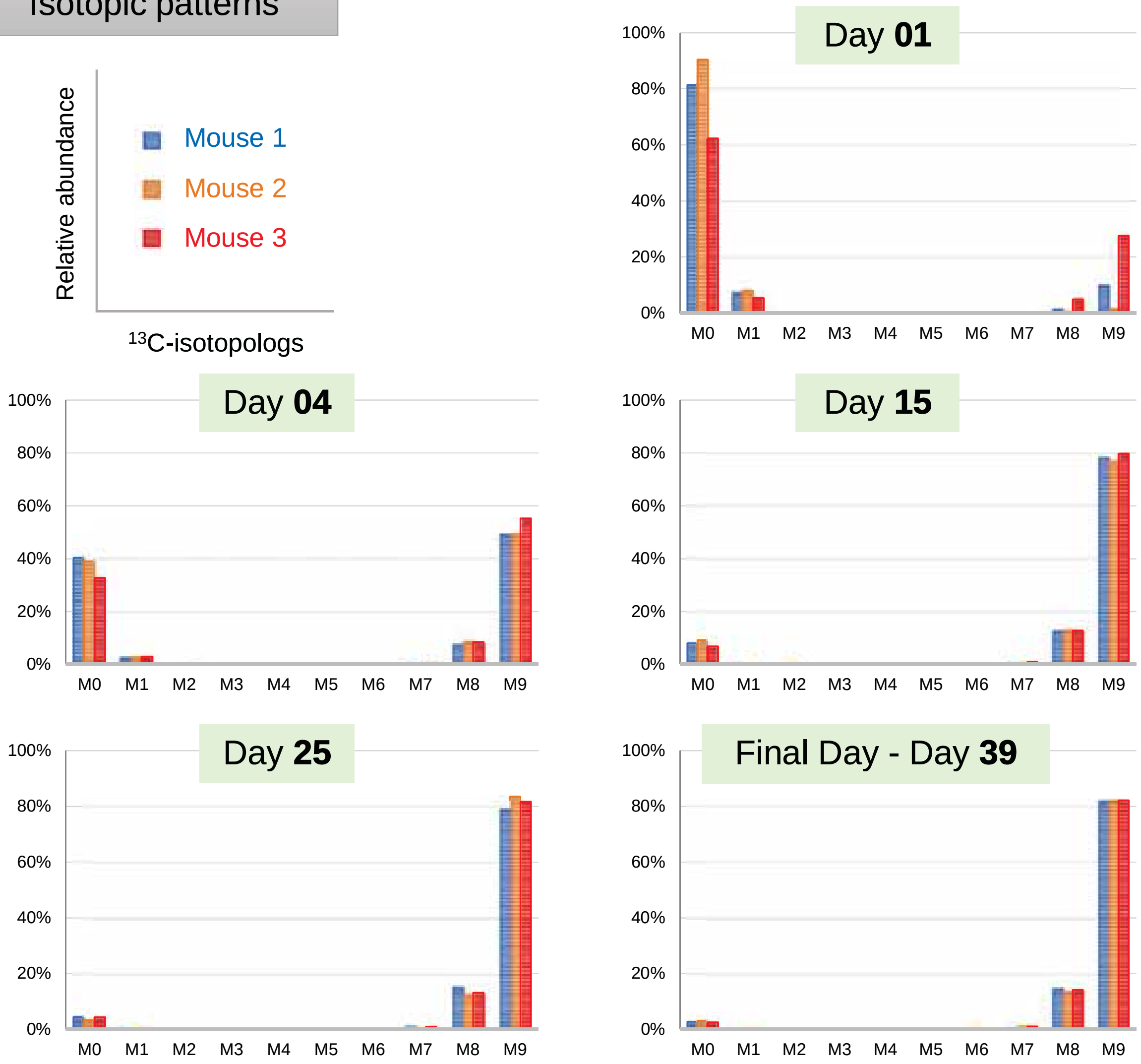

# Identified metabolites in murine urine

*Metabolites from the chemical library*

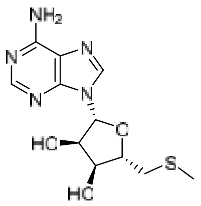


| | |
|---|---|
| **Metabolite** | **5-Deoxy-5-(methylthio)adenosine** |
| **Formula** | C11H15N5O3S |
| **Exact mass** | 297.0896 |

| | |
|---|---|
| **Ion type** | [M+H]+ |
| **m/z** | 298.0968 |

## Isotopic patterns

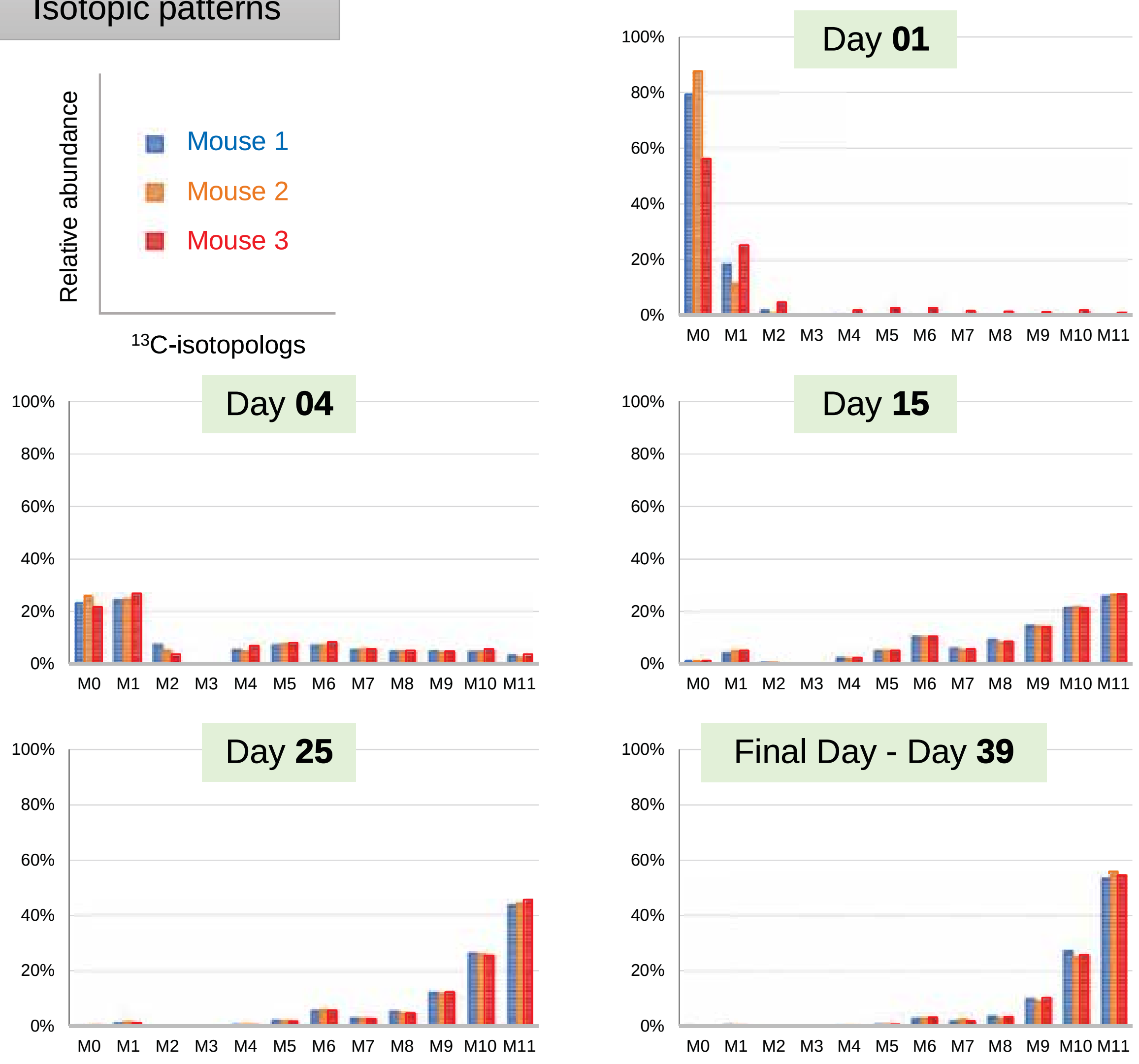


## Fractional $^{13}$C-enrichment

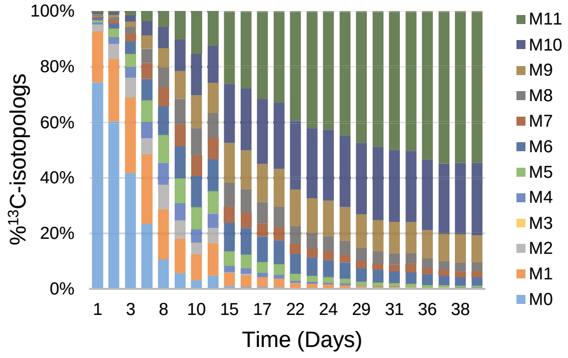


## $^{13}$C-enrichment kinetics

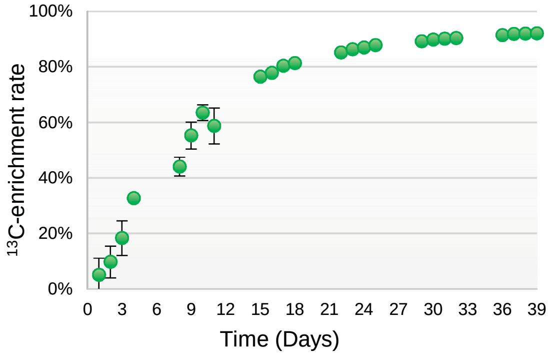

# Identified metabolites in murine urine

*Metabolites from the chemical library*

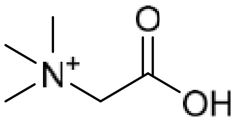


| Metabolite | Betaine |
|---|---|
| Formula | C5H11NO2 |
| Exact mass | 117.079 |

| Ion type | [M+H]+ |
|---|---|
| m/z | 118.0863 |

## Isotopic patterns

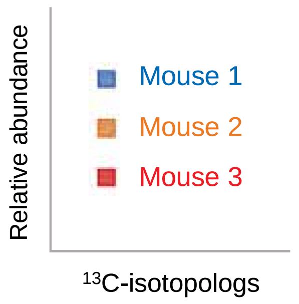


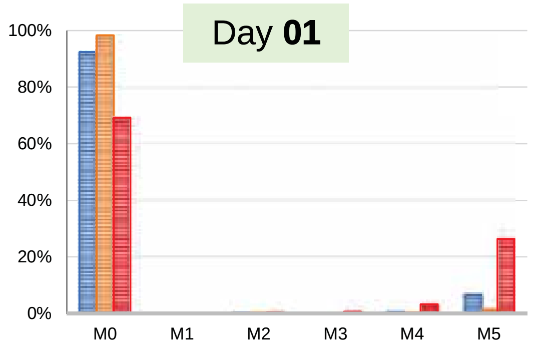


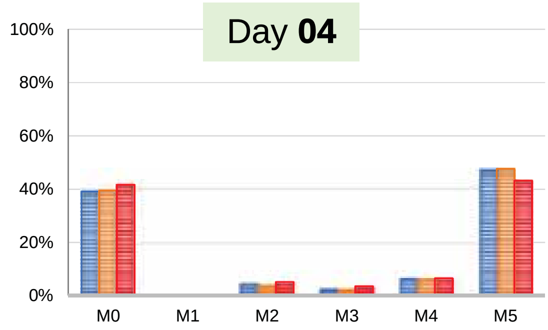


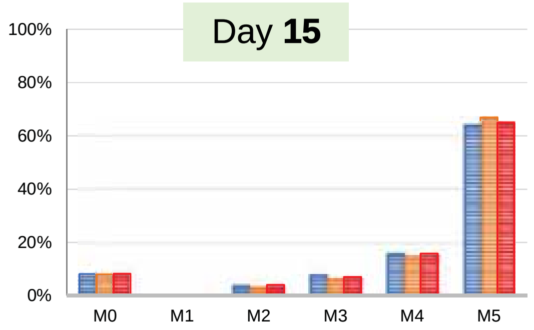


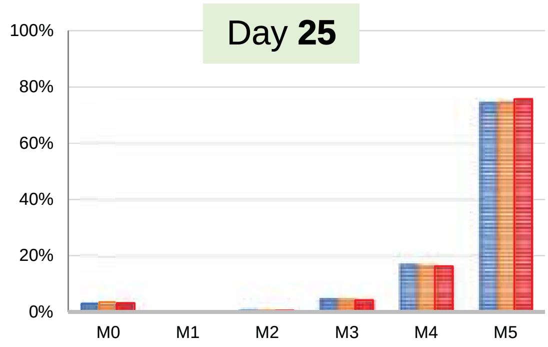


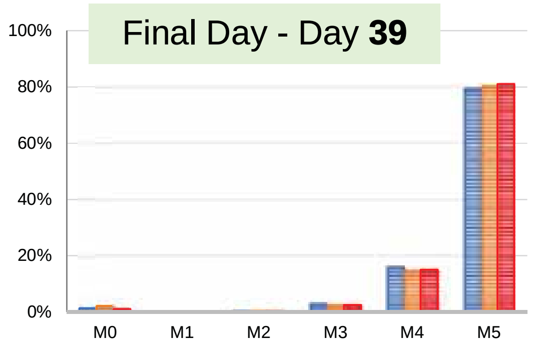


## Fractional $^{13}$C-enrichment

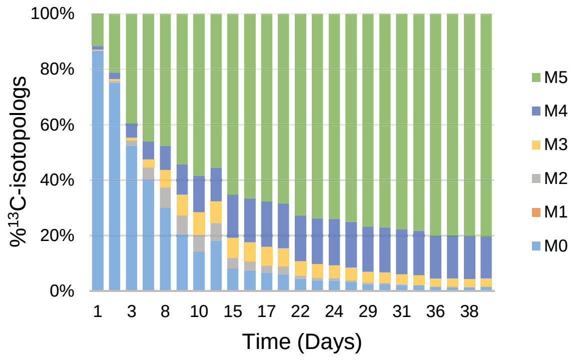


## $^{13}$C-enrichment kinetics

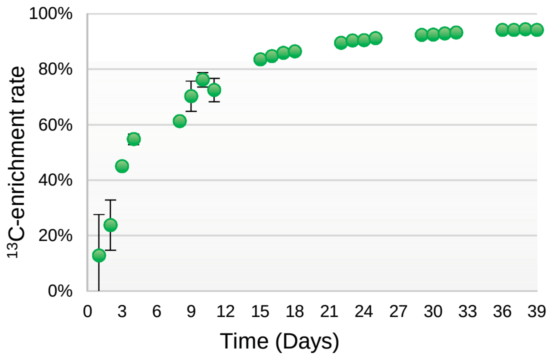

# Identified metabolites in murine urine

*Metabolites from the chemical library*

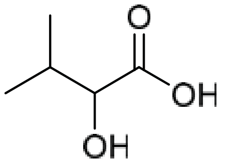


| Metabolite | 2-Hydroxy-3-methylbutyric acid |
|---|---|
| **Formula** | C5H10O3 |
| **Exact mass** | 118.063 |

| Ion type | [M-H]- |
|---|---|
| **m/z** | 117.0557 |

## Isotopic patterns

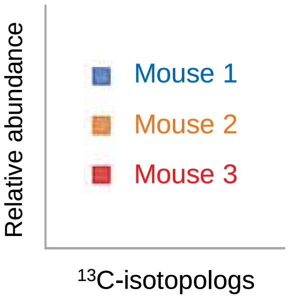


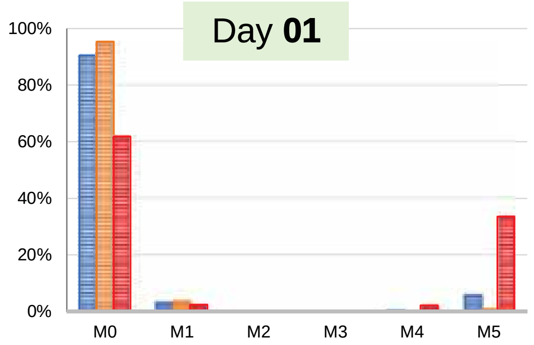


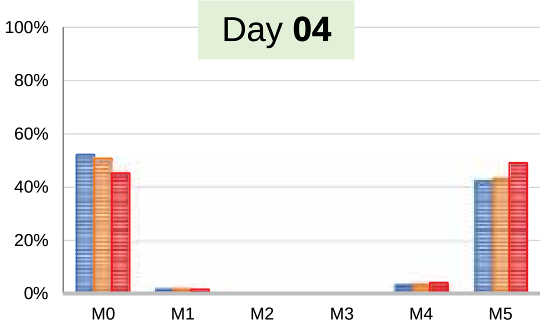


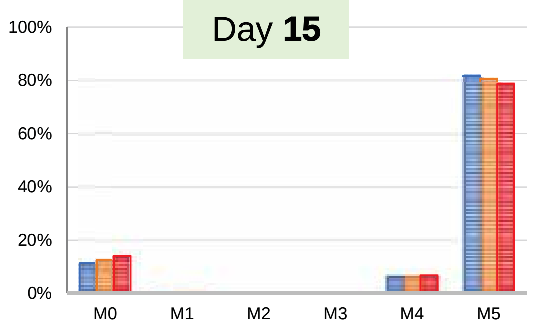


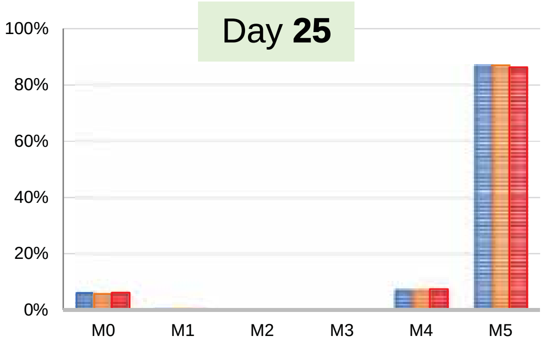


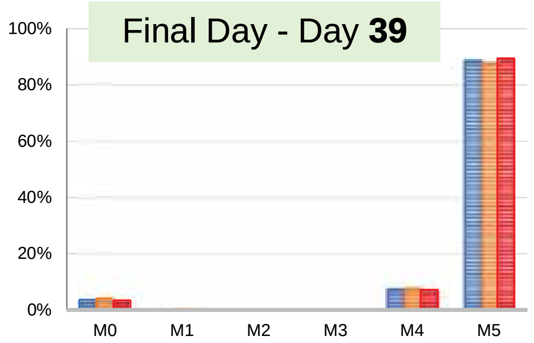


## Fractional $^{13}$C-enrichment

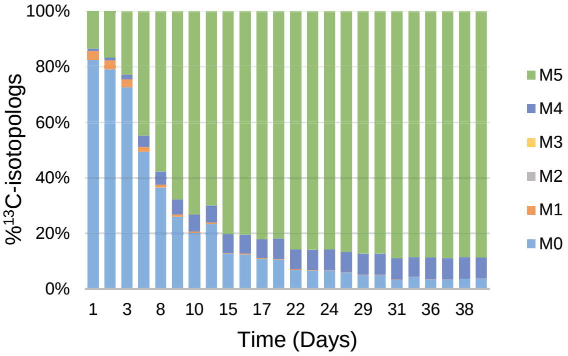


## $^{13}$C-enrichment kinetics

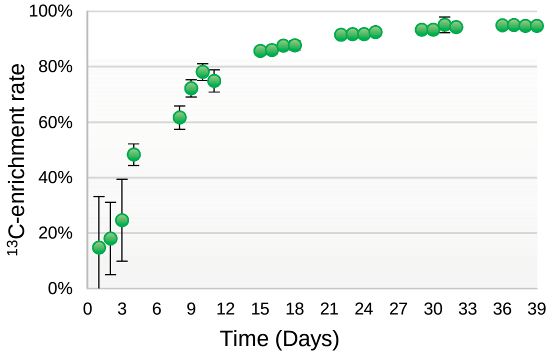

# Identified metabolites in murine urine

*Metabolites from the chemical library*

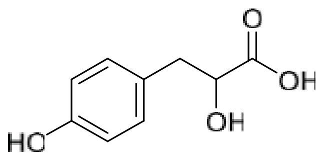


| **Metabolite** | **DL-p-Hydroxyphenyllactic acid** |
|---|---|
| **Formula** | C9H10O4 |
| **Exact mass** | 182.0579 |

| **Ion type** | [M-H]- |
|---|---|
| **m/z** | 181.0506 |

## Isotopic patterns

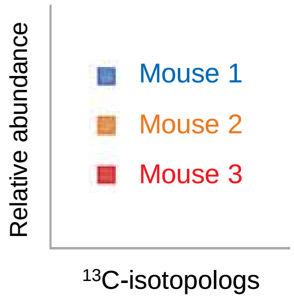


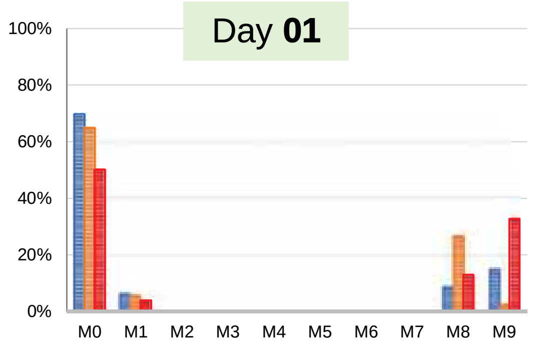


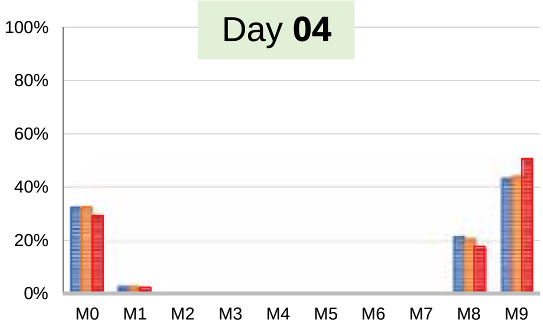


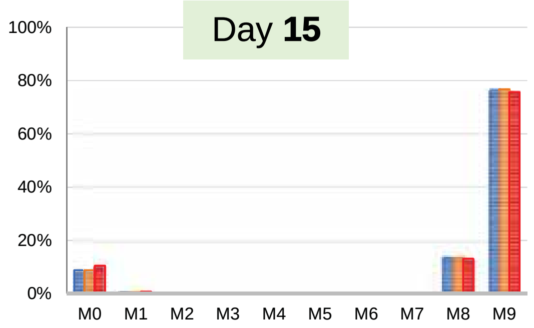


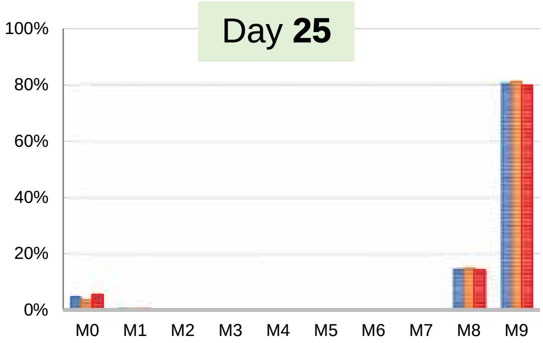


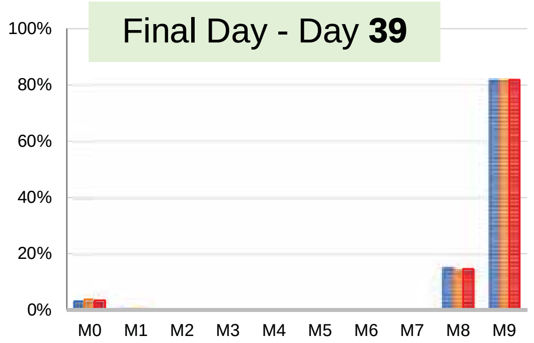


## Fractional $^{13}$C-enrichment

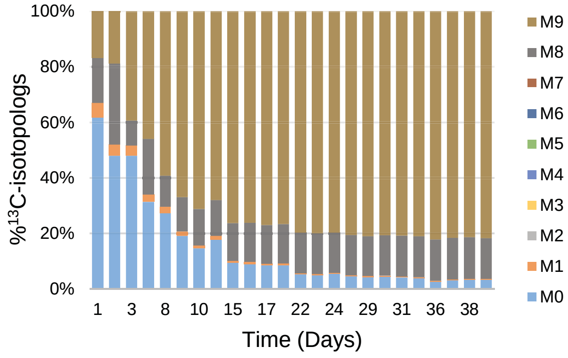


## $^{13}$C-enrichment kinetics

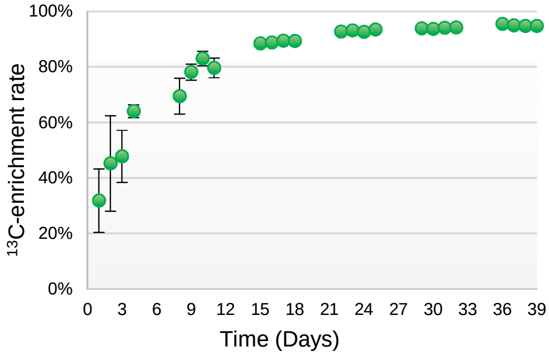

# Identified metabolites in murine urine

*Metabolites from the chemical library*

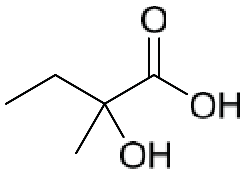


| Metabolite | 2-Hydroxy-2-methylbutyric acid |
|---|---|
| Formula | C5H10O3 |
| Exact mass | 118.063 |

| Ion type | [M-H]- |
|---|---|
| m/z | 117.0557 |

## Isotopic patterns

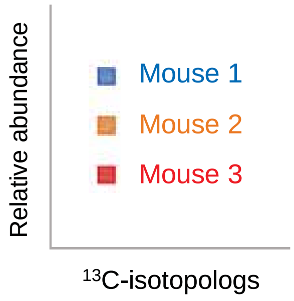


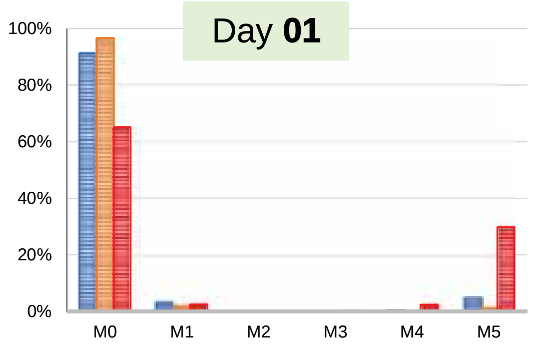


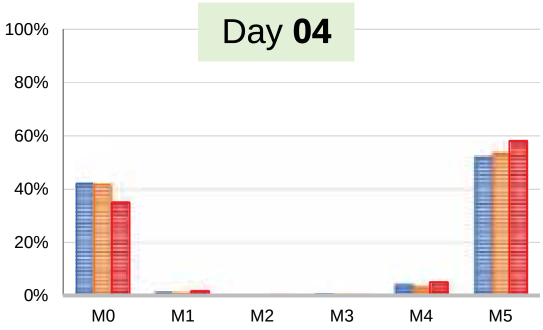


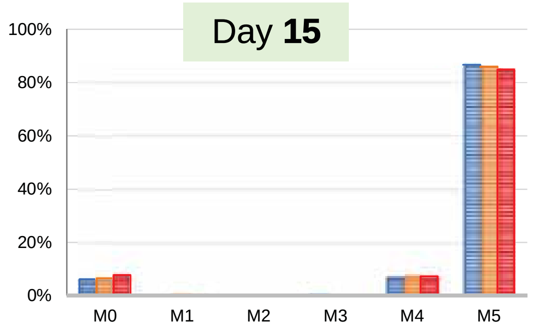


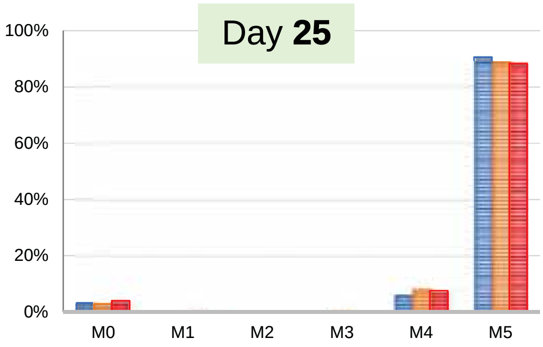


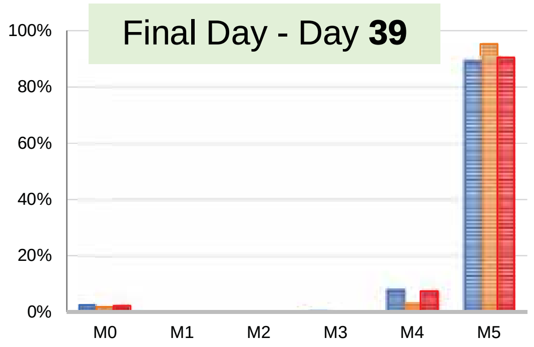

# Identified metabolites in murine urine

*Metabolites from the chemical library*

| Metabolite | 4-Pyridoxic acid |
|---|---|
| Formula | C8H9NO4 |
| Exact mass | 183.0532 |

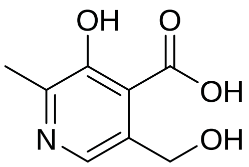


| Ion type | [M+H]+ |
|---|---|
| m/z | 184.0604 |

## Isotopic patterns

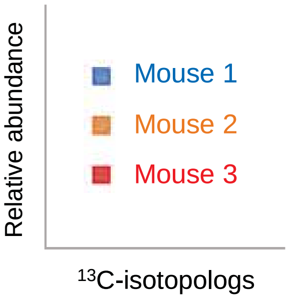


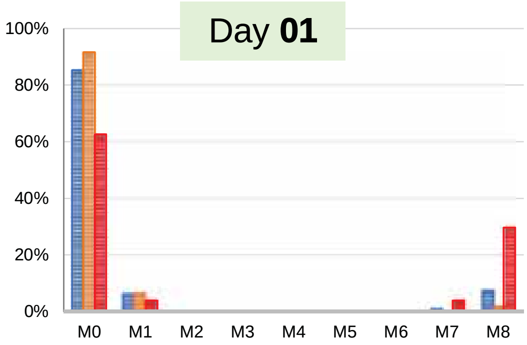


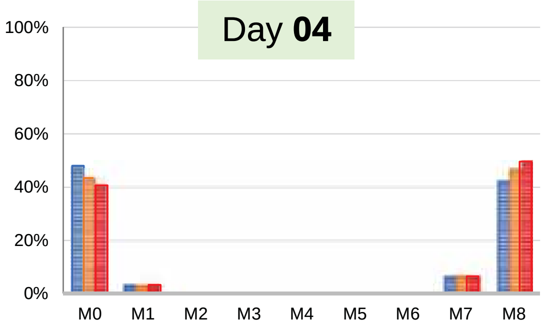


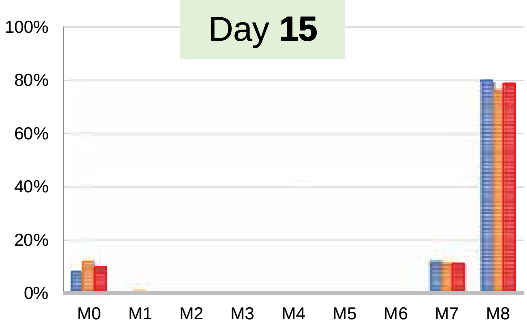


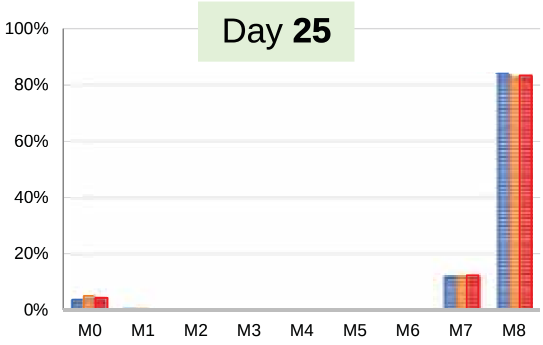


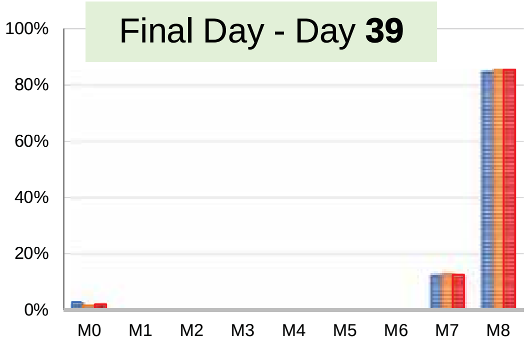


## Fractional $^{13}$C-enrichment

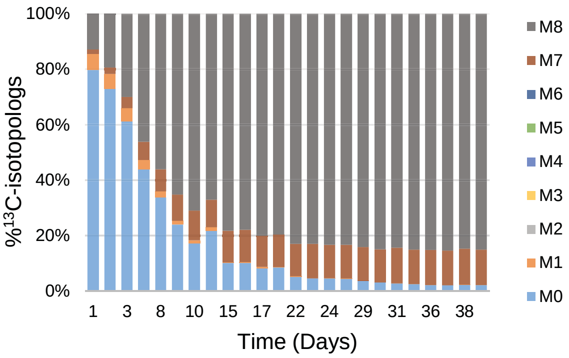


## $^{13}$C-enrichment kinetics

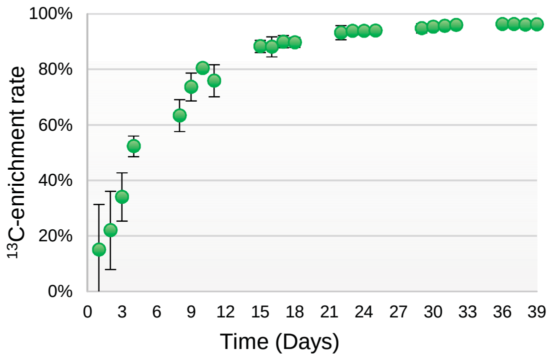

# Identified metabolites in murine urine

*Metabolites from the chemical library*

| Metabolite | Nicotinamide-N-oxide |
|---|---|
| **Formula** | C6H6N2O2 |
| **Exact mass** | 138.0429 |

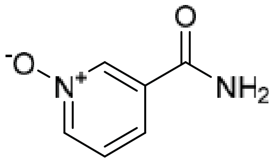

| Ion type | [M+H]+ |
|---|---|
| **m/z** | 139.0502 |

## Isotopic patterns

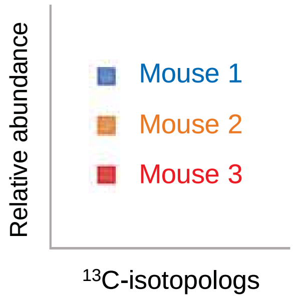


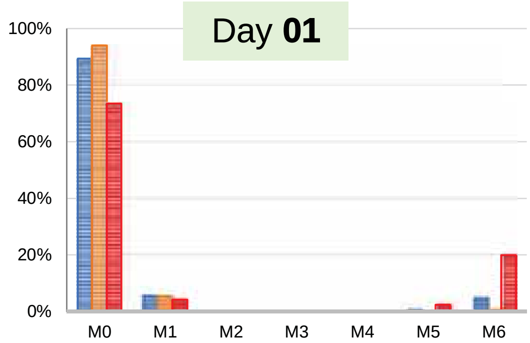


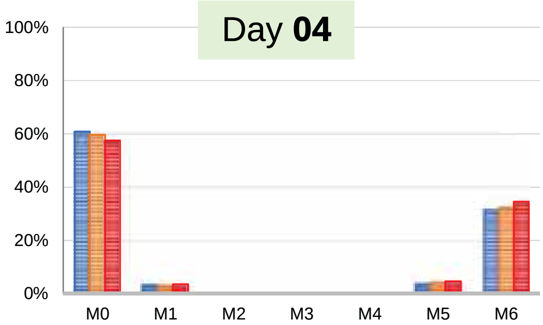


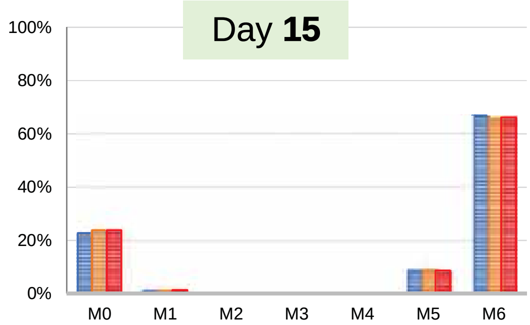


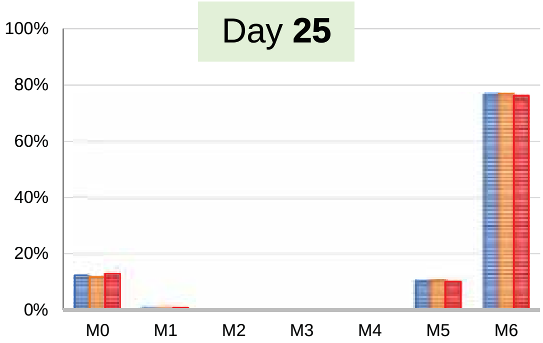


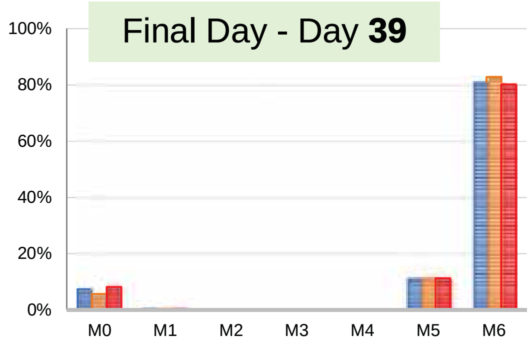


## Fractional $^{13}$C-enrichment

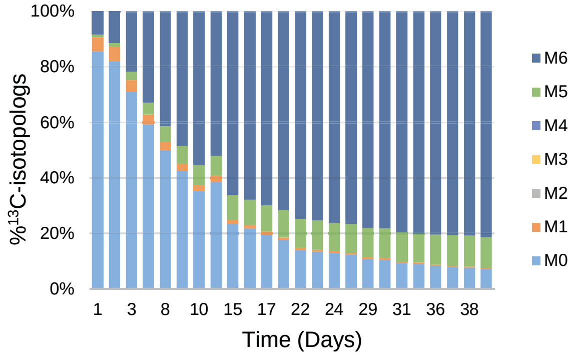


## $^{13}$C-enrichment kinetics

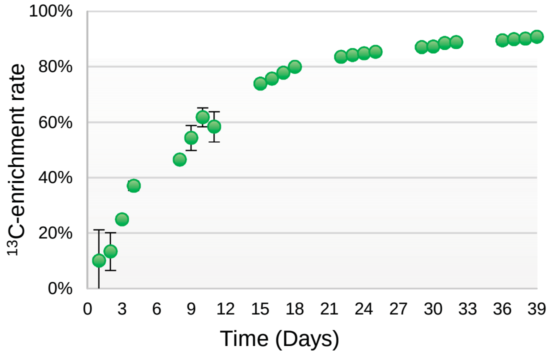

# Identified metabolites in murine urine

*Metabolites from the chemical library*

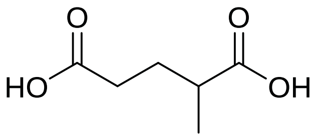


| **Metabolite** | **2-Methylglutaric acid** |
|---|---|
| **Formula** | C6H10O4 |
| **Exact mass** | 146.0579 |

| **Ion type** | [M-H]- |
|---|---|
| **m/z** | 145.0506 |

## Isotopic patterns

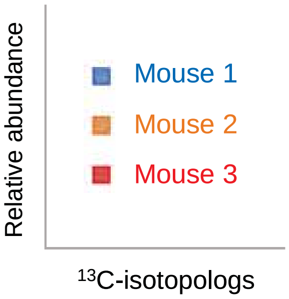


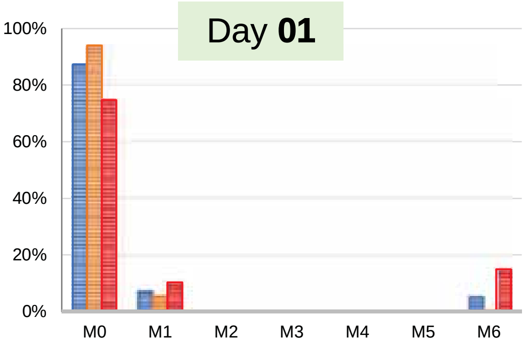


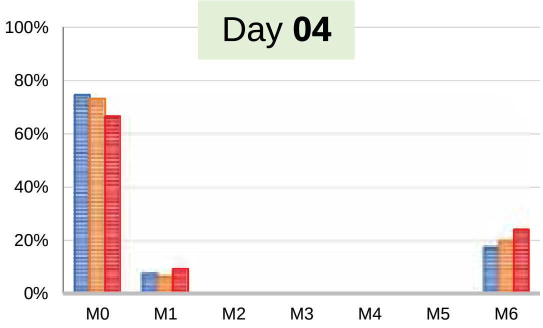


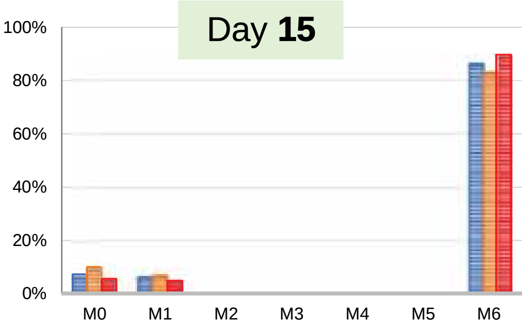


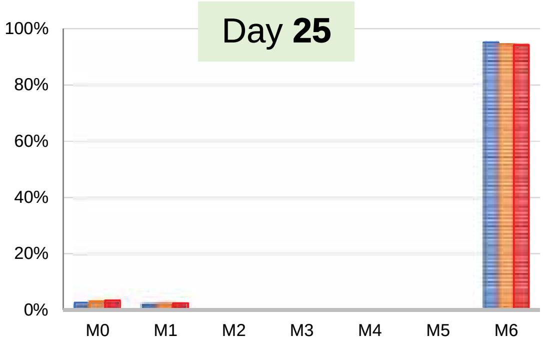


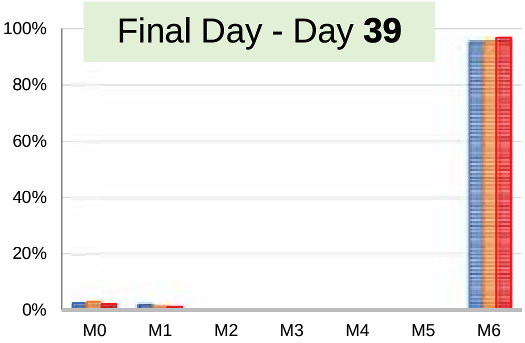


## Fractional $^{13}$C-enrichment

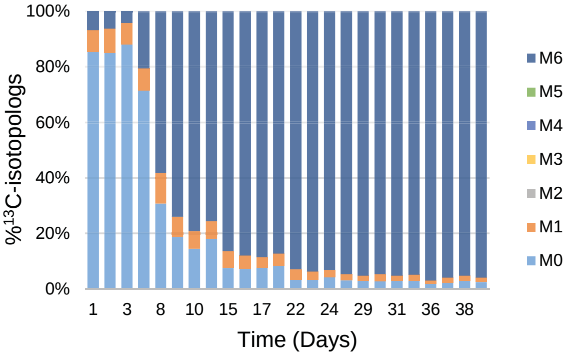


## $^{13}$C-enrichment kinetics

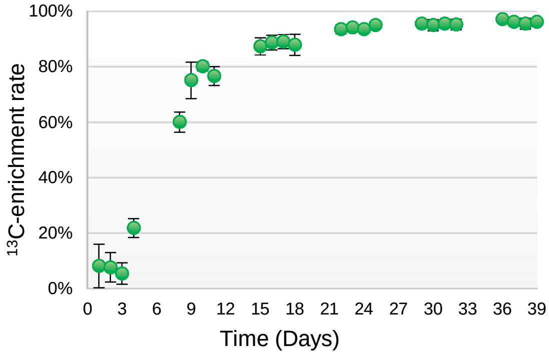

# Identified metabolites in murine urine

*Metabolites from the chemical library*

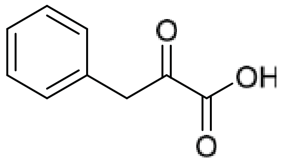


| | |
|---|---|
| **Metabolite** | **Phenylpyruvic acid** |
| **Formula** | C9H8O3 |
| **Exact mass** | 164.0473 |

| | |
|---|---|
| **Ion type** | [M-H]- |
| **m/z** | 163.0401 |

## Isotopic patterns

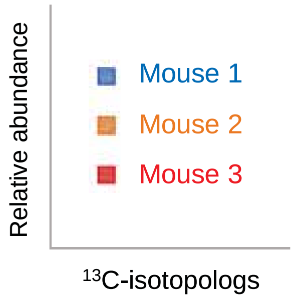


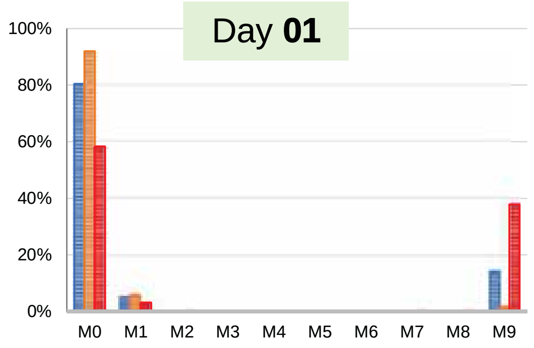


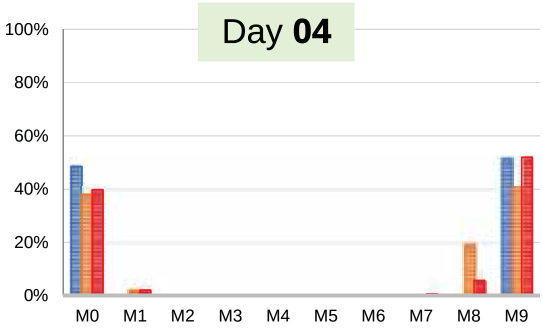


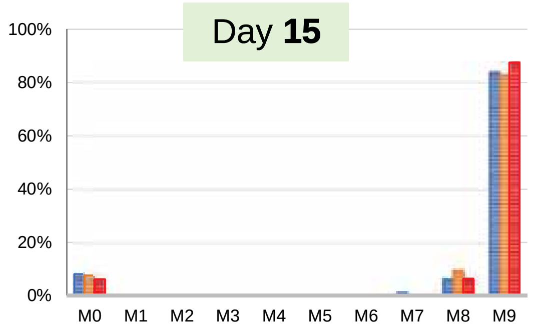


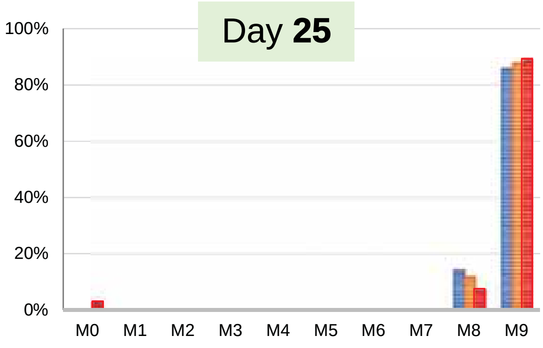


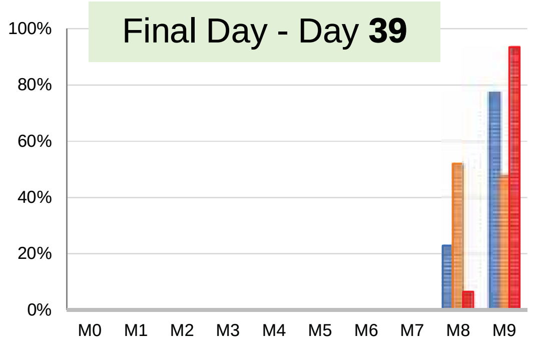

# Identified metabolites in murine urine

*Metabolites from the chemical library*

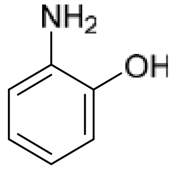


| **Metabolite** | **2-Aminophenol** |
|---|---|
| **Formula** | C6H7NO |
| **Exact mass** | 109.0528 |

| **Ion type** | [M+H]+ |
|---|---|
| **m/z** | 110.06 |

## Isotopic patterns

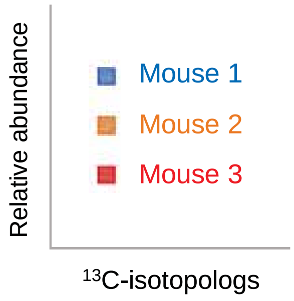


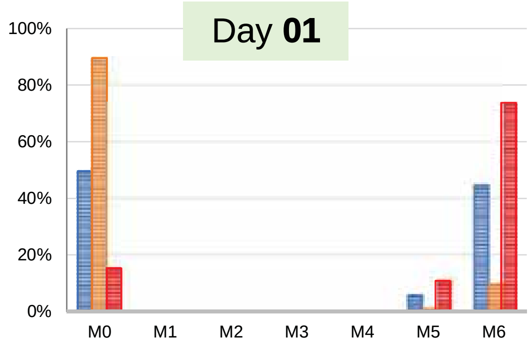


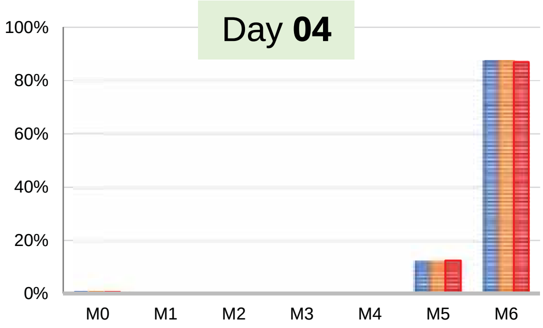


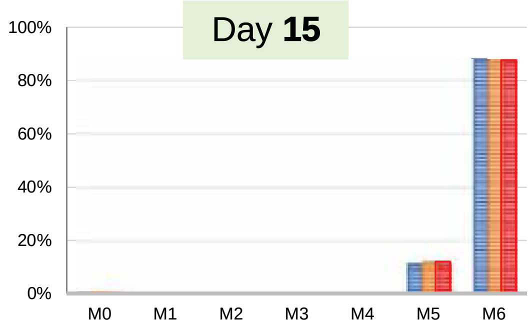


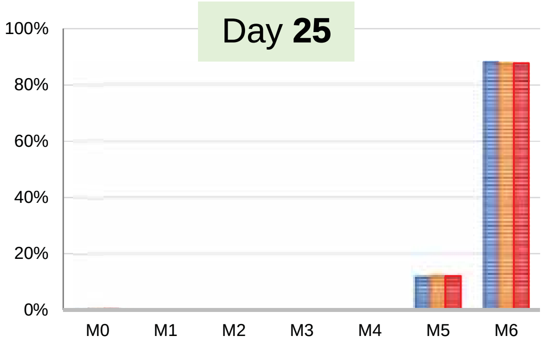


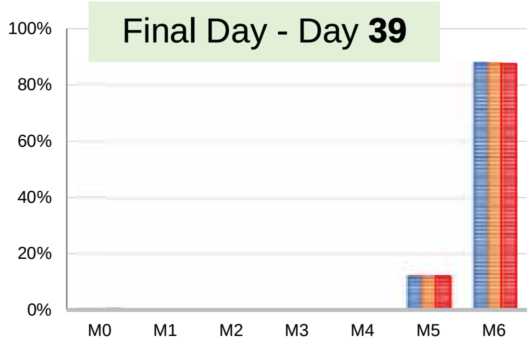


## Fractional $^{13}$C-enrichment

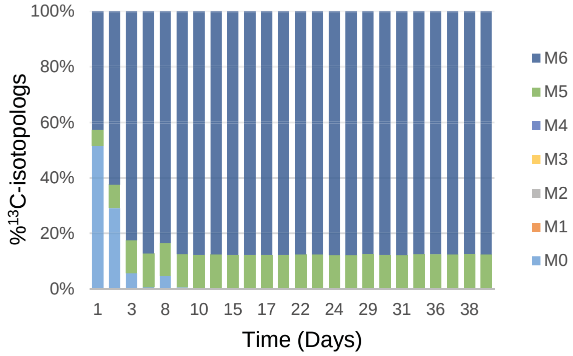


## $^{13}$C-enrichment kinetics

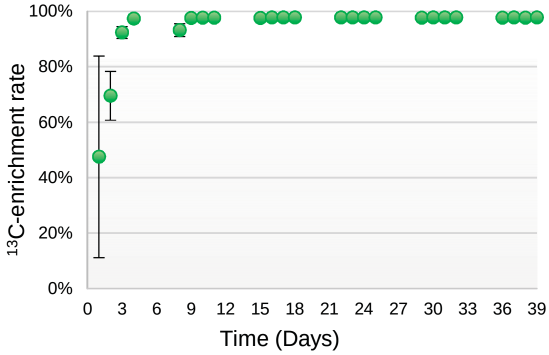

# Identified metabolites in murine urine

*Metabolites from the chemical library*

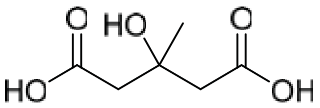


| | |
|---|---|
| **Metabolite** | **3-Hydroxy-3-methylglutaric acid** |
| **Formula** | C6H10O5 |
| **Exact mass** | 162.0528 |

| | |
|---|---|
| **Ion type** | [M-H]- |
| **m/z** | 161.0455 |

## Isotopic patterns

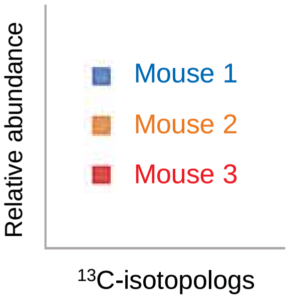


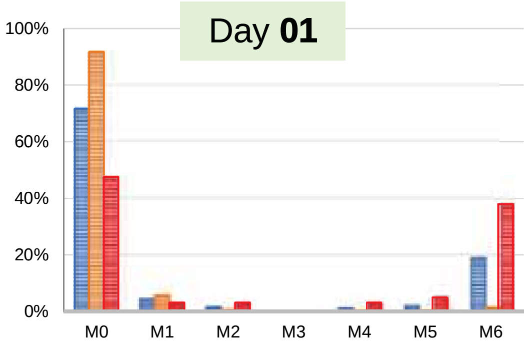


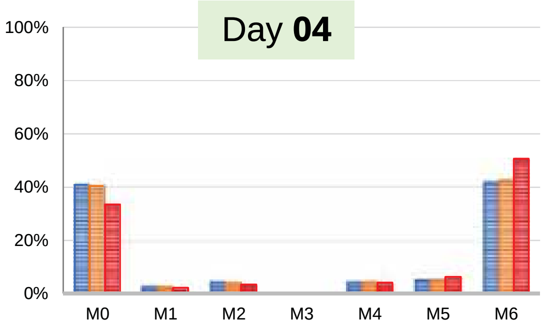


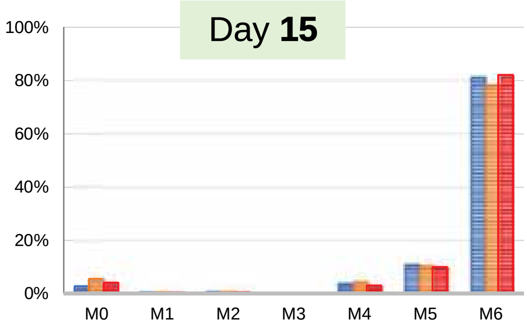


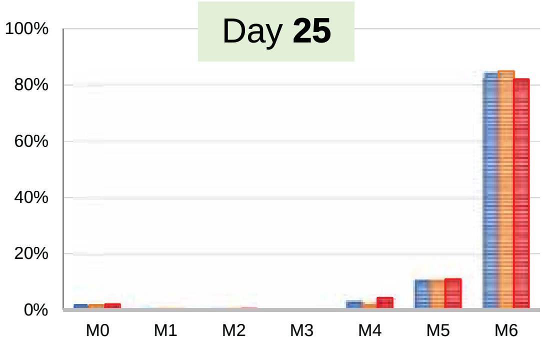


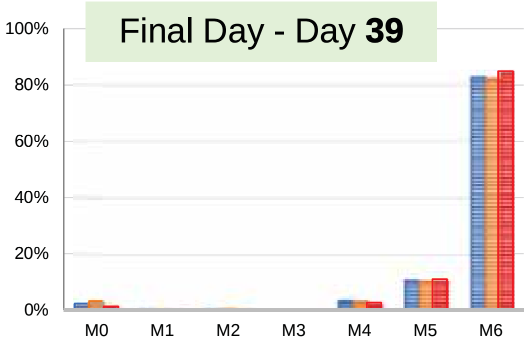


## Fractional $^{13}$C-enrichment

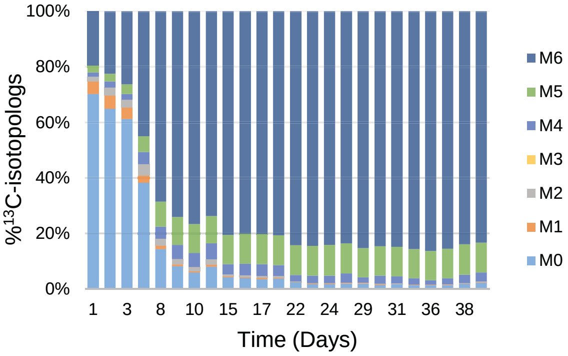


## $^{13}$C-enrichment kinetics

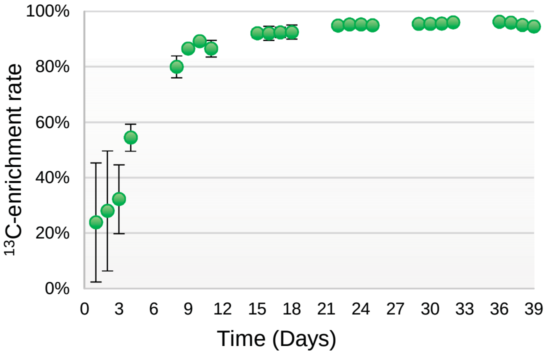

# Identified metabolites in murine urine

*Metabolites from the chemical library*

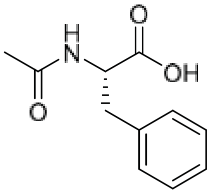


| Metabolite | N-Acetyl-L-phenylalanine |
|---|---|
| Formula | $C_{11}H_{13}NO_3$ |
| Exact mass | 207.0895 |

| Ion type | [M-H]- |
|---|---|
| m/z | 206.0823 |

## Isotopic patterns

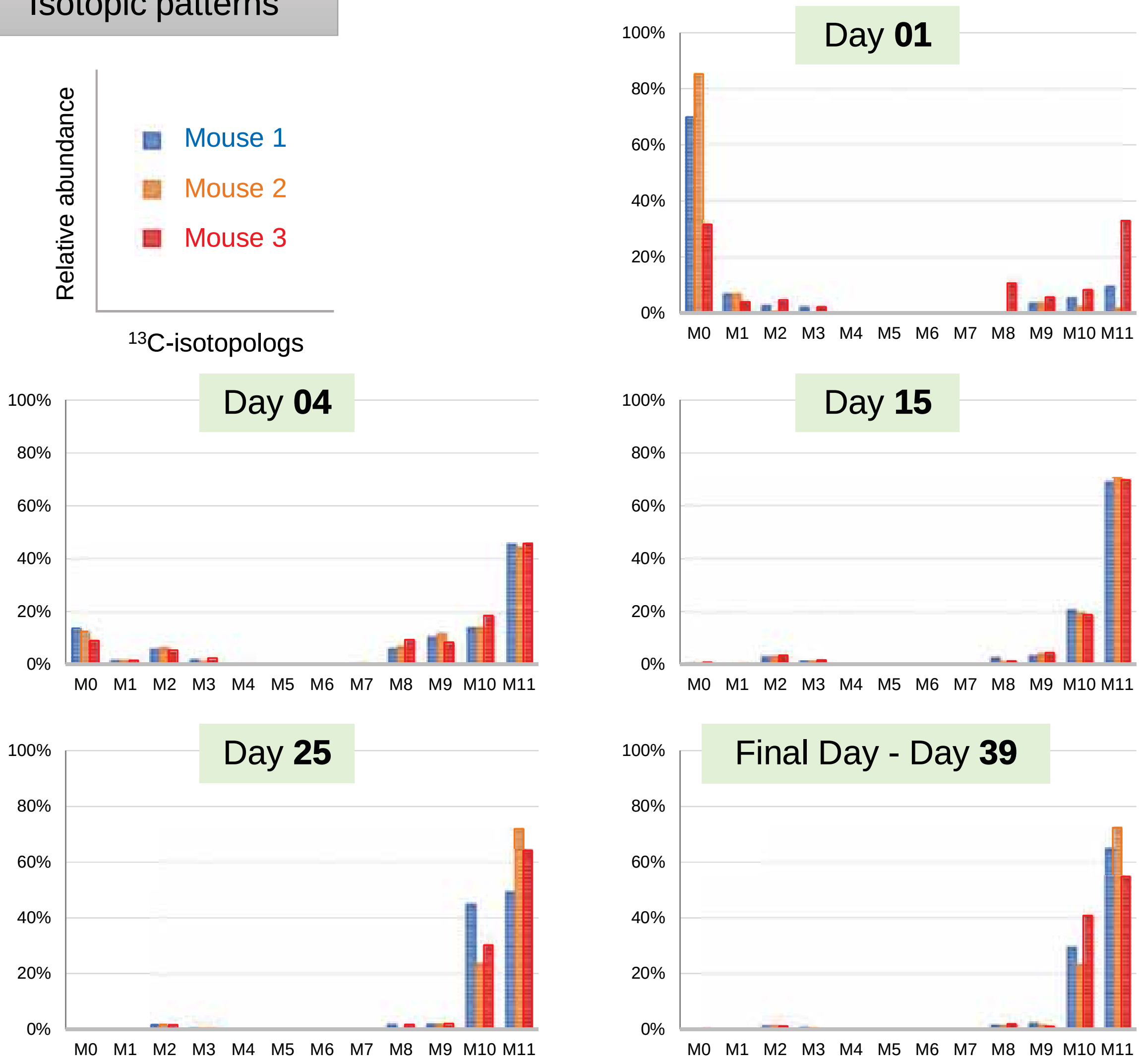

# Identified metabolites in murine urine

*Metabolites from the chemical library*

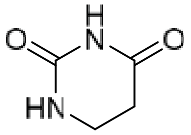

| Metabolite | Dihydrouracil |
|---|---|
| Formula | C4H6N2O2 |
| Exact mass | 114.0429 |

| Ion type | [M+H]+ |
|---|---|
| m/z | 115.0502 |

## Isotopic patterns

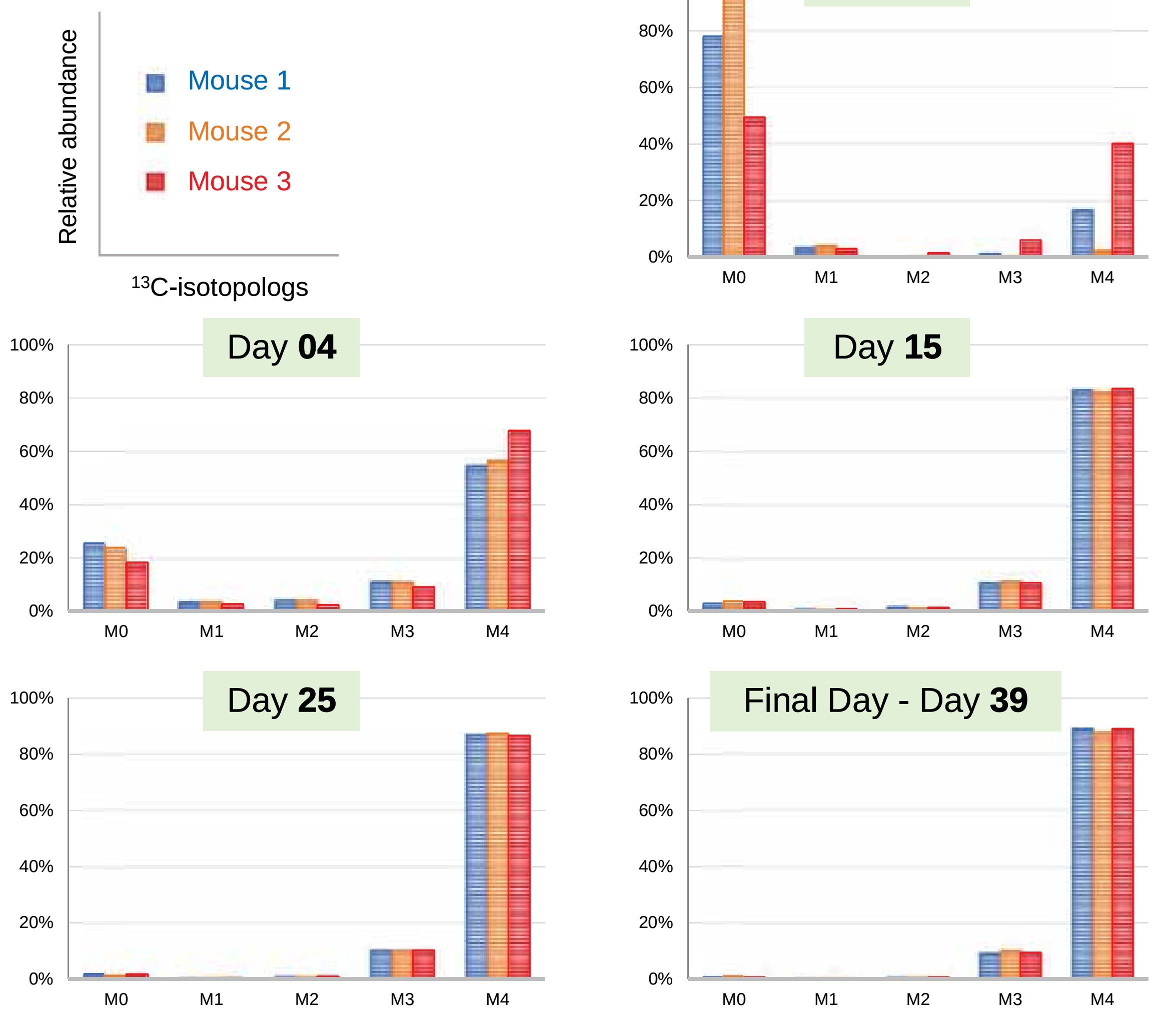


## Fractional 13C-enrichment

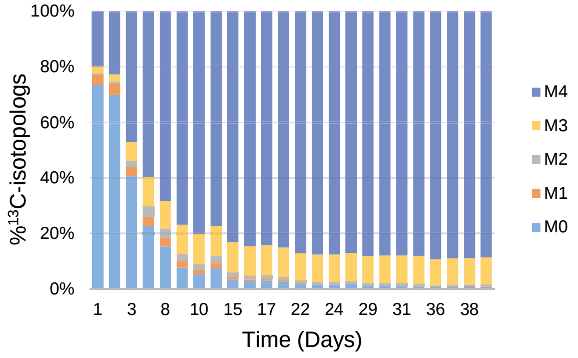


## 13C-enrichment kinetics

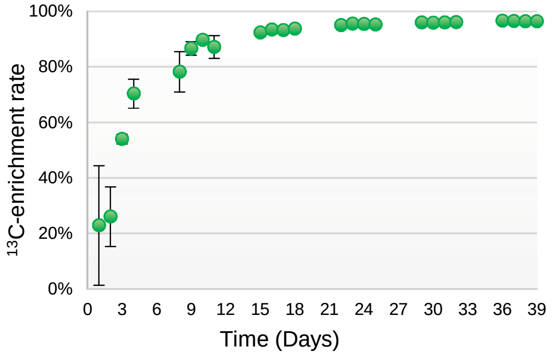

# Identified metabolites in murine urine

*Metabolites from the chemical library*

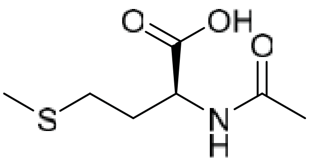


| Metabolite | N-Acetyl-L-methionine |
|---|---|
| **Formula** | C7H13NO3S |
| **Exact mass** | 191.0616 |

| Ion type | [M-H]- |
|---|---|
| **m/z** | 190.0543 |

## Isotopic patterns

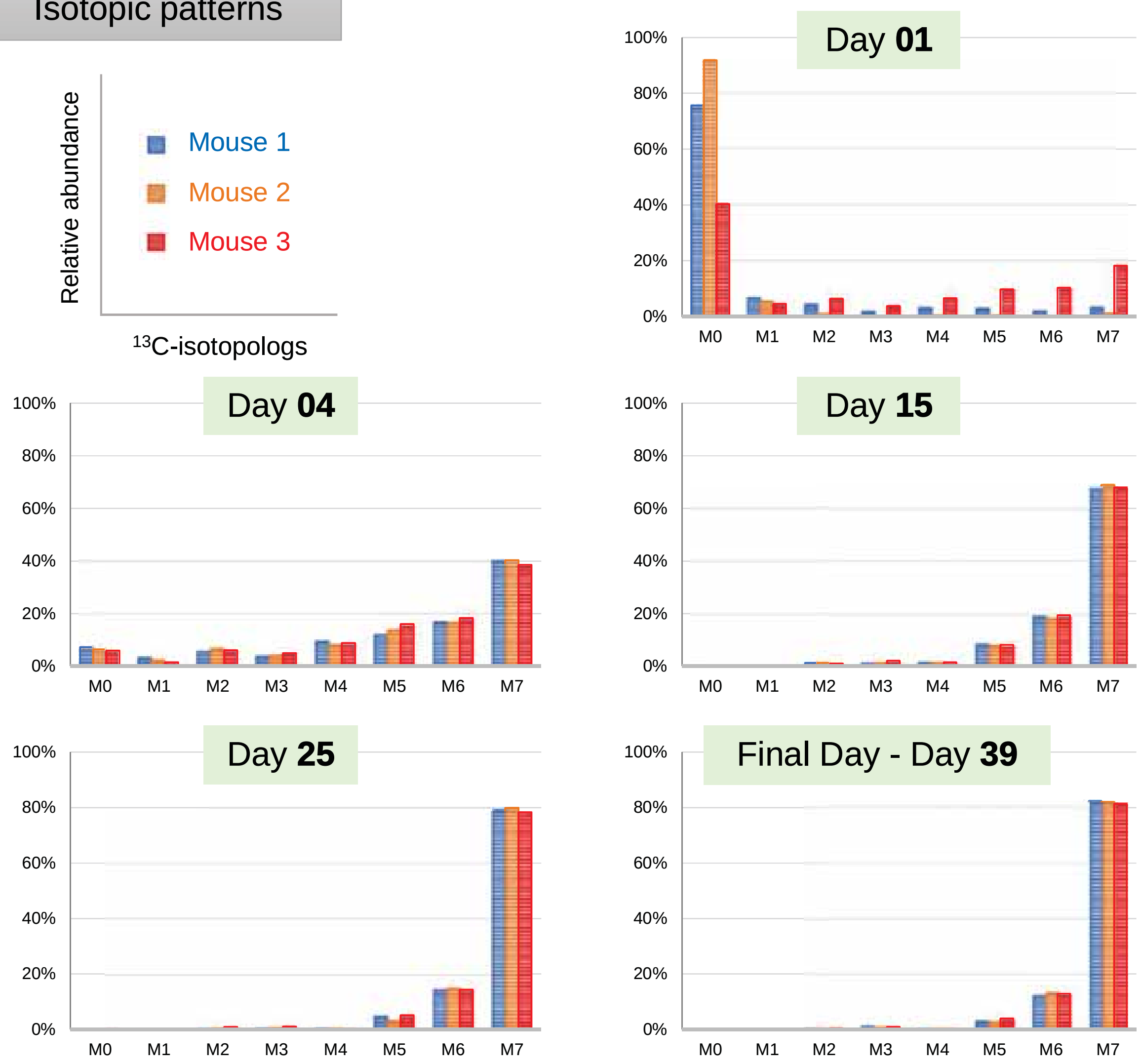

# Identified metabolites in murine urine

*Metabolites from the chemical library*

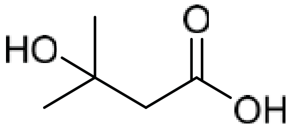


| Metabolite | 3-Hydroxy-3-methylbutyric acid |
|---|---|
| Formula | C5H10O3 |
| Exact mass | 118.063 |

| Ion type | [M-H]- |
|---|---|
| m/z | 117.0557 |

## Isotopic patterns

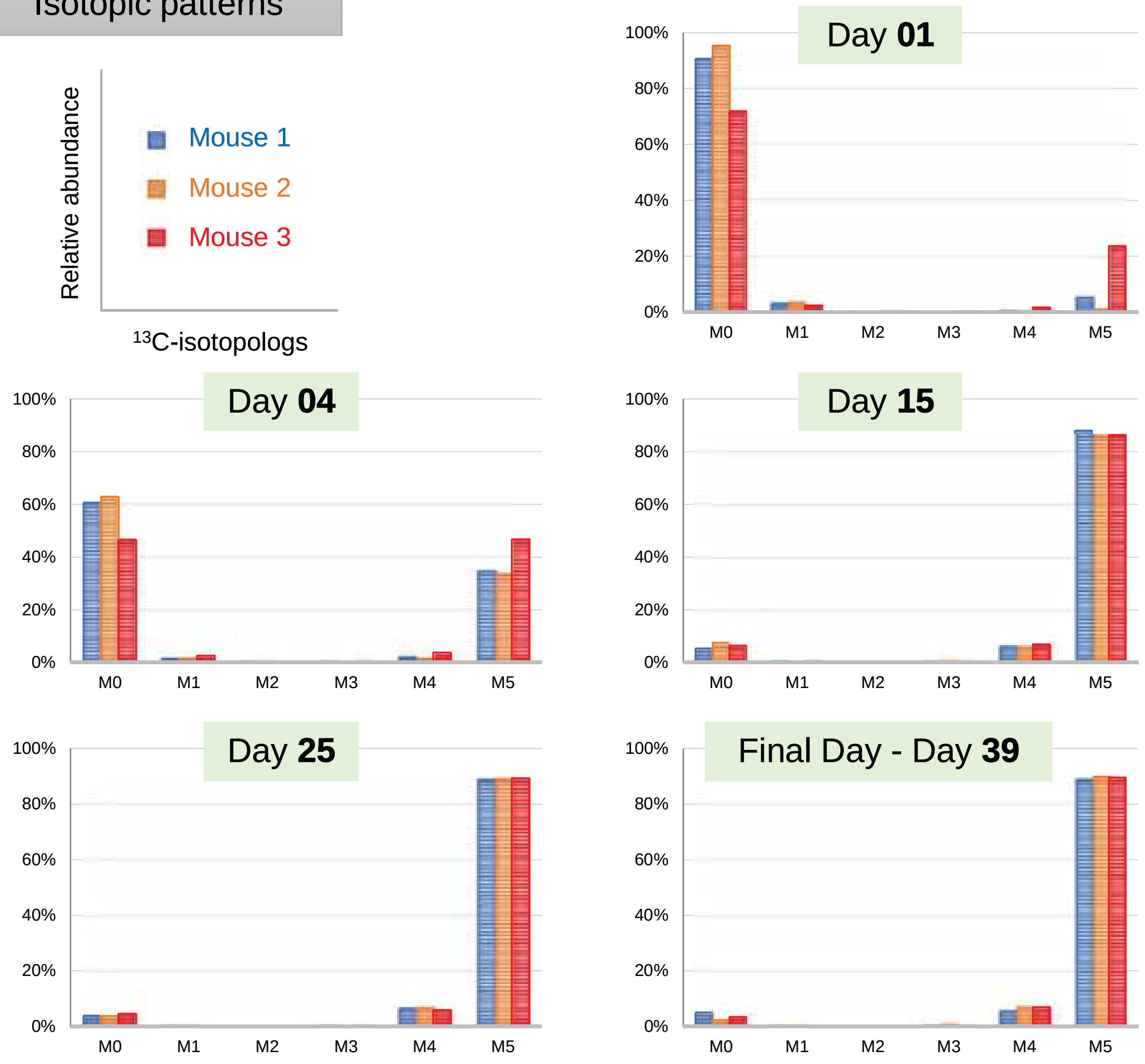

# Identified metabolites in murine urine

*Metabolites from the chemical library*

| Metabolite | Hypoxanthine |
|---|---|
| Formula | C5H4N4O |
| Exact mass | 136.0385 |

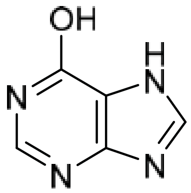


| Ion type | [M+H]+ |
|---|---|
| m/z | 137.0458 |

## Isotopic patterns

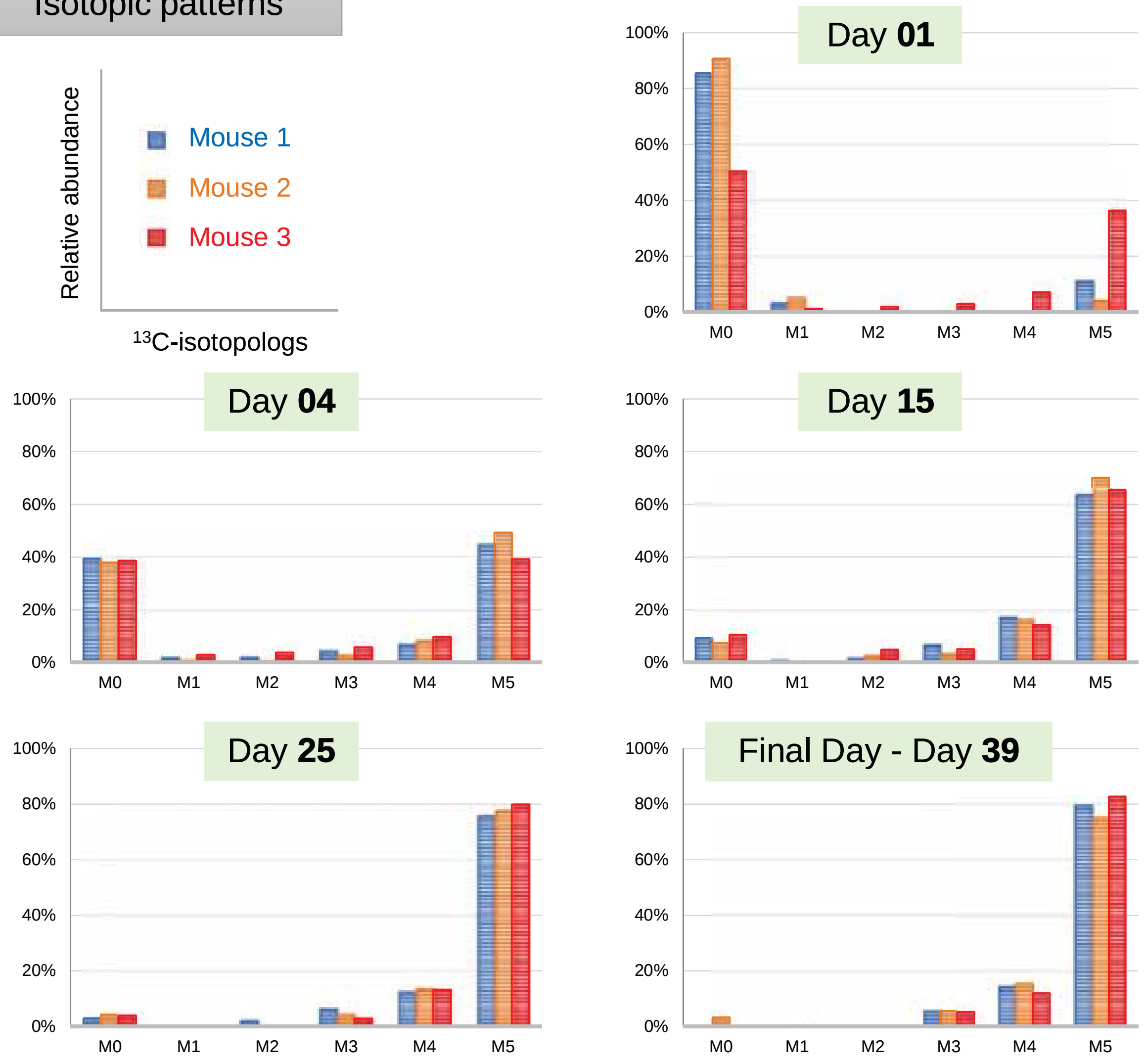

# Identified metabolites in murine urine

*Metabolites from the chemical library*

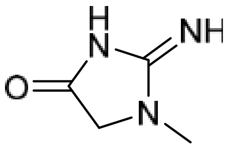


| Metabolite | Creatinine |
|---|---|
| Formula | C4H7N3O |
| Exact mass | 113.0589 |

| Ion type | [M+H]+ |
|---|---|
| m/z | 114.0662 |

## Isotopic patterns

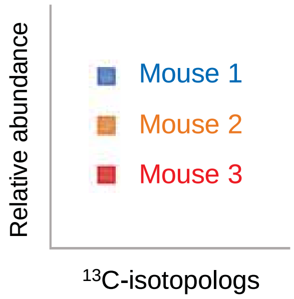


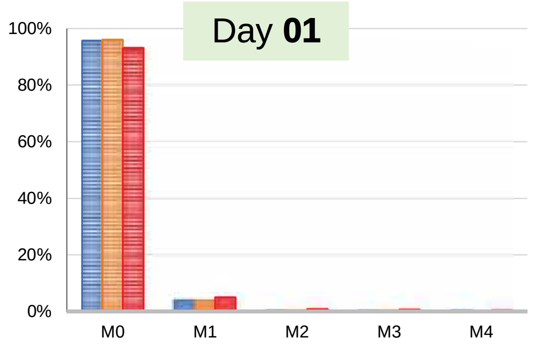


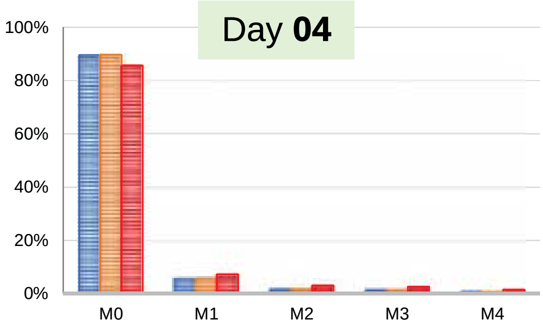


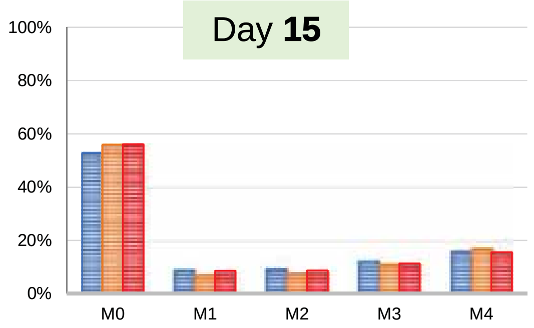


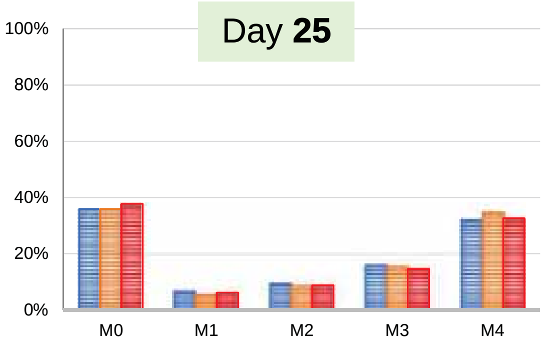


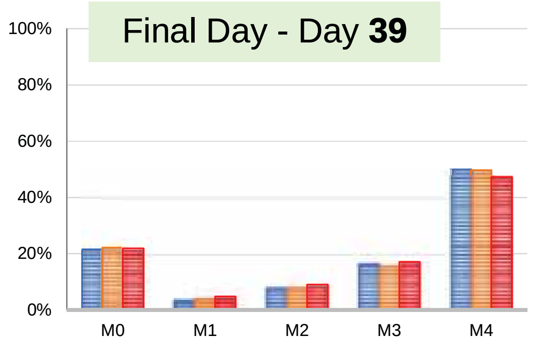


## Fractional $^{13}$C-enrichment

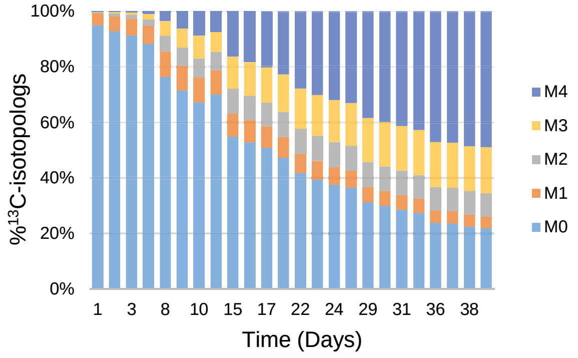


## $^{13}$C-enrichment kinetics

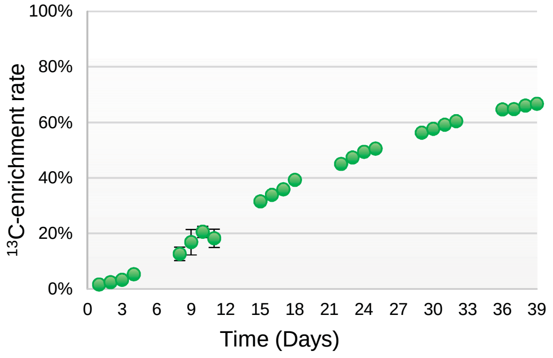

# Identified metabolites in murine urine

*Metabolites from the chemical library*

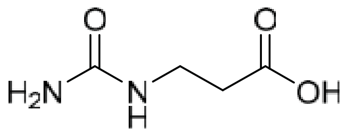


| Metabolite | 3-Ureidopropionic acid |
|---|---|
| Formula | C4H8N2O3 |
| Exact mass | 132.0535 |

| Ion type | [M-H]- |
|---|---|
| m/z | 131.0462 |

## Isotopic patterns

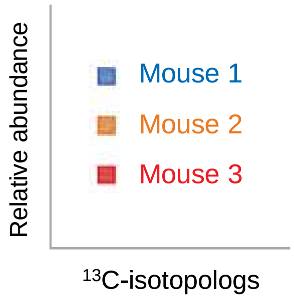


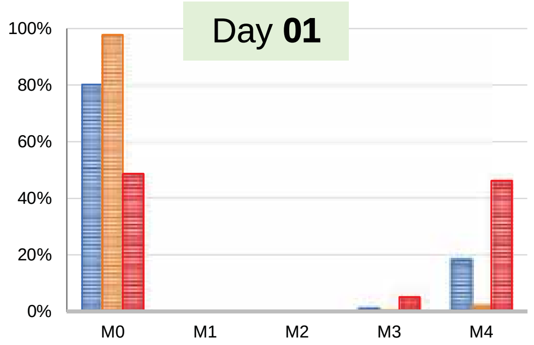


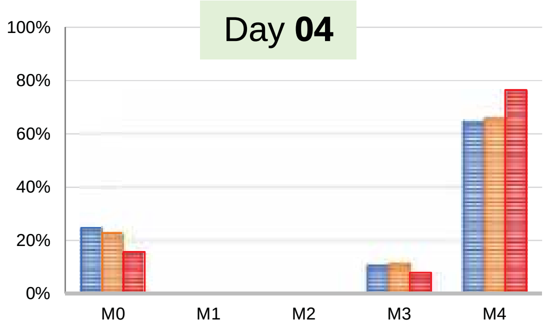


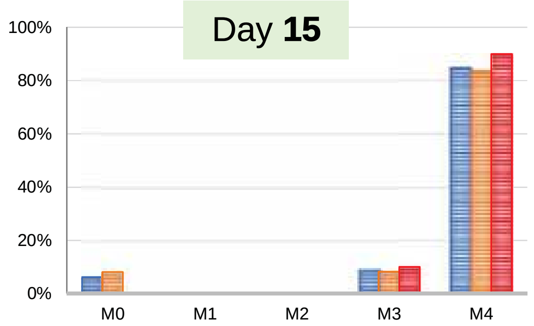


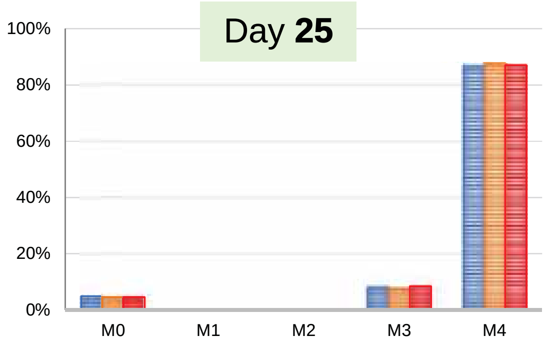


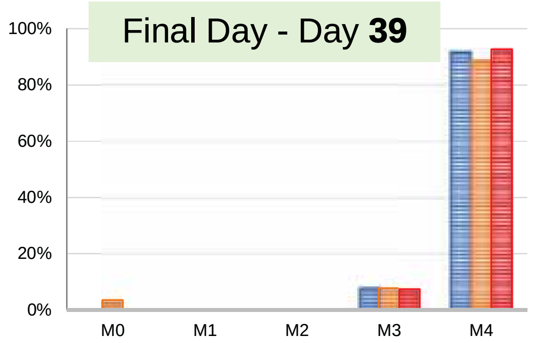


## Fractional $^{13}$C-enrichment

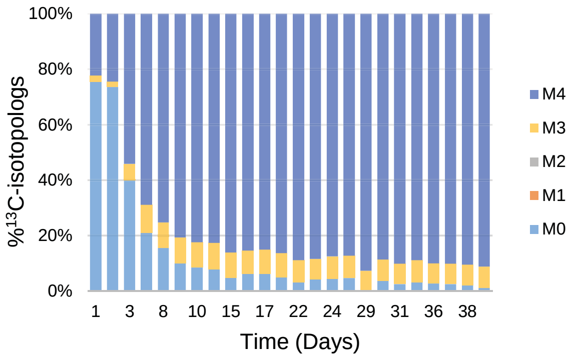


## $^{13}$C-enrichment kinetics

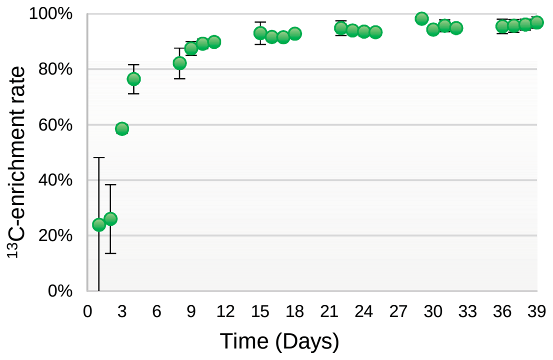

# Identified metabolites in murine urine

*Metabolites from the chemical library*

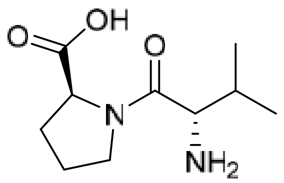


| Metabolite | Val-Pro |
|---|---|
| Formula | C10H18N2O3 |
| Exact mass | 214.1317 |

| Ion type | [M+H]+ |
|---|---|
| m/z | 215.139 |

## Isotopic patterns

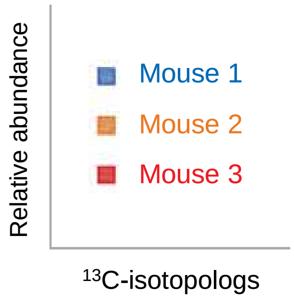


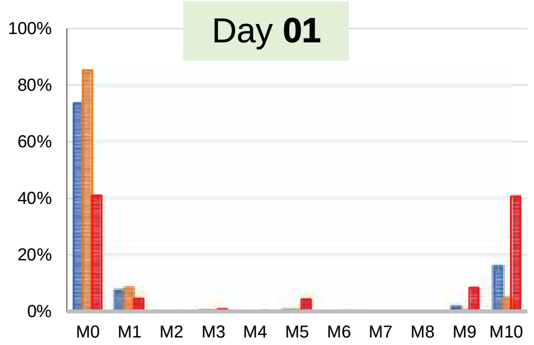


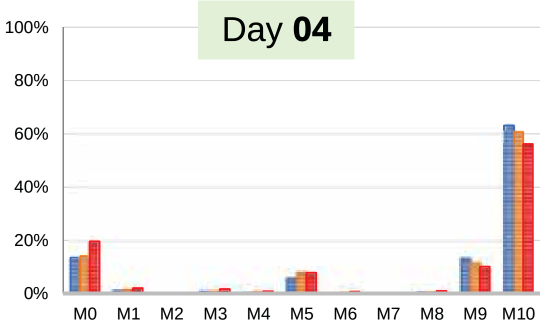


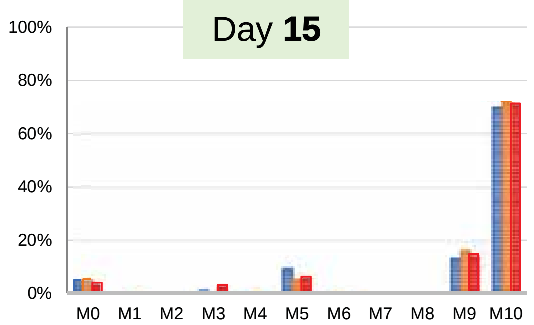


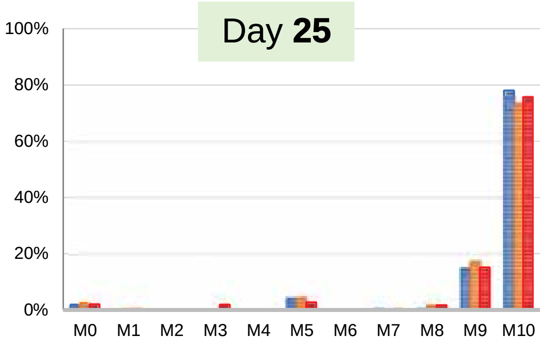


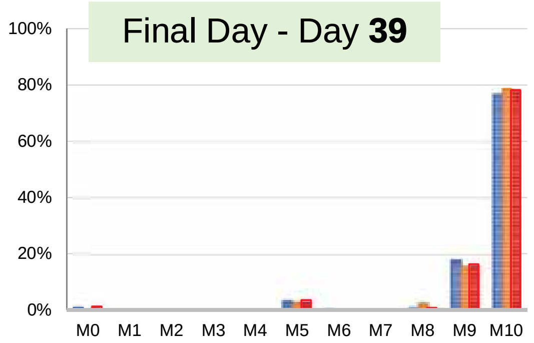

# Identified metabolites in murine urine

*Metabolites from the chemical library*

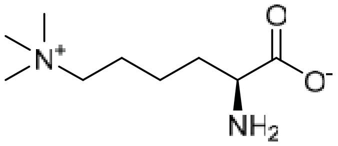


| | |
|---|---|
| **Metabolite** | **N6-N6-N6-Trimethyl-L-lysine** |
| **Formula** | C9H20N2O2 |
| **Exact mass** | 188.1525 |

| | |
|---|---|
| **Ion type** | [M+H]+ |
| **m/z** | 189.1598 |

## Isotopic patterns

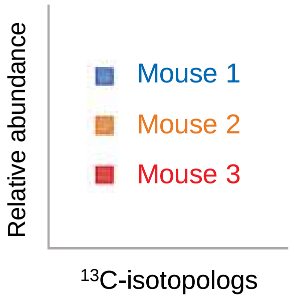


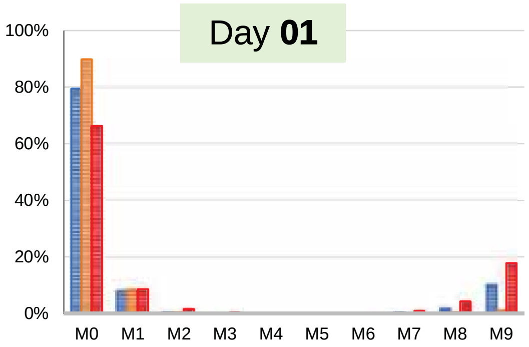


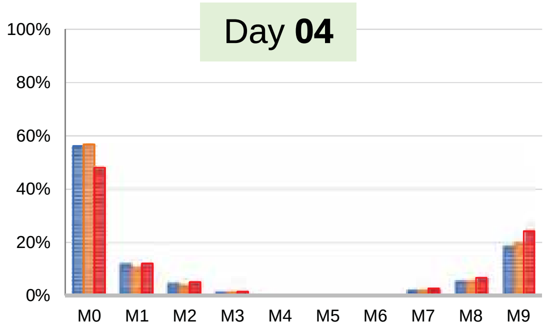


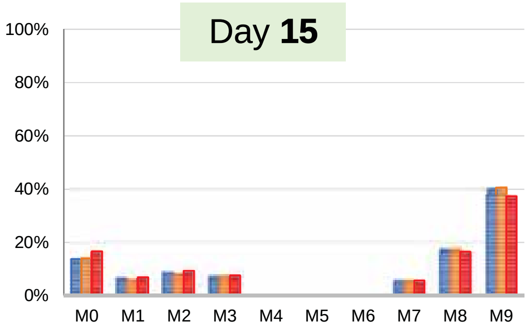


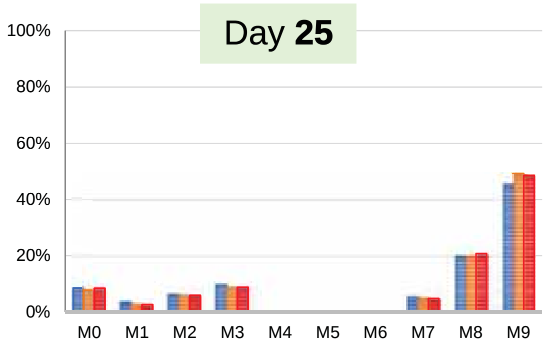


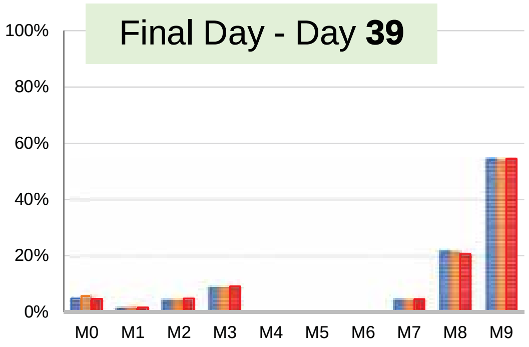


## Fractional $^{13}$C-enrichment

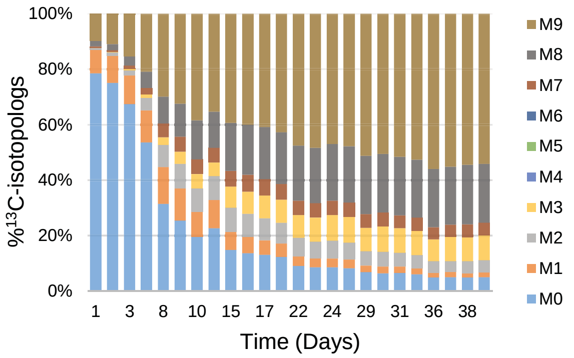


## $^{13}$C-enrichment kinetics

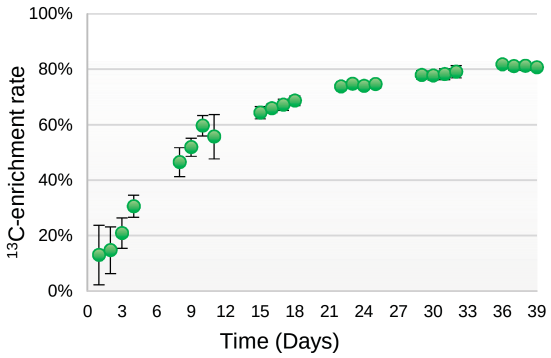

# Identified metabolites in murine urine

*Metabolites from the chemical library*

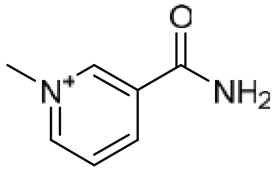


| **Metabolite** | **1-Methylnicotinamide** |
|---|---|
| **Formula** | C7H8N2O |
| **Exact mass** | 136.0637 |

| **Ion type** | [M+H]+ |
|---|---|
| **m/z** | 137.0709 |

## Isotopic patterns

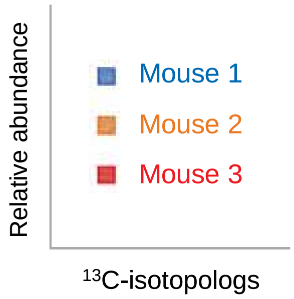


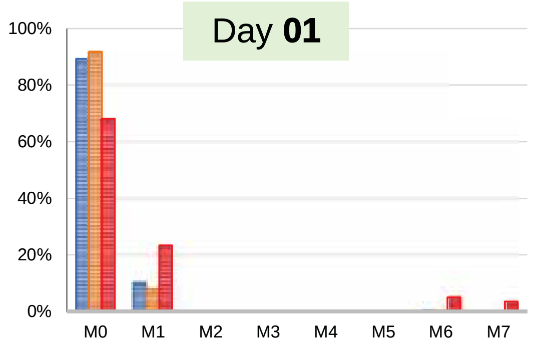


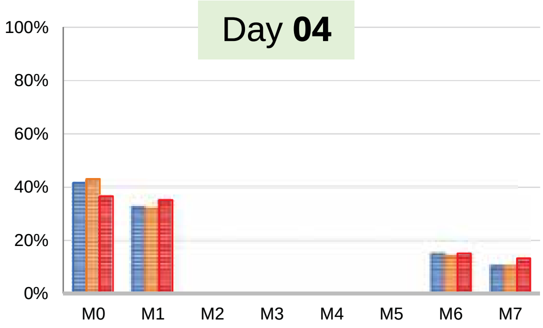


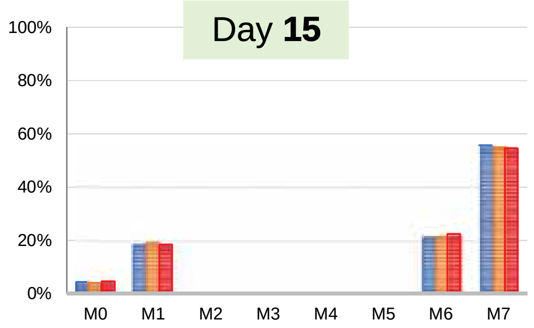


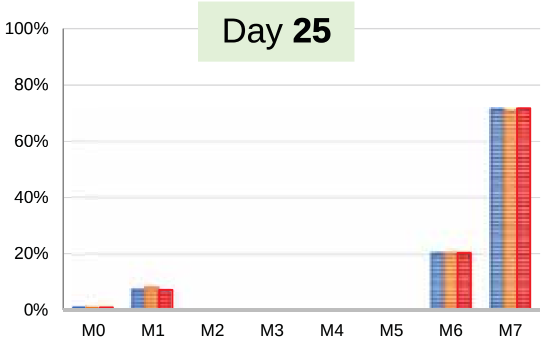


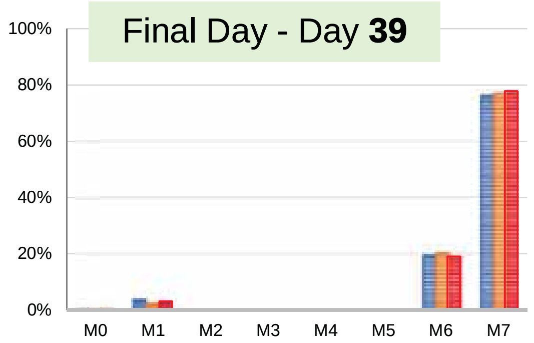


## Fractional $^{13}$C-enrichment

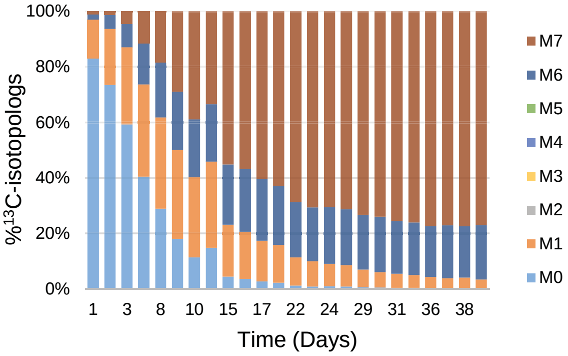


## $^{13}$C-enrichment kinetics

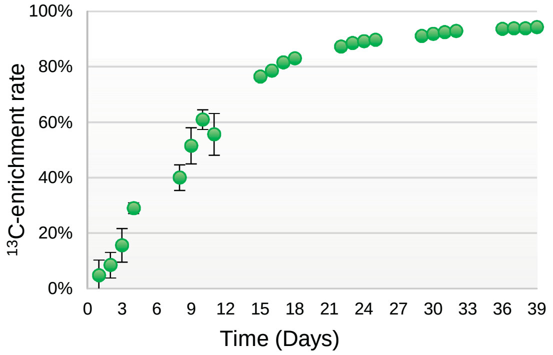

# Identified metabolites in murine urine

*Metabolites from the chemical library*

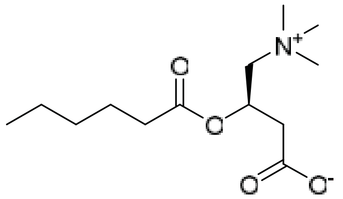

| | |
|---|---|
| **Metabolite** | **Hexanoylcarnitine** C5H11-CO-Carnitine |
| **Formula** | $C_{13}H_{25}NO_4$ |
| **Exact mass** | 259.1784 |

| | |
|---|---|
| **Ion type** | [M+H]+ |
| **m/z** | 260.1856 |

## Isotopic patterns

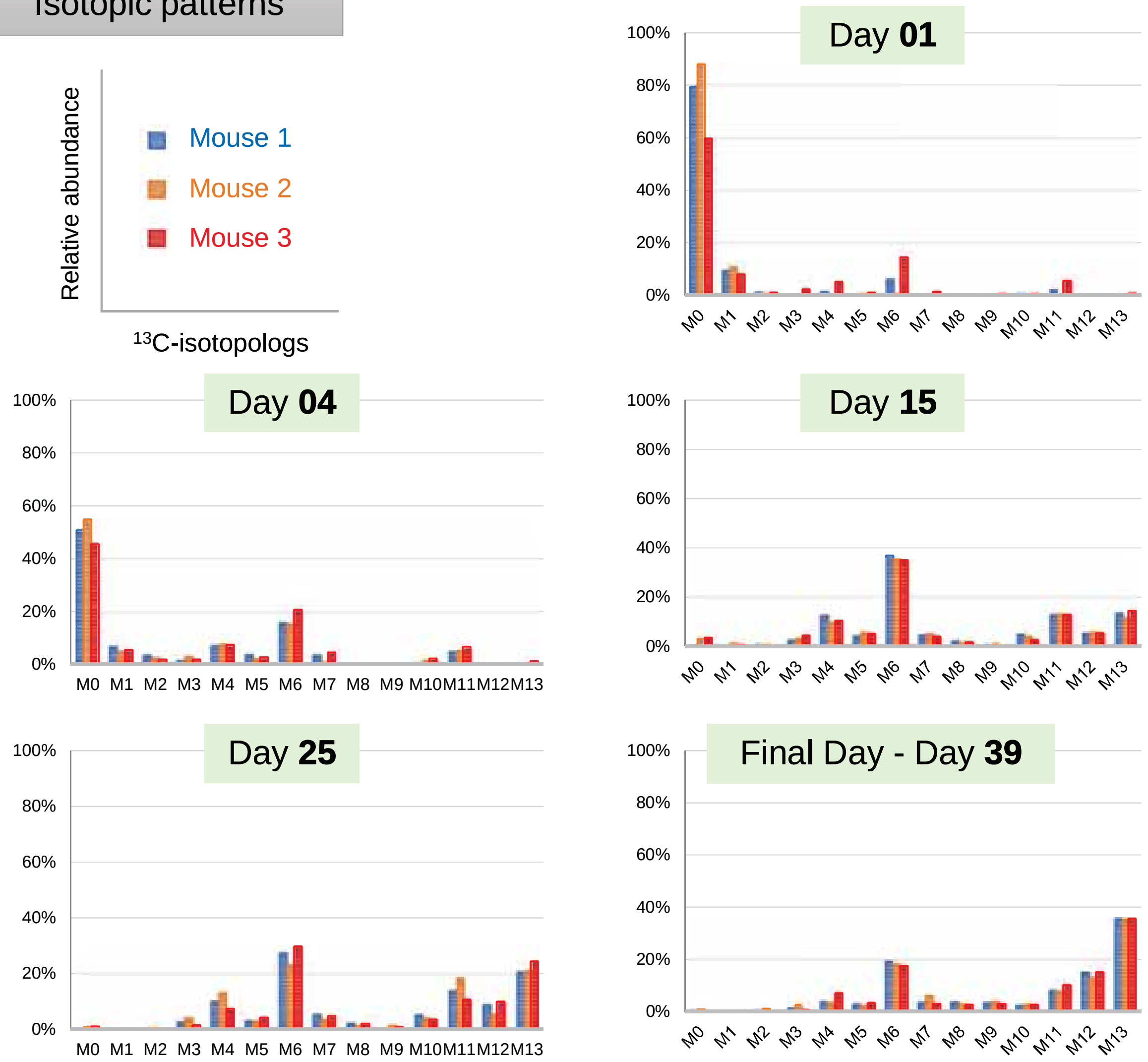

# Identified metabolites in murine urine

*Metabolites from the chemical library*

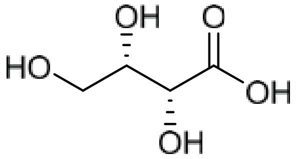


| Metabolite | L-Threonic acid |
|---|---|
| Formula | C4H8O5 |
| Exact mass | 136.0372 |

| Ion type | [M-H]- |
|---|---|
| m/z | 135.0299 |

## Isotopic patterns

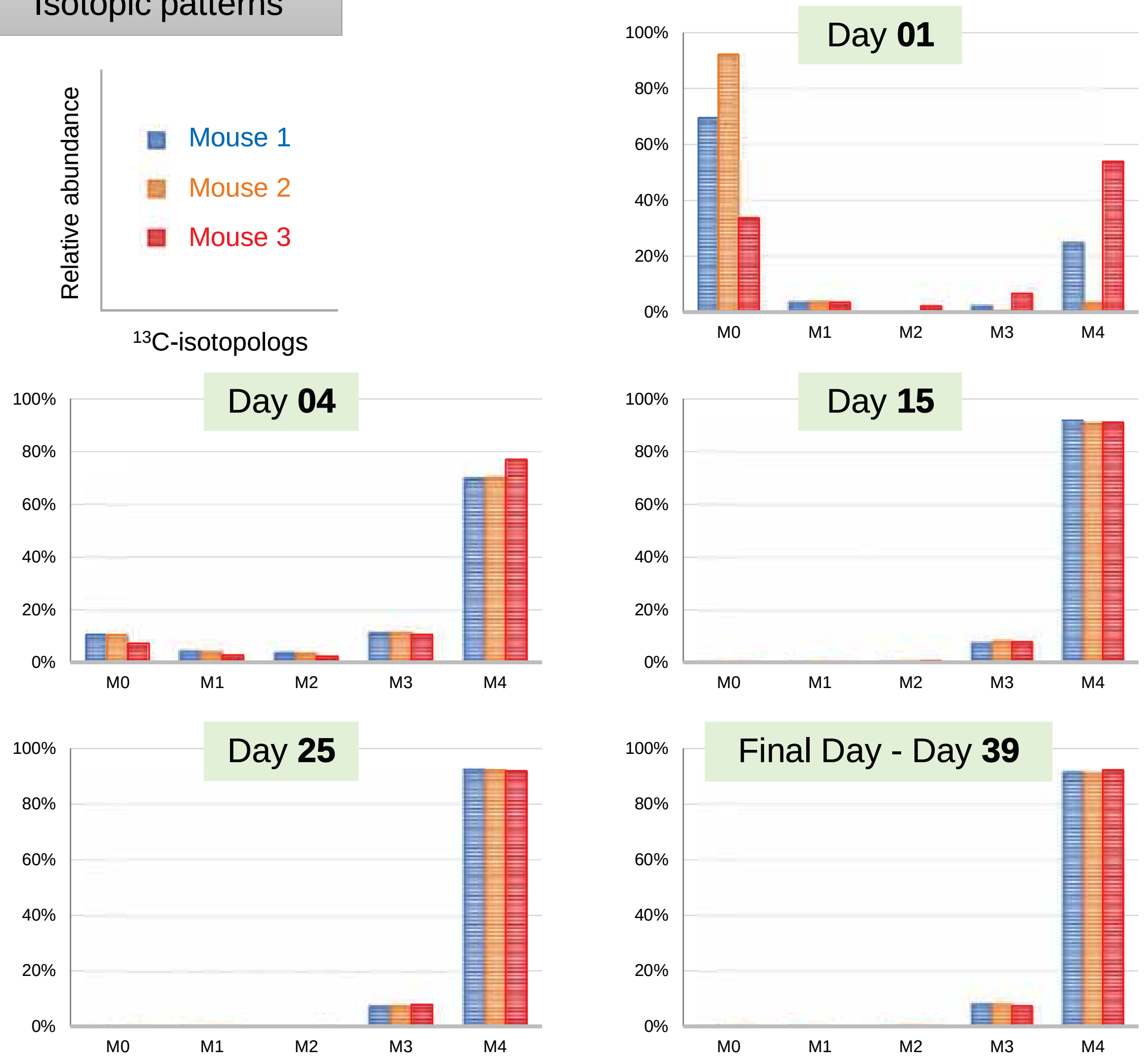


## Fractional $^{13}$C-enrichment

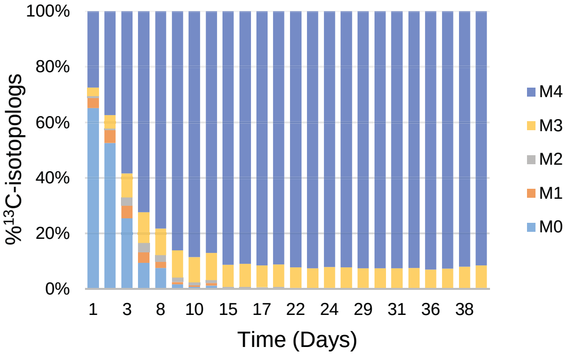


## $^{13}$C-enrichment kinetics

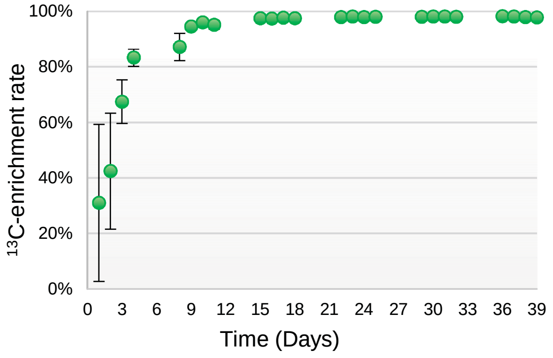

# Identified metabolites in murine urine

*Metabolites from the chemical library*

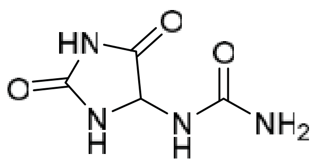

| Metabolite | Allantoin |
| --- | --- |
| Formula | $C_4H_6N_4O_3$ |
| Exact mass | 158.044 |

| Ion type | [M-H]- |
| --- | --- |
| m/z | 157.0367 |

## Isotopic patterns

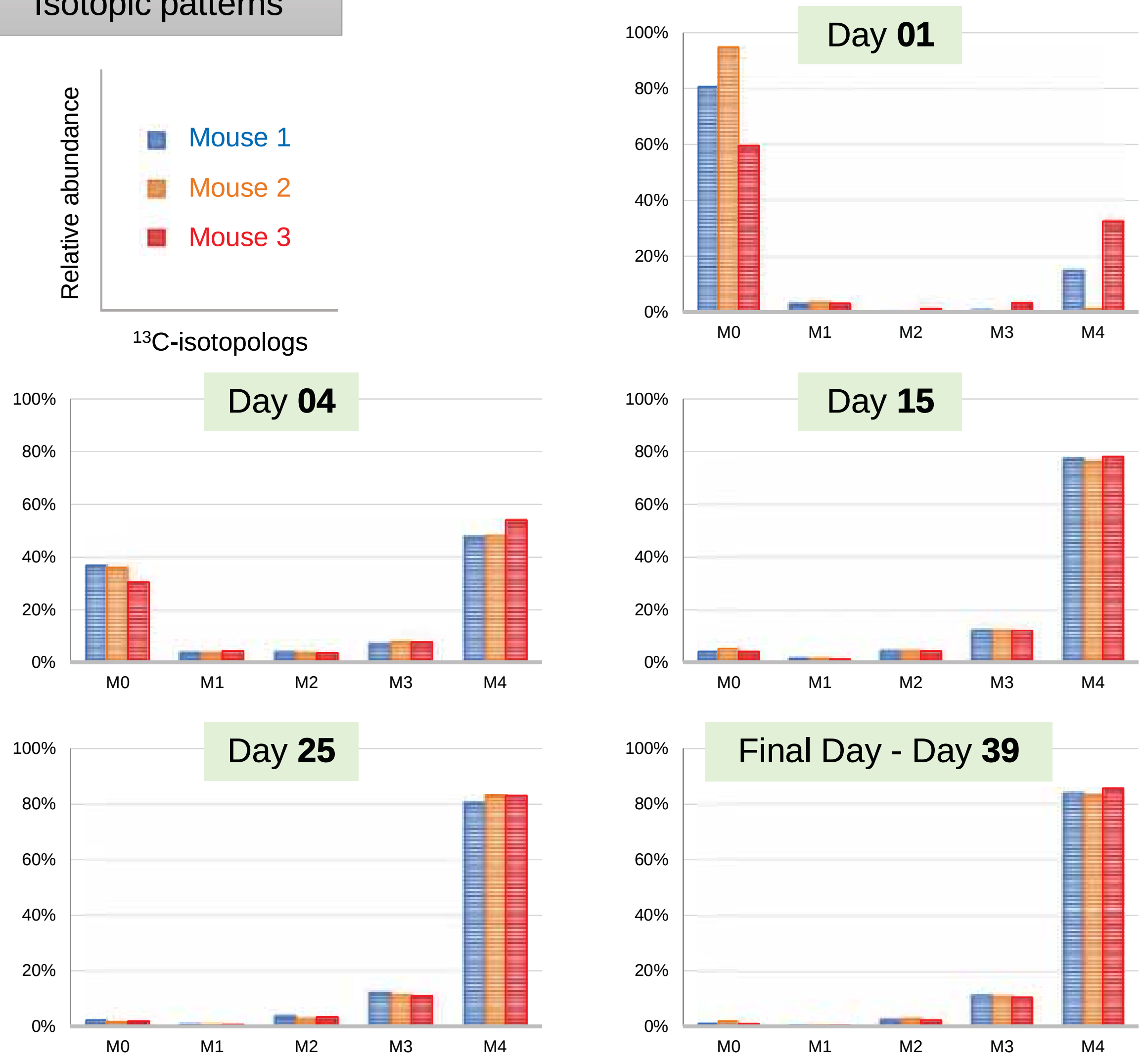


## Fractional $^{13}$C-enrichment

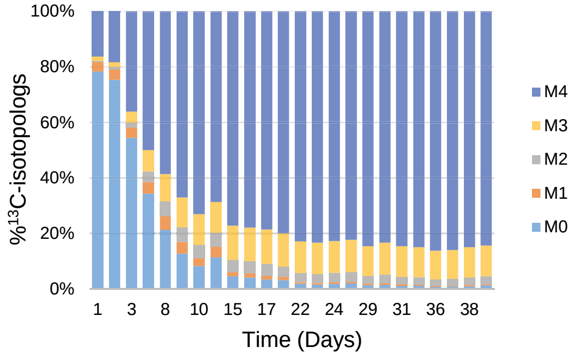


## $^{13}$C-enrichment kinetics

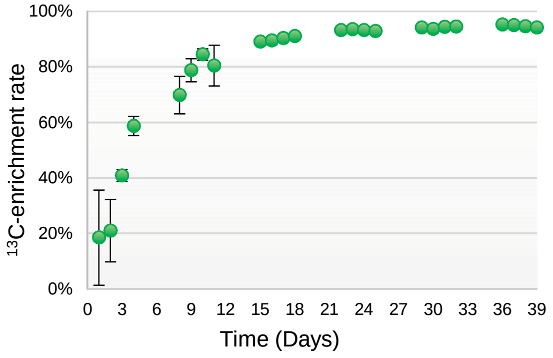

# Identified metabolites in murine urine

*Metabolites from the chemical library*

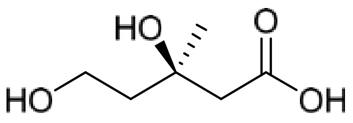


| Metabolite | Mevalonic acid |
|---|---|
| Formula | C6H12O4 |
| Exact mass | 148.0736 |

| Ion type | [M-H]- |
|---|---|
| m/z | 147.0663 |

## Isotopic patterns

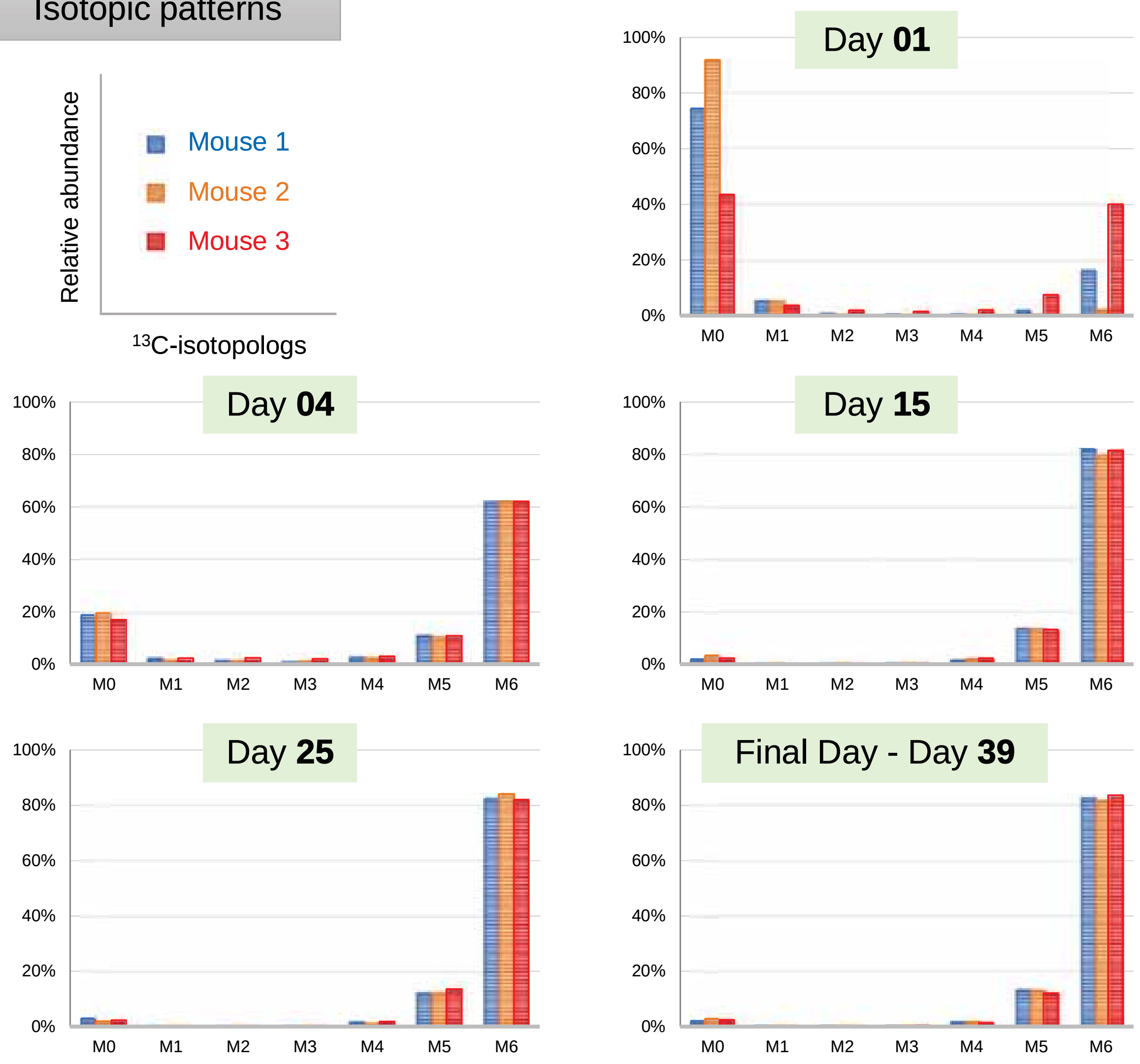

# Identified metabolites in murine urine

*Metabolites from the chemical library*

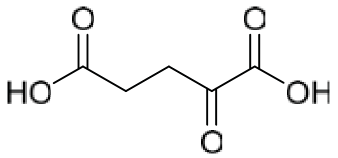


| Metabolite | alpha-Ketoglutaric acid |
|---|---|
| Formula | C5H6O5 |
| Exact mass | 146.0215 |

| Ion type | [M-H]- |
|---|---|
| m/z | 145.0142 |

## Isotopic patterns

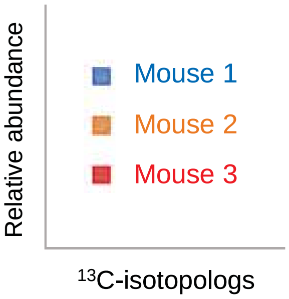


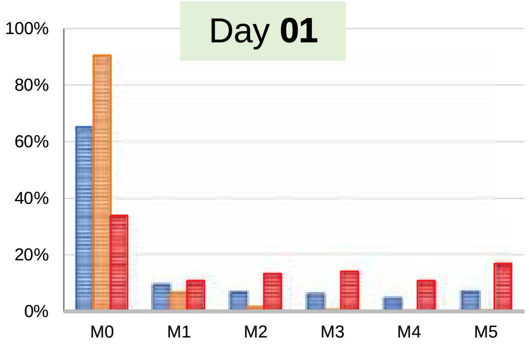


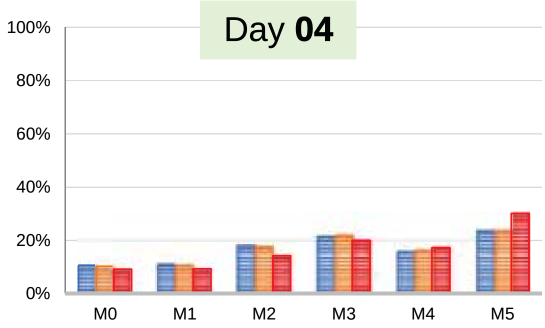


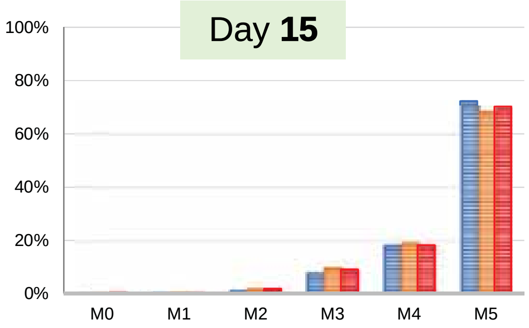


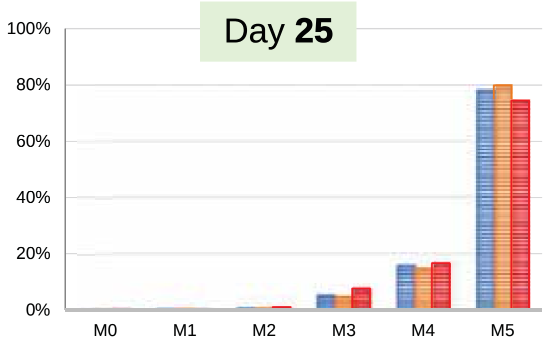


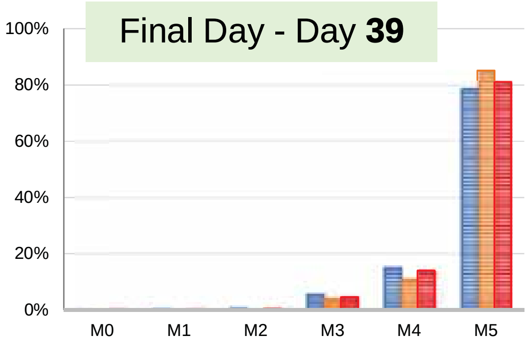


## Fractional $^{13}$C-enrichment

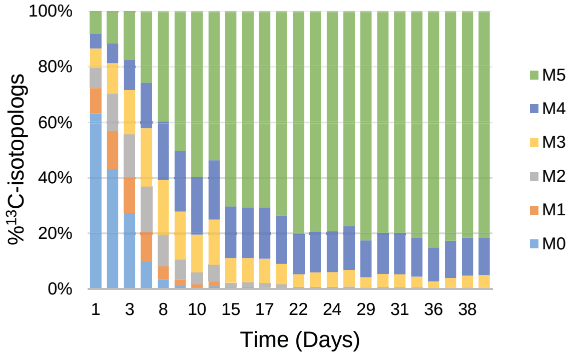


## $^{13}$C-enrichment kinetics

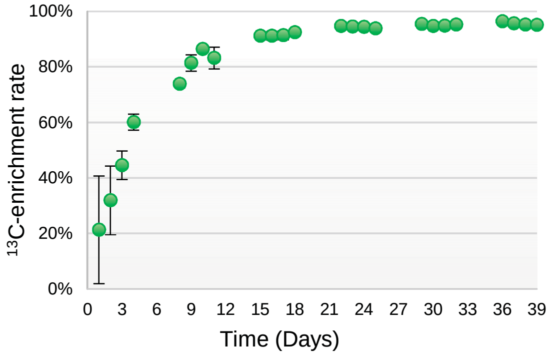

# Identified metabolites in murine urine

*Metabolites from the chemical library*

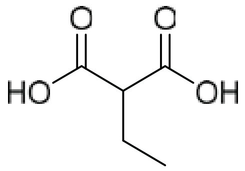


| | |
|---|---|
| **Metabolite** | **Ehtylmalonic acid** |
| **Formula** | C5H8O4 |
| **Exact mass** | 132.0423 |

| | |
|---|---|
| **Ion type** | [M-H]- |
| **m/z** | 131.035 |

## Isotopic patterns

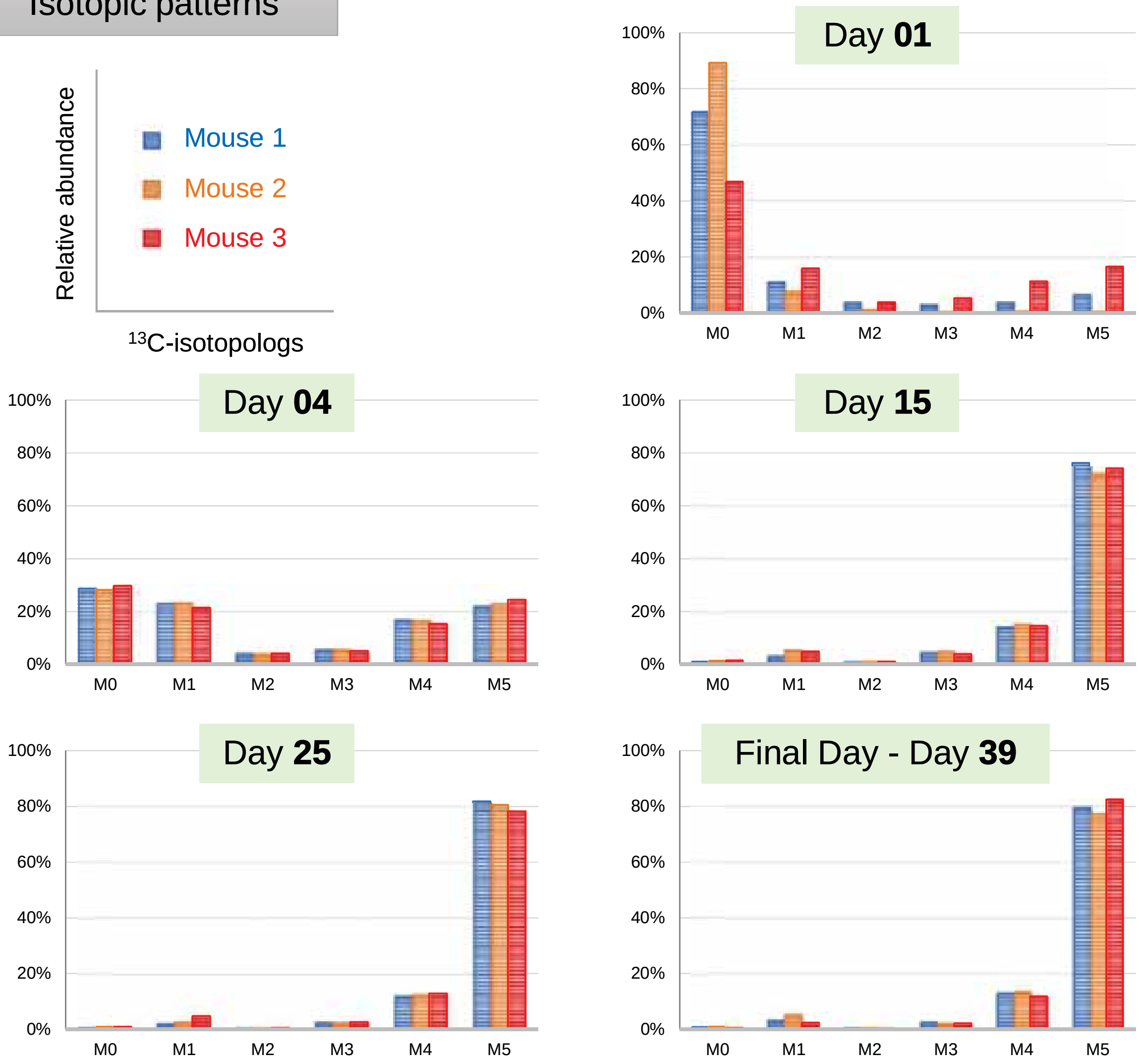


## Fractional $^{13}$C-enrichment

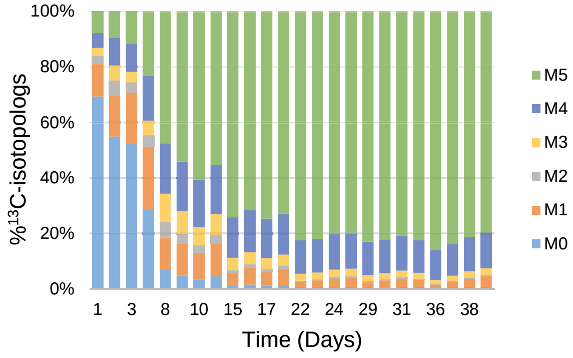


## $^{13}$C-enrichment kinetics

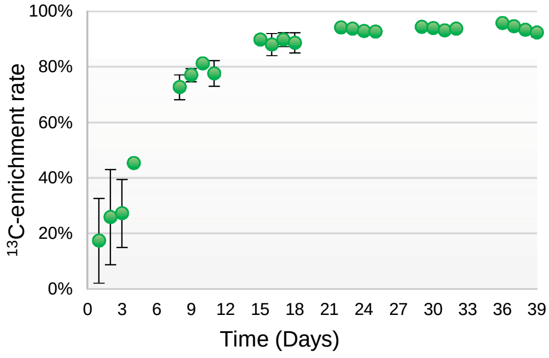

# Identified metabolites in murine urine

*Metabolites from the chemical library*

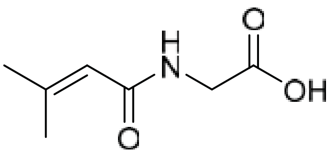


| | |
|---|---|
| **Metabolite** | **3-Methylcrotonyl-glycine**<br>C4H7-CO-Glycine_RT3.9 |
| **Formula** | C7H11NO3 |
| **Exact mass** | 157.0738 |

| | |
|---|---|
| **Ion type** | [M-H]- |
| **m/z** | 156.0666 |

## Isotopic patterns

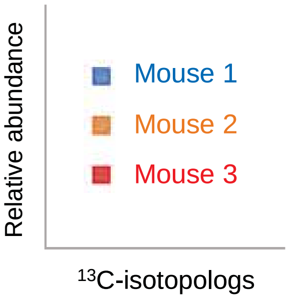


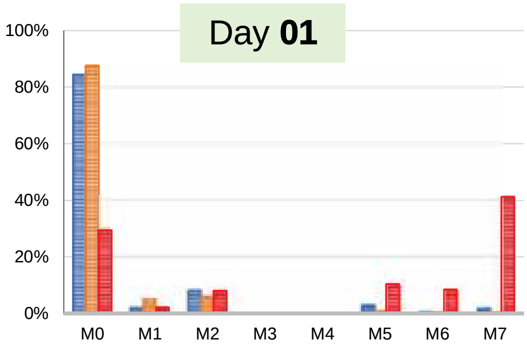


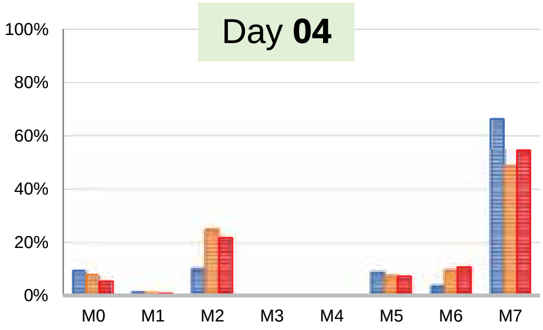


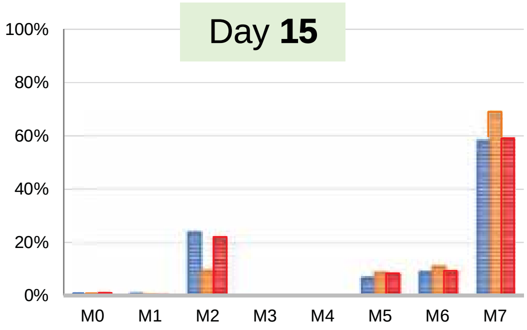


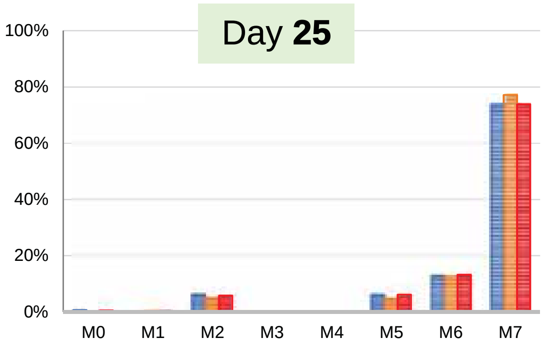


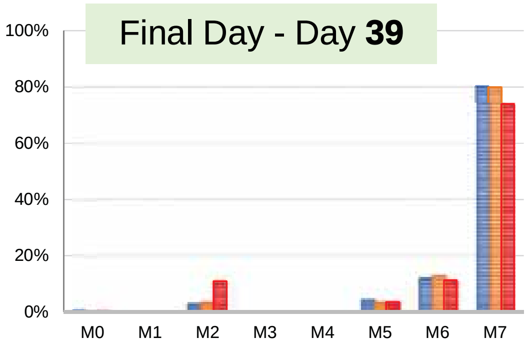


## Fractional $^{13}$C-enrichment

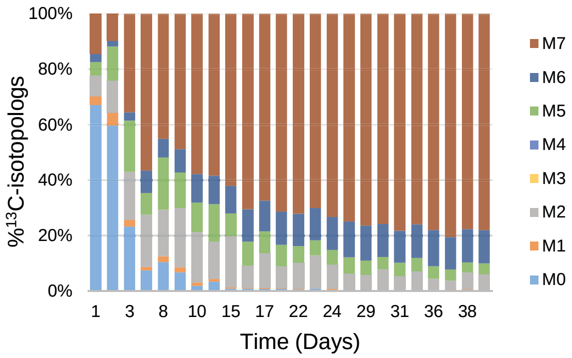


## $^{13}$C-enrichment kinetics

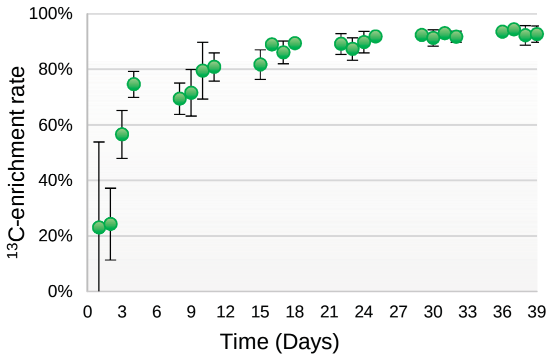

# Identified metabolites in murine urine

*Metabolites from the chemical library*

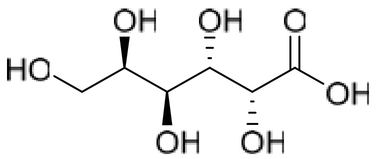


| Metabolite | Galactonic acid |
|---|---|
| **Formula** | C6H12O7 |
| **Exact mass** | 196.0583 |

| Ion type | [M-H]- |
|---|---|
| **m/z** | 195.051 |

## Isotopic patterns

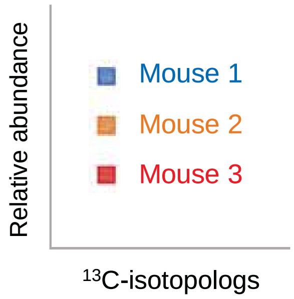


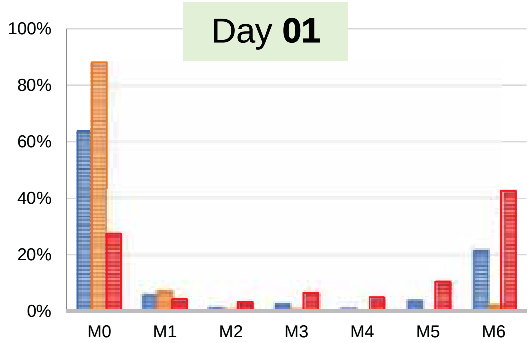


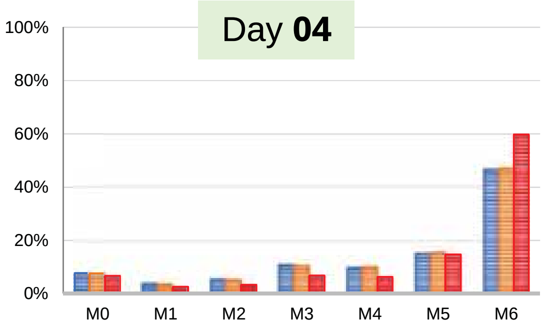


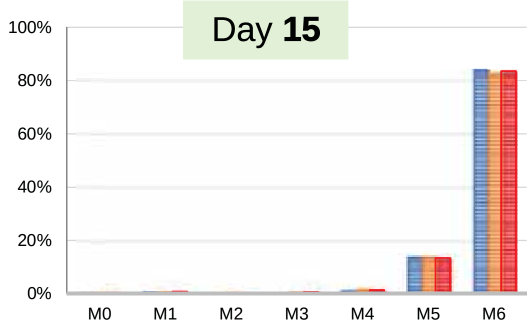


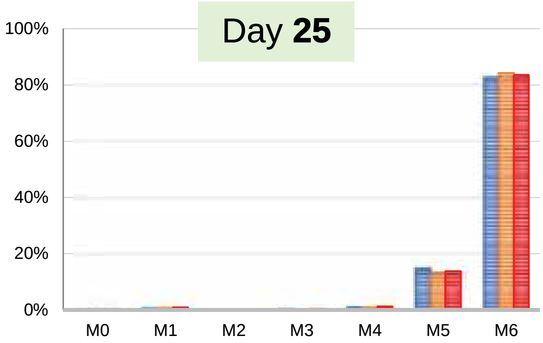


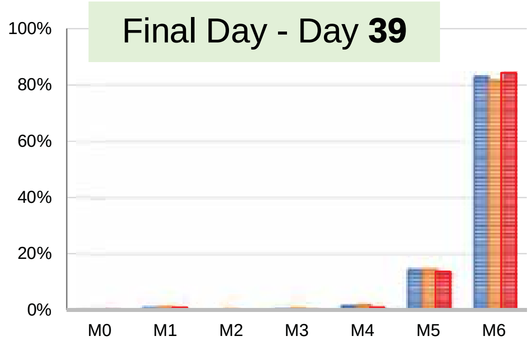


## Fractional $^{13}$C-enrichment

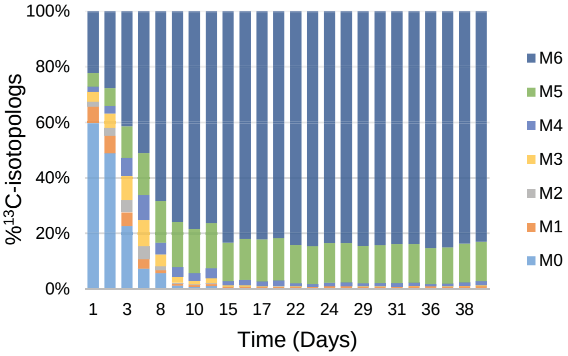


## $^{13}$C-enrichment kinetics

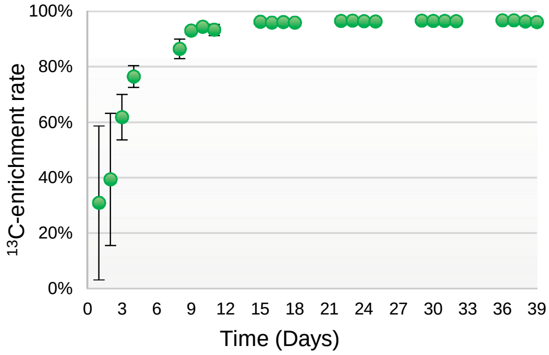

# Identified metabolites in murine urine

*Metabolites from the chemical library*

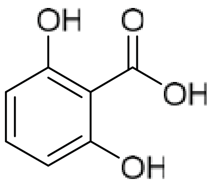


| **Metabolite** | **2-6-Dihydroxybenzoic acid** |
|---|---|
| **Formula** | C7H6O4 |
| **Exact mass** | 154.0266 |

| **Ion type** | [M-H]- |
|---|---|
| **m/z** | 153.0193 |

Isotopic patterns

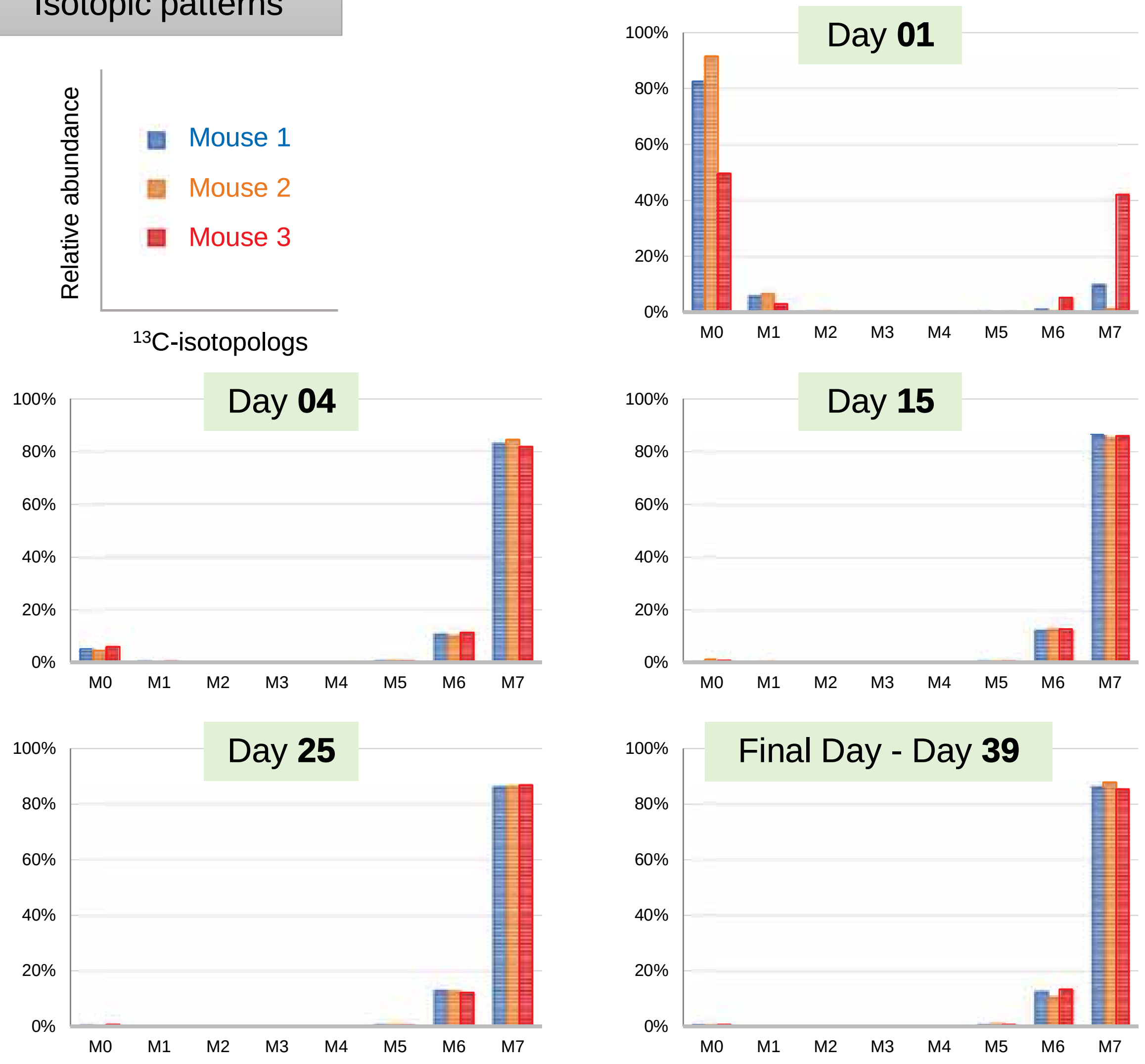

# Identified metabolites in murine urine

*Metabolites from the chemical library*

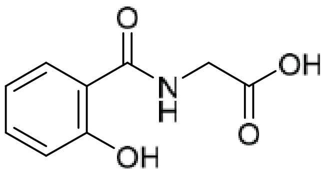

| Metabolite | o-Hydroxyhippuric acid |
|---|---|
| Formula | C9H9NO4 |
| Exact mass | 195.0532 |

| Ion type | [M-H]- |
|---|---|
| m/z | 194.0459 |

## Isotopic patterns

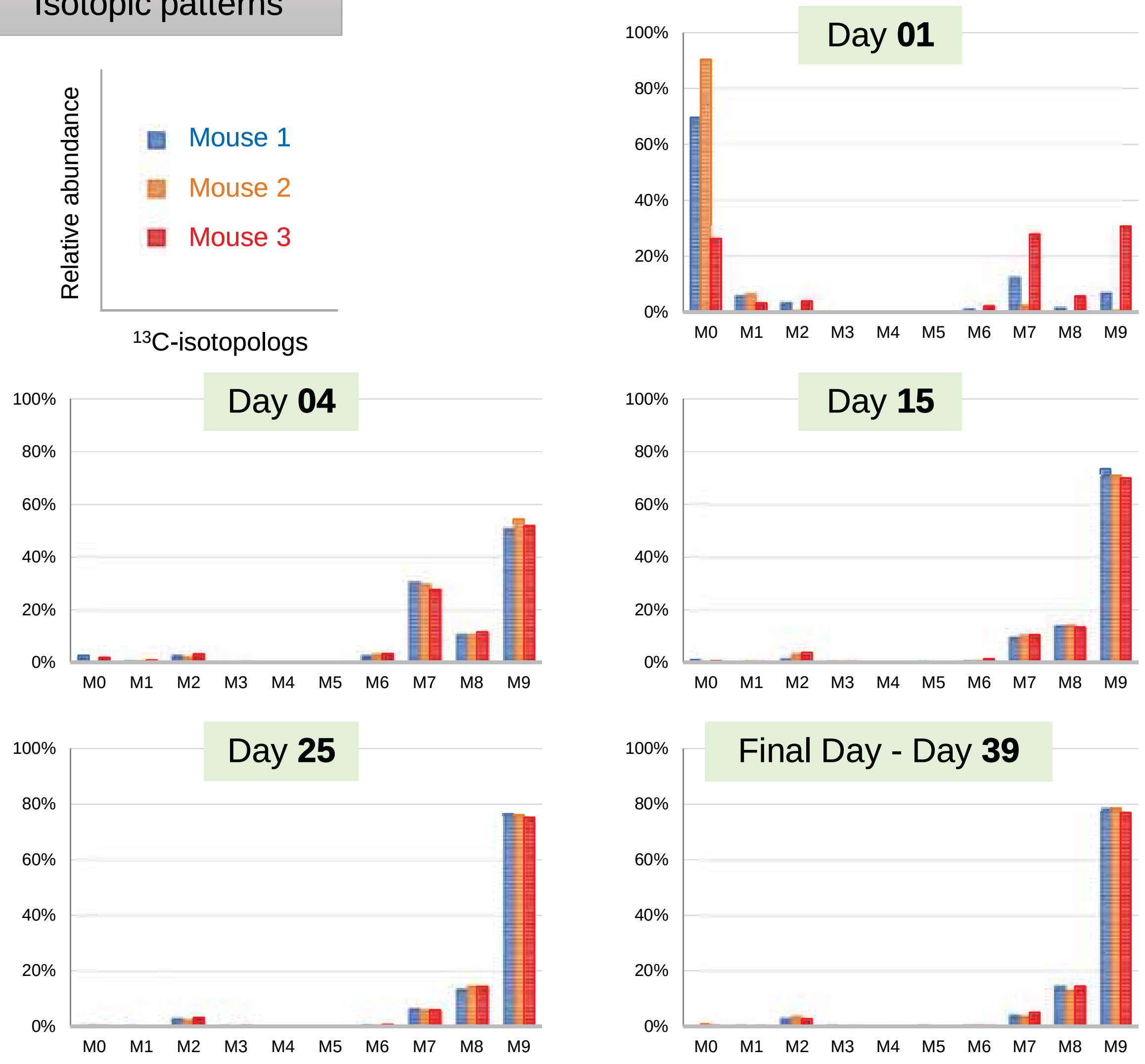

# Identified metabolites in murine urine

*Metabolites from the chemical library*

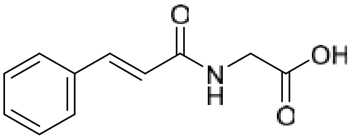


| | |
|---|---|
| **Metabolite** | **Cinnamoylglycine** C8H7-CO-Glycine |
| **Formula** | C11H11NO3 |
| **Exact mass** | 205.0739 |

| | |
|---|---|
| **Ion type** | [M-H]- |
| **m/z** | 204.0666 |

## Isotopic patterns

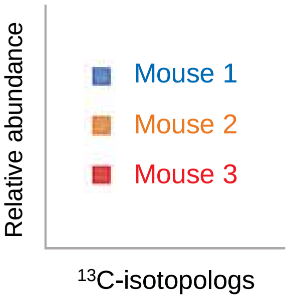


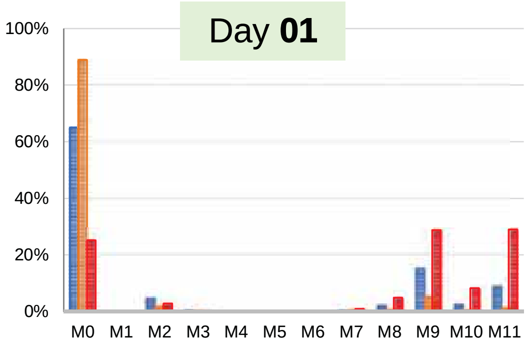


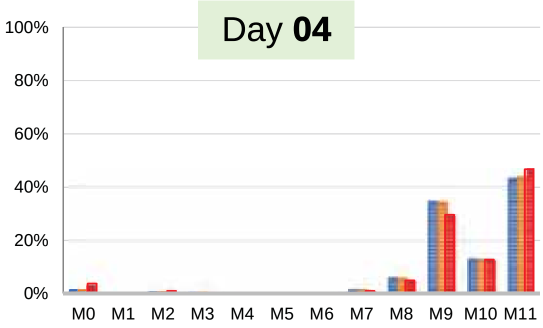


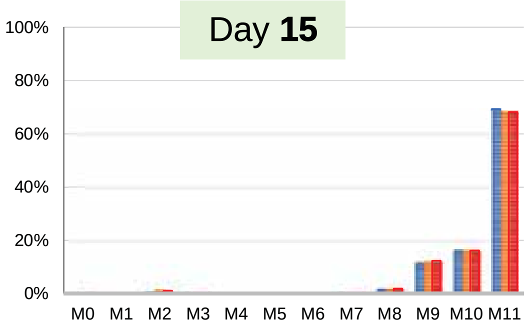


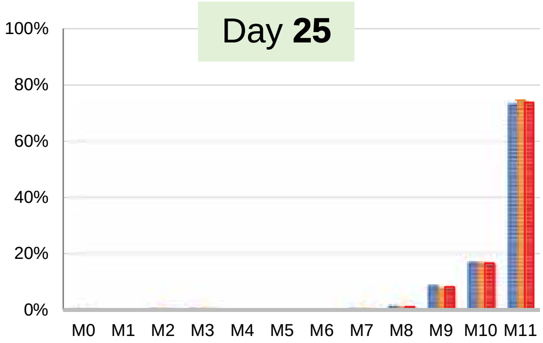


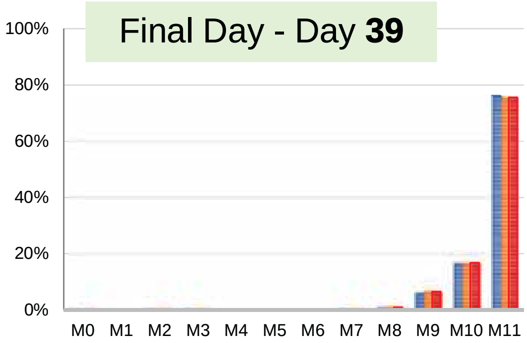


## Fractional $^{13}$C-enrichment

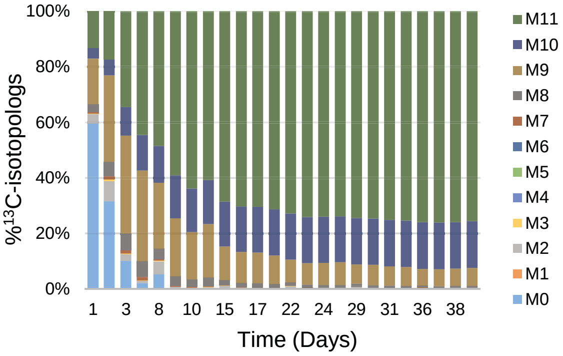


## $^{13}$C-enrichment kinetics

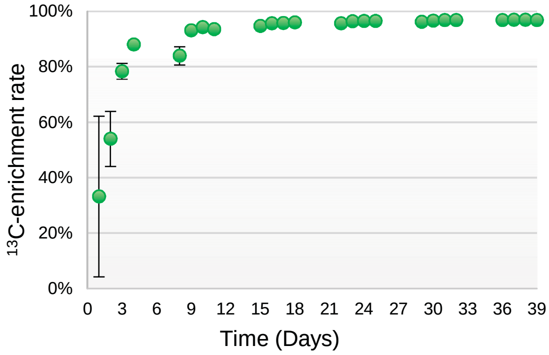

# Identified metabolites in murine urine

*Metabolites from the chemical library*

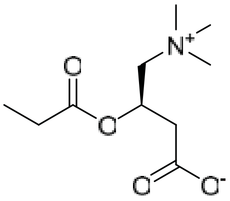

| | |
|---|---|
| **Metabolite** | **Propionylcarnitine**<br>C2H5-CO-Carnitine |
| **Formula** | C10H19NO4 |
| **Exact mass** | 217.1314 |

| | |
|---|---|
| **Ion type** | [M+H]+ |
| **m/z** | 218.1387 |

## Isotopic patterns

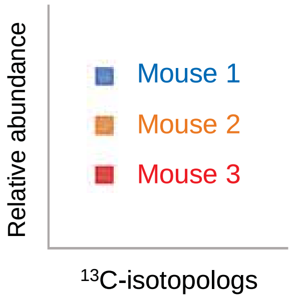


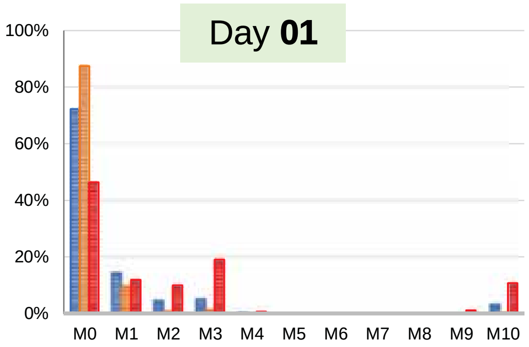


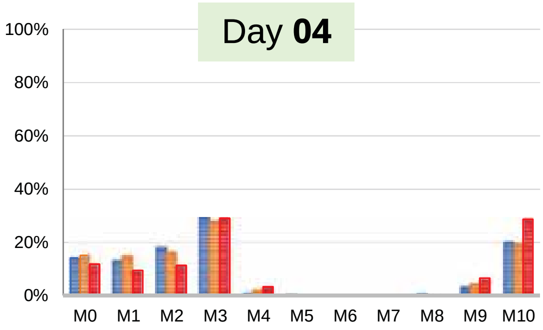


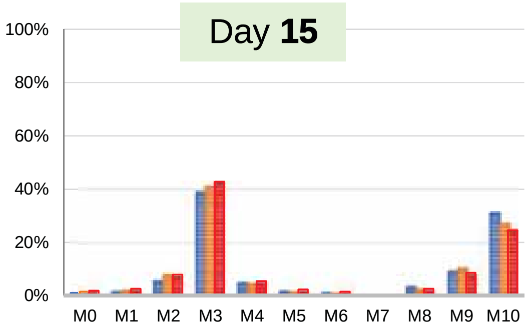


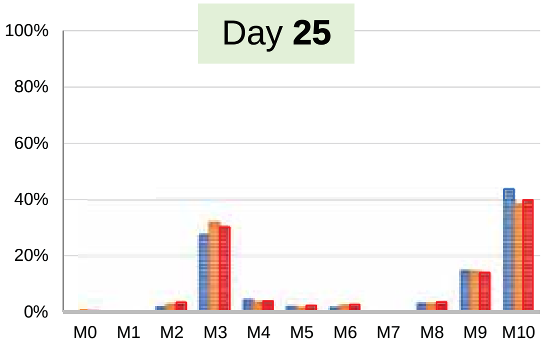


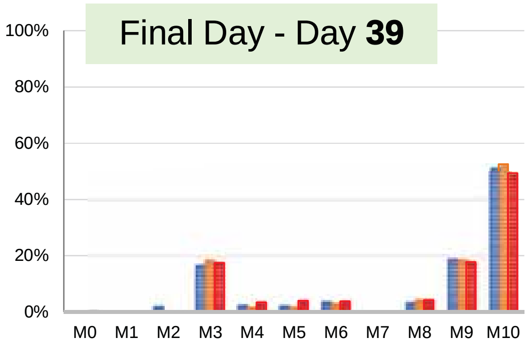


## Fractional $^{13}$C-enrichment

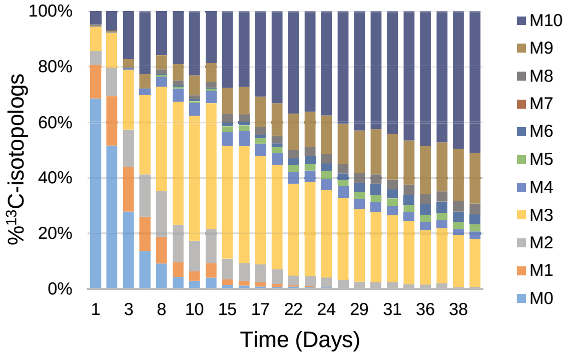


## $^{13}$C-enrichment kinetics

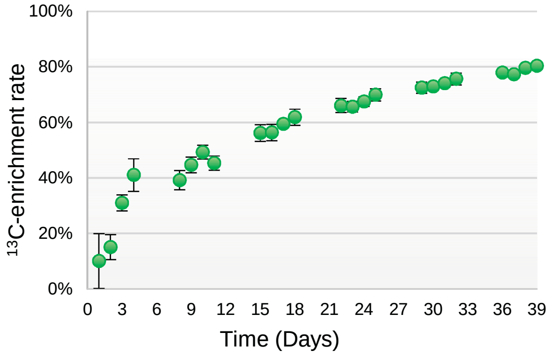

# Identified metabolites in murine urine

*Metabolites from the chemical library*

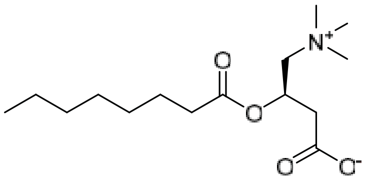

| **Metabolite** | **Octanoylcarnitine**<br>C7H15-CO-Carnitine_RT7.4 |
|---|---|
| **Formula** | C15H29NO4 |
| **Exact mass** | 287.2097 |

| **Ion type** | [M+H]+ |
|---|---|
| **m/z** | 288.2169 |

## Isotopic patterns

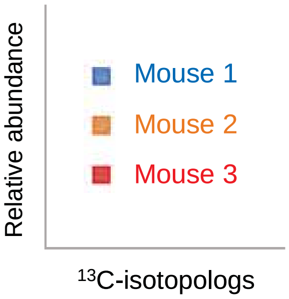


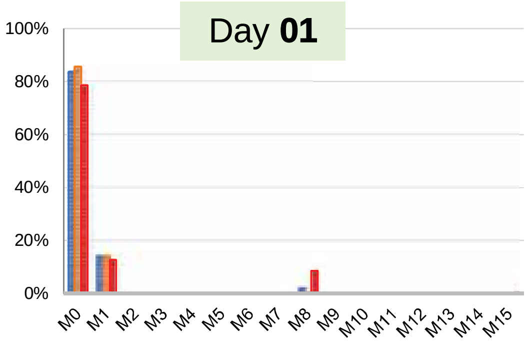


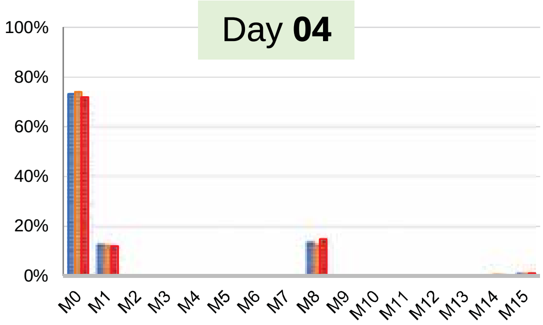


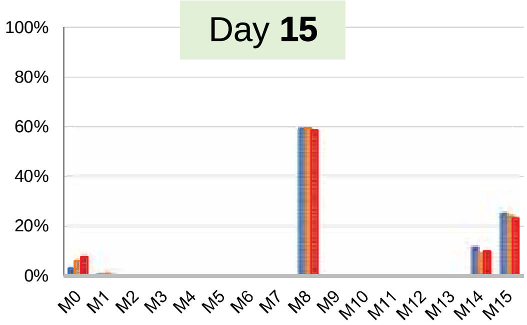


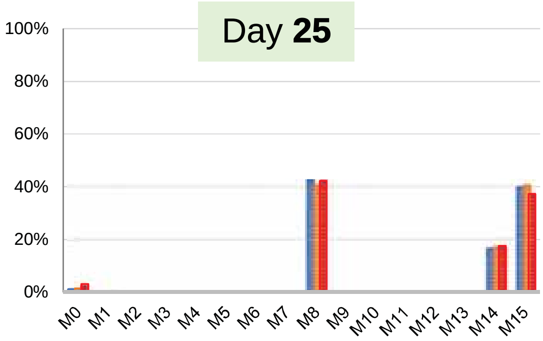


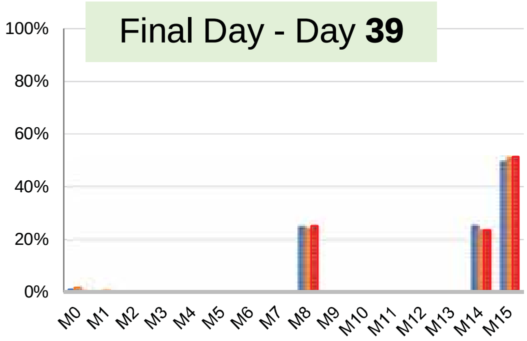


## Fractional $^{13}$C-enrichment

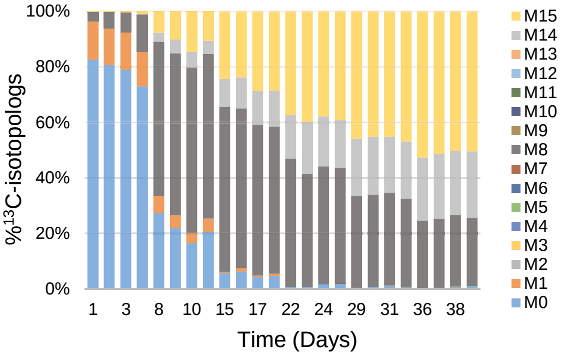


## $^{13}$C-enrichment kinetics

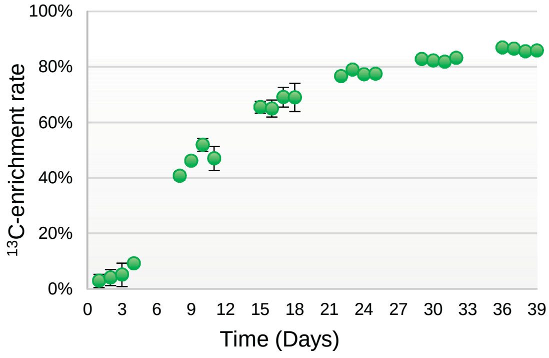

# Identified metabolites in murine urine

*Metabolites from the chemical library*

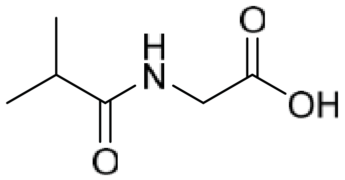


| Metabolite | **N-Isobutyrylglycine**<br>C4H9-CO-Glycine |
|---|---|
| **Formula** | C6H11NO3 |
| **Exact mass** | 145.0739 |

| Ion type | [M-H]- |
|---|---|
| **m/z** | 144.0666 |

## Isotopic patterns

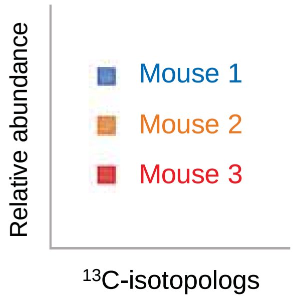


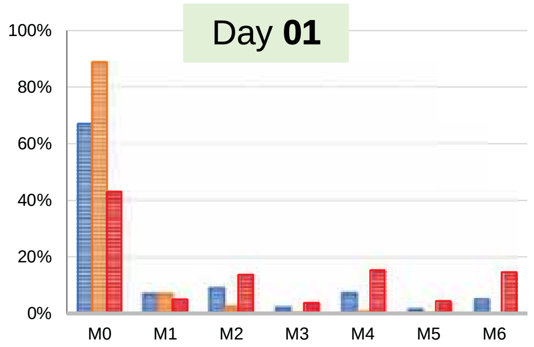


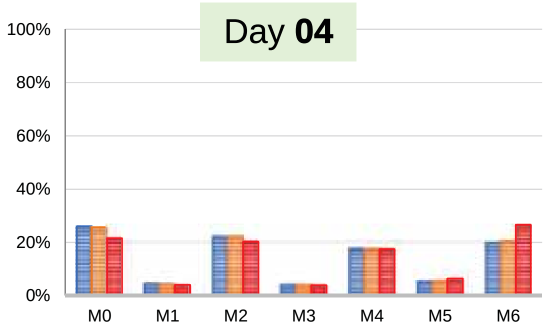


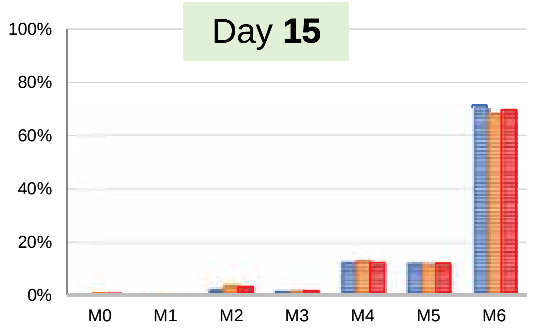


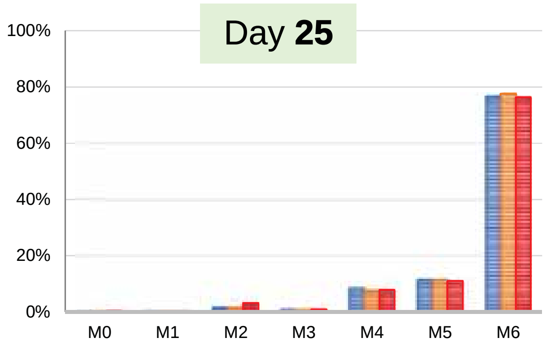


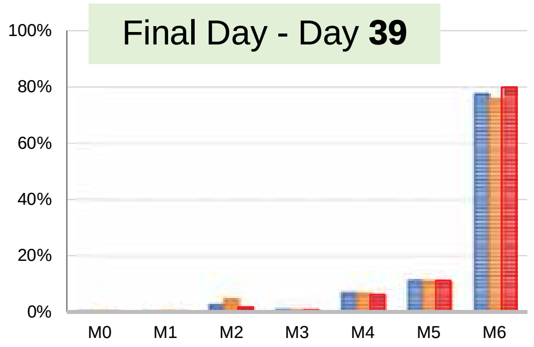


## Fractional $^{13}C$-enrichment

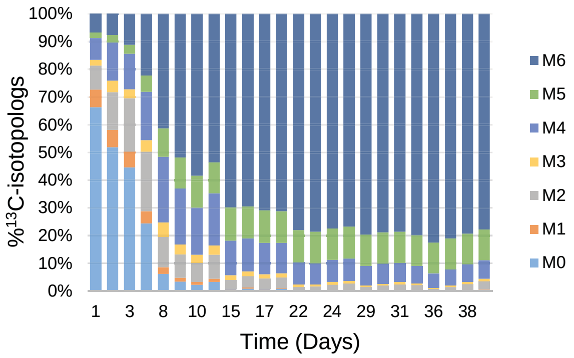


## $^{13}C$-enrichment kinetics

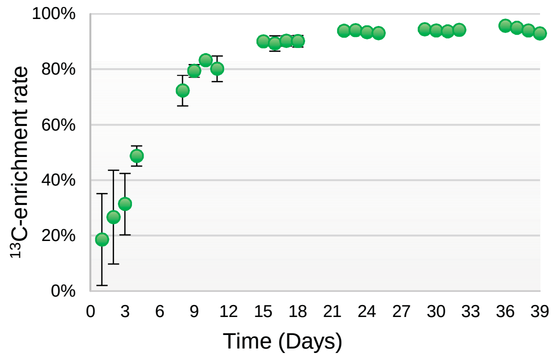

# Identified metabolites in murine urine

*Metabolites from the chemical library*

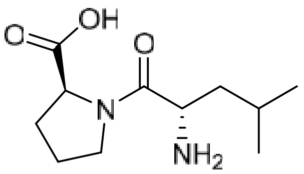


| Metabolite | Leu-Pro |
|---|---|
| Formula | C11H20N2O3 |
| Exact mass | 228.1474 |

| Ion type | [M+H]+ |
|---|---|
| m/z | 229.1547 |

## Isotopic patterns

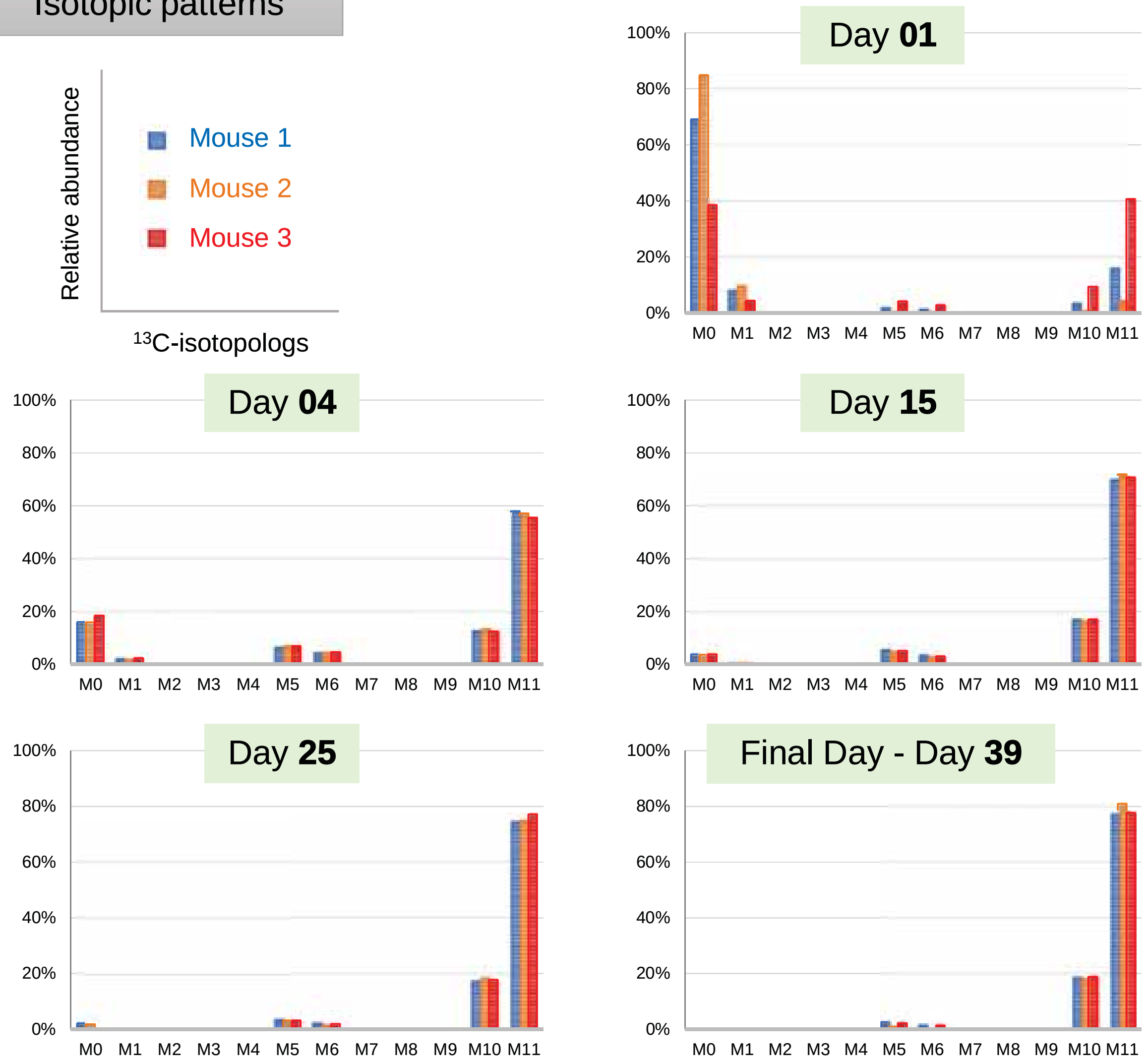


## Fractional $^{13}$C-enrichment

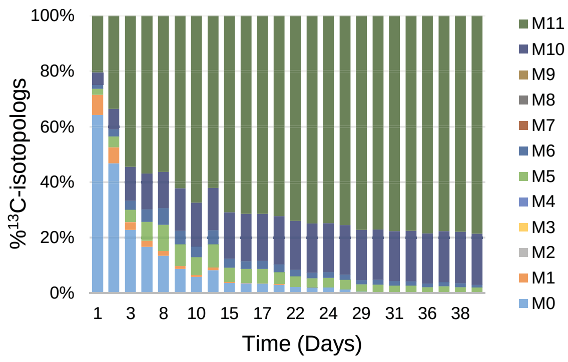


## $^{13}$C-enrichment kinetics

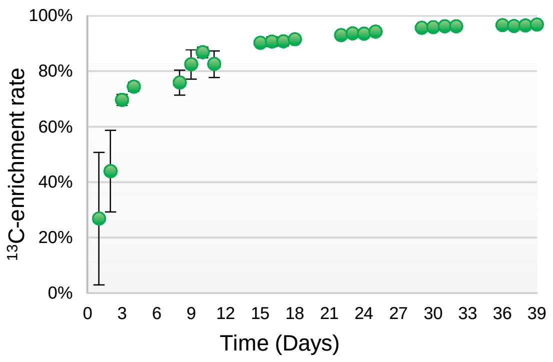

# Identified metabolites in murine urine

*Metabolites from the chemical library*

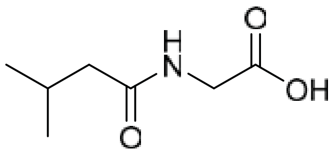


| **Metabolite** | **N-Isovaleroylglycine** |
|---|---|
| **Formula** | C7H13NO3 |
| **Exact mass** | 159.0895 |

| **Ion type** | [M-H]- |
|---|---|
| **m/z** | 158.0822 |

## Isotopic patterns

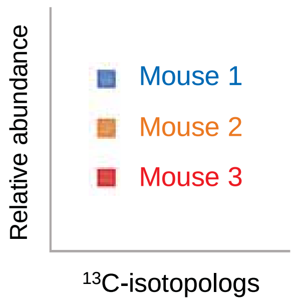


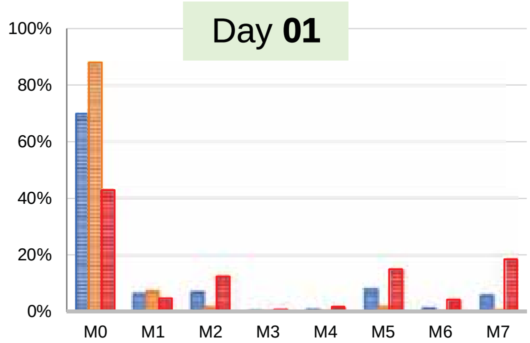


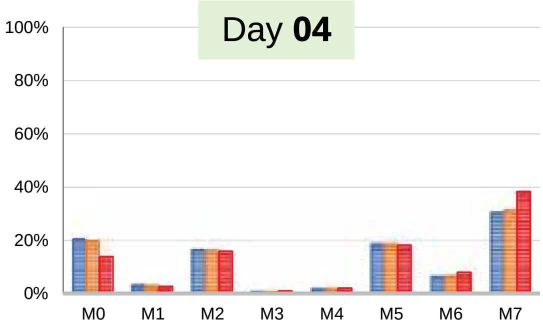


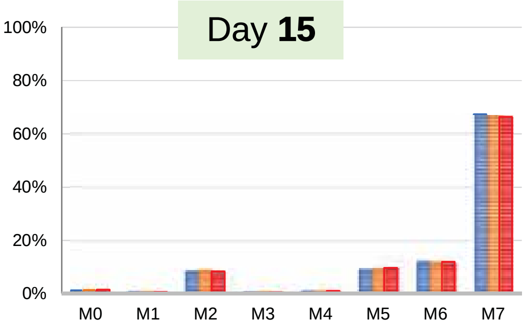


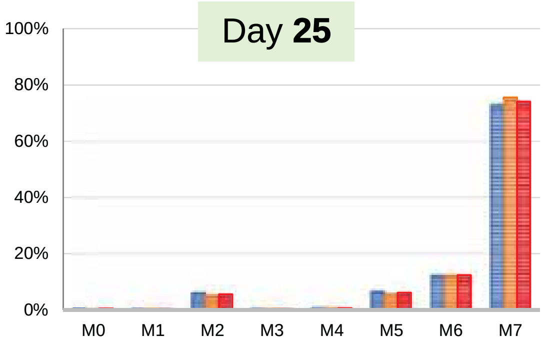


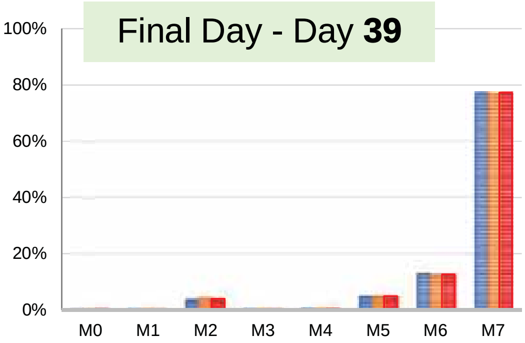


## Fractional $^{13}$C-enrichment

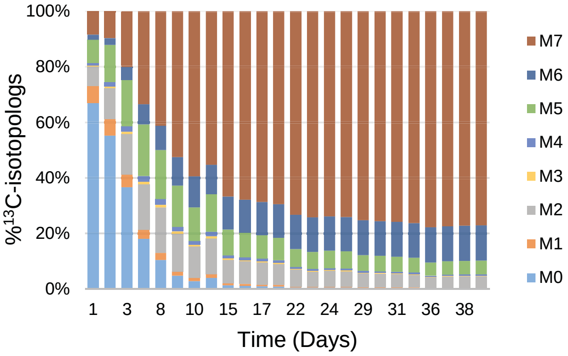


## $^{13}$C-enrichment kinetics

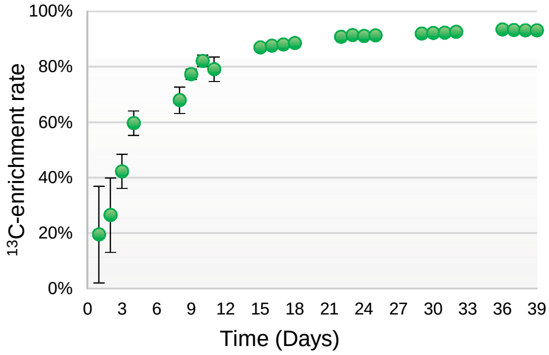

# Identified metabolites in murine urine

*Metabolites from the chemical library*

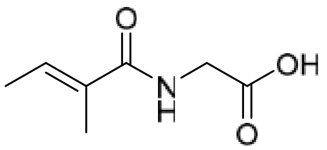


| | |
|---|---|
| **Metabolite** | **N-Tiglylglycine**<br>C4H7-CO-Glycine_RT3.7 |
| **Formula** | C7H11NO3 |
| **Exact mass** | 157.0738 |

| | |
|---|---|
| **Ion type** | [M-H]- |
| **m/z** | 156.0666 |

## Isotopic patterns

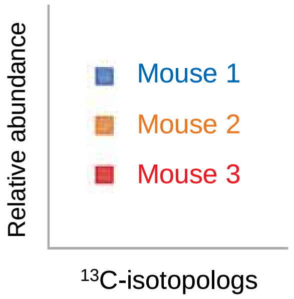


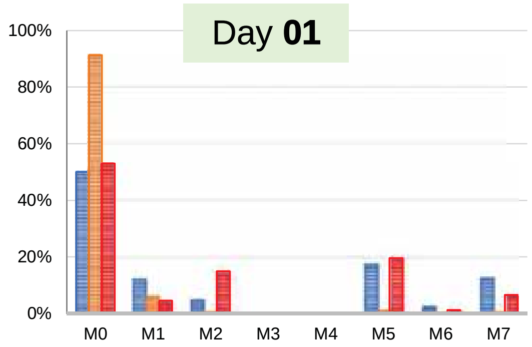


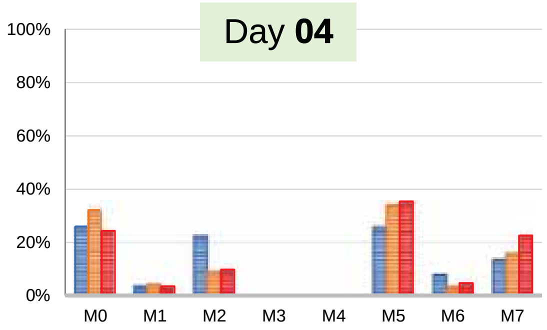


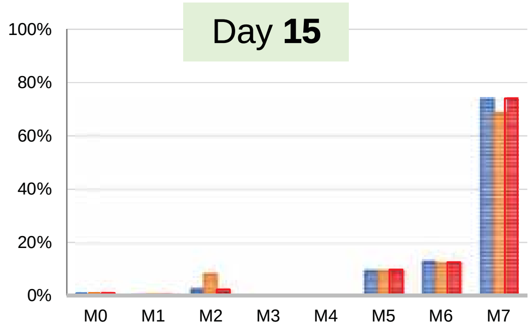


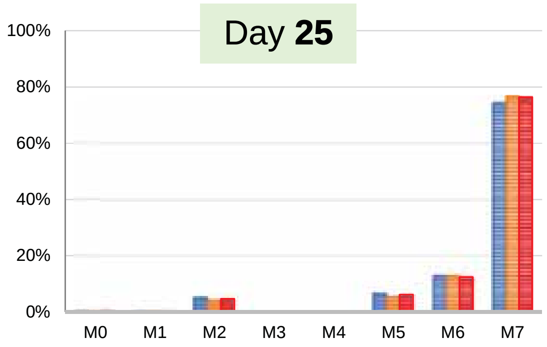


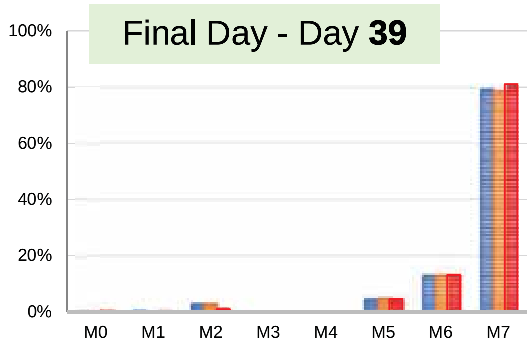


## Fractional $^{13}$C-enrichment

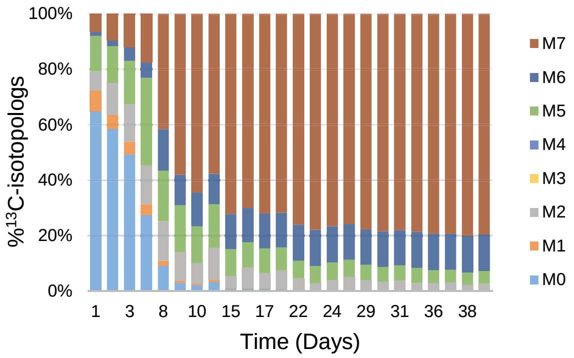


## $^{13}$C-enrichment kinetics

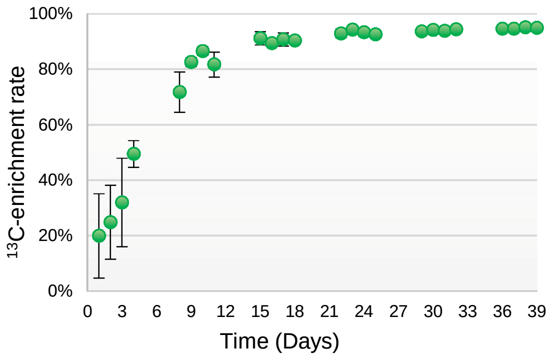

# Identified metabolites in murine urine

*Metabolites from the chemical library*

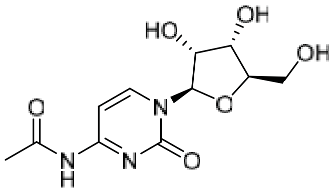


| Metabolite | N4-Acetylcytidine |
|---|---|
| Formula | C11H15N3O6 |
| Exact mass | 285.0961 |

| Ion type | [M+H]+ |
|---|---|
| m/z | 286.1034 |

## Isotopic patterns

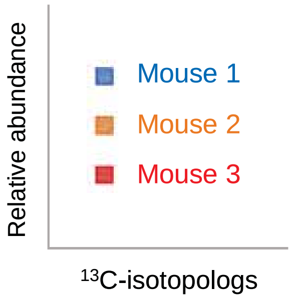


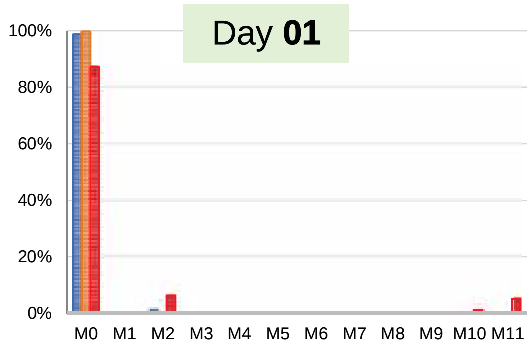


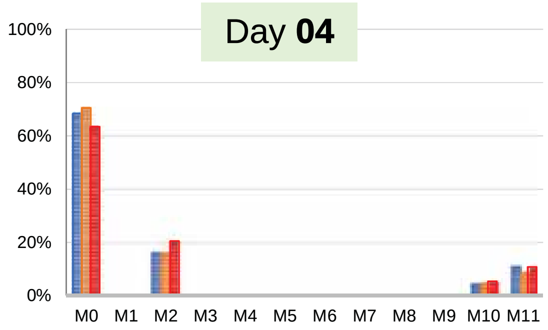


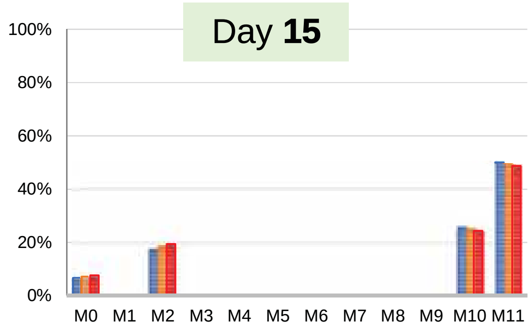


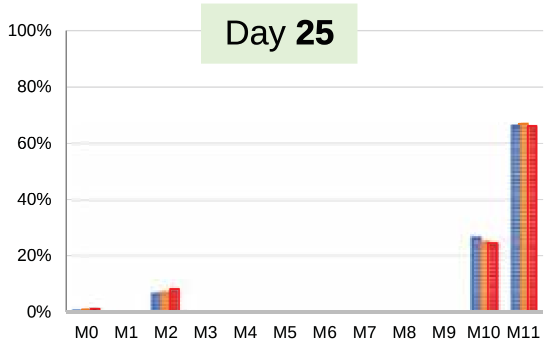


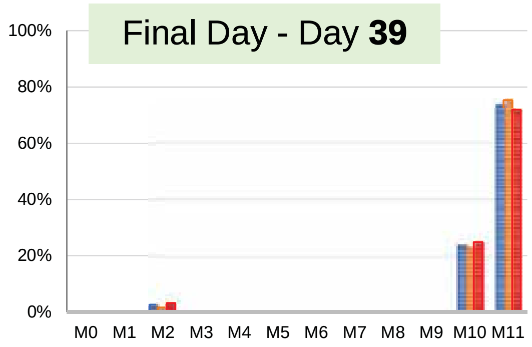


## Fractional $^{13}$C-enrichment

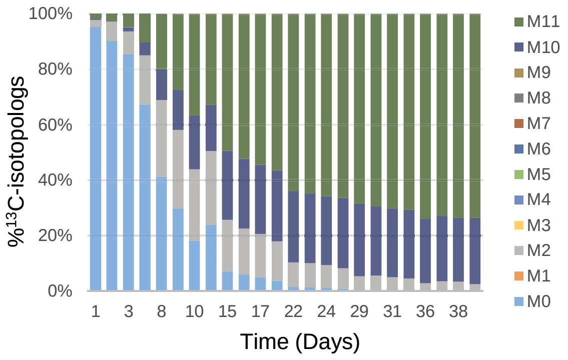


## $^{13}$C-enrichment kinetics

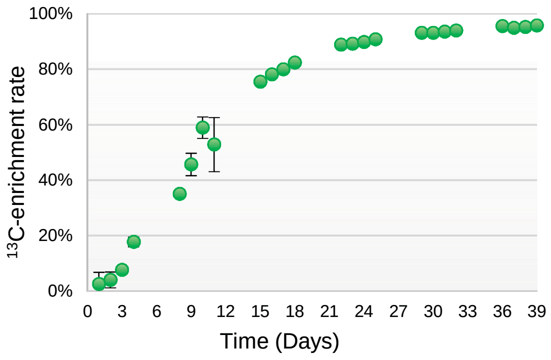

# Identified metabolites in murine urine

*Metabolites from the chemical library*

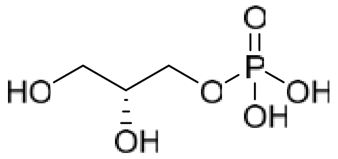


| Metabolite | Glycerol-3-phosphate |
|---|---|
| Formula | C3H9O6P |
| Exact mass | 172.0137 |

| Ion type | [M-H]- |
|---|---|
| m/z | 171.0064 |

## Isotopic patterns

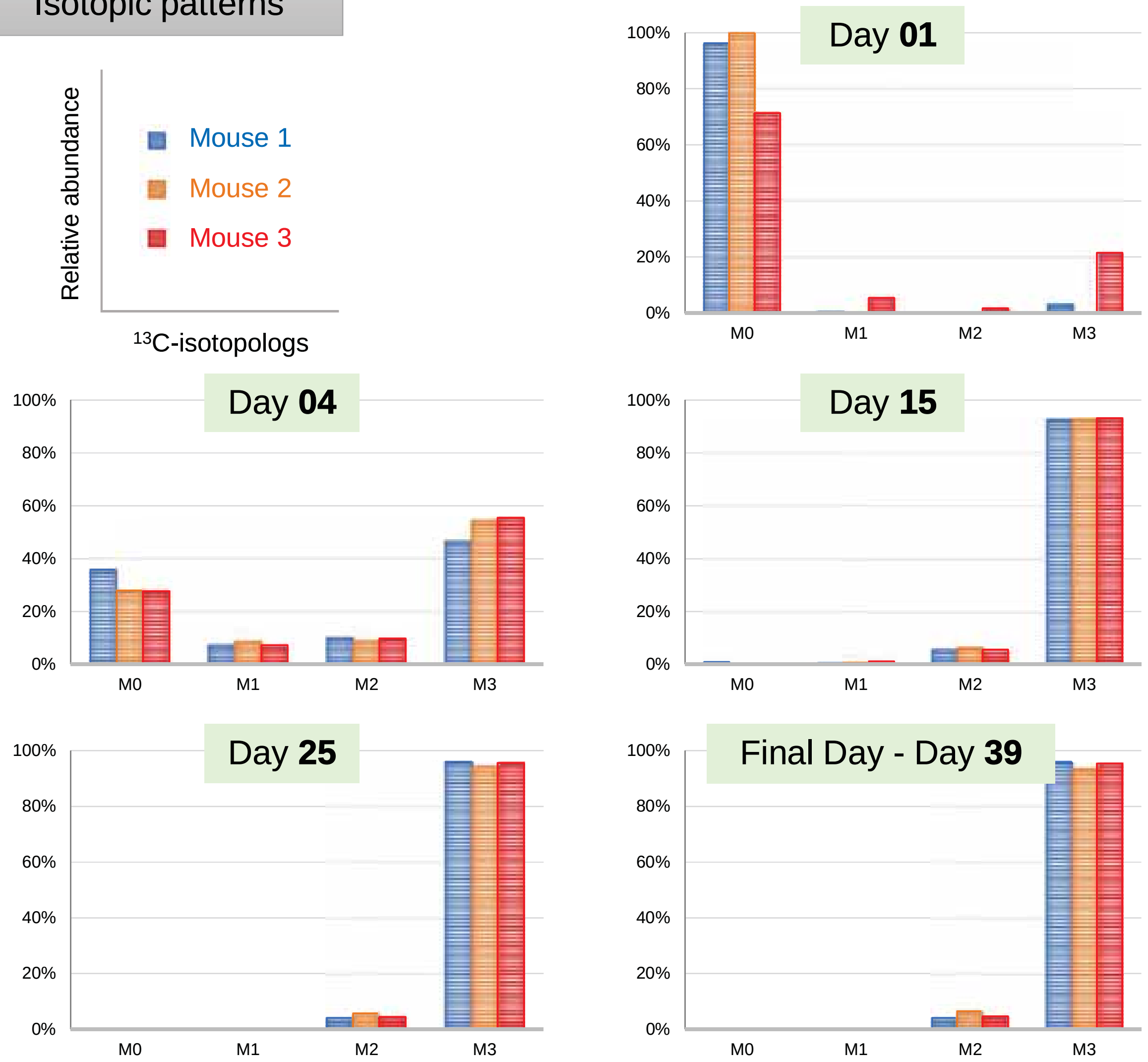

# Identified metabolites in murine urine

*Metabolites from the chemical library*

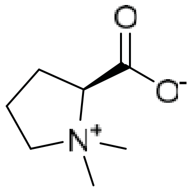

| Metabolite | Stachydrine |
|---|---|
| **Formula** | C7H13NO2 |
| **Exact mass** | 143.0946 |

| Ion type | [M+H]+ |
|---|---|
| **m/z** | 144.1019 |

## Isotopic patterns

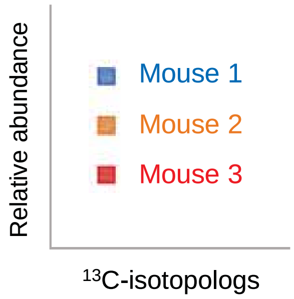


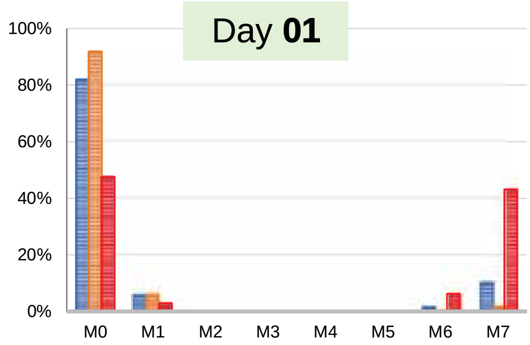


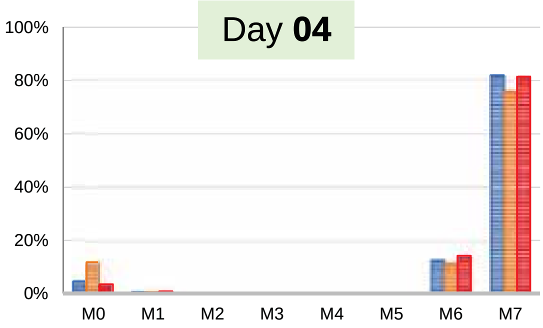


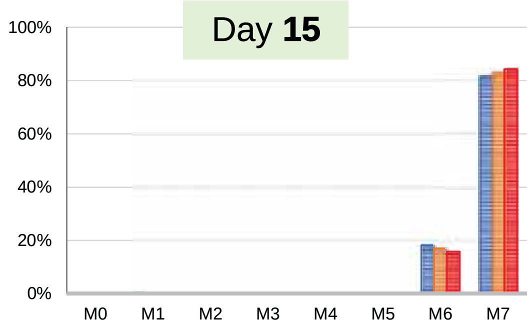


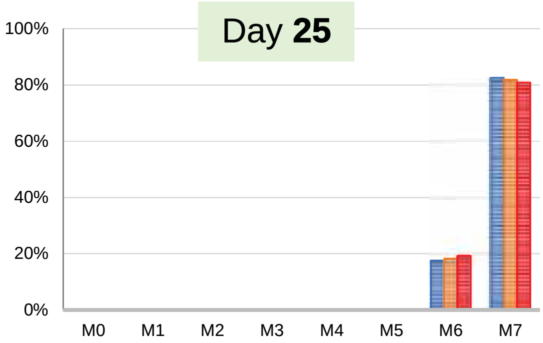


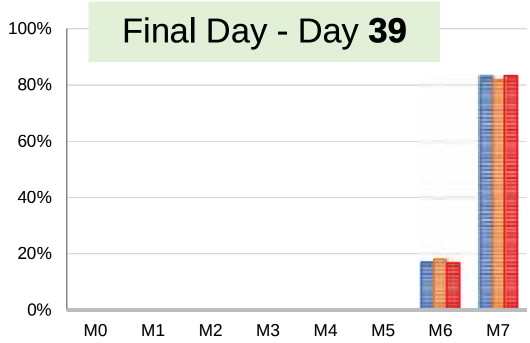


## Fractional $^{13}$C-enrichment

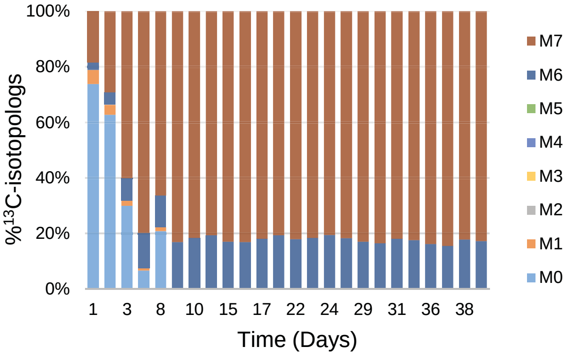


## $^{13}$C-enrichment kinetics

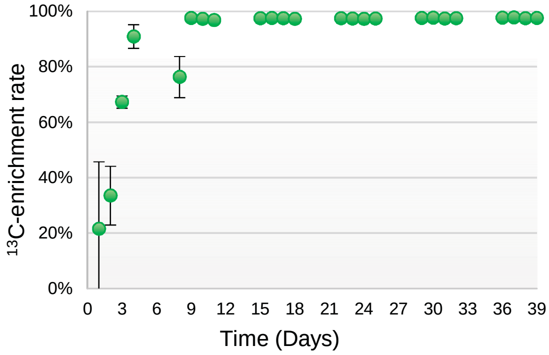

# Identified metabolites in murine urine

*Metabolites from the chemical library*

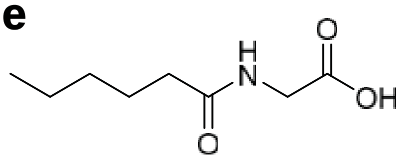


| | |
|---|---|
| **Metabolite** | **Hexanoyl-glycine**<br>C5H11-CO-Glycine |
| **Formula** | C8H15NO3 |
| **Exact mass** | 173.1052 |

| | |
|---|---|
| **Ion type** | [M-H]- |
| **m/z** | 172.0979 |

## Isotopic patterns

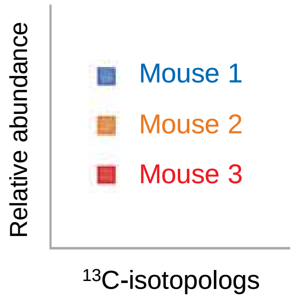


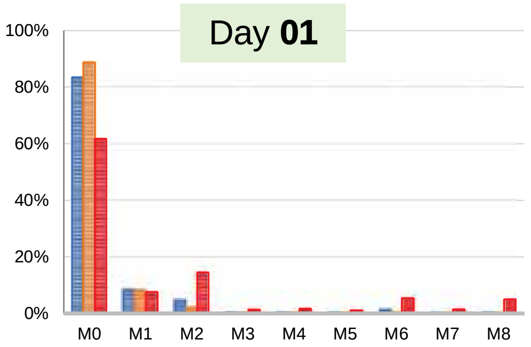


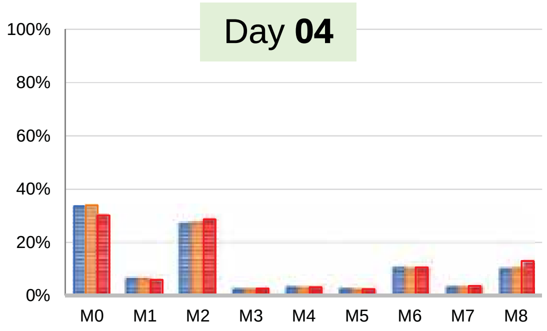


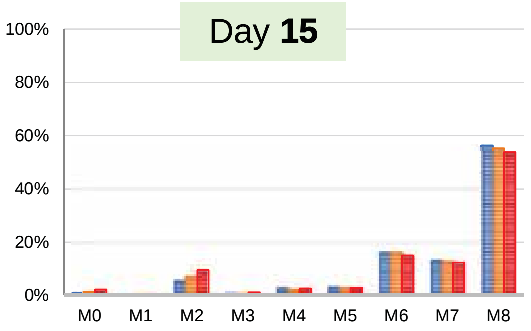


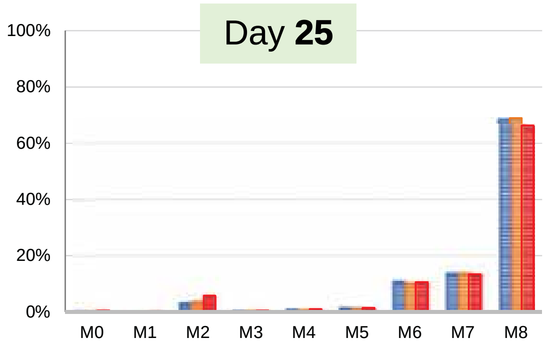


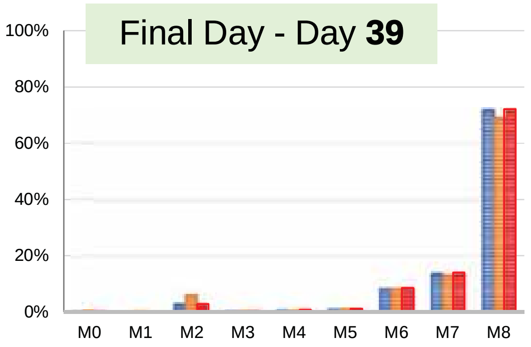


## Fractional $^{13}$C-enrichment

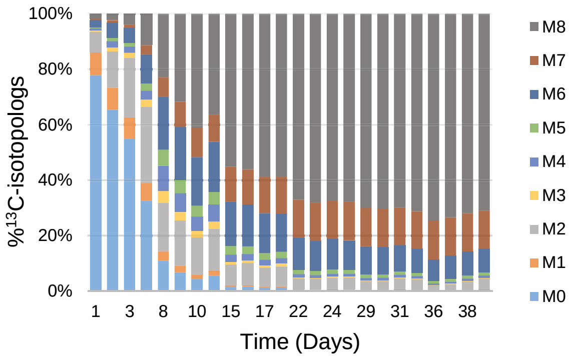


## $^{13}$C-enrichment kinetics

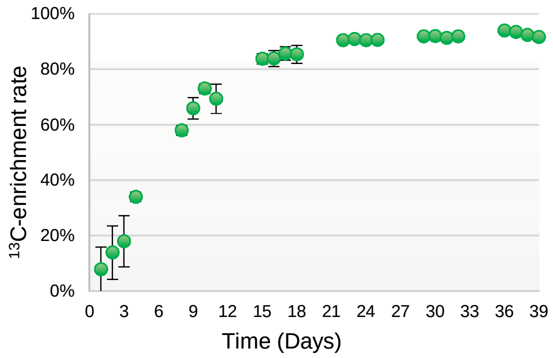

# Identified metabolites in murine urine

*Metabolites from the chemical library*

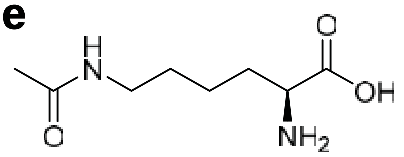

| Metabolite | N6-Acetyl-L-lysine |
|---|---|
| Formula | C8H16N2O3 |
| Exact mass | 188.1161 |

| Ion type | [M+H]+ |
|---|---|
| m/z | 189.1234 |

## Isotopic patterns

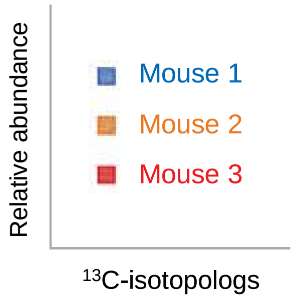


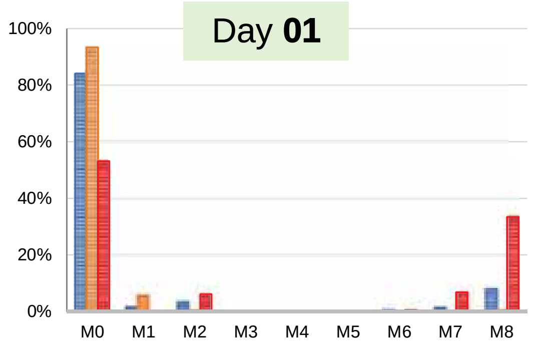


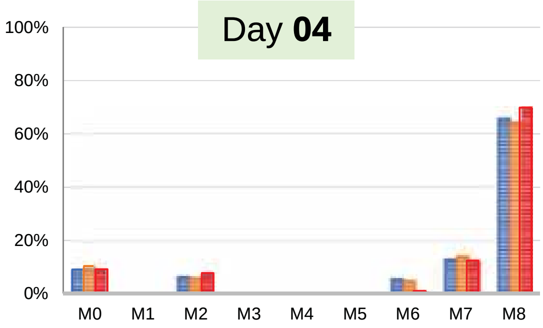


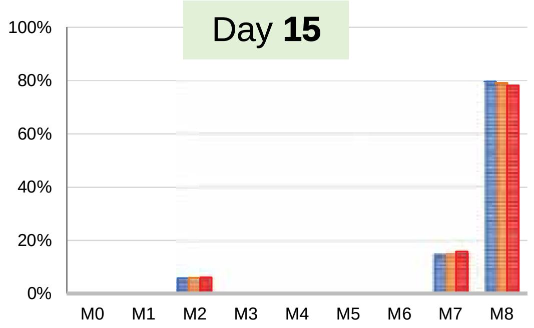


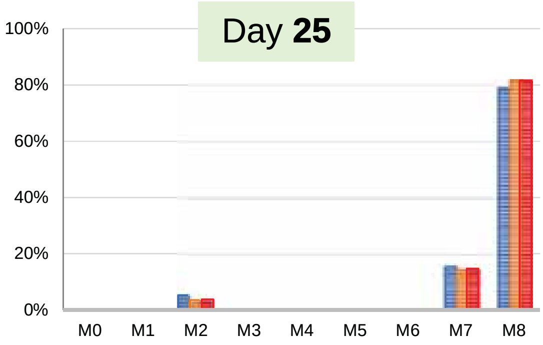


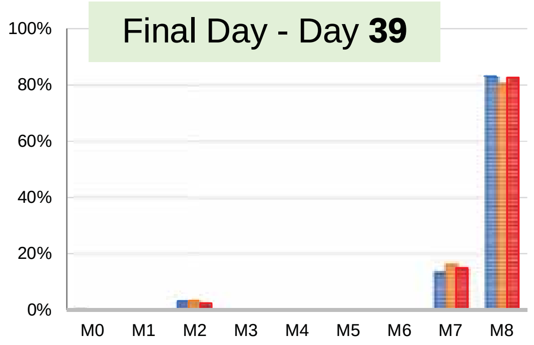


## Fractional $^{13}$C-enrichment

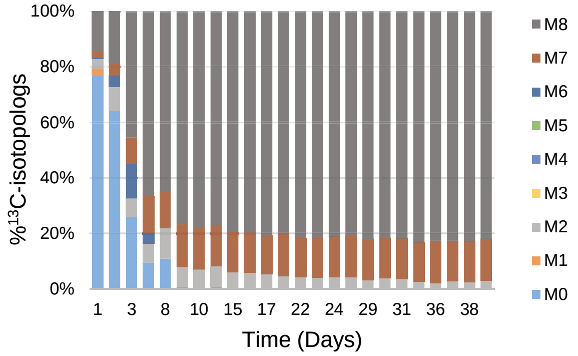


## $^{13}$C-enrichment kinetics

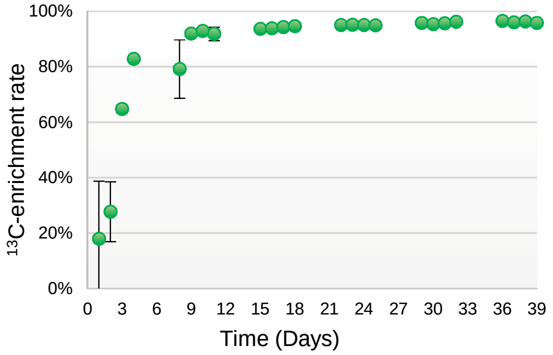

# Identified metabolites in murine urine

*Metabolites from the chemical library*

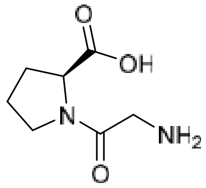


| **Metabolite** | **Gly-Pro** |
|---|---|
| **Formula** | C7H12N2O3 |
| **Exact mass** | 172.0848 |

| **Ion type** | [M+H]+ |
|---|---|
| **m/z** | 173.0921 |

## Isotopic patterns

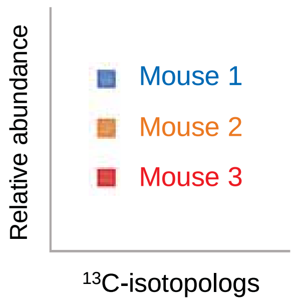


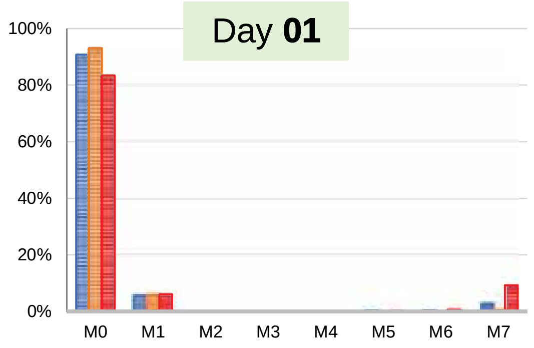


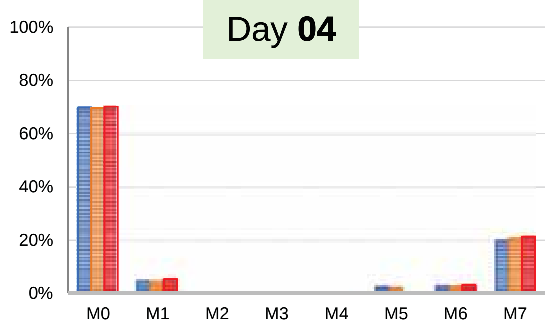


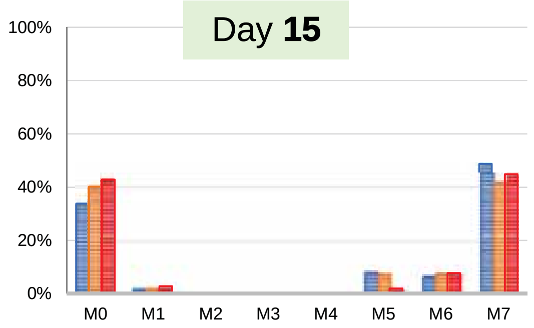


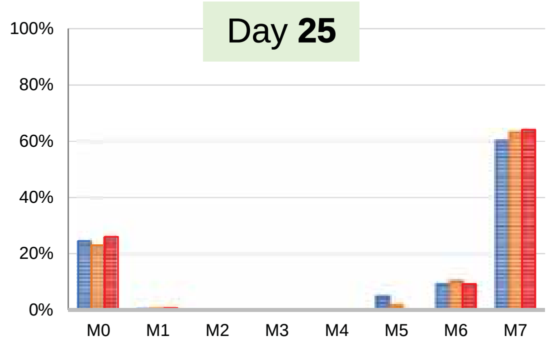


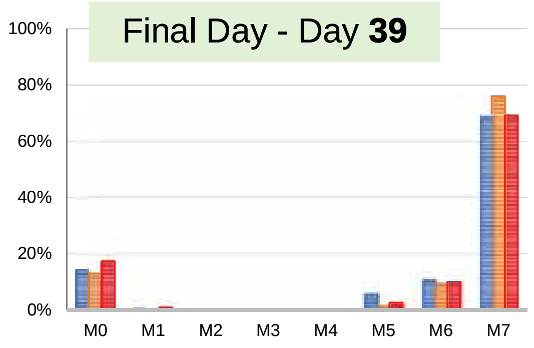


## Fractional $^{13}$C-enrichment

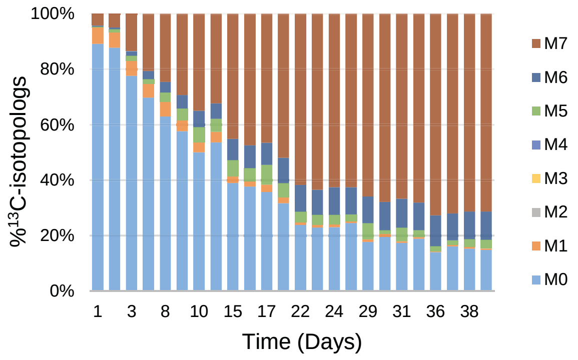


## $^{13}$C-enrichment kinetics

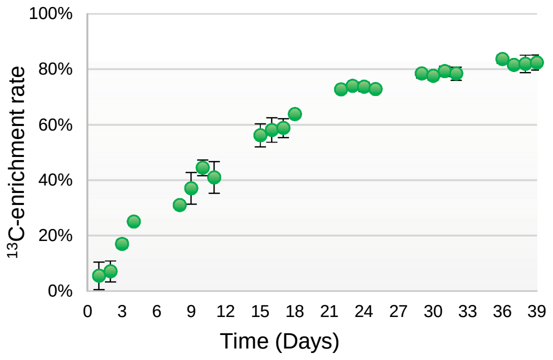

# Identified metabolites in murine urine

*Metabolites from the chemical library*

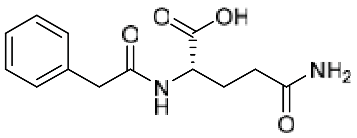


| **Metabolite** | **Phenylacetyl-L-glutamine** |
|---|---|
| **Formula** | C13H16N2O4 |
| **Exact mass** | 264.111 |

| **Ion type** | [M+H]+ |
|---|---|
| **m/z** | 265.1183 |

## Isotopic patterns

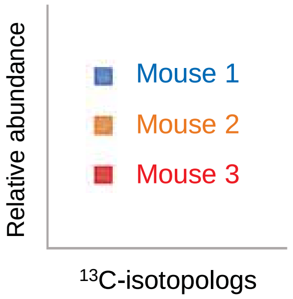


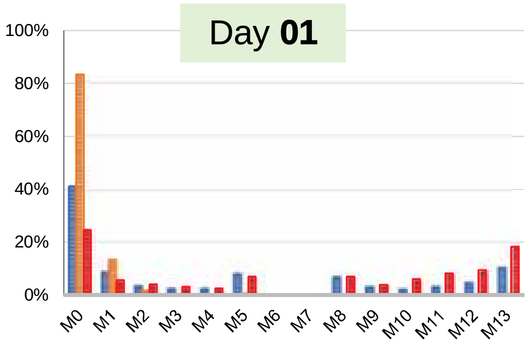


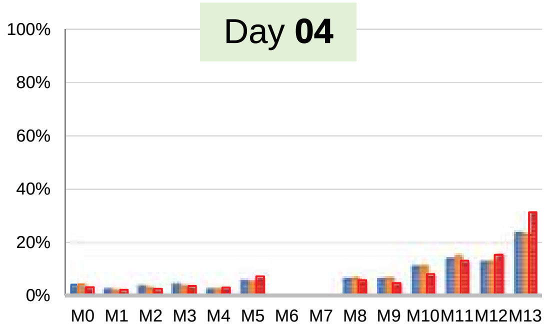


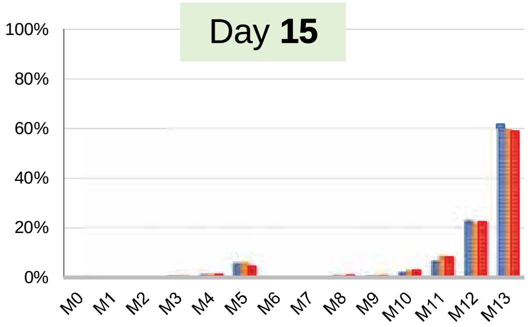


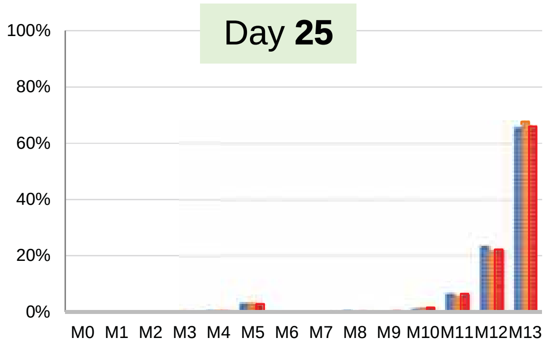


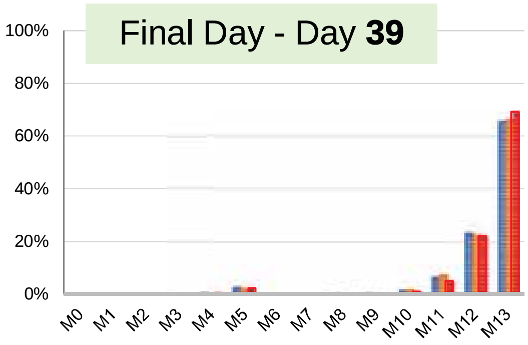


## Fractional $^{13}$C-enrichment

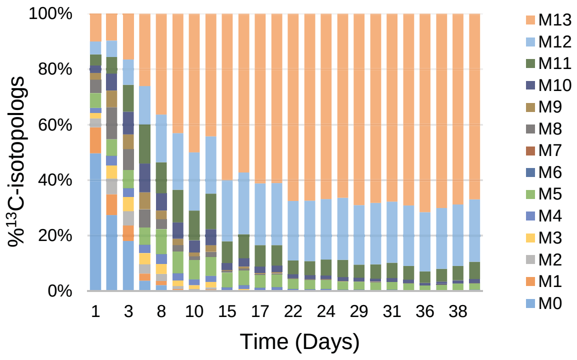


## $^{13}$C-enrichment kinetics

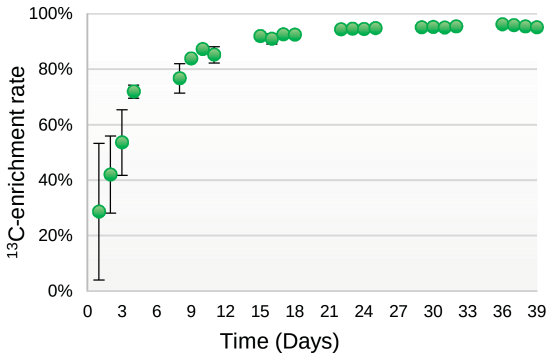

# Identified metabolites in murine urine

*Metabolites from the chemical library*

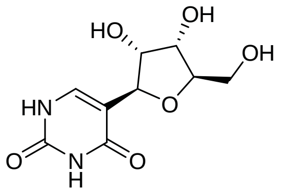


| Metabolite | beta-Pseudouridine |
|---|---|
| Formula | C9H12N2O6 |
| Exact mass | 244.0695 |

| Ion type | [M-H]- |
|---|---|
| m/z | 243.0623 |

## Isotopic patterns

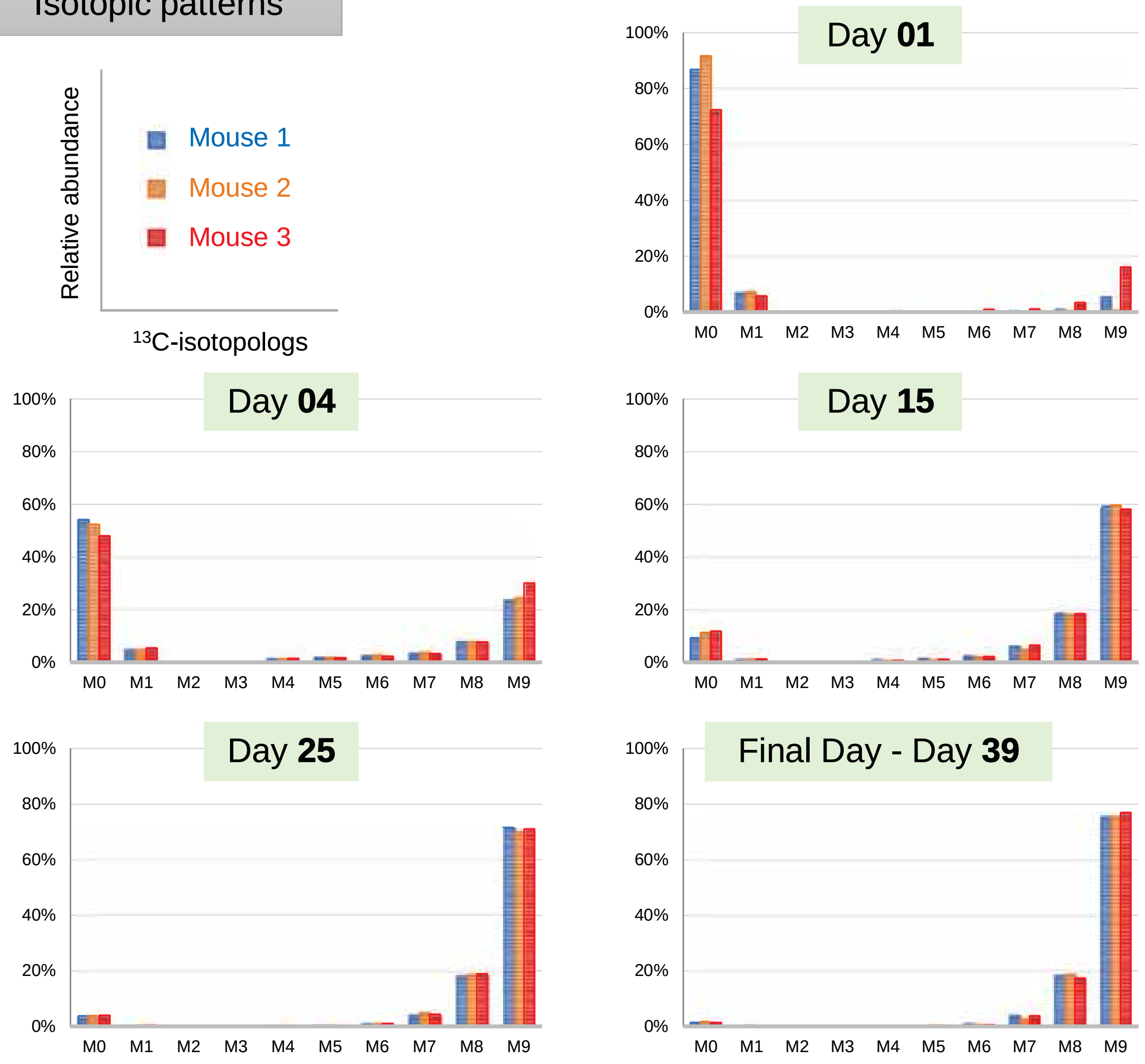


## Fractional $^{13}$C-enrichment

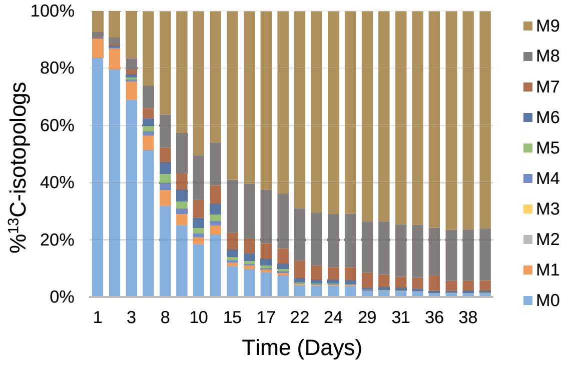


## $^{13}$C-enrichment kinetics

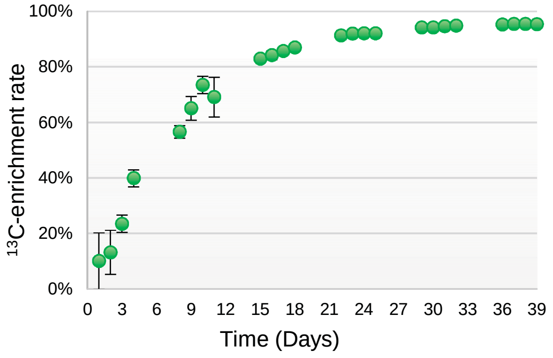

# Identified metabolites in murine urine

*Metabolites from the chemical library*

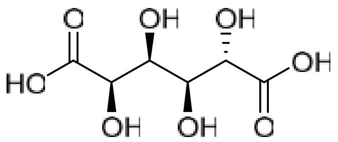


| Metabolite | D-Saccharic acid |
|---|---|
| Formula | C6H10O8 |
| Exact mass | 210.0376 |

| Ion type | [M-H]- |
|---|---|
| m/z | 209.0303 |

## Isotopic patterns

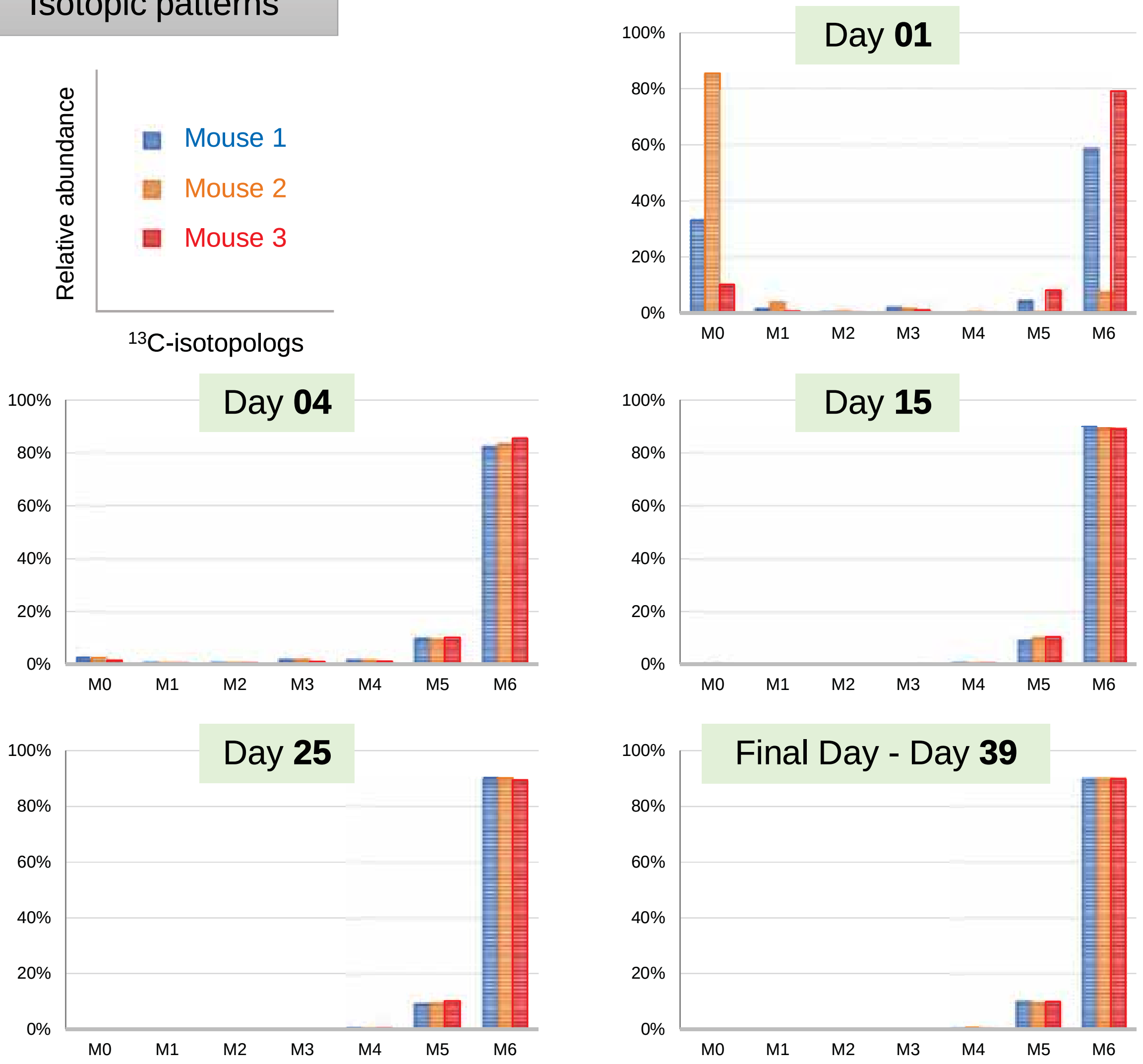

# Identified metabolites in murine urine

*Metabolites from the chemical library*

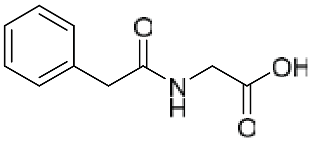


| | |
|---|---|
| **Metabolite** | **Phenylacetylglycine**<br>C7H7-CO-Glycine |
| **Formula** | C10H11NO3 |
| **Exact mass** | 193.0739 |

| | |
|---|---|
| **Ion type** | [M-H]- |
| **m/z** | 192.0666 |

## Isotopic patterns

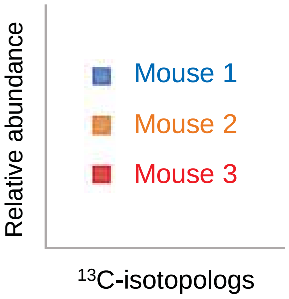


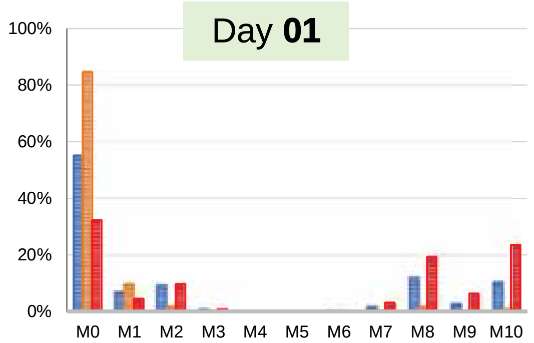


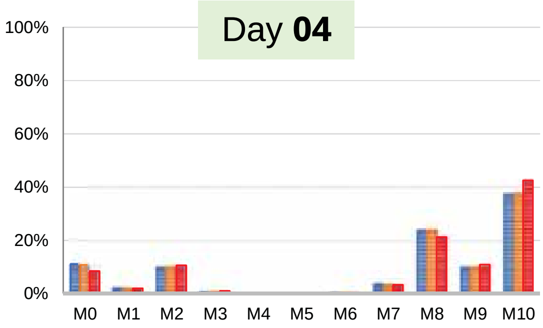


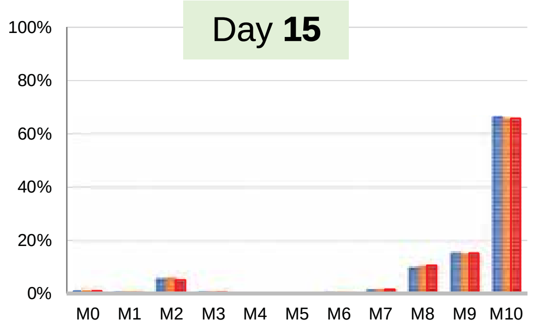


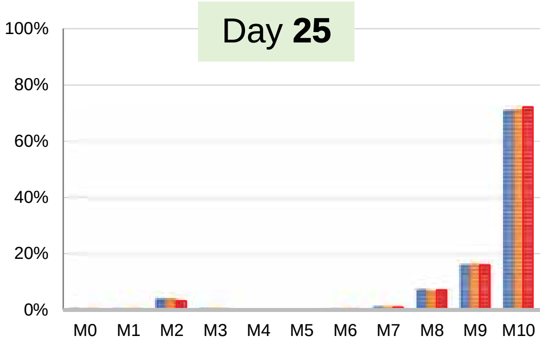


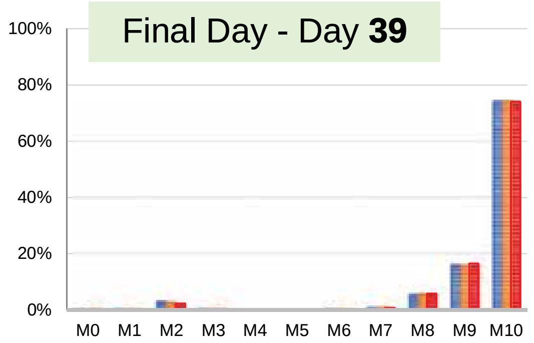


## Fractional $^{13}$C-enrichment

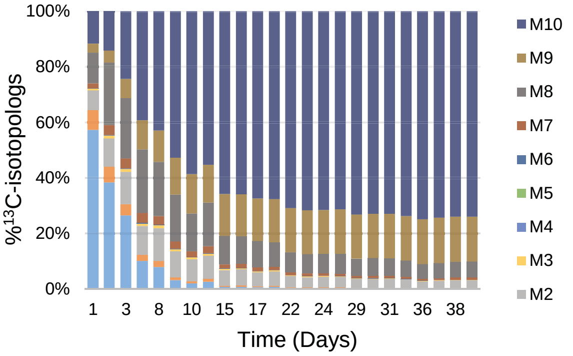


## $^{13}$C-enrichment kinetics

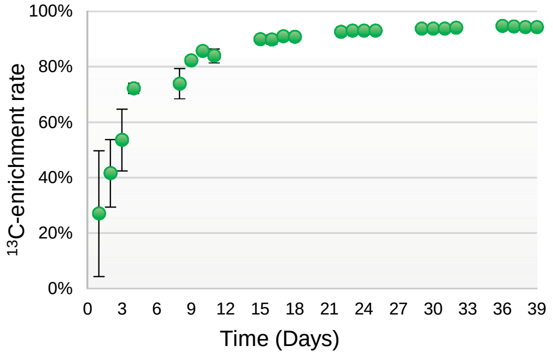

# Identified metabolites in murine urine

*Metabolites from the chemical library*

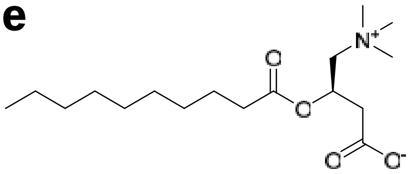


| Metabolite | Decanoylcarnitine<br>C9H19-CO-Carnitine |
|---|---|
| **Formula** | C17H33NO4 |
| **Exact mass** | 315.241 |

| Ion type | [M+H]+ |
|---|---|
| **m/z** | 316.2482 |

## Isotopic patterns

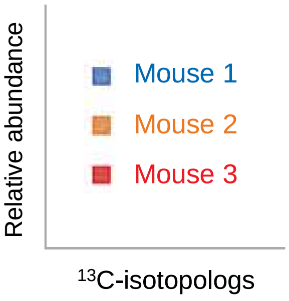


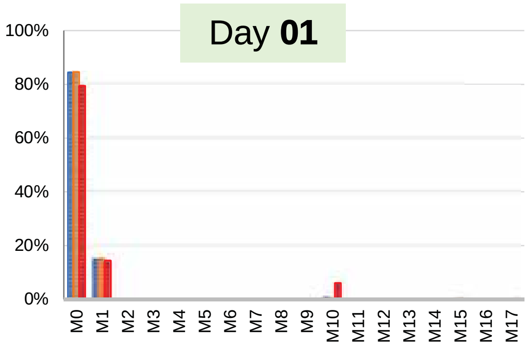


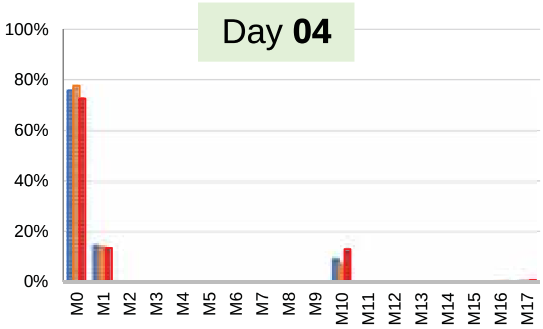


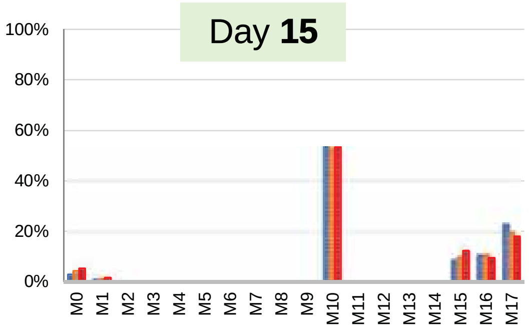


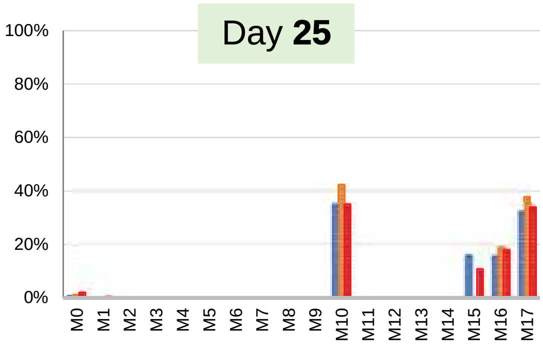


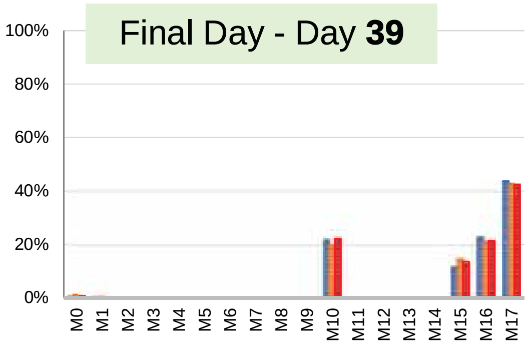


## Fractional $^{13}$C-enrichment

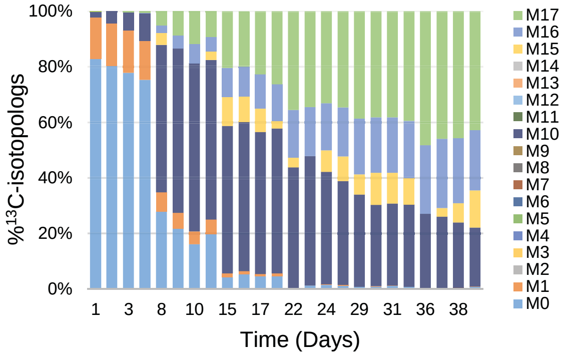


## $^{13}$C-enrichment kinetics

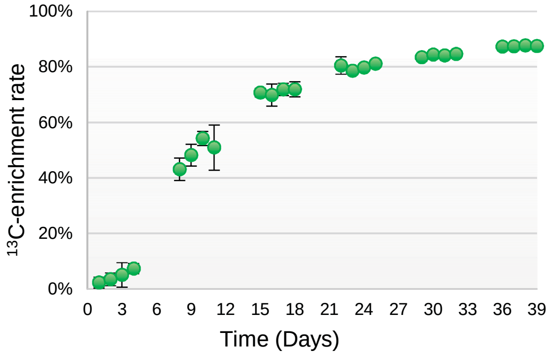

# Identified metabolites in murine urine

*Metabolites from the chemical library*

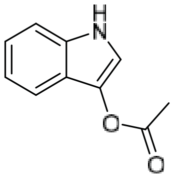

| Metabolite | Indoxyl-acetate |
|---|---|
| Formula | C10H9NO2 |
| Exact mass | 175.0633 |

| Ion type | [M+H]+ |
|---|---|
| m/z | 176.0706 |

## Isotopic patterns

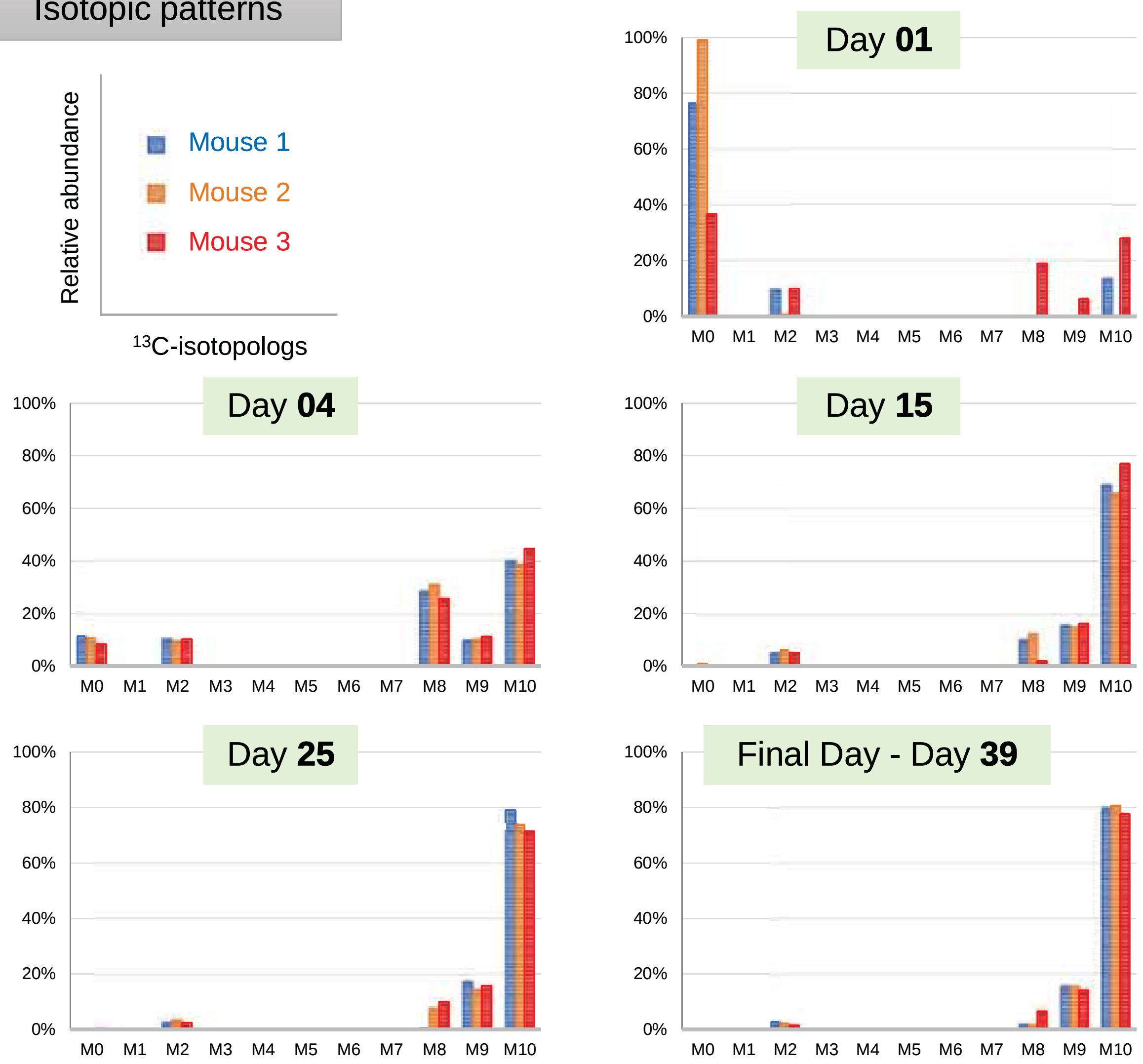


## Fractional $^{13}$C-enrichment

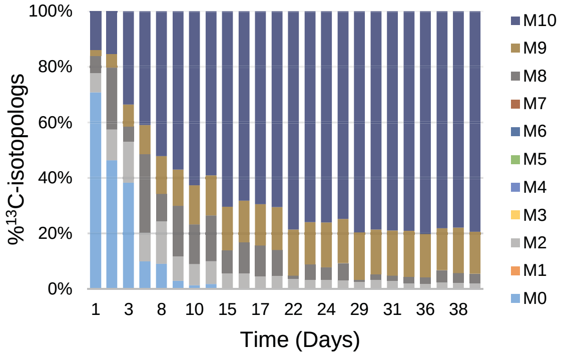


## $^{13}$C-enrichment kinetics

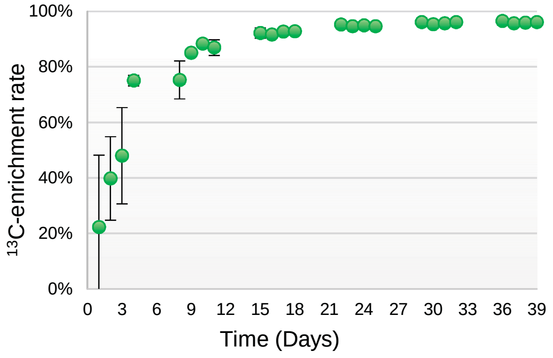

# Identified metabolites in murine urine

*Metabolites from the chemical library*

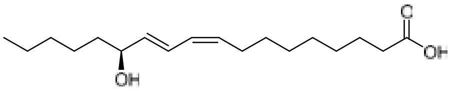


| | |
|---|---|
| **Metabolite** | **13(S)-Hydroxyoctadeca-9Z-11E-dienoic acid** |
| **Formula** | C18H32O3 |
| **Exact mass** | 296.2351 |

| | |
|---|---|
| **Ion type** | [M-H]- |
| **m/z** | 295.2279 |

## Isotopic patterns

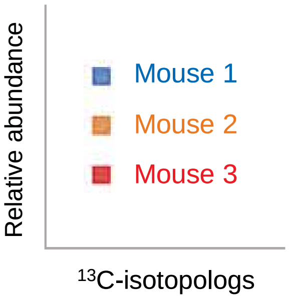


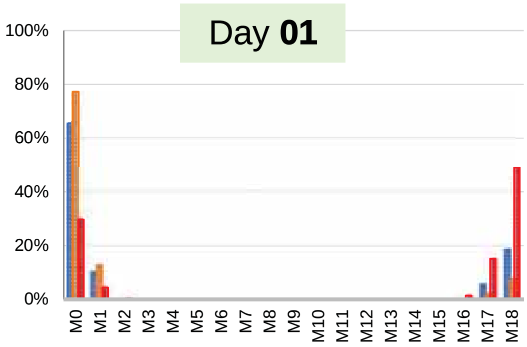


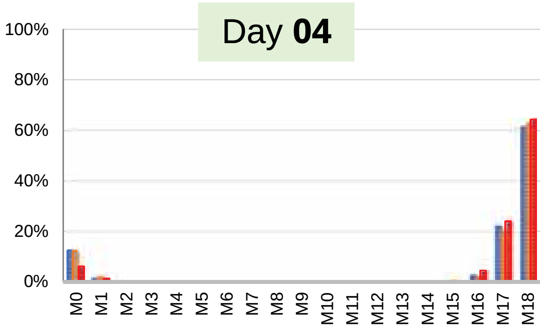


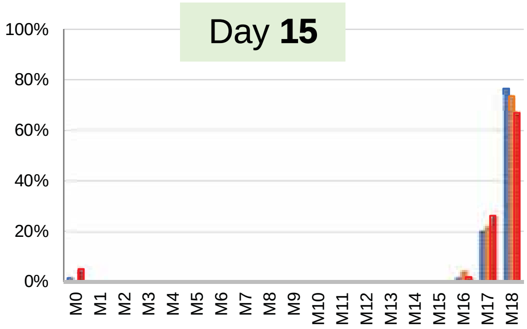


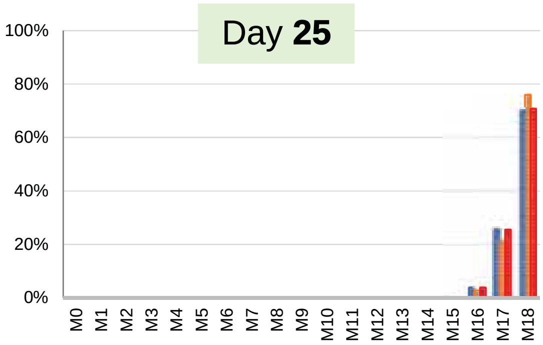


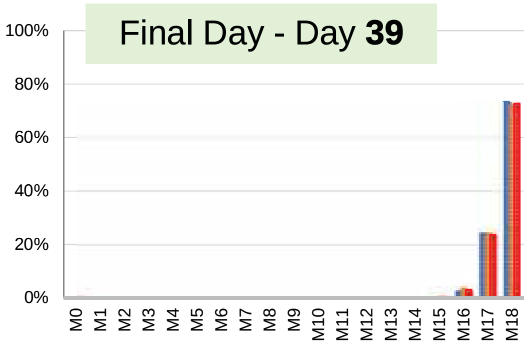

# Identified metabolites in murine urine

*Metabolites from the chemical library*

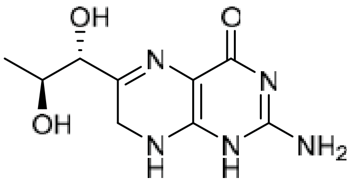


| Metabolite | 7-8-Dihydro-L-biopterin |
|---|---|
| **Formula** | C9H13N5O3 |
| **Exact mass** | 239.1018 |

| Ion type | [M+H]+ |
|---|---|
| **m/z** | 240.1091 |

## Isotopic patterns

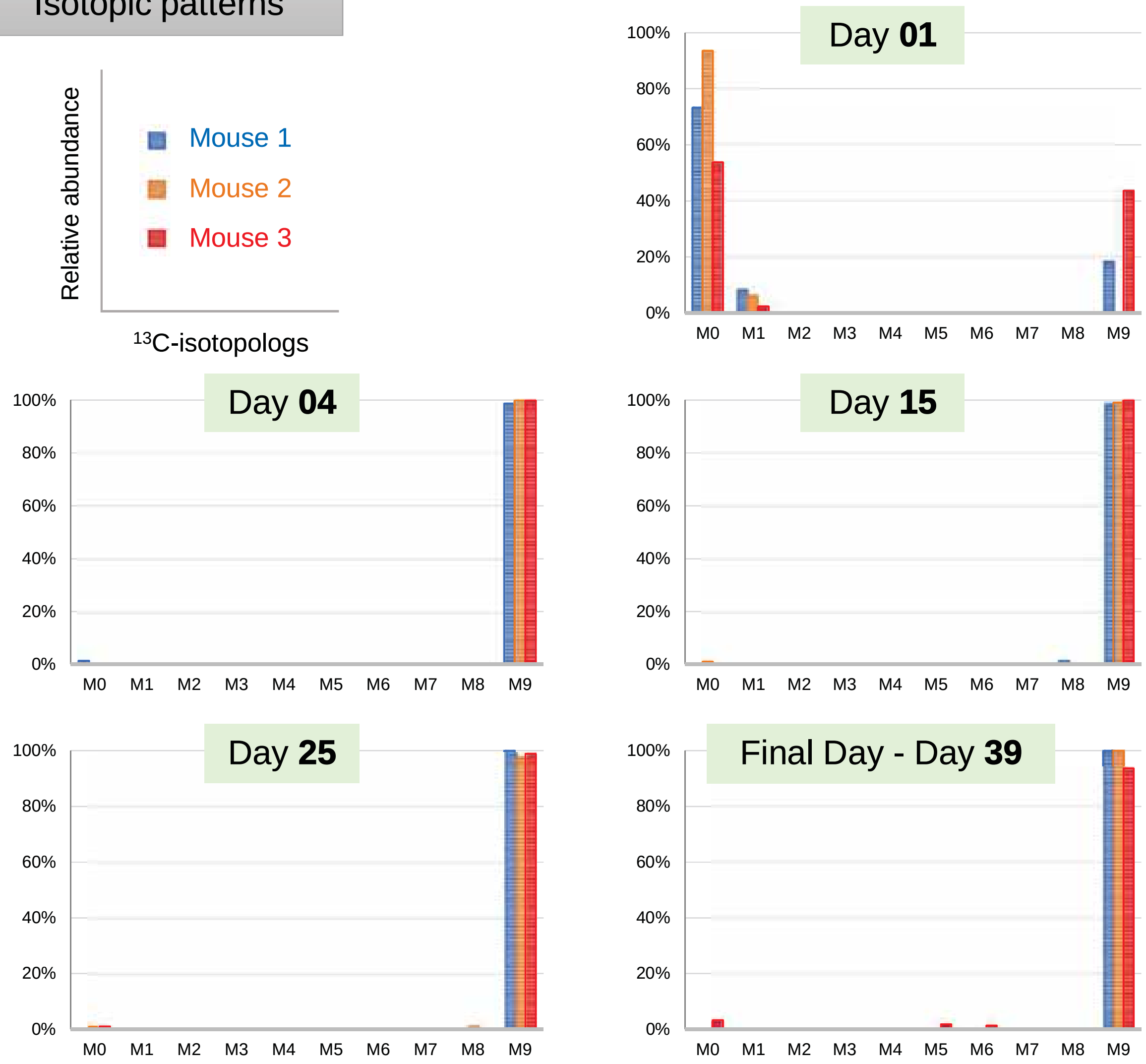

# Identified metabolites in murine urine

*Metabolites from the chemical library*

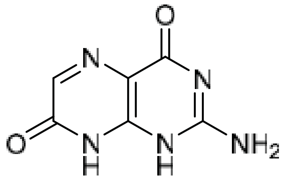


| Metabolite | Isoxanthopterin |
|---|---|
| Formula | C6H5N5O2 |
| Exact mass | 179.0443 |

| Ion type | [M-H]- |
|---|---|
| m/z | 178.037 |

## Isotopic patterns

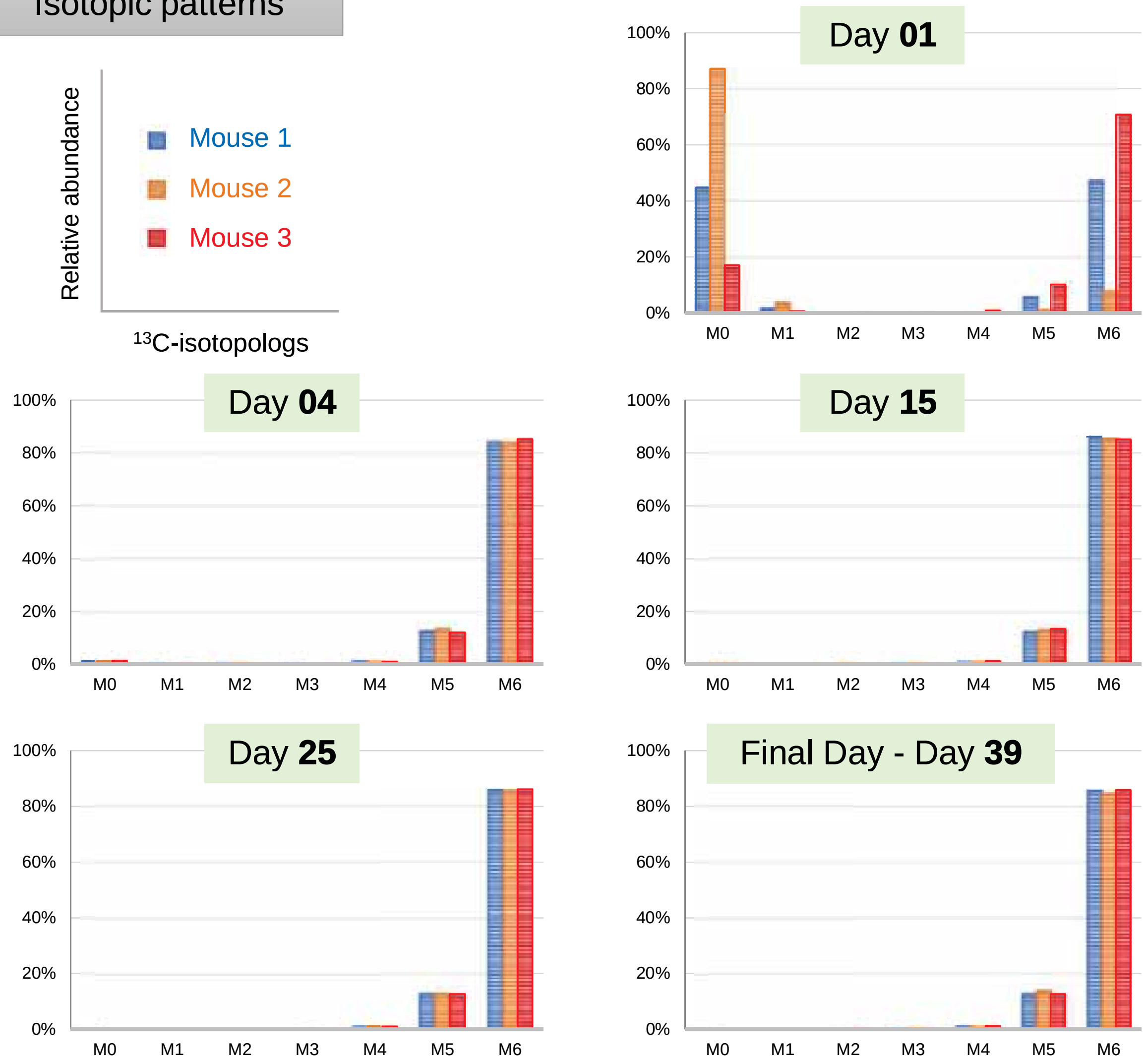

# Identified metabolites in murine urine

*Metabolites from the chemical library*

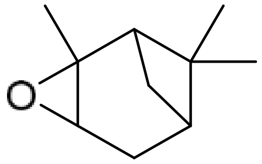


| | |
|---|---|
| **Metabolite** | **a-Pinene-oxide** |
| **Formula** | C10H16O |
| **Exact mass** | 152.1201 |

| | |
|---|---|
| **Ion type** | [M+H]+ |
| **m/z** | 153.1274 |

## Isotopic patterns

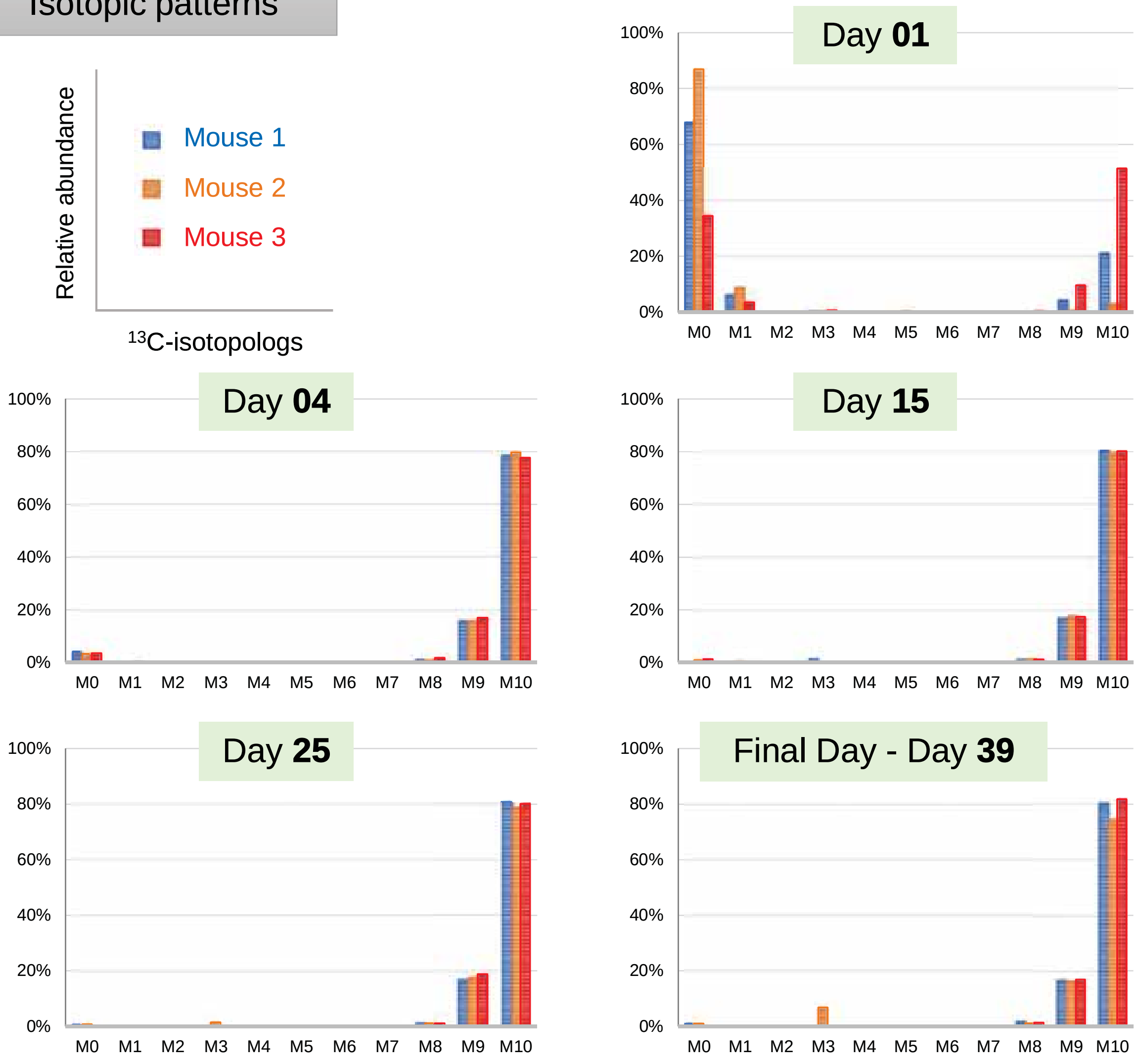

# Identified metabolites in murine urine

*Metabolites from the chemical library*

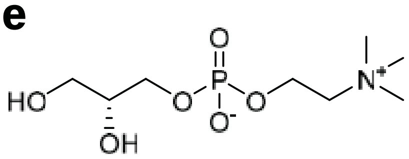


| Metabolite | L-a-Glycerophosphorylcholine |
|---|---|
| Formula | C8H20NO6P |
| Exact mass | 257.1028 |

| Ion type | [M+H]+ |
|---|---|
| m/z | 258.1101 |

## Isotopic patterns

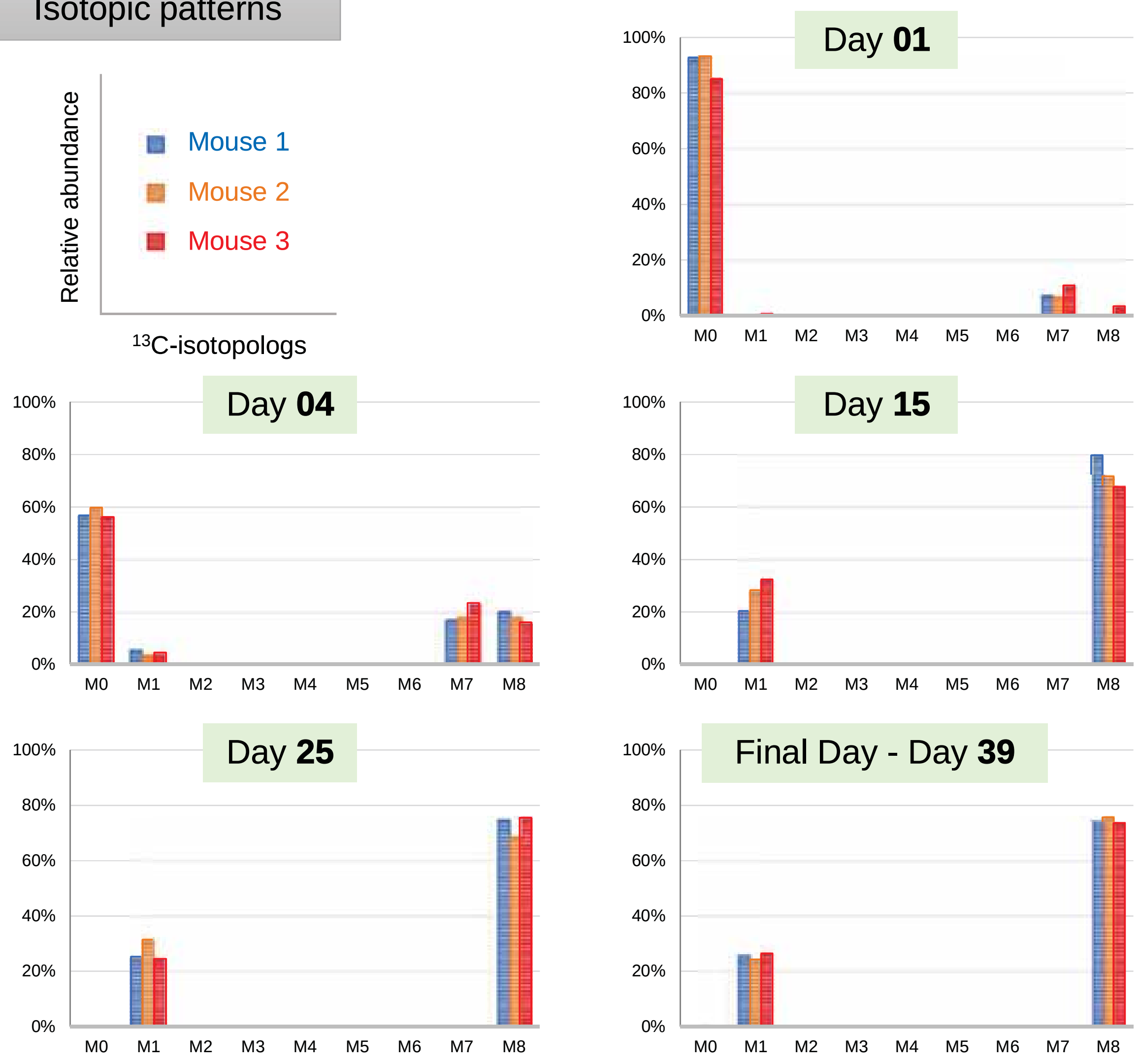


## Fractional $^{13}$C-enrichment

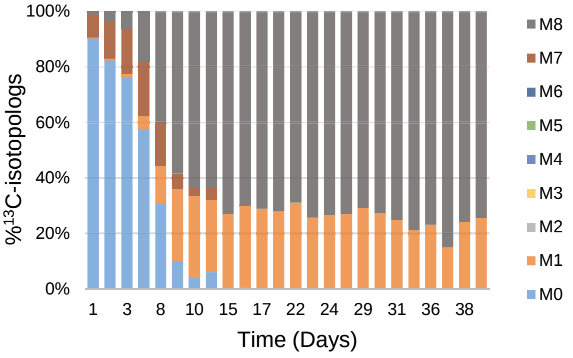


## $^{13}$C-enrichment kinetics

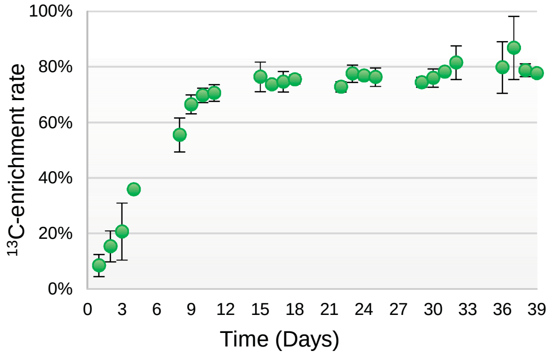

# Identified metabolites in murine urine

*Metabolites from the chemical library*

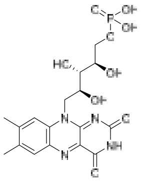

| **Metabolite** | **Riboflavin-5-monophosphate** |
| --- | --- |
| **Formula** | C17H21N4O9P |
| **Exact mass** | 456.1046 |

| **Ion type** | [M+H]+ |
| --- | --- |
| **m/z** | 457.1119 |

## Isotopic patterns

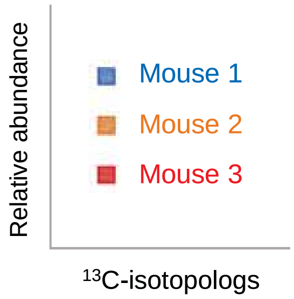


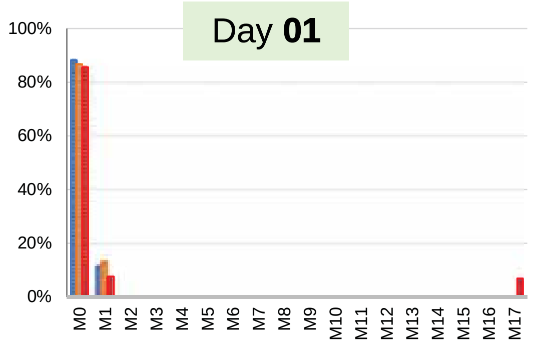


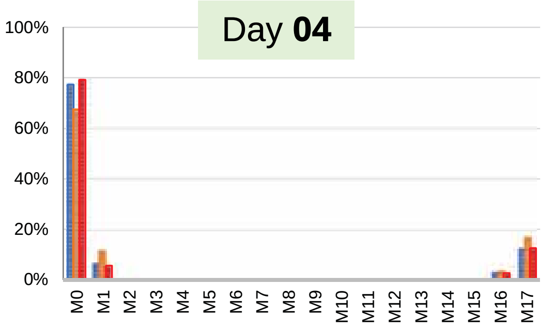


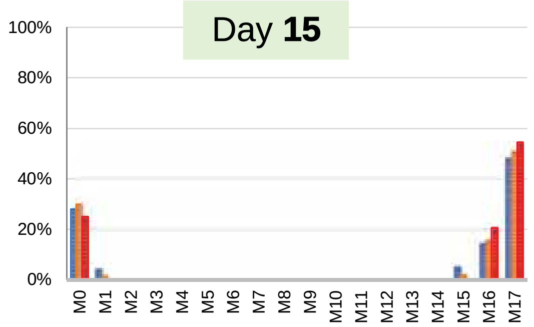


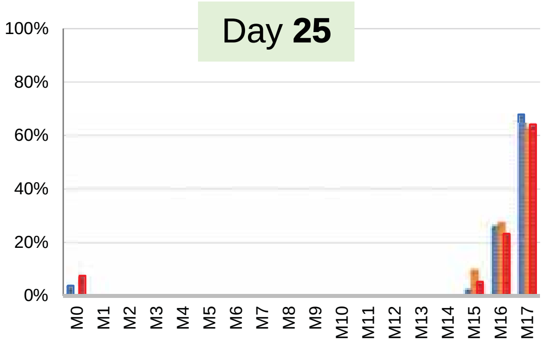


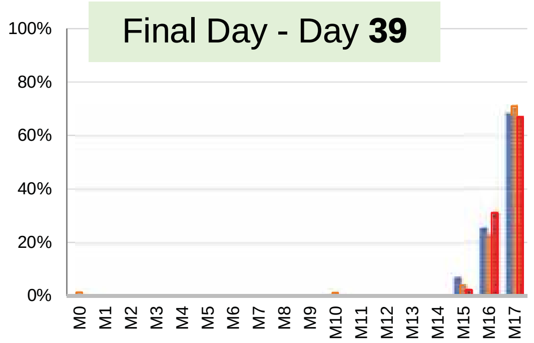

# Identified metabolites in murine urine

*Metabolites from the chemical library*

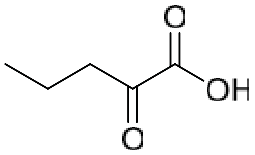


| Metabolite | 2-Oxovaleric acid |
|---|---|
| Formula | C5H8O3 |
| Exact mass | 116.0473 |

| Ion type | [M-H]- |
|---|---|
| m/z | 115.0401 |

## Isotopic patterns

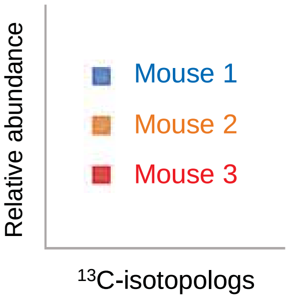


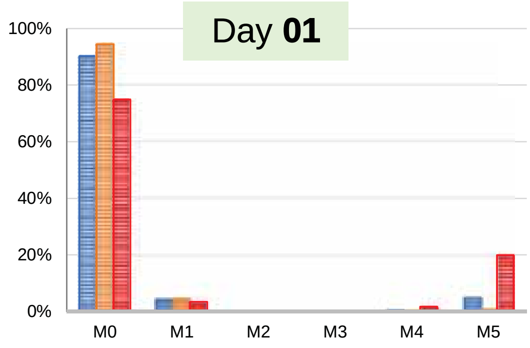


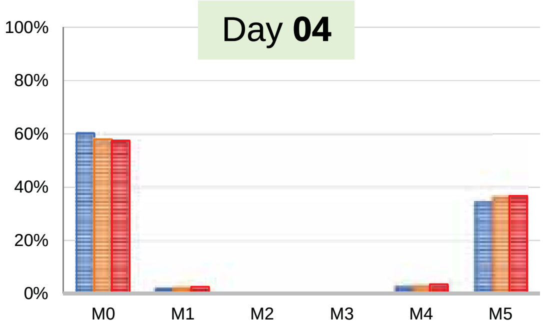


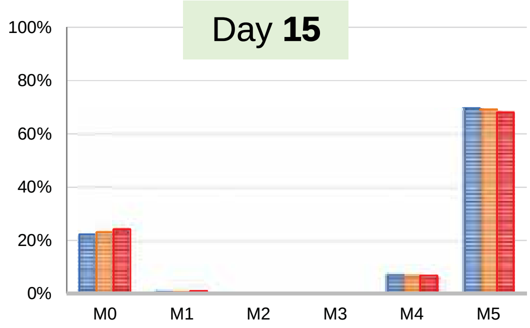


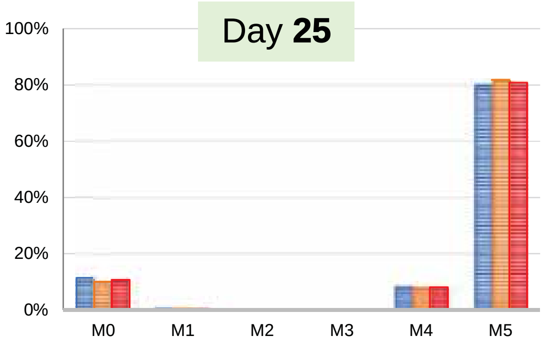


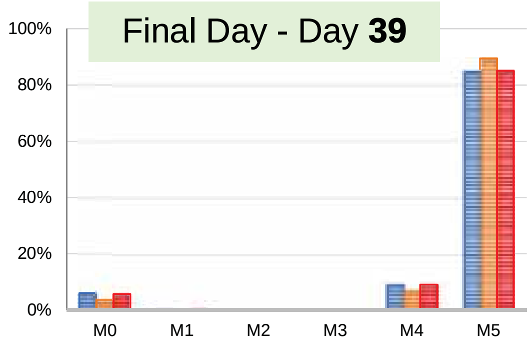


## Fractional $^{13}$C-enrichment

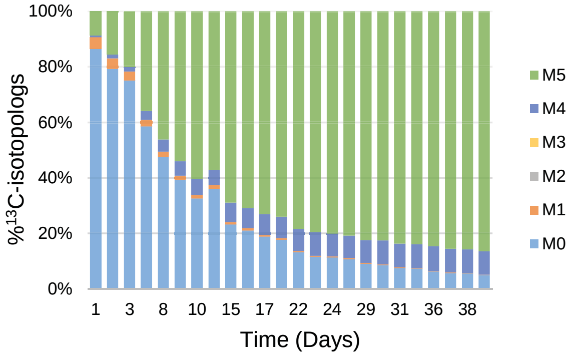


## $^{13}$C-enrichment kinetics

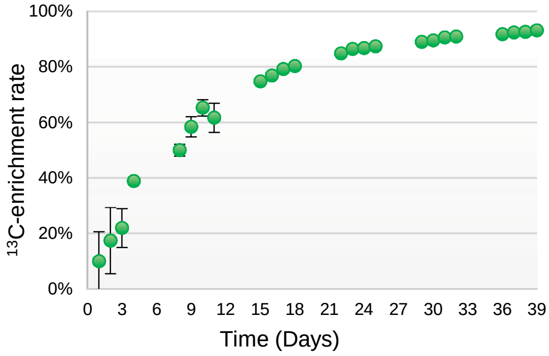

# Identified metabolites in murine urine

*Metabolites from the chemical library*

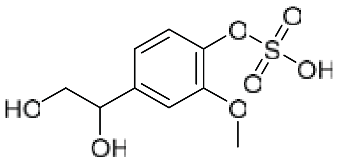


| **Metabolite** | **4-Hydroxy-3-methoxyphenylglycol-sulfate** |
|---|---|
| **Formula** | C9H12O7S |
| **Exact mass** | 264.0304 |

| **Ion type** | [M-H]- |
|---|---|
| **m/z** | 263.0231 |

## Isotopic patterns

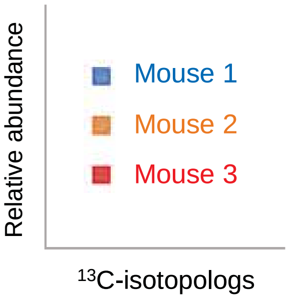


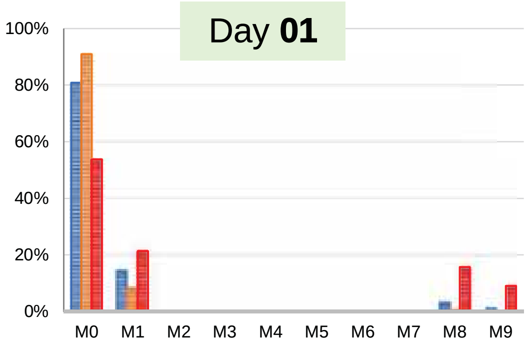


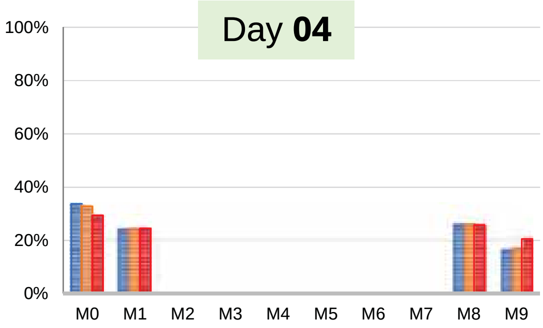


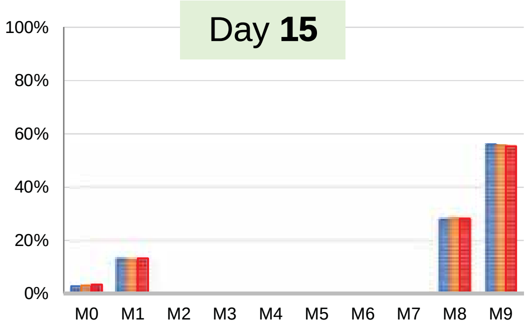


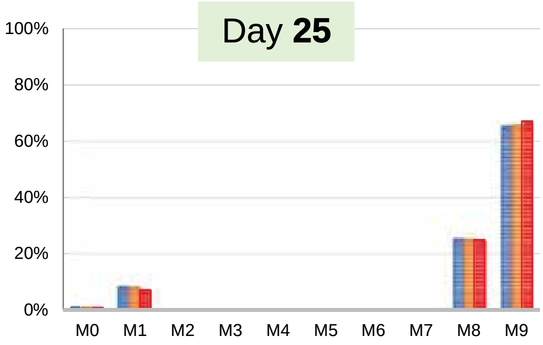


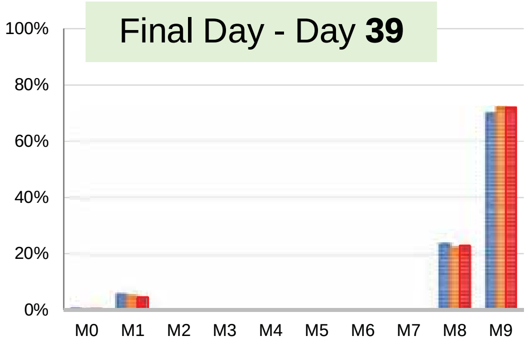


## Fractional $^{13}$C-enrichment

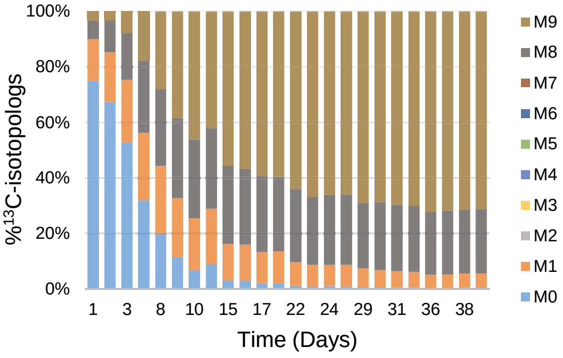


## $^{13}$C-enrichment kinetics

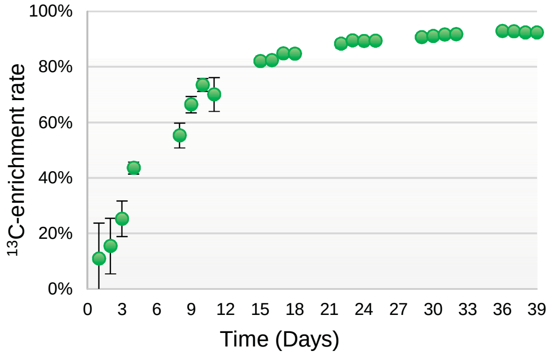

# Identified metabolites in murine urine

*Metabolites from the chemical library*

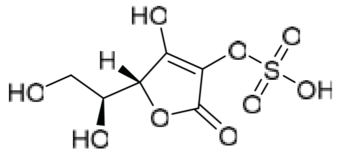


| Metabolite | L-Ascorbic acid-2-sulfate |
|---|---|
| Formula | C6H8O9S |
| Exact mass | 255.9889 |

| Ion type | [M-H]- |
|---|---|
| m/z | 254.9816 |

## Isotopic patterns

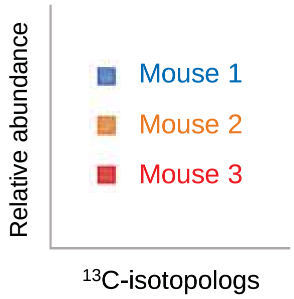


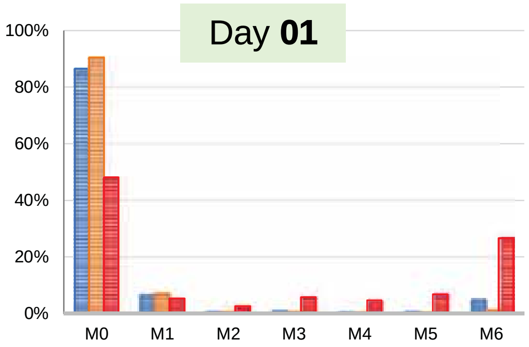


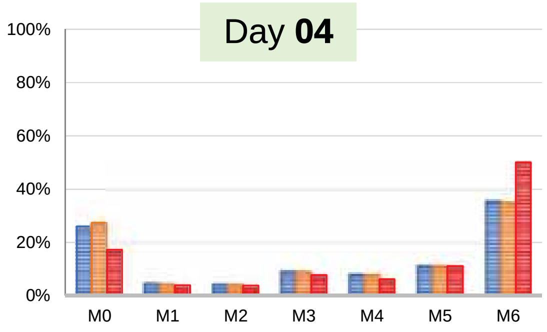


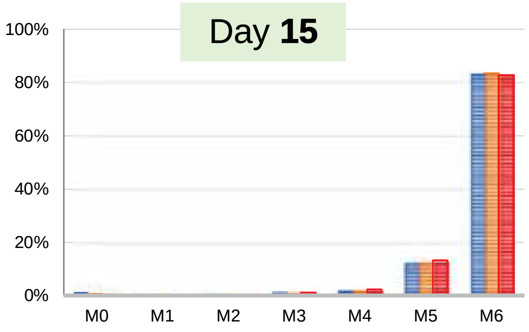


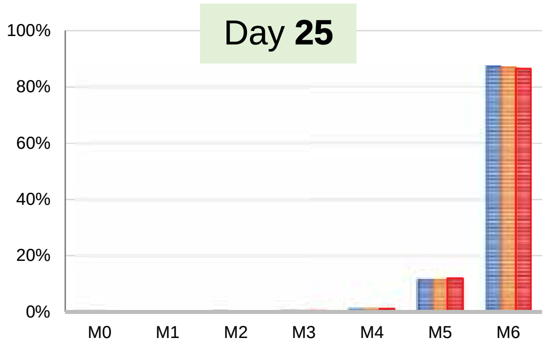


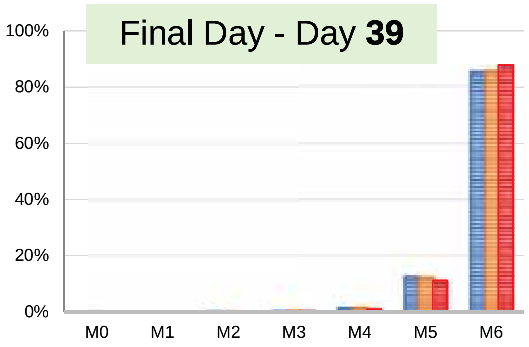


## Fractional $^{13}$C-enrichment

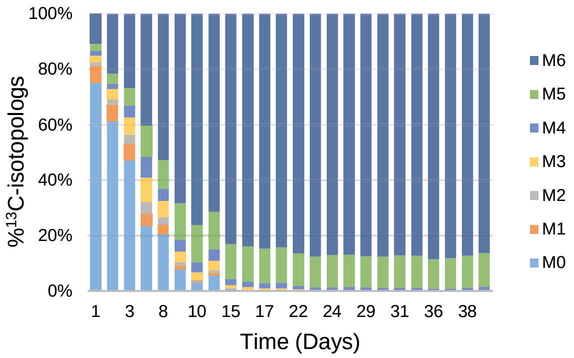


## $^{13}$C-enrichment kinetics

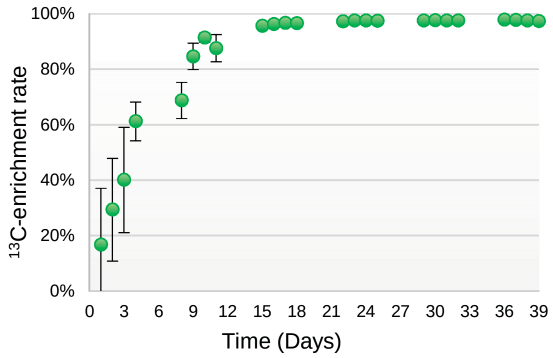

# Identified metabolites in murine urine

*Metabolites from the chemical library*

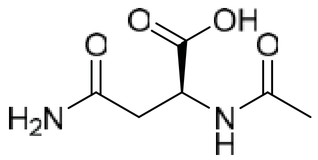

| **Metabolite** | **Nalpha-Acetyl-L-asparagine** |
|---|---|
| **Formula** | C6H10N2O4 |
| **Exact mass** | 174.0641 |

| **Ion type** | [M-H]- |
|---|---|
| **m/z** | 173.0568 |

## Isotopic patterns

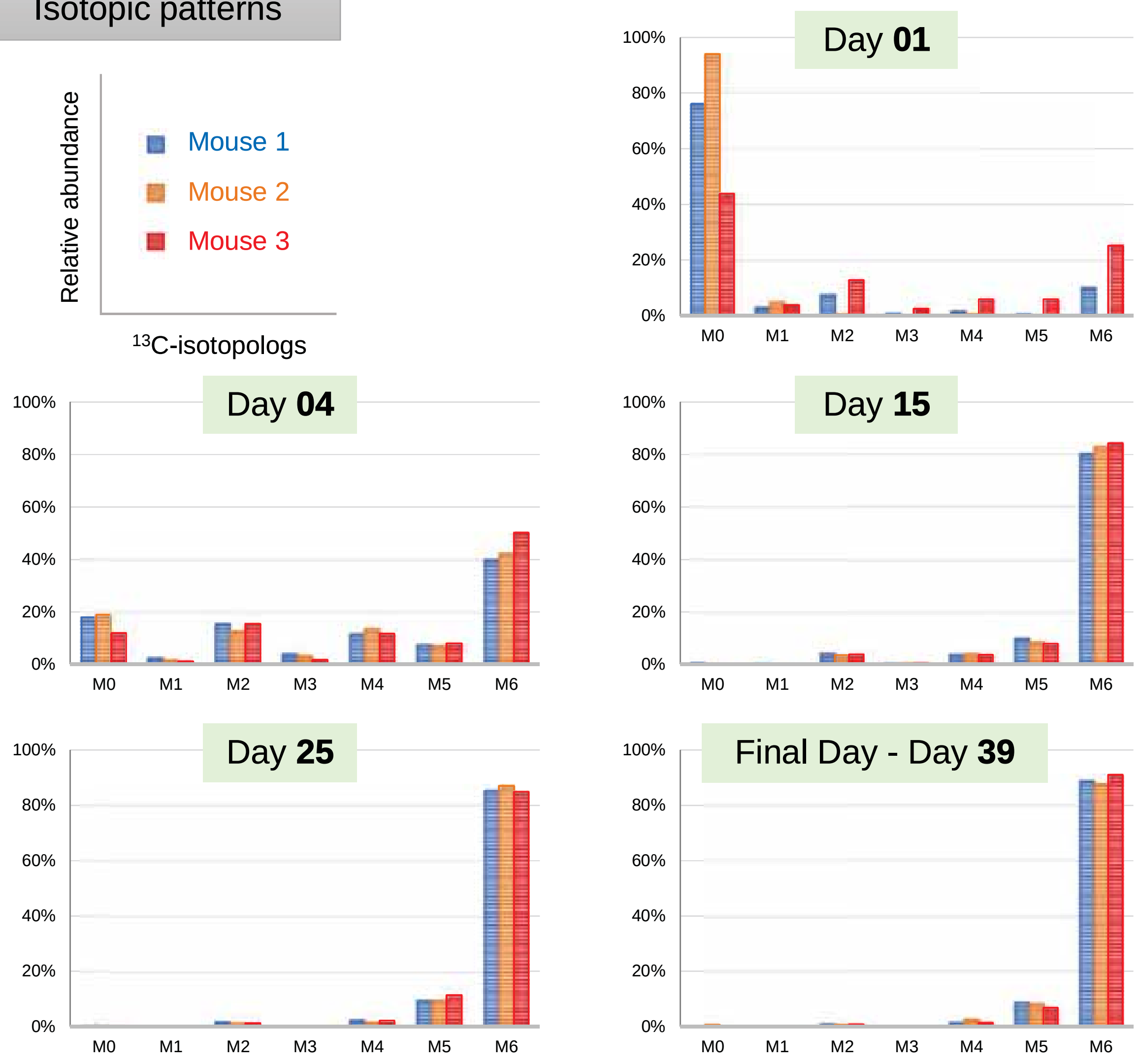

# Identified metabolites in murine urine

*Metabolites from the chemical library*

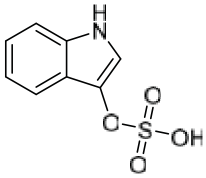


| Metabolite | Indoxyl-sulfate |
|---|---|
| Formula | C8H7NO4S |
| Exact mass | 213.0096 |

| Ion type | [M-H]- |
|---|---|
| m/z | 212.0018 |

## Isotopic patterns

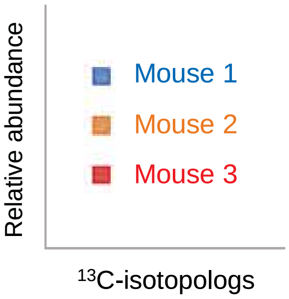


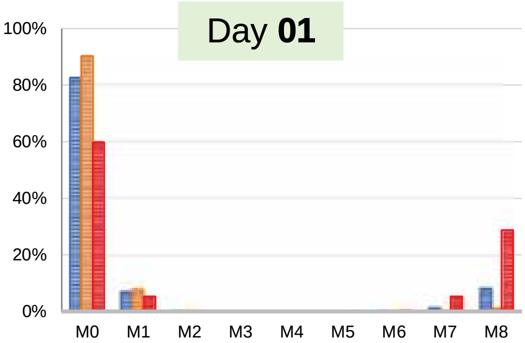


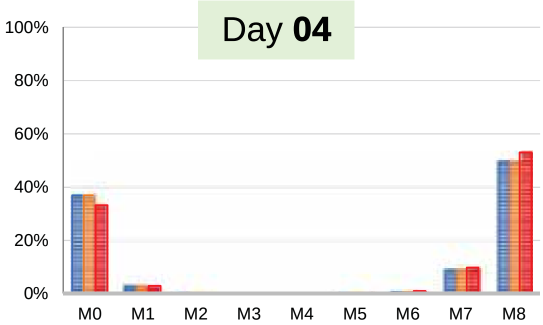


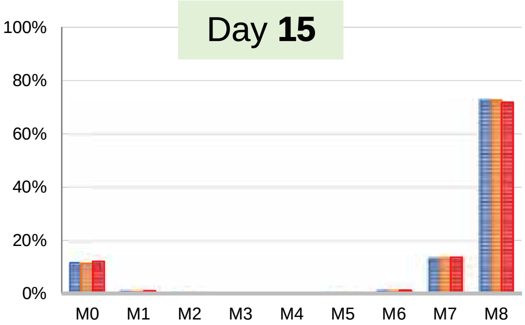


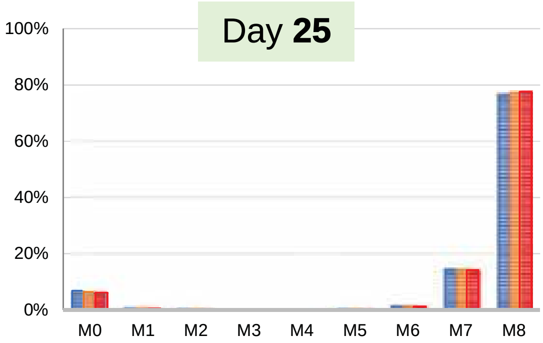


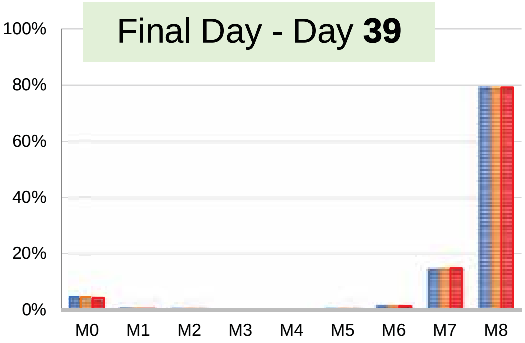


## Fractional $^{13}$C-enrichment

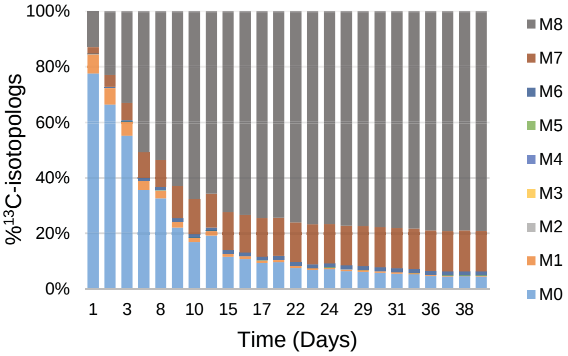


## $^{13}$C-enrichment kinetics

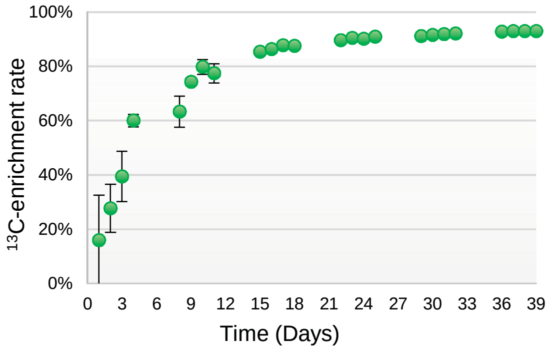

# Identified metabolites in murine urine

*Metabolites from the chemical library*

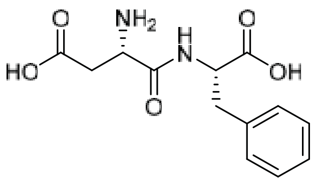


| Metabolite | Asp-Phe |
|---|---|
| Formula | C13H16N2O5 |
| Exact mass | 280.1059 |

| Ion type | [M+H]+ |
|---|---|
| m/z | 281.1132 |

## Isotopic patterns

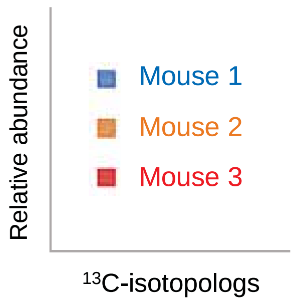


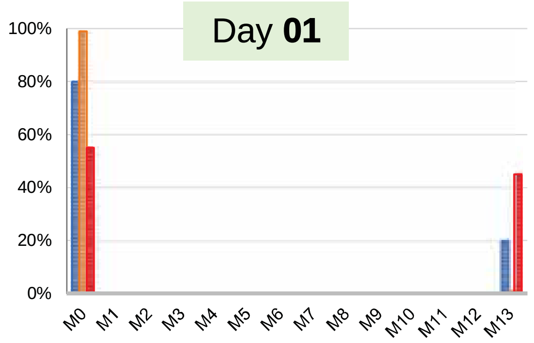


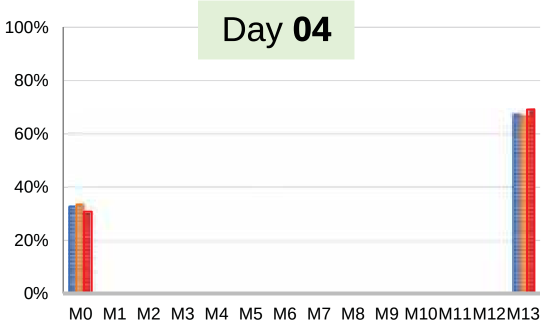


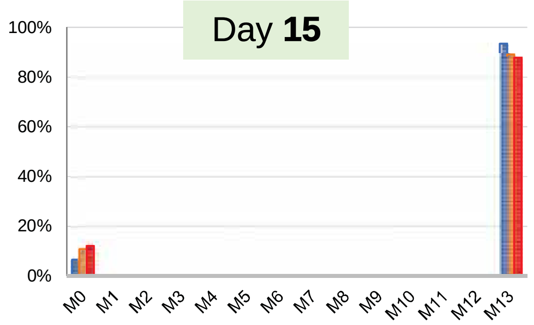


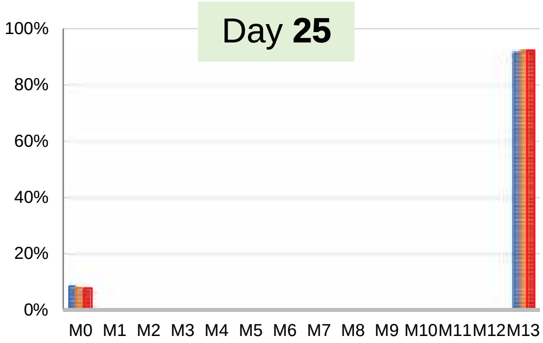


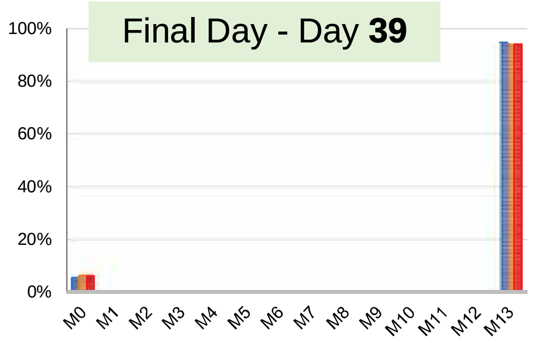


## Fractional $^{13}$C-enrichment

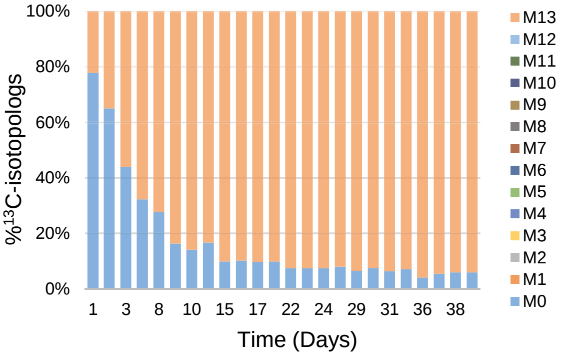


## $^{13}$C-enrichment kinetics

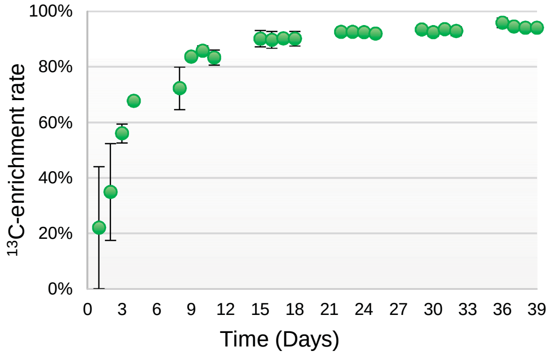

# Identified metabolites in murine urine

*Metabolites from the chemical library*

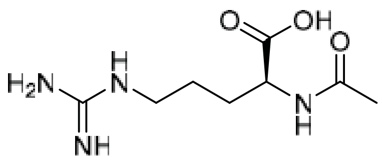

| **Metabolite** | **N-alpha-acetyl-L-arginine** |
|---|---|
| **Formula** | C8H16O3N4 |
| **Exact mass** | 216.1222 |

| **Ion type** | [M+H]+ |
|---|---|
| **m/z** | 217.1295 |

## Isotopic patterns

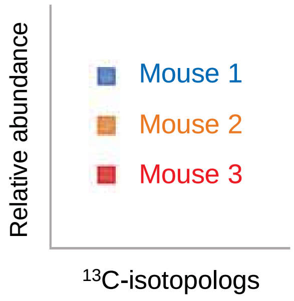


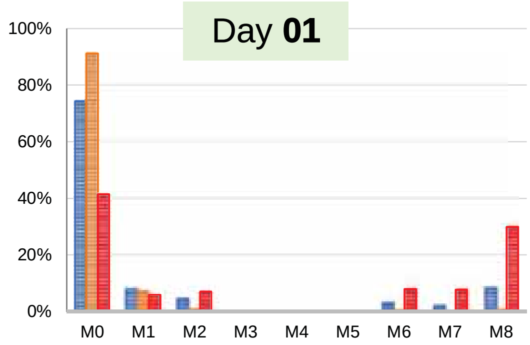


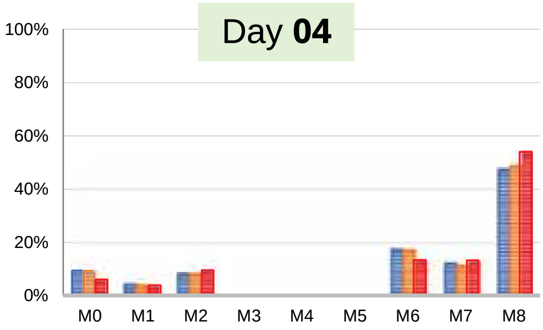


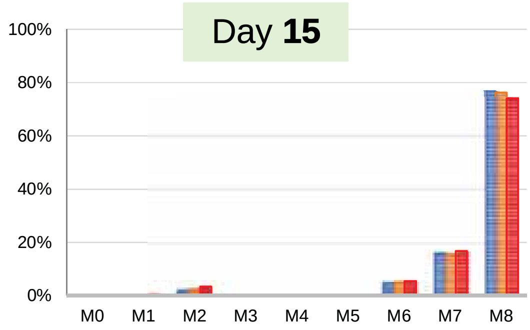


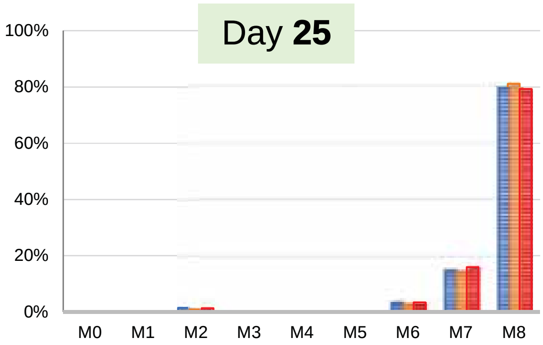


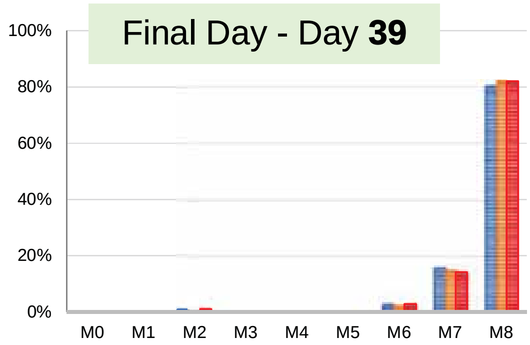


## Fractional $^{13}$C-enrichment

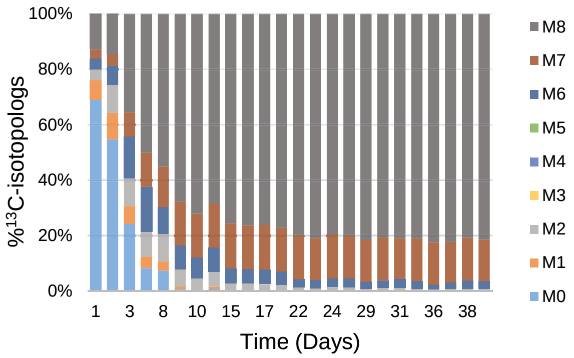


## $^{13}$C-enrichment kinetics

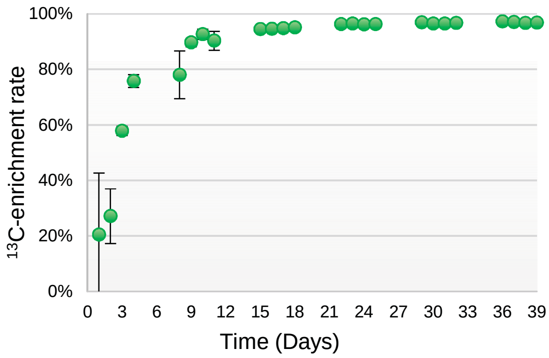

# Identified metabolites in murine urine

*Metabolites from the chemical library*

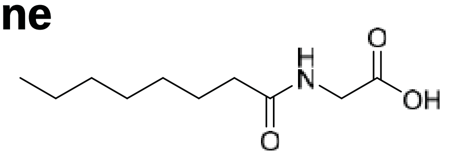


| | |
|---|---|
| **Metabolite** | **Caprylolyglycine**<br>C7H15-CO-Glycine_RT7.7 |
| **Formula** | C10H19NO3 |
| **Exact mass** | 201.1365 |

| | |
|---|---|
| **Ion type** | [M-H]- |
| **m/z** | 200.1292 |

## Isotopic patterns

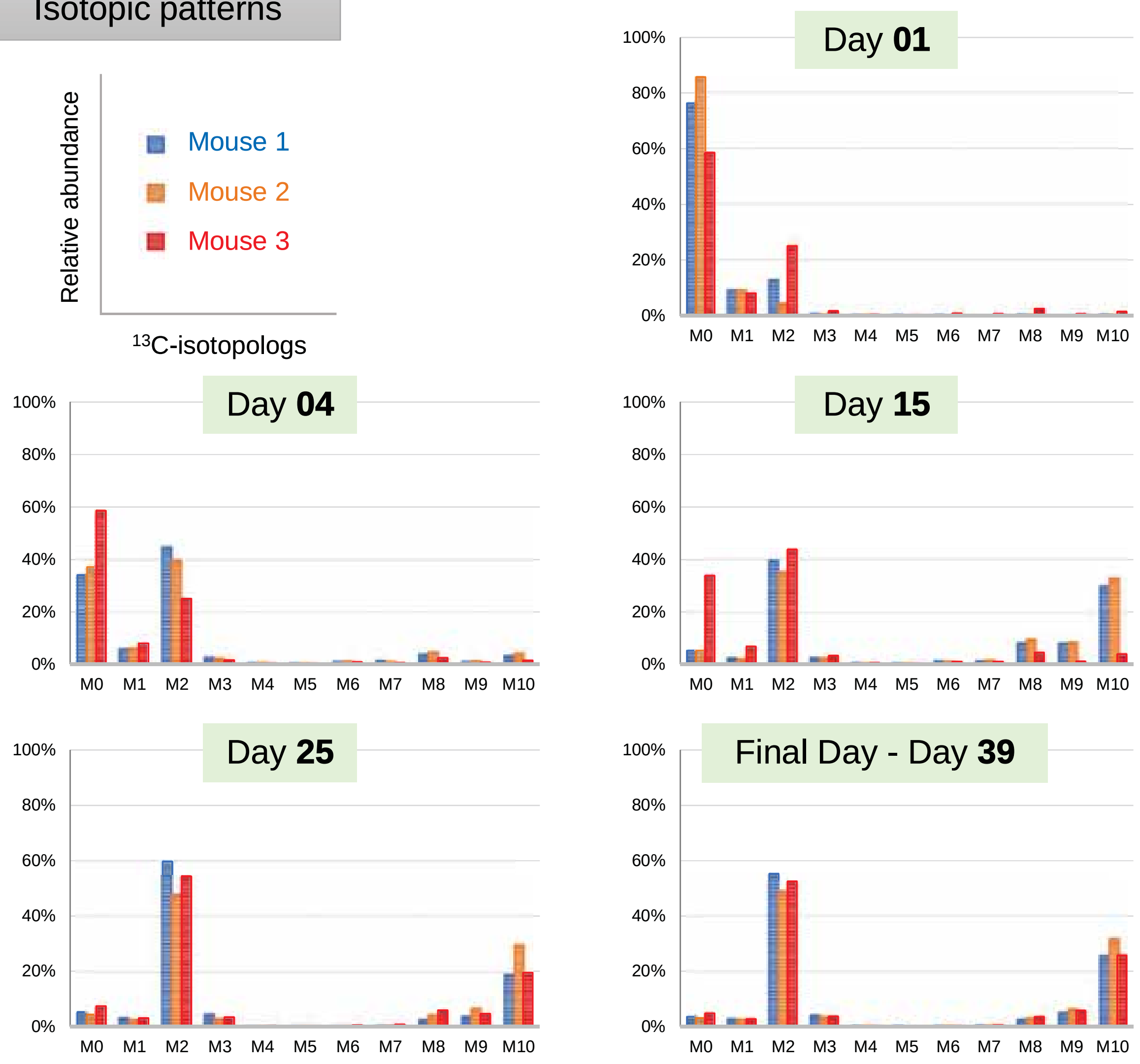

# Identified metabolites in murine urine

*Metabolites from the chemical library*

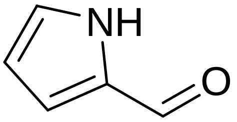


| | |
|---|---|
| **Metabolite** | **Pyrrole-2-carboxaldehyde** |
| **Formula** | C5H5NO |
| **Exact mass** | 95.0371 |

| | |
|---|---|
| **Ion type** | [M+H]+ |
| **m/z** | 96.0444 |

## Isotopic patterns

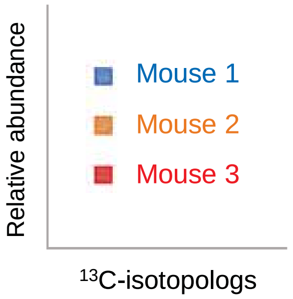


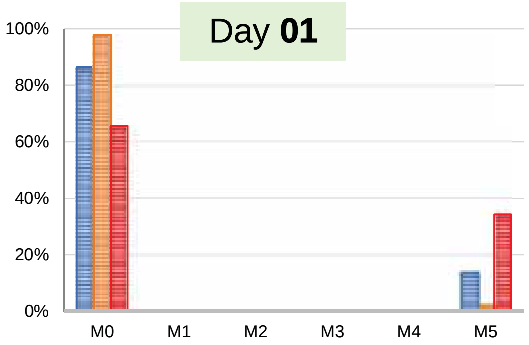


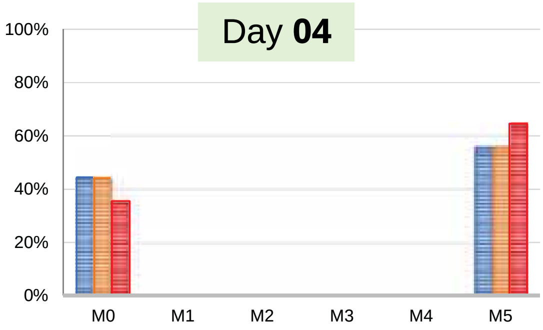


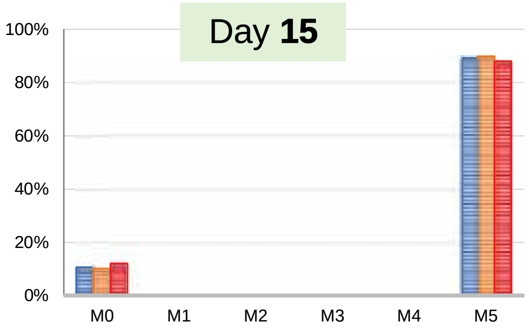


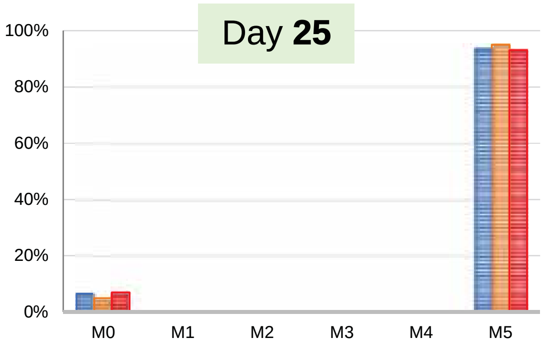


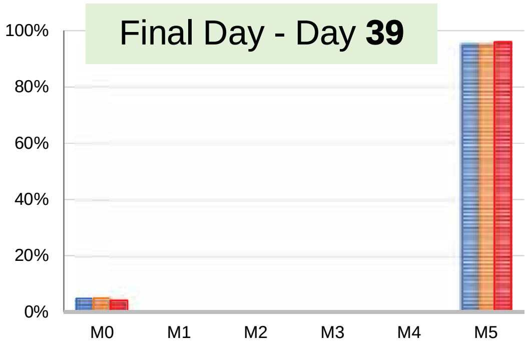


## Fractional $^{13}C$-enrichment

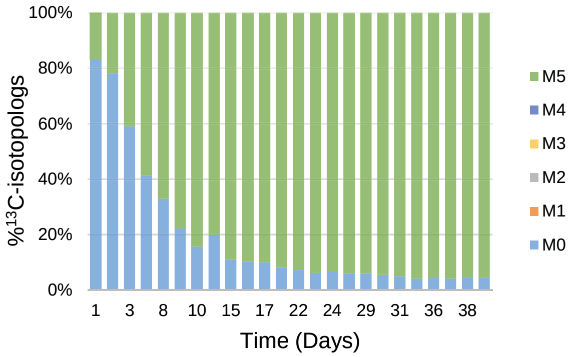


## $^{13}C$-enrichment kinetics

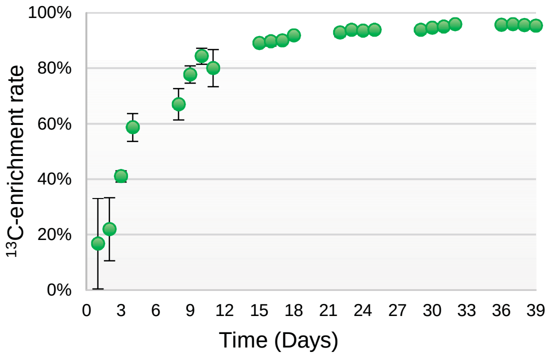

# Identified metabolites in murine urine

*Metabolites from the chemical library*

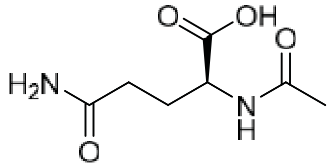

| Metabolite | N-acetyl-L-glutamine |
|---|---|
| Formula | C7H12N2O4 |
| Exact mass | 188.0797 |

| Ion type | [M-H]- |
|---|---|
| m/z | 187.0724 |

## Isotopic patterns

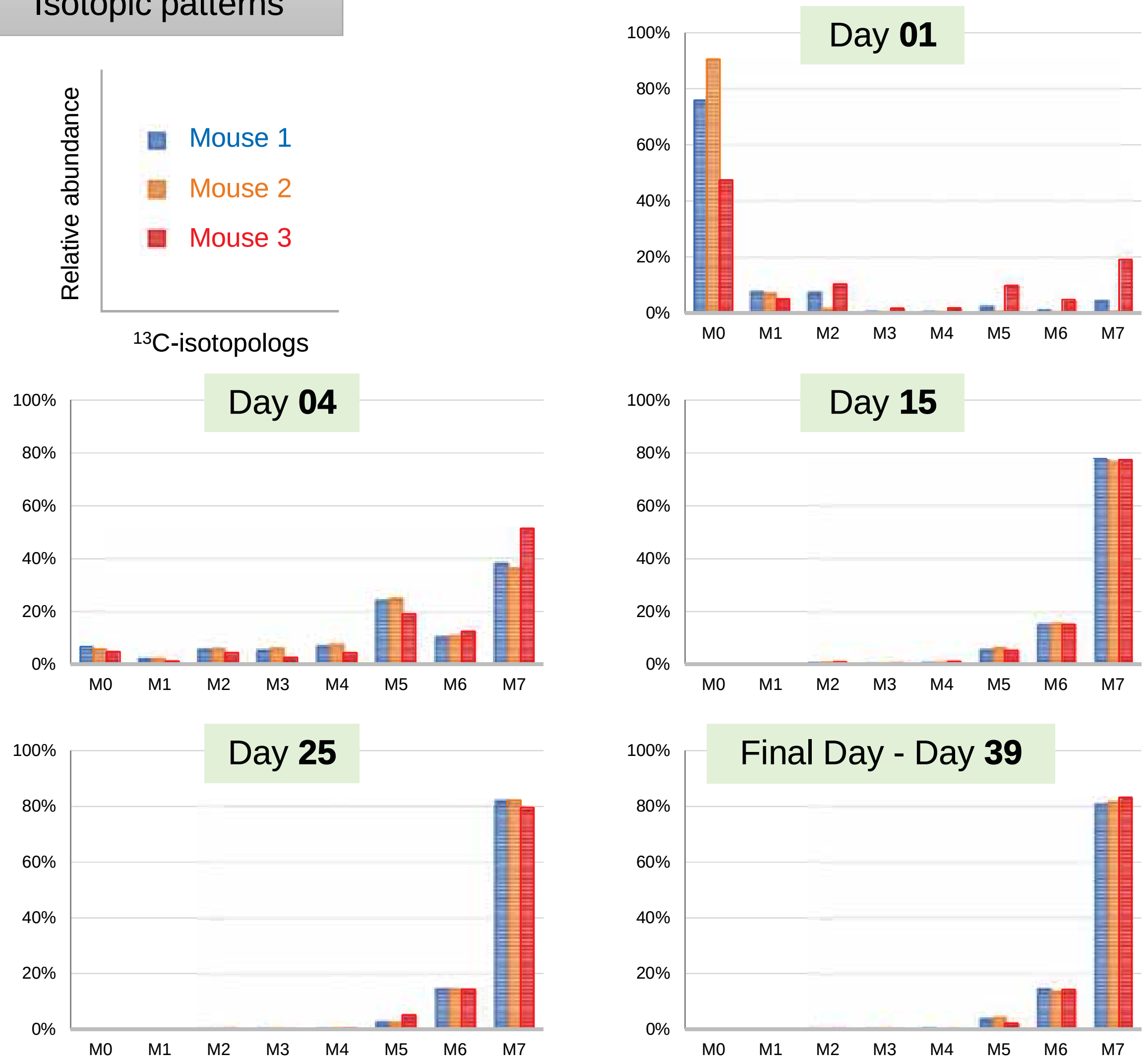


## Fractional $^{13}$C-enrichment

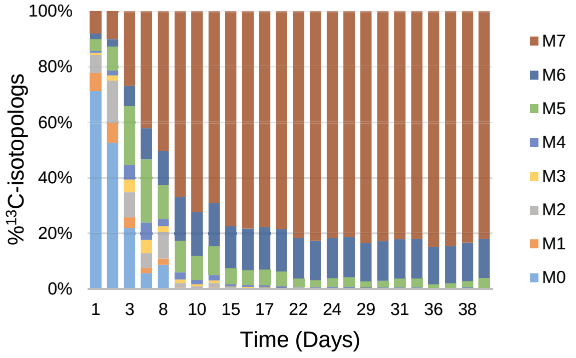


## $^{13}$C-enrichment kinetics

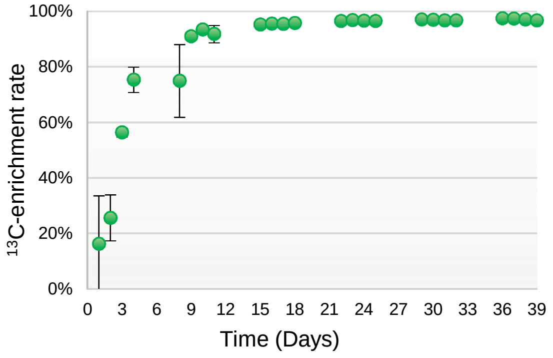

Supplement: Supplementary file 4 — Supplementary Material 4 [file 11306_2025_2391_MOESM4_ESM.pdf]
